# Mapping the emotional face. How individual face parts contribute to successful emotion recognition

**DOI:** 10.1371/journal.pone.0177239

**Published:** 2017-05-11

**Authors:** Martin Wegrzyn, Maria Vogt, Berna Kireclioglu, Julia Schneider, Johanna Kissler

**Affiliations:** 1 Department of Psychology, Bielefeld University, Bielefeld, Germany; 2 Center of Excellence Cognitive Interaction Technology (CITEC), Bielefeld University, Bielefeld, Germany; Universitatsklinikum Tubingen, GERMANY

## Abstract

Which facial features allow human observers to successfully recognize expressions of emotion? While the eyes and mouth have been frequently shown to be of high importance, research on facial action units has made more precise predictions about the areas involved in displaying each emotion. The present research investigated on a fine-grained level, which physical features are most relied on when decoding facial expressions. In the experiment, individual faces expressing the basic emotions according to Ekman were hidden behind a mask of 48 tiles, which was sequentially uncovered. Participants were instructed to stop the sequence as soon as they recognized the facial expression and assign it the correct label. For each part of the face, its contribution to successful recognition was computed, allowing to visualize the importance of different face areas for each expression. Overall, observers were mostly relying on the eye and mouth regions when successfully recognizing an emotion. Furthermore, the difference in the importance of eyes and mouth allowed to group the expressions in a continuous space, ranging from sadness and fear (reliance on the eyes) to disgust and happiness (mouth). The face parts with highest diagnostic value for expression identification were typically located in areas corresponding to action units from the facial action coding system. A similarity analysis of the usefulness of different face parts for expression recognition demonstrated that faces cluster according to the emotion they express, rather than by low-level physical features. Also, expressions relying more on the eyes or mouth region were in close proximity in the constructed similarity space. These analyses help to better understand how human observers process expressions of emotion, by delineating the mapping from facial features to psychological representation.

## Introduction

In daily life, human observers are extremely proficient in recognizing faces, discriminating between them and using them to derive a vast range of information, be it about static features like age, gender or identity, or changeable features like gaze direction, lip movements or emotional states [1,2]. Characteristically, during free inspection of whole faces, observers will focus their gaze on specific parts, with a pronounced tendency to focus on the eye region when trying to distinguish between different emotional expressions [3]. However, there is likewise strong evidence that each basic expression of emotion is associated with a specific set of features [4,5], for example with the eyes important for fear recognition or the mouth for identifying happiness [6–8]. Furthermore, if observers are able to decode this information in an efficient way [9], they should make use of each of these diagnostic features to different degrees, depending on the emotion in question.

In studies where faces are partly masked and observers can only use limited information in each trial, an average face can be reconstructed, reflecting which areas are most strongly associated with a correct response [10,11]. Each emotion expression can thus be described by a different set of diagnostic areas, potentially allowing to distinguish each expression equally well from all others. But configurations of diagnostic areas might also reflect a characteristic pattern of similarities between some emotions, helping to explain certain kinds of systematic confusions between expressions [12,13] and allowing to better understand how the different expressions are represented in the observer’s mind [14]. Charles Darwin [15] in his early research on expression of emotions already postulated that opposing emotions like anger and fear (i.e. fight or flight) will be expressed by the body and face in an antithetical way, and many psychological models tried to map the similarity or opposition of emotions regarding their occurrence and subjective experience [16–18].

Empirical support for some aspects of these models comes from studies on the recognition of expressions, where, for example happiness is recognized the best and confused the least [12,19], in line with the fact that it is the only positive of the basic emotions [20] and hence should be most distinct. Also, numerous reports of systematic mistakes or confusions between expressions demonstrate that anger and disgust [13], as well as fear and surprise [12] are most frequently confused. These emotions are also conceptualized as similar in their subjective experience [16,17]. Regarding the way they are expressed in the face, easily confused expressions can be described by a partly overlapping set of action units [7], with lowered eyebrows in anger and disgust or the raised eyebrows and eye lids in fear and surprise [21], together indicating a similarity of experience, expression and physical appearance.

In the present experiment we therefore asked whether recognition of facial expressions relies on a unique set of features for each emotion, and derived these features from the observers’ recognition performance. Observers were presented with masked faces which were sequentially revealed part-by-part and had to be recognized as fast as possible. Areas of the face whose unmasking most often led to fast and accurate responses were identified for each expression, allowing to subsequently compare the faces and construct a representational space [22,23], in which faces are grouped by how similarly they are perceived. This aims to complement previous studies with masked faces [8,10] by not only reconstructing the visual input that gives rise to correct recognition, but also deriving a numerical metric for each area of the face and combine these areas into patterns that have highest diagnostic value.

## Methods

### Participants

94 student participants took part in the experiment (60 female). Mean age was 24 years (range 18–36). Participants reported no history of neurological or psychiatric illness and had normal or corrected-to-normal vision. They received course credit or 5 EUR as compensation. The study was approved by the ethics board of Bielefeld University (Ethic Statement Nr. 2015–108). All participants gave oral informed consent before taking part in the experiments.

### Face stimuli

Two identities from the NimStim database [24] were used (female model #01 and male model #28). For each identity, facial photographs showing all basic expressions of emotion (happy, sad, angry, fearful, disgusted, surprised) plus the neutral expression were chosen, all with open mouths. Images were cropped, with inner parts of the face occupying most of the image and rescaled to 300x400 pixels. Furthermore, images were manually rotated and shifted to ensure that the faces were as well-aligned with each other as possible and the position of their features comparable across pictures to facilitate similar mask placement.

### Design

For each participant, the experiment consisted of a total of 224 trials (2 face identities times 7 expressions times 16 repetitions per expression).

Each trial consisted of a face masked by a 6x8 grid of white tiles, starting with one randomly revealed tile and subsequently revealing one additional tile every second, in a fully randomized fashion. Participants were instructed to click a stop button below the image as soon as they were able to make a decision regarding the face's expression. After clicking the button, unmasking of the face stopped and participants made a seven-way forced-choice decision between the possible expressions by clicking on one of the respectively labelled buttons. After each decision, participants received feedback about whether they answered correctly.

The experiment was split into two parts of 112 trials, with a pause after half of the experiment to increase participants' comfort. Average time taken to complete the experiment was 50 minutes (range 33–72 minutes). After each half of the experiment, participants received an on-line feedback regarding their speed (average number of revealed tiles needed to make a decision) and accuracy (percentage of correct responses), in comparison to all participants that took part in the experiment so far. This was done to increase participant motivation and emphasize the requirement to respond fast but accurately.

The experiment was programmed using Python 2.7 (www.python.org), Flask 0.10.1 (http://flask.pocoo.org/) and JavaScript and was rendered in a Firefox browser (www.mozilla.org/firefox). The full code to recreate the experiment can be retrieved from the Supplement (S1 Code).

### Analysis

#### Global metrics

To compute a global measure for each face indicating how fast and accurately it was recognized, the percentage of revealed tiles for correct decisions and the percentage of correct responses were computed. Incorrect decisions for each face were also analysed to investigate which kinds of confusions occurred for each expression. Differences between conditions were compared using 95% confidence intervals [25,26], to provide both comprehensive results for all comparisons as well as ensure intelligibility, given the large number of possible comparisons (2 faces x 7 expressions x 2 dependent variables). Differences between conditions were considered significant if the 95% confidence intervals were non-overlappingg.

#### Derivation of metrics for each tile

**Fig 1:**
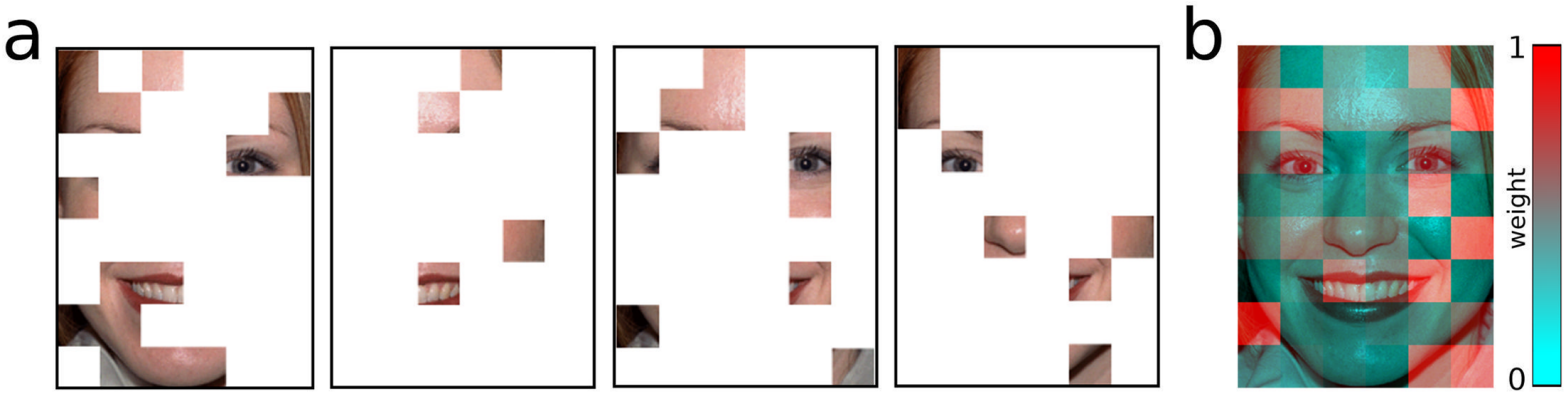
Example of tile weighting. a, an example of four trials with the happy female face, where the unmasking sequence was stopped as illustrated, and a correct answer was given; b, the weights of all tiles for the happy female face, based on 16 trials of one participant. The weights are visualised with a green-red colour spectrum which is min-max-scaled, so lowest weights are green and highest weights are red.

The main metric for the importance of each of the 48 face tiles was computed by a formula in which a tile got a larger weight the more often it was part of short and correct trials. For example, if only one tile is revealed and the participant already stops the sequence, this will results in a high weight for this trial, as computed by the formula “(1-length of trial)/maximal trial length”. These weights were then summed across trials for each participant and expression. The sensitivity of this measure for small differences between trial lengths was further increased by eliminating overly long and unspecific trials form the analysis (25% of the longest trials, which meant eliminating trials with more than 16 revealed tiles). A weight was computed for each tile across trials and averaged for each face (cf. Fig 1). To make weight values easily interpretable, they were transformed into a “percent signal change” measure, by the formula “weight of tile / mean weight of all tiles * 100–100”, resulting in values of 0 for tiles with average weight, negative numbers for values below the mean and positive numbers for values above the mean.

#### Comparison of upper and lower face halves

While the weights for each tile can be analysed independently, they were also aggregated over regions spanning multiple adjacent tiles. The first and most coarse level of aggregation was to divide each face into an upper and lower half (the top 24 and bottom 24 tiles of the face, respectively). Therefore, each face was assigned a difference score (upper minus lower), which was compared to investigate whether the lower of the upper half is more important for recognizing that expression.

#### Comparison across action units

**Fig 2:**
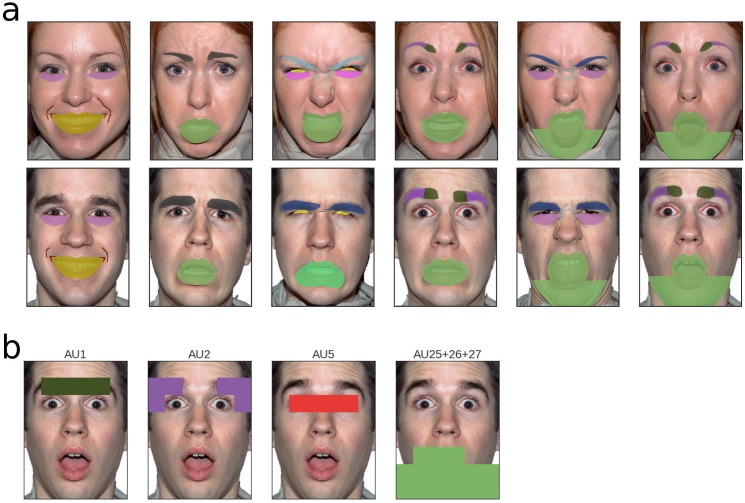
Hand-drawn action units. a, for each of the faces (except the two neutral ones), action units were hand-drawn as defined in the Facial Action Coding System. For example, the last face (male, surprised) has been labelled with action unit (AU) 1 “inner brow raiser” in dark green, AU 2 “outer brow raiser” in purple, AU 5 “upper lid raiser” in red and a compound AU 25+26+27: “lips part”, “jaw drop”, “mouth stretch” in light green; b, the same action units, as assigned to the 48 tiles into which each face is divided; please refer to S1 Fig and S2 Fig for a comprehensive list of labels for each face. Refer to S5 Code for a visualisation of all tile assignments.

The second, more fine-grained level of analysis consisted of aggregating the tile weights by action units. These have been identified by two independent raters (MV and MW), who agreed on a final coding. Then, action units were hand-drawn onto each face (cf. Fig 2A). To subsequently assign each tile to an action unit, an algorithm was written which checks whether a tile contains at least 25 pixels of that hand-drawn action unit. If so, the whole tile is considered as belonging to that action unit, which leads to a relatively liberal assignment, while preventing omissions (Fig 2B). To have a reference against which the AU-based weights can be compared, all tiles in the face which do not belong to any action unit were averaged and their value served as a baseline. Hence, each action unit has a weight which indicates how strongly it differs from the baseline, with positive weights indicating that it is more diagnostic then the non-action unit areas and negative weights indicating that it is less diagnostic.

#### Principal component analysis

For the most fine-grained level of analysis, the values of all individual tiles were used as input to a principal component analysis (PCA), performed with scikit-learn [27]. A matrix was constructed with tile weights as features and participants as observations. All values were normalized to have a mean of zero and standard deviation of one. To illustrate which areas in the face contribute to each principal component (PC), the loading of each face tile on the two first PCs was displayed on the face tiles. For statistical analysis, the values of each expression, as projected onto the two PCs, were compared by means of 95% confidence intervals.

#### Representational similarity analysis

In a similar vein as the PCA, a representational similarity analysis (RSA; [28]) was computed, in which the weights matrix of the 48 tiles of each face was correlated with that of all other 13 faces. This results in a correlation matrix, with the 14 faces are in rows and columns, and the similarity of each possible face pair is indicated by their Pearson correlation. Adopting a rationale popular in brain imaging [22,28,29] the results were presented as dissimilarities (1 minus the Pearson correlation), so a perfect correlation of 1 will give rise to a distance metric of 0 and a perfect negative correlation to a maximum distance of 2. The distances between all faces were then visualized in a simplified 2D space by means of multidimensional scaling (MDS; [28]), carried out using scikit-learn [27]. This allows inspection as to which faces are most similar in the way observers use different face areas to arrive at a correct decision, with dissimilar patterns of features will give rise to larger distances between faces.

As an RSA can be compared between different modalities (e.g. behaviour, brain imaging, performance of a computer vision algorithm [23]), it was also performed on the pixel values of all images, to visualize how the images cluster regarding their low-level properties. For simplicity, the images were grey-scaled so each pixel has only one value instead of three (the RGB channels). Therefore, this additional analysis should be considered tentative, while providing a first approximation of whether the low-level image properties can explain the grouping of faces based on our behavioural data.

## Results

### Global metrics

**Fig 3:**
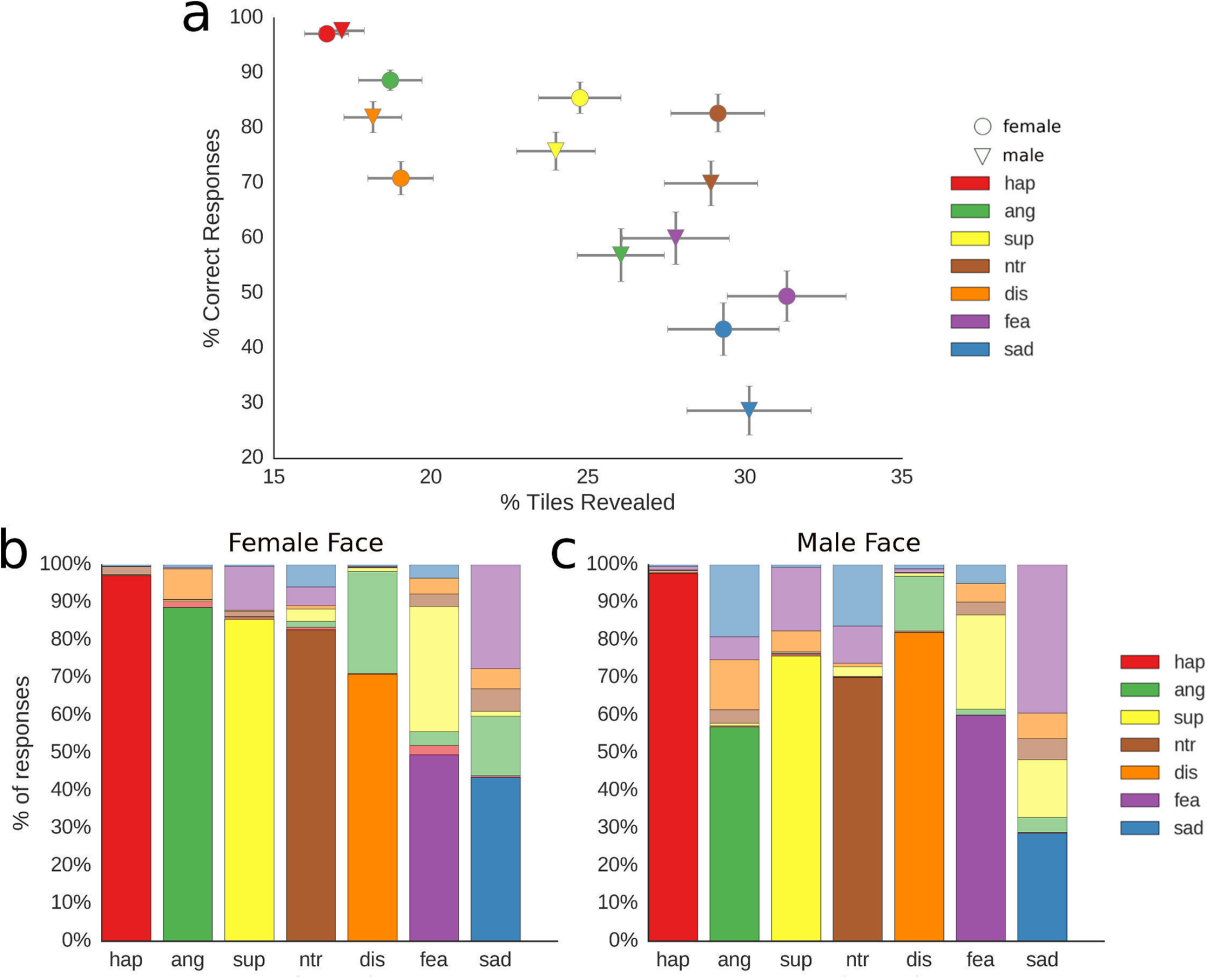
Global metrics for all faces. a, for each condition, the average percentage of revealed tiles needed until a correct response is given is plotted on the x-axis; the average percentage of correct responses in plotted on the y-axis; error bars illustrate 95% confidence intervals; b and c, percentage of all responses for female (b) and male (c) faces, including confusions. Correct responses are plotted in strong colours at the bottom of each bar. Incorrect responses are plotted in muted colours and are at the top of each bar; acronyms: hap, happy; ang, angry; sup, surprised; ntr, neutral; dis, disgust; fea, fear; sad, sad.

Speed and accuracy values for each of the fourteen faces are illustrated in Fig 3A. The plot illustrates that happy faces are recognized significantly more accurately than all other expressions. Overall, the two face models showing the same expression tended to cluster together, although there is a strong deviation from this pattern for the anger expression, which is recognized poorly in the male face. The pattern also shows that sadness is recognized worst, followed by fear.

**Fig 4:**
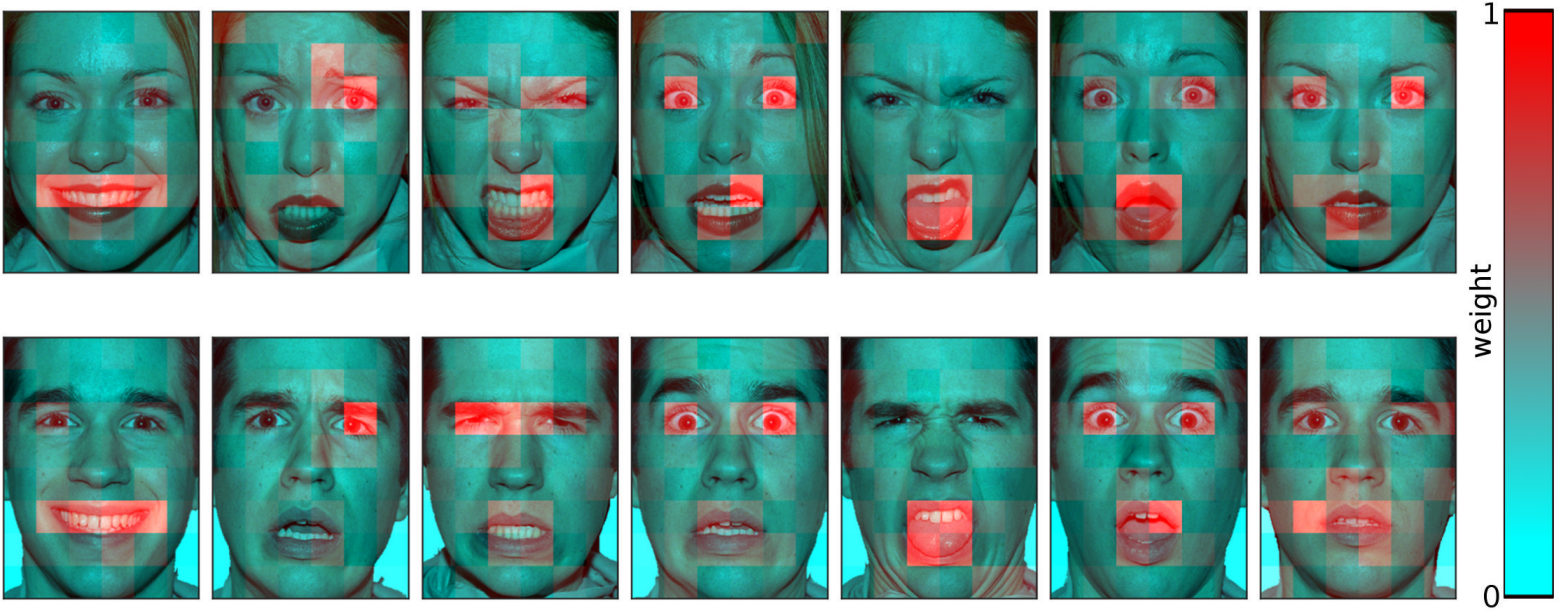
Visual illustration of tile weights for each face. Tile weights, averaged over the whole participant sample. The weights are visualised with a green-red colour spectrum which is min-max-scaled within each face, so lowest weights are green and highest weights are red. These rescaled data are used for visualisation only.

Descriptive statistics were also computed for each face model to illustrate which expressions are most often confused with each other (Fig 4B and 4C). Visual inspection reveals that especially fear and surprise tend to be confused with each other.

### Computing tile-wise weights

For each face, the 48 tiles were assigned weights as described in the methods section. The resulting weights maps are displayed in Fig 4. Descriptively, they indicate that the eyes and mouth regions tend to receive the highest weights. To formally test these differences, a set of statistical analyses are performed, as described in the subsequent sections. Firstly, the tiles are averaged to form meaningful regions (upper and lower face half; action units). Secondly, the tile-by-tile values are used as input for multivariate statistics (PCA, RSA).

### Comparison of upper and lower face halves

**Fig 5:**
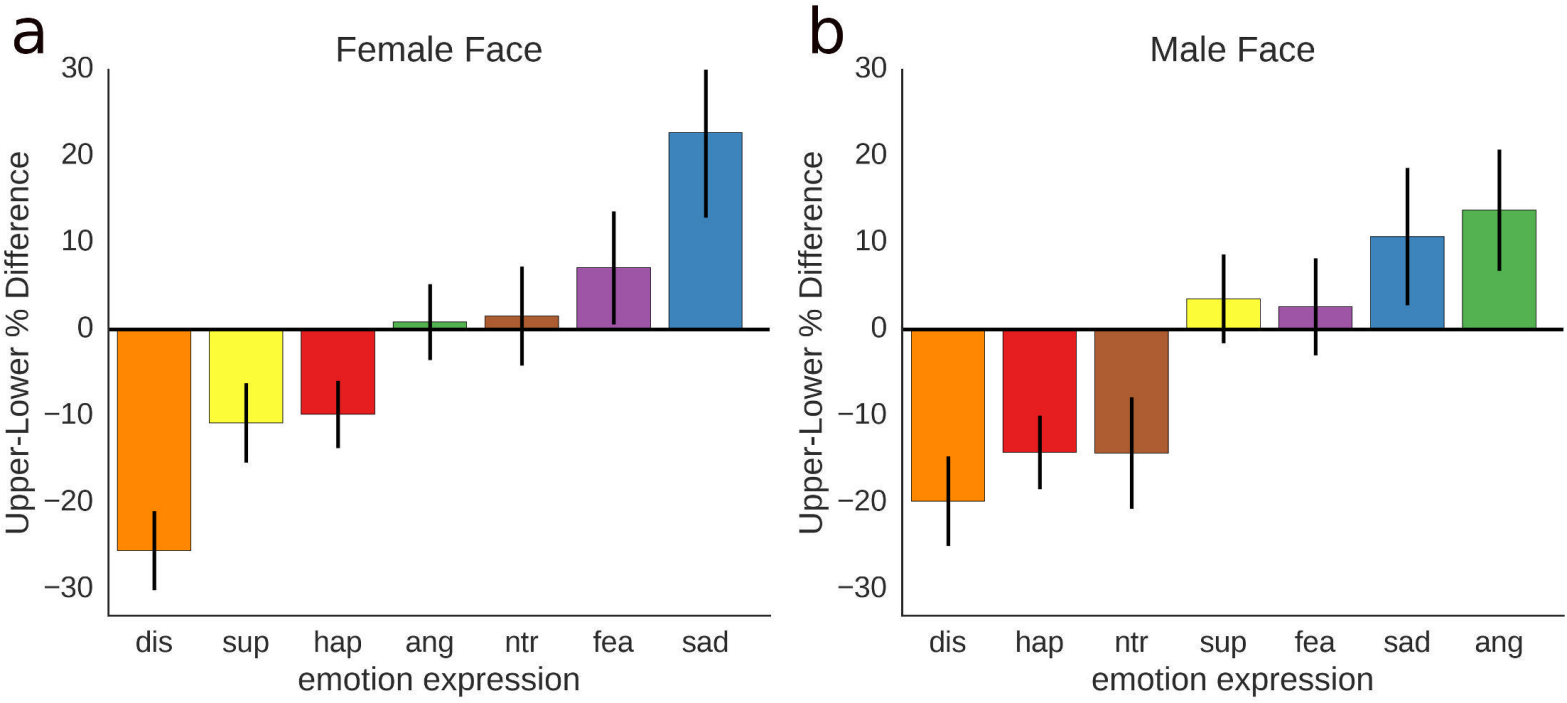
Role of upper and lower face half. Difference score “upper face half” minus “lower face half” for each of the 14 faces used in the experiment, with positive values indicating bigger importance of the upper face half and negative values indicating bigger importance of lower face half; a, for the female face; b, for the male face; error bars represent 95% confidence intervals.

As the difference scores (upper half minus lower half) in Fig 5 illustrate, the disgusted and happy face show significantly highest reliance on the mouth region, while the angry, fearful and sad face show stronger reliance on the eyes, for at least one of the face models.

### Analysis of the role of action units

**Fig 6:**
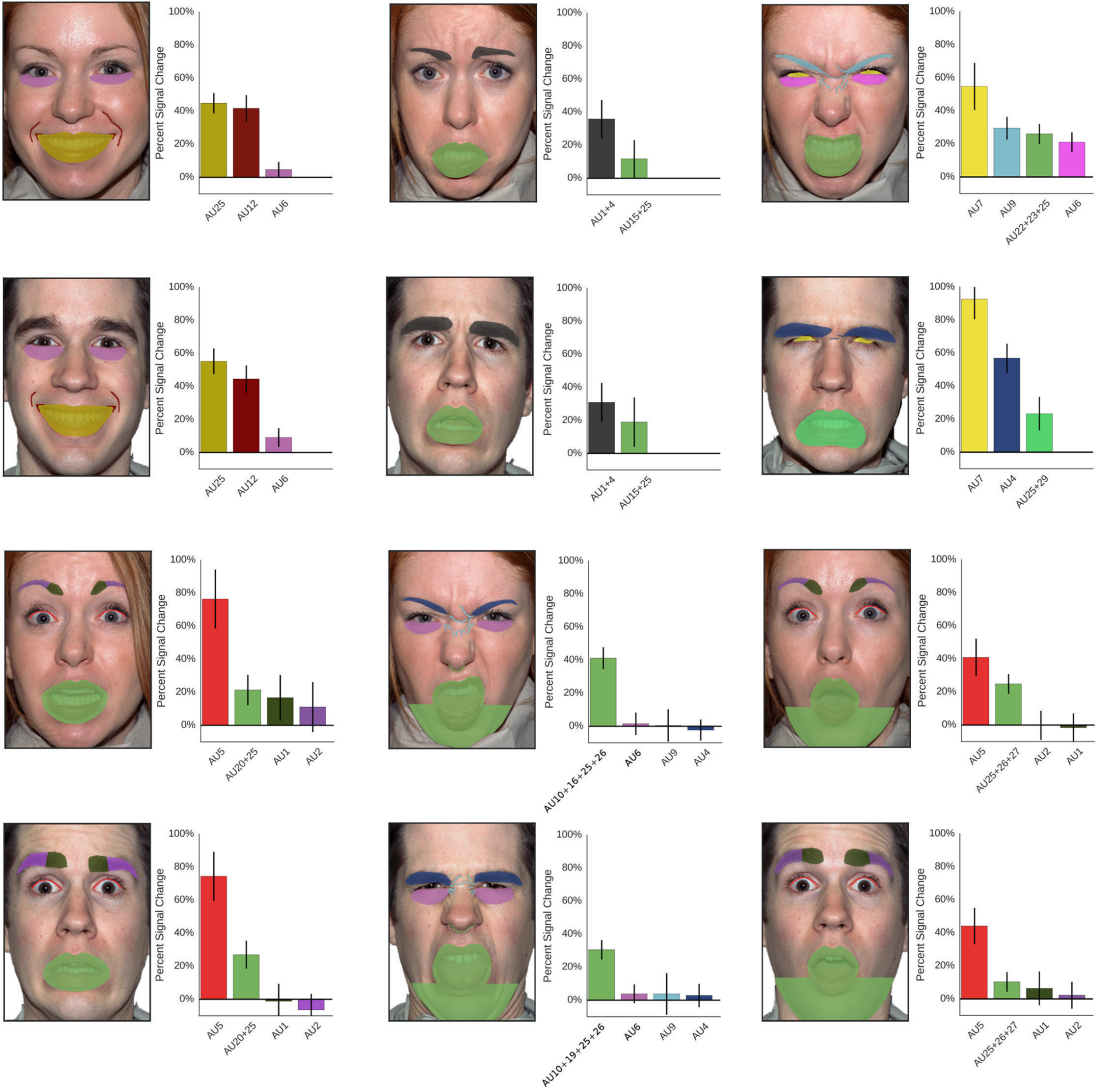
Role of specific action units for emotion recognition. Colours of each action unit as drawn on the face correspond to colours of bars, which are labelled with the respective action unit according to the facial action coding system. Values indicate importance of each action unit as compared to the baseline of all non-action unit tiles. Error bars represent 95% confidence intervals.

The more fine-grained aggregation of tiles by action units reveals that each face has at least one action unit whose tiles have significantly higher weights than the non-action unit baseline (Fig 6). For happiness, this significantly most diagnostic AUs are around the mouth (AU12 and 25, lip corner puller and parting of lips). For disgust, there is also a significant effect for the compound action units around the mouth (including raising the lip and plucking it). This is in line with the above results, showing that the lower face half is most diagnostic for these two expressions.

For fear, surprise, anger and sadness the regions around the eyes have the highest weights, with the lid raiser (exposing the sclera of the eyes; AU5) significantly most important for fear and the lid tightener (AU7) significantly most important for anger. There is a tendency to focus more on the brows in sadness (AU1+4) and both eyes and mouth contribute significantly to the recognition of surprise.

### Principal component analysis

**Fig 7:**
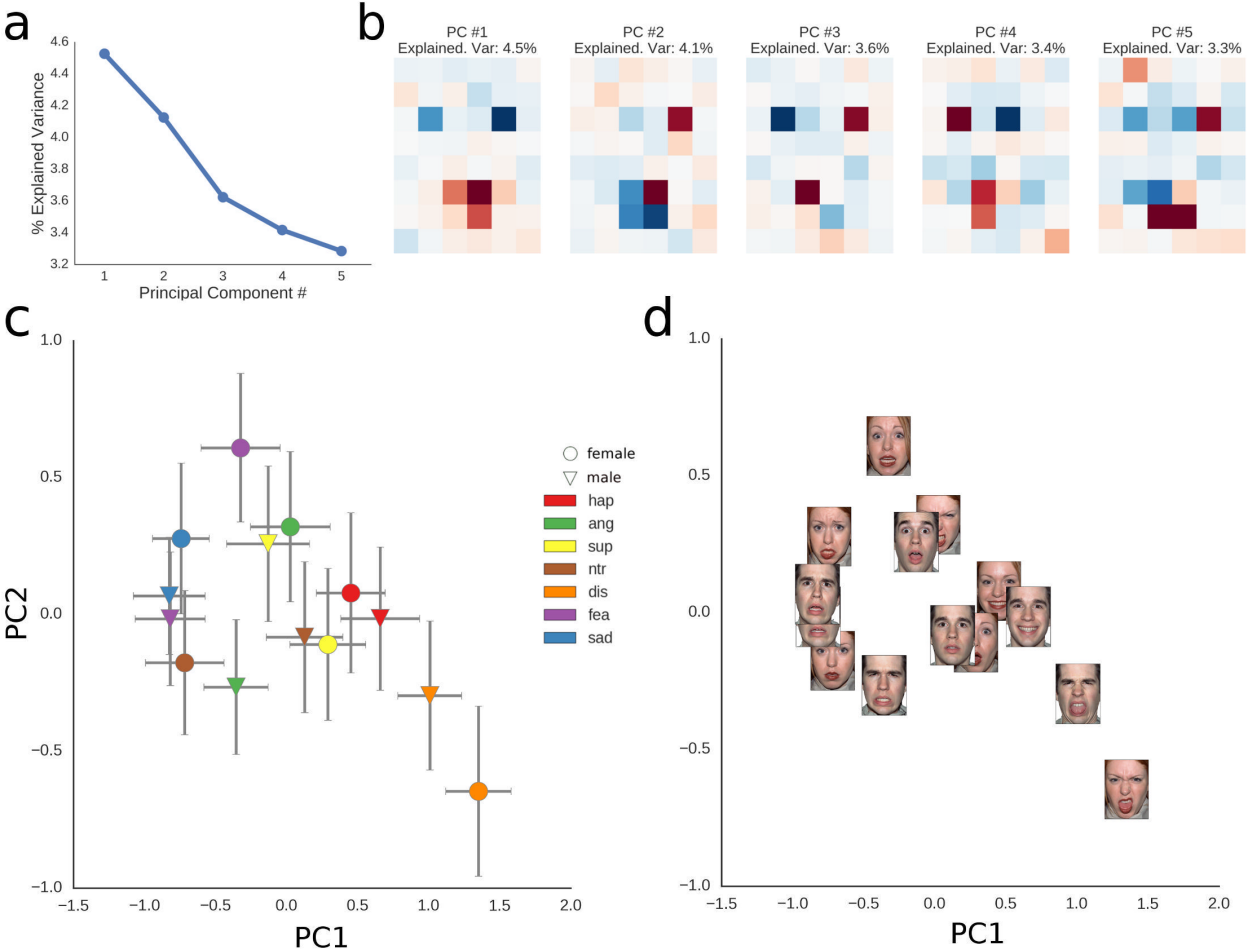
Principal component analysis of face weights. a, percentage of explained variance by the first five principal components; b, visualisation of weights of each principal component in “tile space”, with positive weights in red and negative weights in blue; c, plotting each face in the space defined by the first two principal components, error bars represent 95% confidence intervals; d, same as c, but with the actual stimuli replacing the markers.

The performed PCA reveals that when projecting the PCs into the face space, a face-like pattern emerges for most PCs, with PC1 characterized by high weights around the mouth and low weights around the eyes (Fig 7B). When projecting the data of each participant onto the first two PCs, a pattern emerges in which the faces of the two actors tend to cluster together by emotion. Here, there is a gradient beginning with sadness, passing a more convoluted cluster of anger, fear and surprise, and leading up to happiness and disgust (Fig 7C and 7D). Given that PC1 has high weights around the mouth and low ones around the eyes, these results are well in line with the previous univariate analyses on face halves and action units.

### Representational similarity analysis

**Fig 8:**
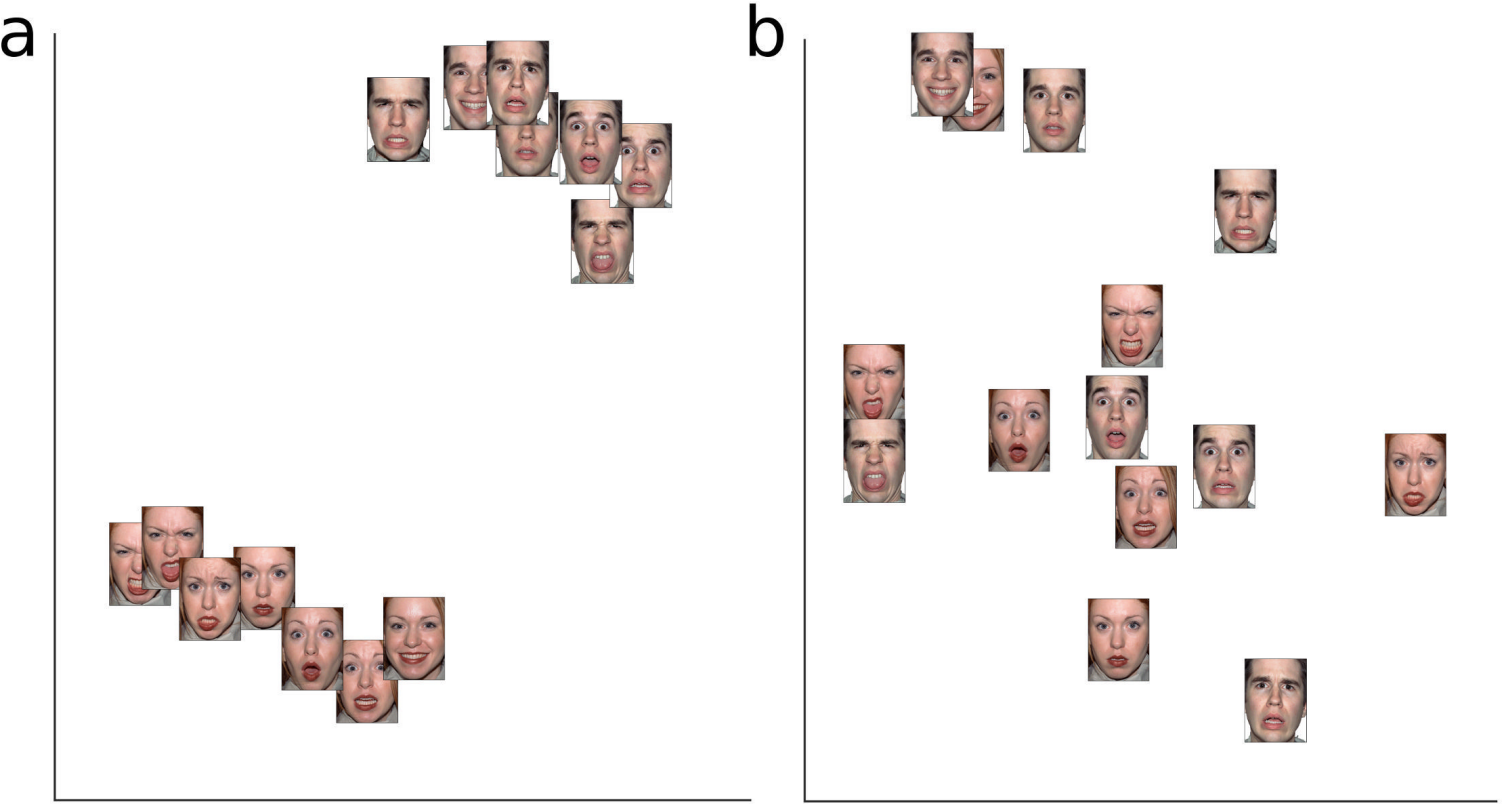
Representational similarity analysis (RSA). Dissimilarity (1—Pearson correlation) for all faces, projected into distances in 2D-space by means of multidimensional scaling; a, RSA with (greyscale) pixel values of images; b, RSA with tile weights from the emotion recognition task; note that axes are not labelled, as they do not represent distinct dimensions and only the distances in 2D space are interpretable.

The RSA results are projected into a 2D space using MDS, which means that the distances only serve as an approximation of the dissimilarities and that the space can be freely rotated, and hence has no axis labels [29]. Fig 8A shows the dissimilarity of images for the low-level pixel values, while Fig 8B illustrates the dissimilarities based on the tile weights from the behavioural data. Descriptively, a pattern emerges in which the pixel values lead to a clustering by identity, while the behavioural data from the experiment lead to a clustering by expressions, with the faces of the two actors well-aligned especially for happiness and disgust. Furthermore, there is a cluster with fearful and surprised faces, which might reflect that these expressions are often confused with one another. Overall, the RSA analysis is in line with the PCA and provides additional descriptive measures for better understanding the similarities in how faces are decoded by the observers.

## Discussion

The present study investigated how human observers use information from different areas of the face to successfully recognize the emotion expressed. We demonstrate that a fine-grained unmasking of faces reveals individual facial features diagnostic for each of the seven expressions. Overall, the eyes and mouth regions turned out to be most diagnostic for recognizing emotions, in line with previous research [8,10,30,31] and emotional expressions could be grouped systematically into “upper-face”and “lower-face” expressions. Furthermore, our analyses identify a systematic pattern in which regions belonging to emotion-relevant action units [21] carry the most diagnostic value for recognizing that expression. Both of these results complement each other, as most of the action units are located in and around the eyes and mouth regions.

The results from both employed multivariate analyses reveal a similarity structure in which expressions are grouped by the features which observers use to successfully recognize them. This emerging similarity structure demonstrates a characteristic grouping of expressions, which reflects multiple properties: For one, it probably reflects the relative importance of the eye and mouth regions in recognizing each expression. This receives support from the analyses of averaged upper and lower face halves, where sad, fearful and angry faces are most often recognized by upper facial features, while happiness and disgust are recognized by information in the lower face half. A similar grouping emerges in the PCA, especially for the first component, whose weights predominantly reflect the influence of mouth and eyes. However, it should be noted that a similar gradient (happy, disgusted, surprised, angry, fearful, sad) can be found in the global metrics of correct responses or number of revealed tiles. This illustrates that the emerging patterns might reflect a more complex combination of features, given how rich facial signals are to begin with [1,2]. Therefore, interpretation of the patterns' meaning warrants caution. Here it is also informative to inspect the results of the similarity analysis, which shows a comparable but not identical grouping. Of note, happiness and sadness again are at the opposing ends of the space, while a cluster in the middle of the space shows fear and surprise expressions in close proximity, which is in line with the fact that these two expressions are often confused, both in the present data set as well as shown in previous studies [12].

Taken together, our results provide a number of important impulses to the research on face recognition:

Firstly, we show that when using a fine-grained masking, the eyes and mouth regions emerge as the overall most diagnostic areas. This is in line with the success of therapeutic interventions in autism, Urbach-Wiethe syndrome and related disorders, where face recognition is impaired and training often focuses on fixating the eyes or mouth [6,32]). It might also inform computer vision algorithms, which perform remarkably well for whole faces [33], but have yet to be shown to perform well when only parts of the face are visible. Of note, on some trials our participants could correctly recognize an expression after only one or two revealed tiles (especially for happiness; cf S8 Code). It can be assumed that under such conditions, most computer vision algorithms would not be able to detect a presence of a face in the first place (cf. [34]). Therefore, the present paradigm could possibly serve as a benchmark for computer vision algorithms when only mouth or eyes are visible. To further investigate the role of eyes and mouth, future behavioural studies could apply the present masking paradigm not to full faces, but to the eyes and mouth regions only (cf. [30,35]). This might allow to increase the resolution of the masking, while working with a manageable amount of tiles, to further delineate which parts of the eyes and mouth explain their high diagnostic value. Since our analyses by action unit are also limited in their interpretability by the relative coarseness of the 48 tiles, such a study might further differentiate between the role of specific action units like the upper lid puller or the lid tightener.

A second important impulse from the present study might come from the similarity analysis, which has been popular for neuroimaging data [22,29]. This method has one important strength when it comes to combining data from different disciplines, such as neuroscience, computer science and psychology: Since the resulting stimulus space is agnostic to the data modality from which it was derived [23], one could for example compare the present results with the emerging similarity space when a computer vision algorithm tries to label the faces [36,37] or to multivoxel responses of brain areas along the ventral stream, especially fusiform gyrus and superior temporal gyrus [2,38].

Thus, the neural bases of facial expression recognition could be further elucidated by analysing at which stage of the ventral stream hierarchy a similar grouping of faces emerges.

Also, using behavioural and brain data, one might also address the face processing of some patient groups [6,32]. Here, the construction of a similarity space could prove useful in multiple regards: By analysing which faces cluster together, the RSA could provide a first assessment of individual deficits, showing which faces are most readily confused or perceived as too similar. These faces could be targeted with a tailored training program, and the program's success monitored by visualising whether the distance of these faces in the similarity space can be increased.

However, given that only one identity per gender was used in the present experiment and that the way some of the expressions were displayed might not have been prototypical enough, the present paradigm would benefit from replication with new stimulus material. This certainly concerns the expressions of sadness and anger, which were poorly recognized, varied most strongly between the male and female models and whose action unit labelling deviated somewhat from the reference guide [7,21].

Nevertheless, a third important impulse from the present study is the demonstration that using a relatively fine-grained mask of 48 tiles and only very few trials (16 per face) is feasible and gives robust results. One reason for this might be the pseudo-adaptive nature of the experiment, where each trial can be stopped by the participant at any point, so each one will contain subjectively diagnostic information. Also, the dynamic unmasking gives rise to a very rich set of metrics, which the present analyses only began to tap into. These could be capitalized on in further studies, for example by analysing the order in which the tiles are unmasked. Especially, using the last tile revealed (hence, the one which triggers the response) could provide complementary information about the importance of single face areas. One future challenge might be to develop new algorithms to combine the behavioural data into a more comprehensive measure of face recognition.

Overall, the present study confirms the high importance of the eye and also the mouth region for successfully recognizing expressions of emotion, which is in line with previous masking studies [8] and the work on facial action units [4,7]. It demonstrates that a relatively high-resolution masking of faces can lead to robust results even with few trials and allows to compare the importance of each face area between different expressions. Finally, it shows that a similarity space of facial expressions can be constructed, illustrating the commonalities between the perception of different faces. Thus, the present study provides a first approximation of a representational map of emotion expressions in the observer's mind.

## Supporting information

S1 Fig Action unit assignment for all expressions of female face. (TIFF)

S2 Fig Action unit assignment for all expressions of male face. (TIFF)

S1 Code All files for recreating the experiments. (ZIP)

S2 Code Data import. (HTML)

S3 Code Global metrics for face recognition. (HTML)

S4 Code Assigning weights to all face tiles. (HTML)

S5 Code Metric computation by face regions (action units and face halves). (HTML)

S6 Code Principal component analysis of face recognition. (HTML)

S7 Code Representational similarity analysis of face recognition. (HTML)

S8 Code Interactive reconstruction of every trial of every participant. (HTML)

S9 Code Interactive plotting of action units for correct and incorrect decisions. (HTML)

S10 Code All code files in executable iPython Notebook format. (ZIP)
