# Supplementary figures and images for: Mapping the emotional face. How individual face parts contribute to successful emotion recognition
Source: PLoS One. 2017 May 11;12(5):e0177239. doi: 10.1371/journal.pone.0177239 (PMC5426715; doi:10.1371/journal.pone.0177239)

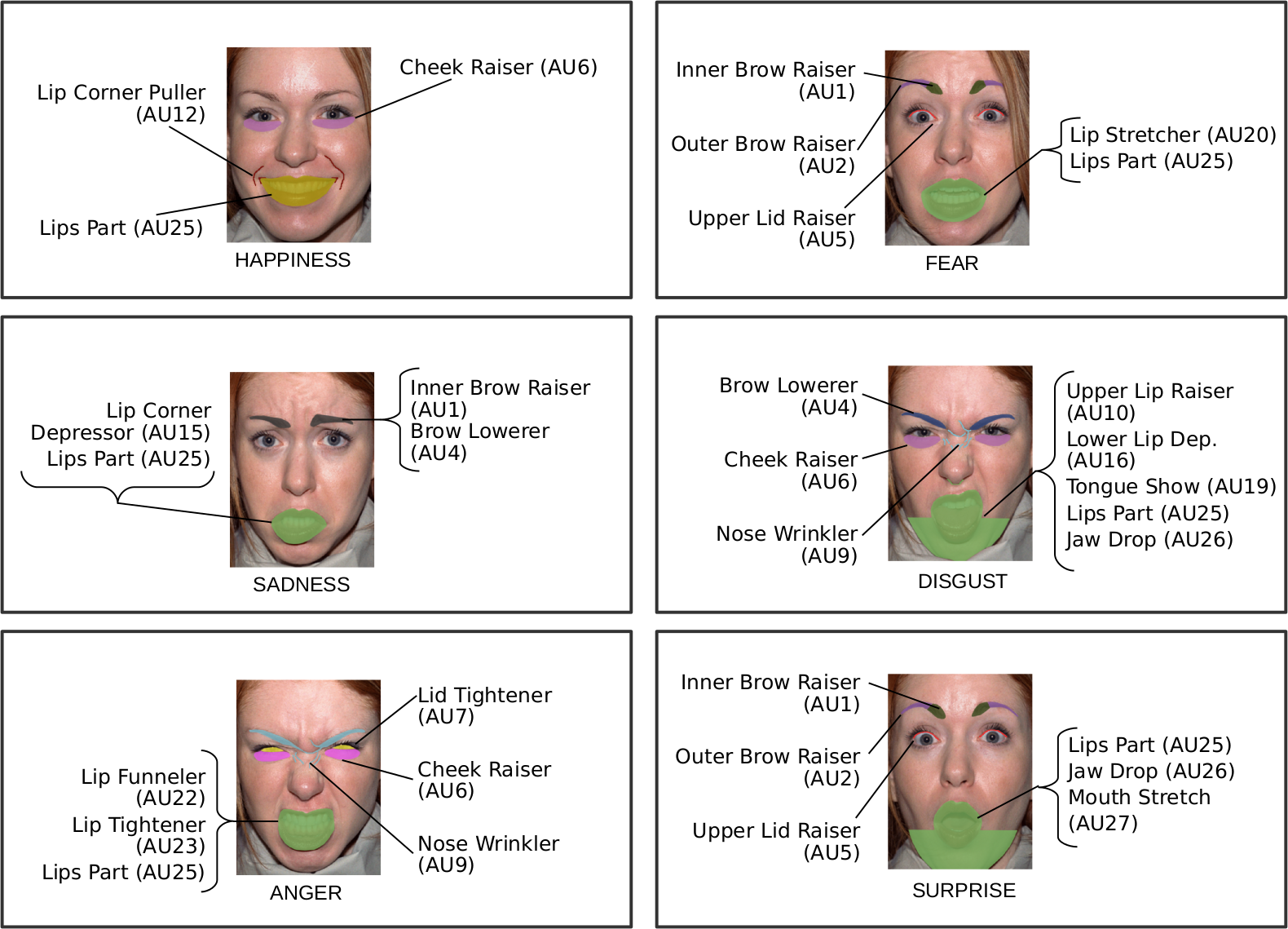

Supplement: S1 Fig — (TIFF) [file pone.0177239.s001.tiff]

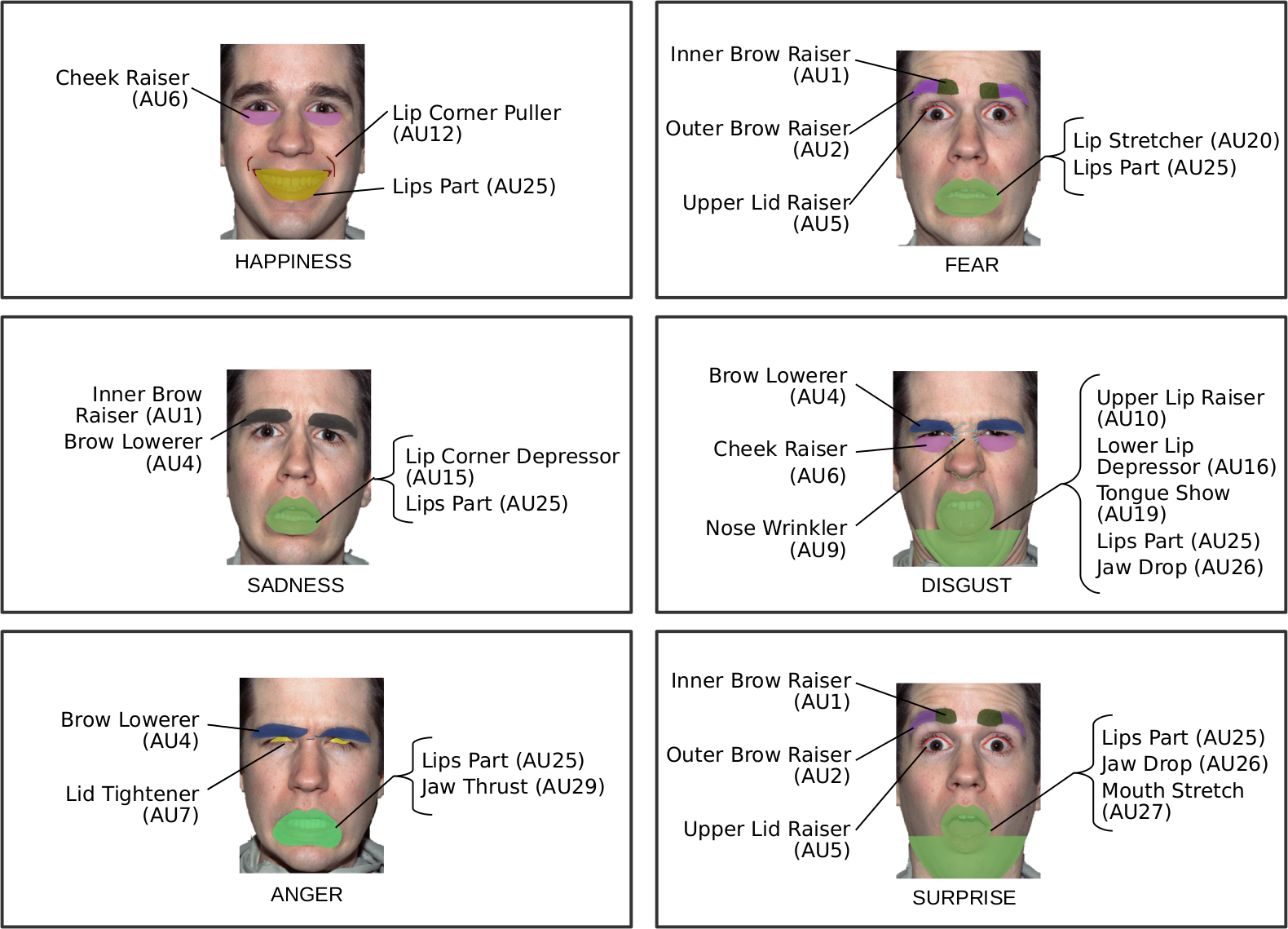

Supplement: S2 Fig — (TIFF) [file pone.0177239.s002.tiff]

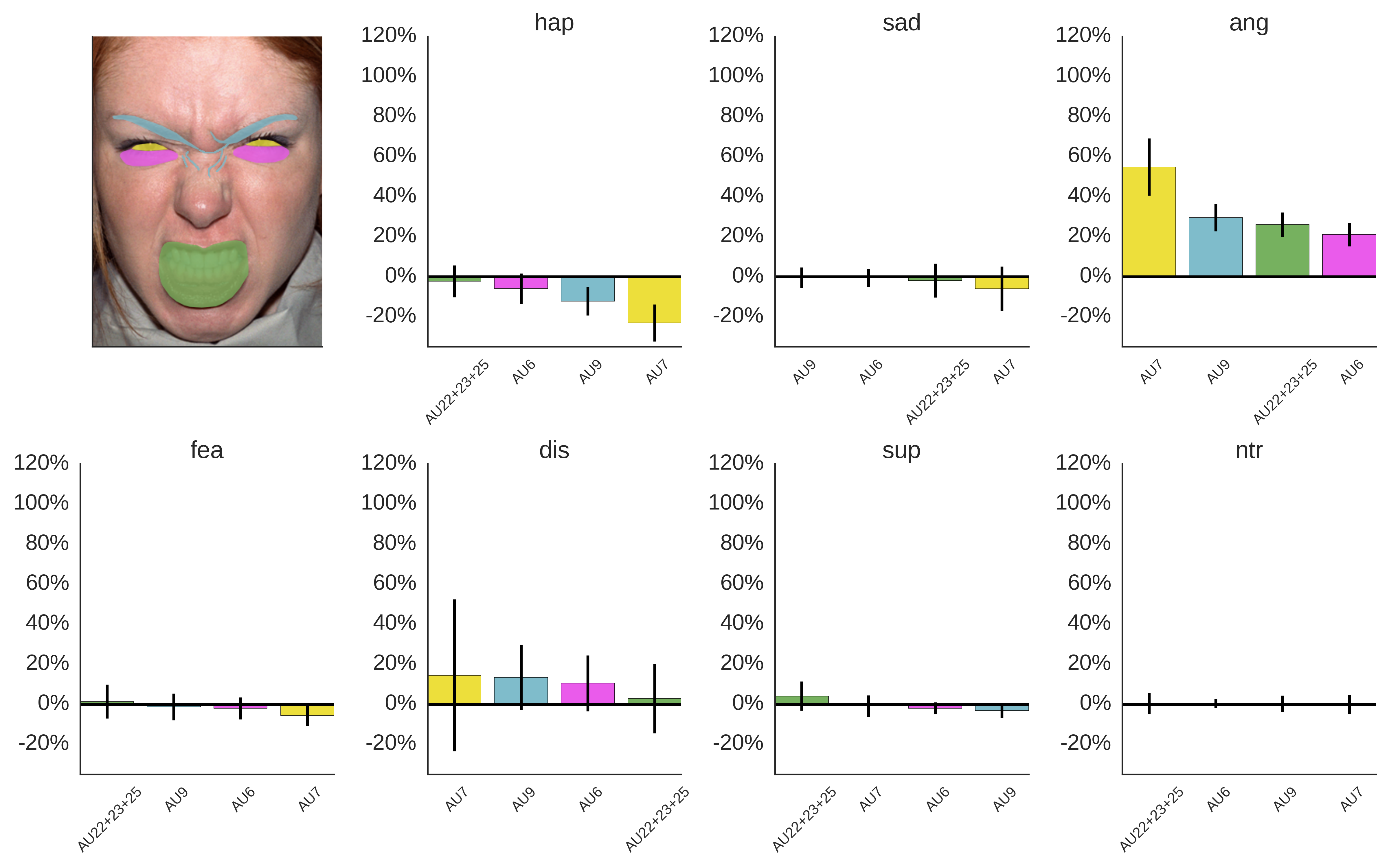

Supplement: S10 Code — (ZIP) [file pone.0177239.s012.zip › mappingTheEmotionalFace-master/auConfusions/confPlot_f_ang.png]

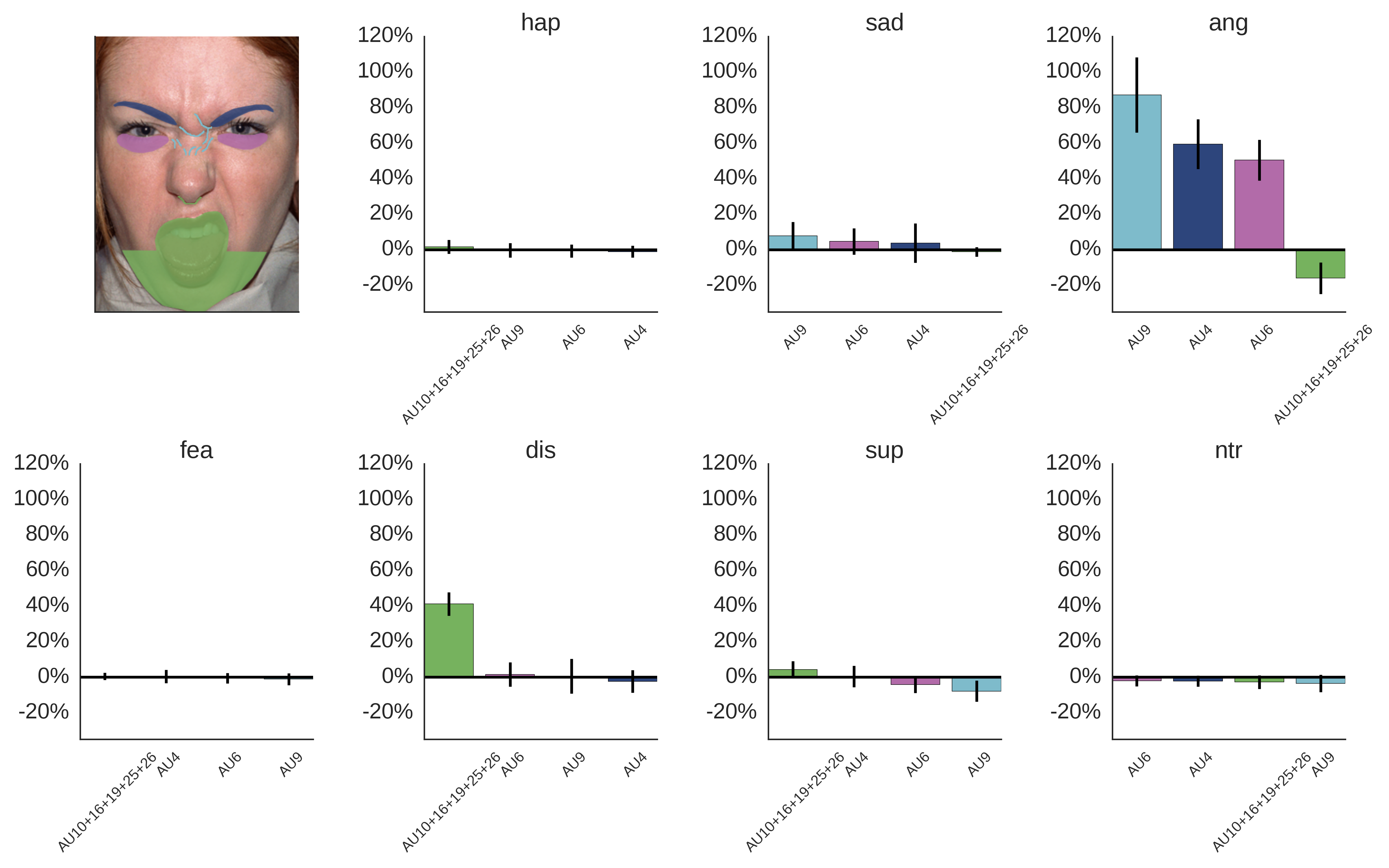

Supplement: S10 Code — (ZIP) [file pone.0177239.s012.zip › mappingTheEmotionalFace-master/auConfusions/confPlot_f_dis.png]

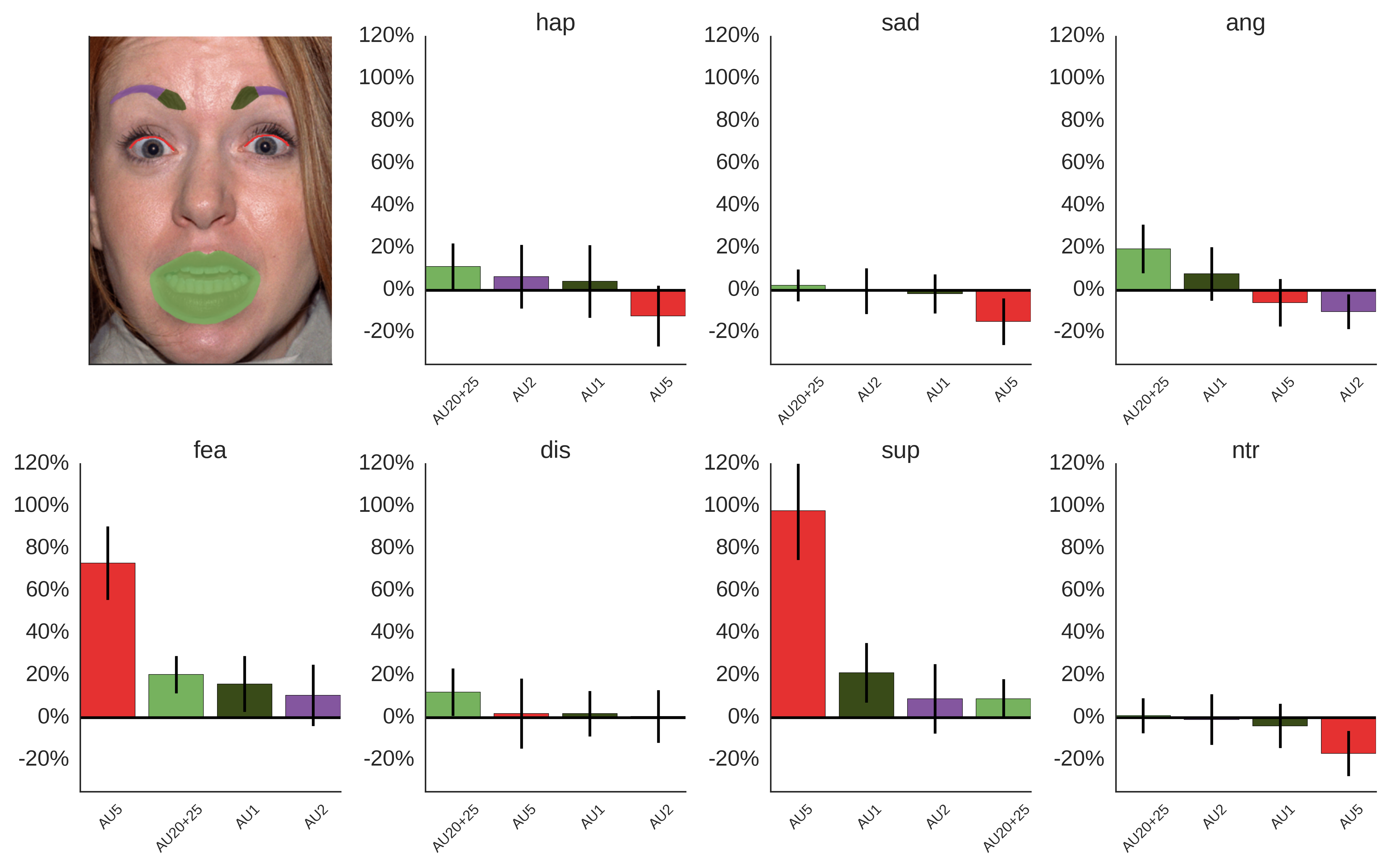

Supplement: S10 Code — (ZIP) [file pone.0177239.s012.zip › mappingTheEmotionalFace-master/auConfusions/confPlot_f_fea.png]

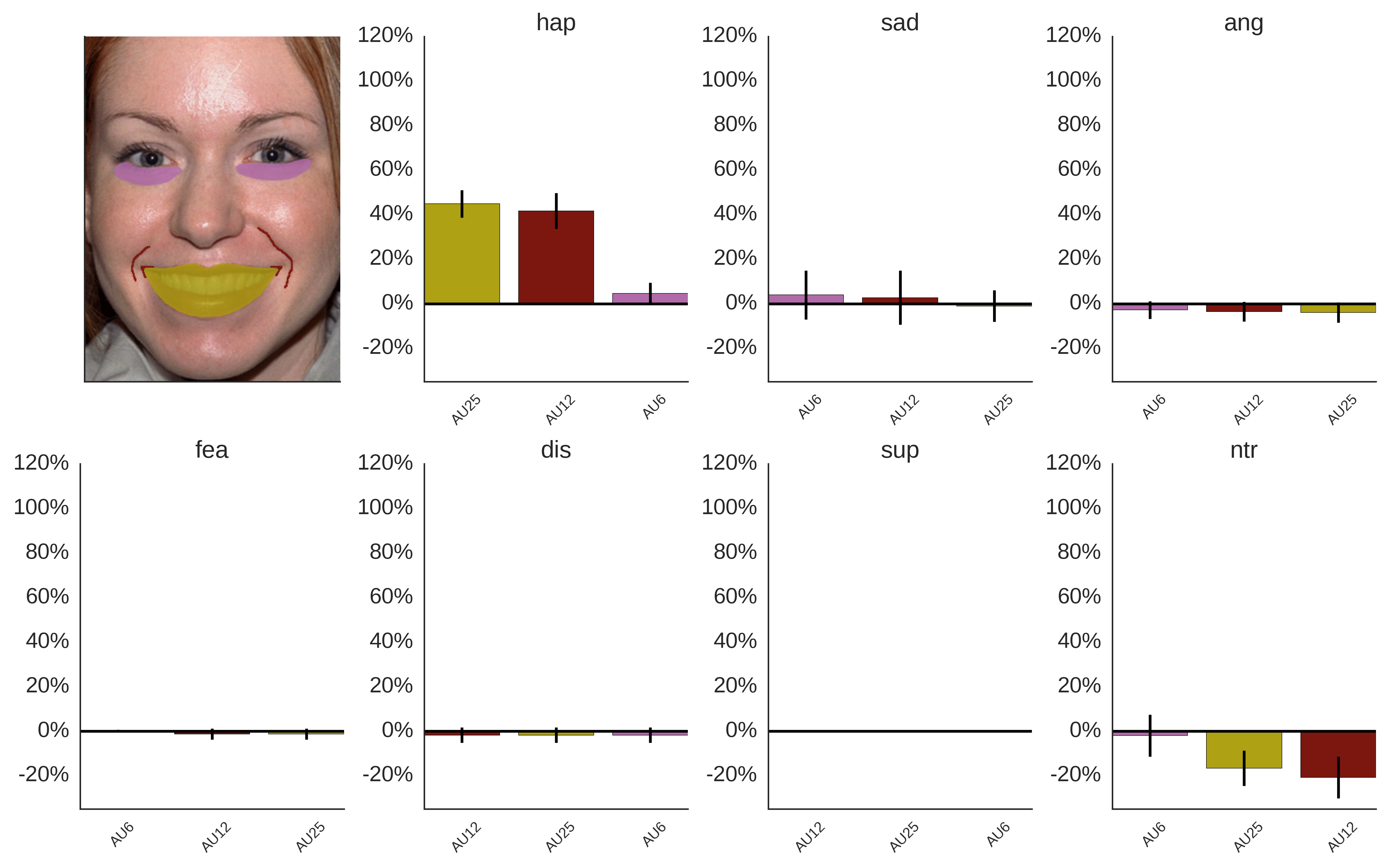

Supplement: S10 Code — (ZIP) [file pone.0177239.s012.zip › mappingTheEmotionalFace-master/auConfusions/confPlot_f_hap.png]

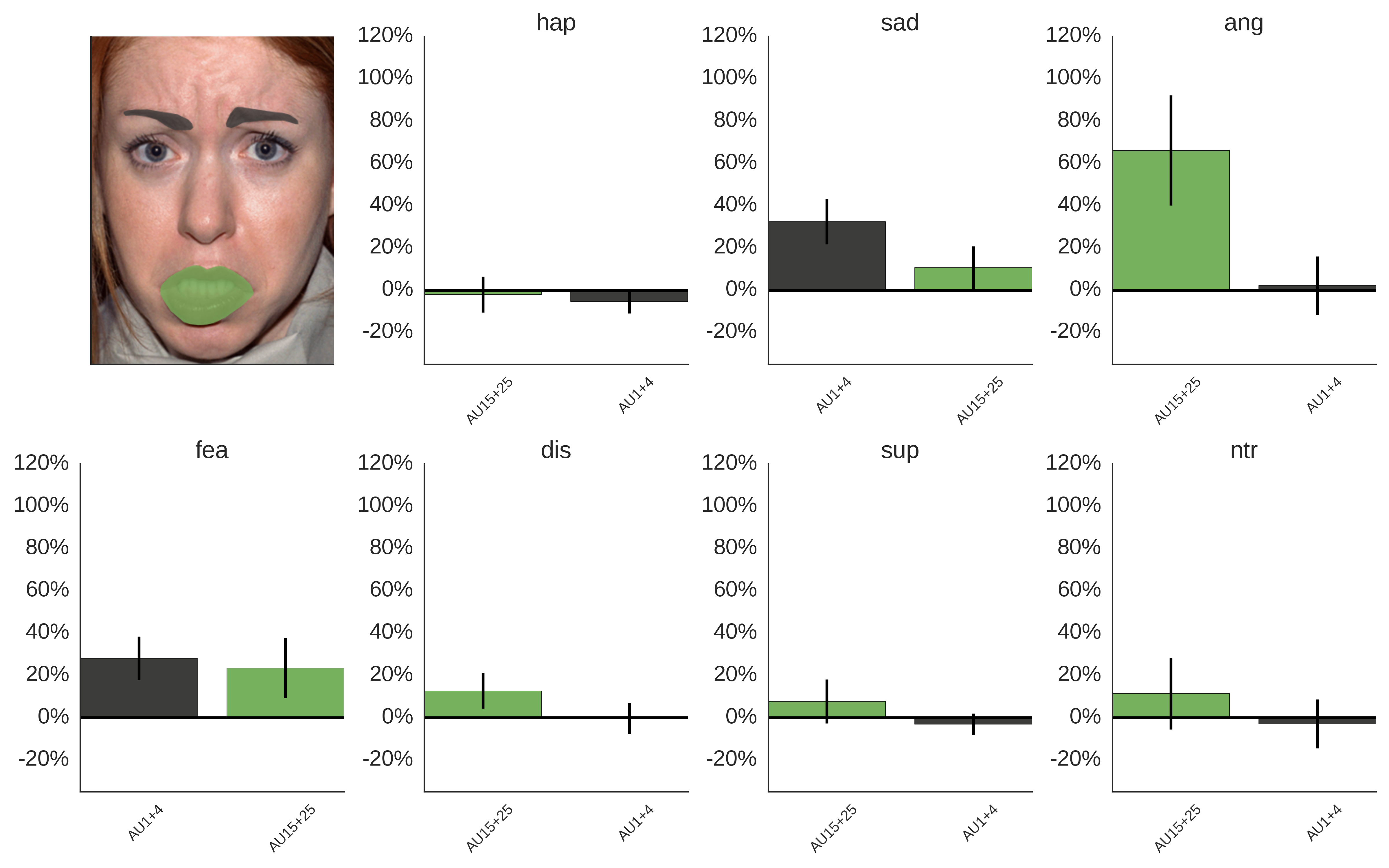

Supplement: S10 Code — (ZIP) [file pone.0177239.s012.zip › mappingTheEmotionalFace-master/auConfusions/confPlot_f_sad.png]

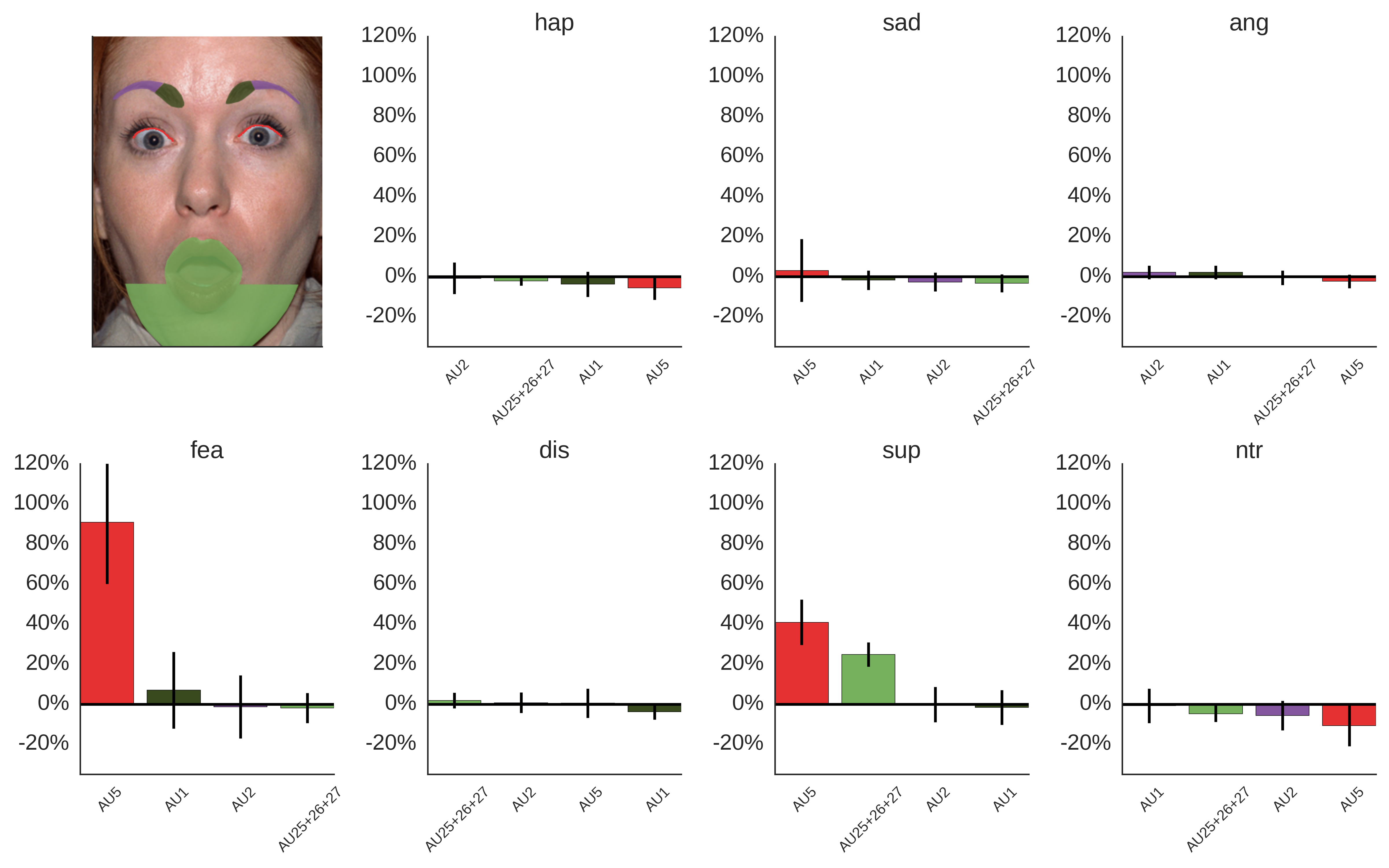

Supplement: S10 Code — (ZIP) [file pone.0177239.s012.zip › mappingTheEmotionalFace-master/auConfusions/confPlot_f_sup.png]

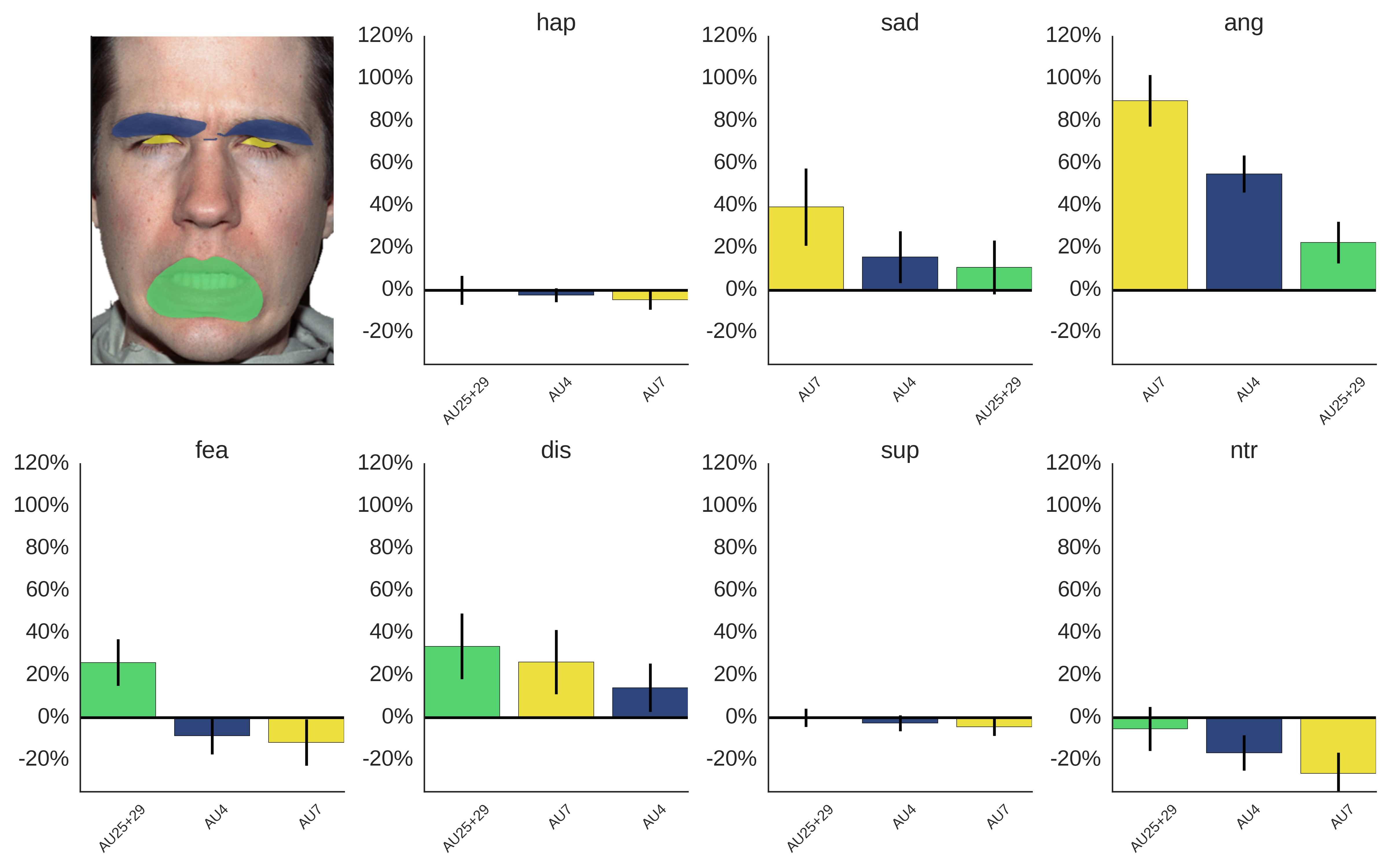

Supplement: S10 Code — (ZIP) [file pone.0177239.s012.zip › mappingTheEmotionalFace-master/auConfusions/confPlot_m_ang.png]

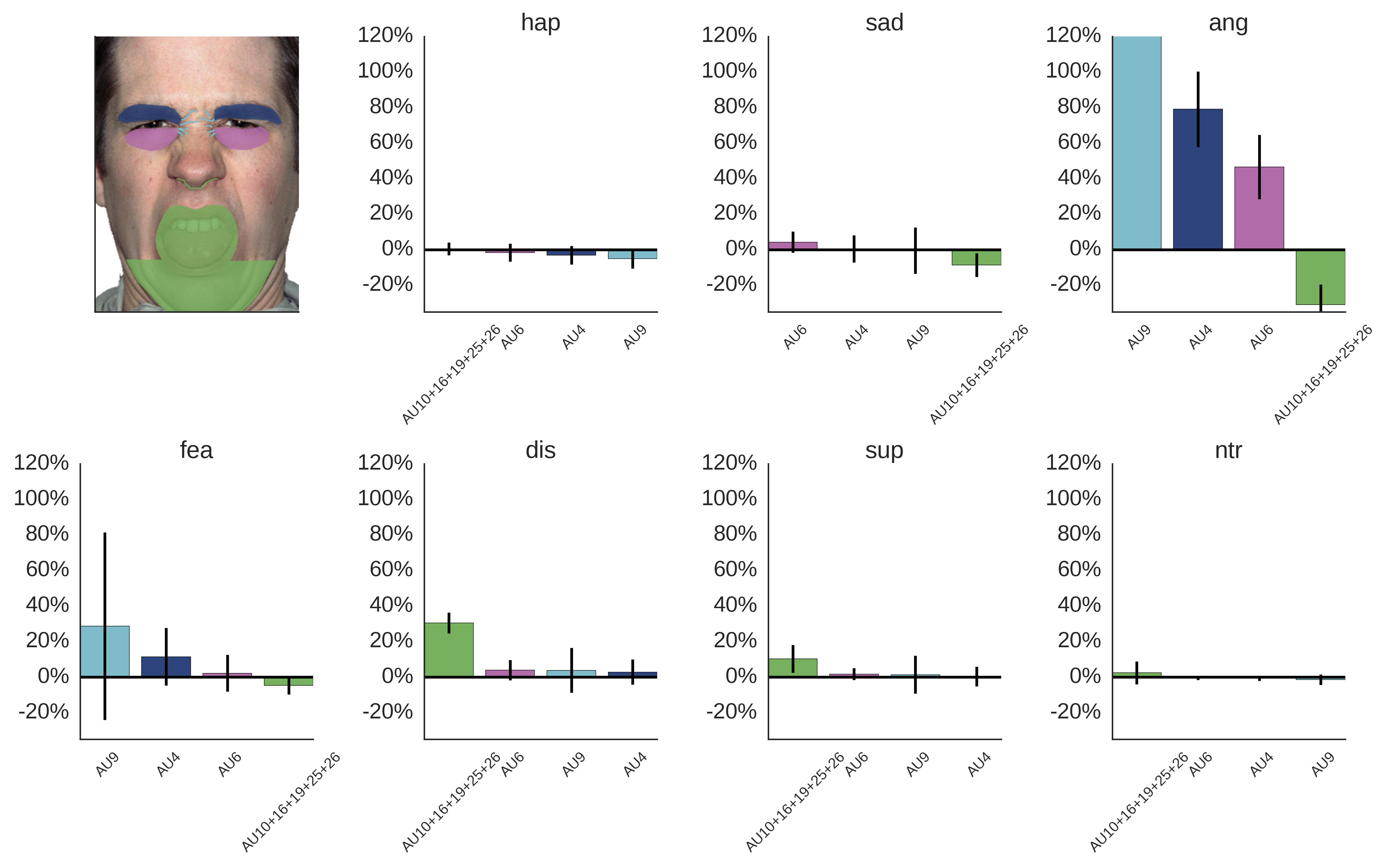

Supplement: S10 Code — (ZIP) [file pone.0177239.s012.zip › mappingTheEmotionalFace-master/auConfusions/confPlot_m_dis.png]

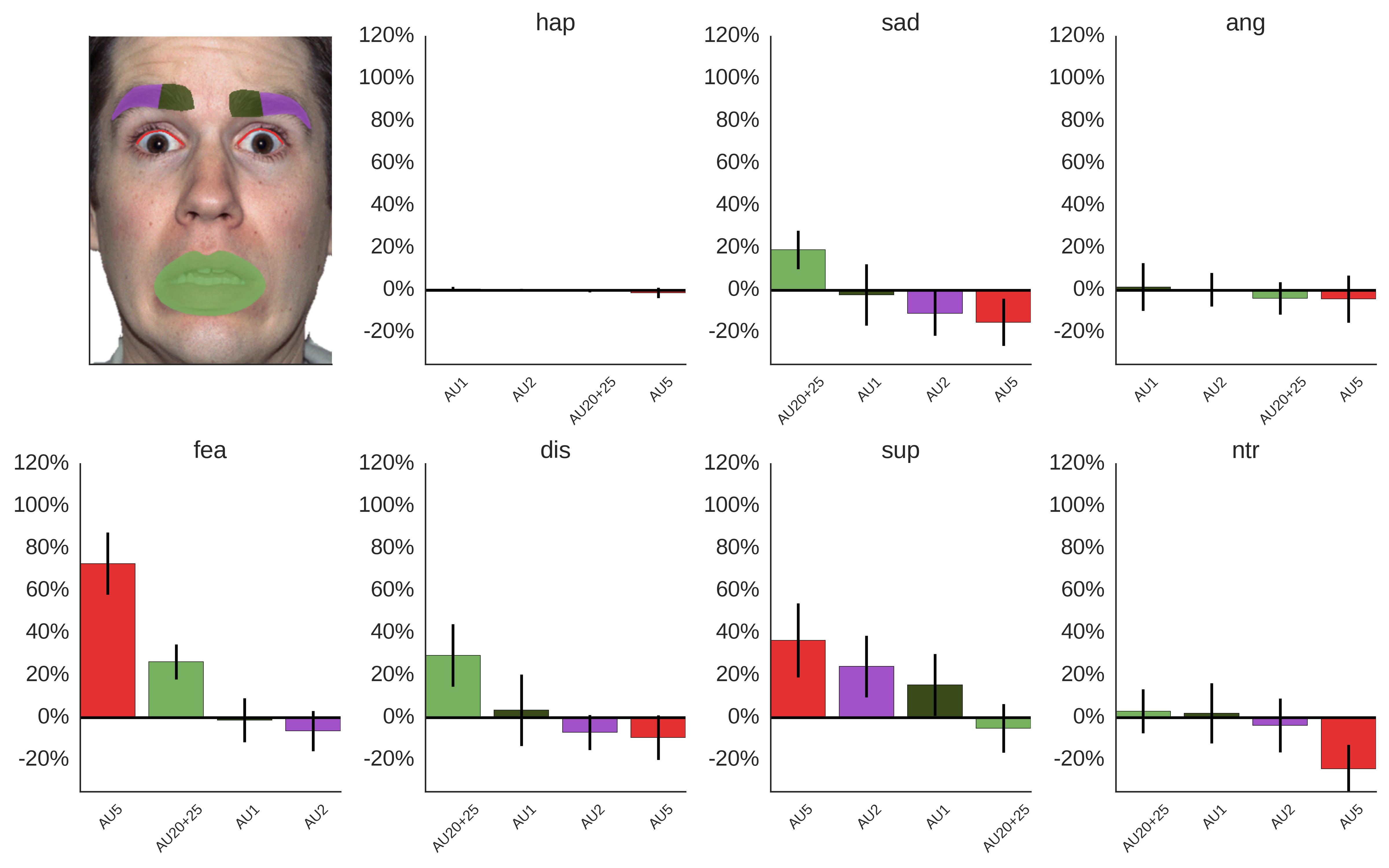

Supplement: S10 Code — (ZIP) [file pone.0177239.s012.zip › mappingTheEmotionalFace-master/auConfusions/confPlot_m_fea.png]

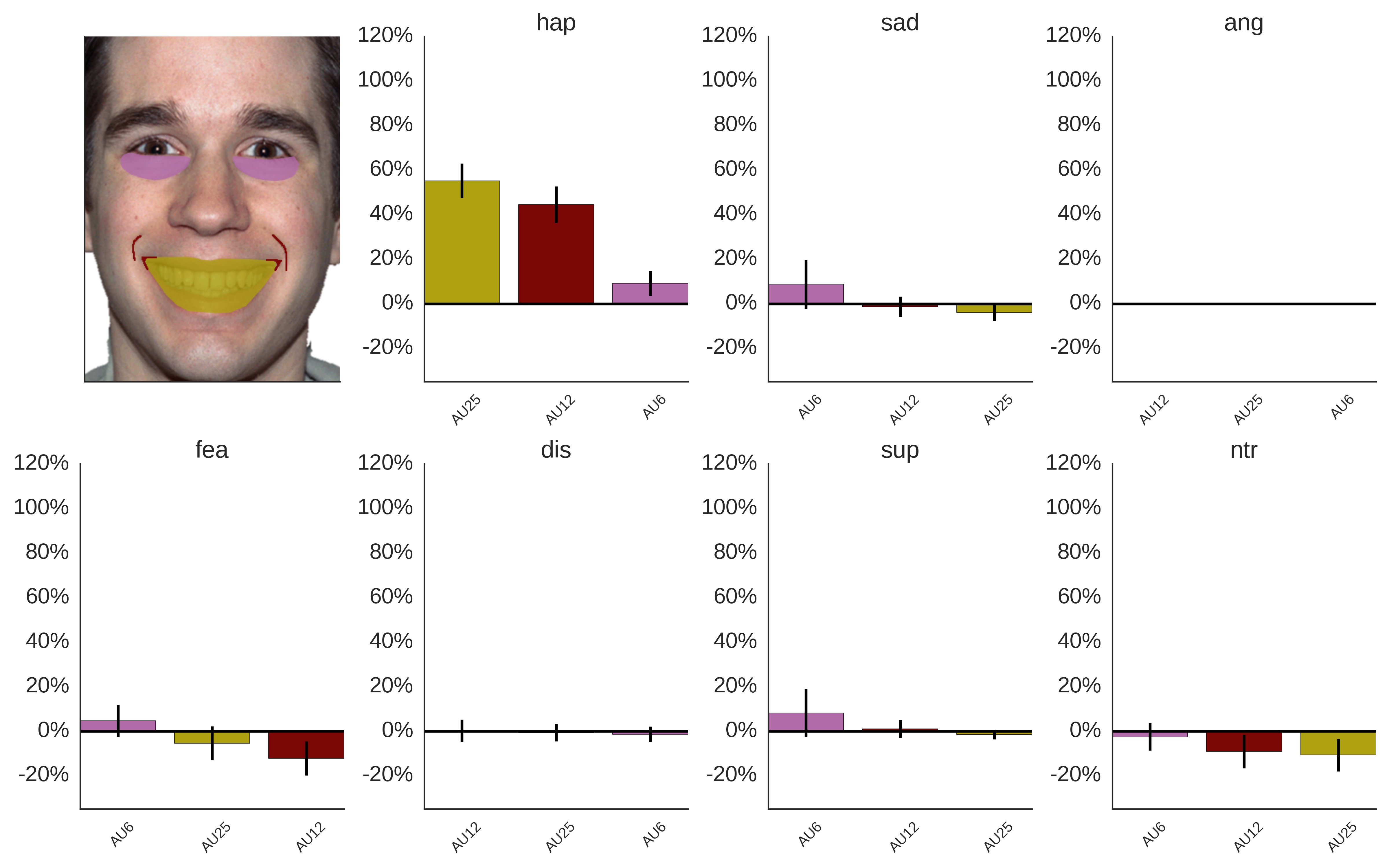

Supplement: S10 Code — (ZIP) [file pone.0177239.s012.zip › mappingTheEmotionalFace-master/auConfusions/confPlot_m_hap.png]

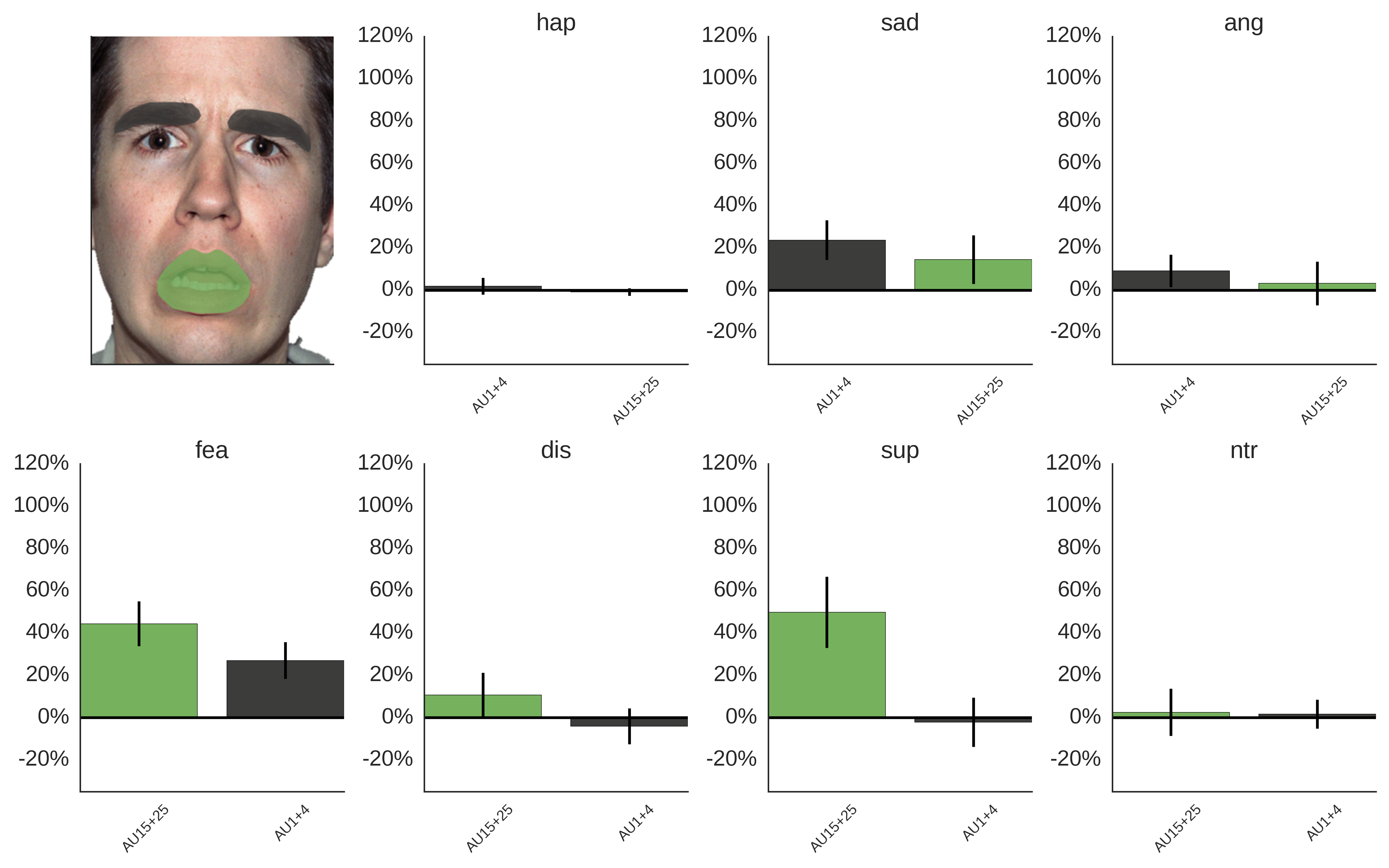

Supplement: S10 Code — (ZIP) [file pone.0177239.s012.zip › mappingTheEmotionalFace-master/auConfusions/confPlot_m_sad.png]

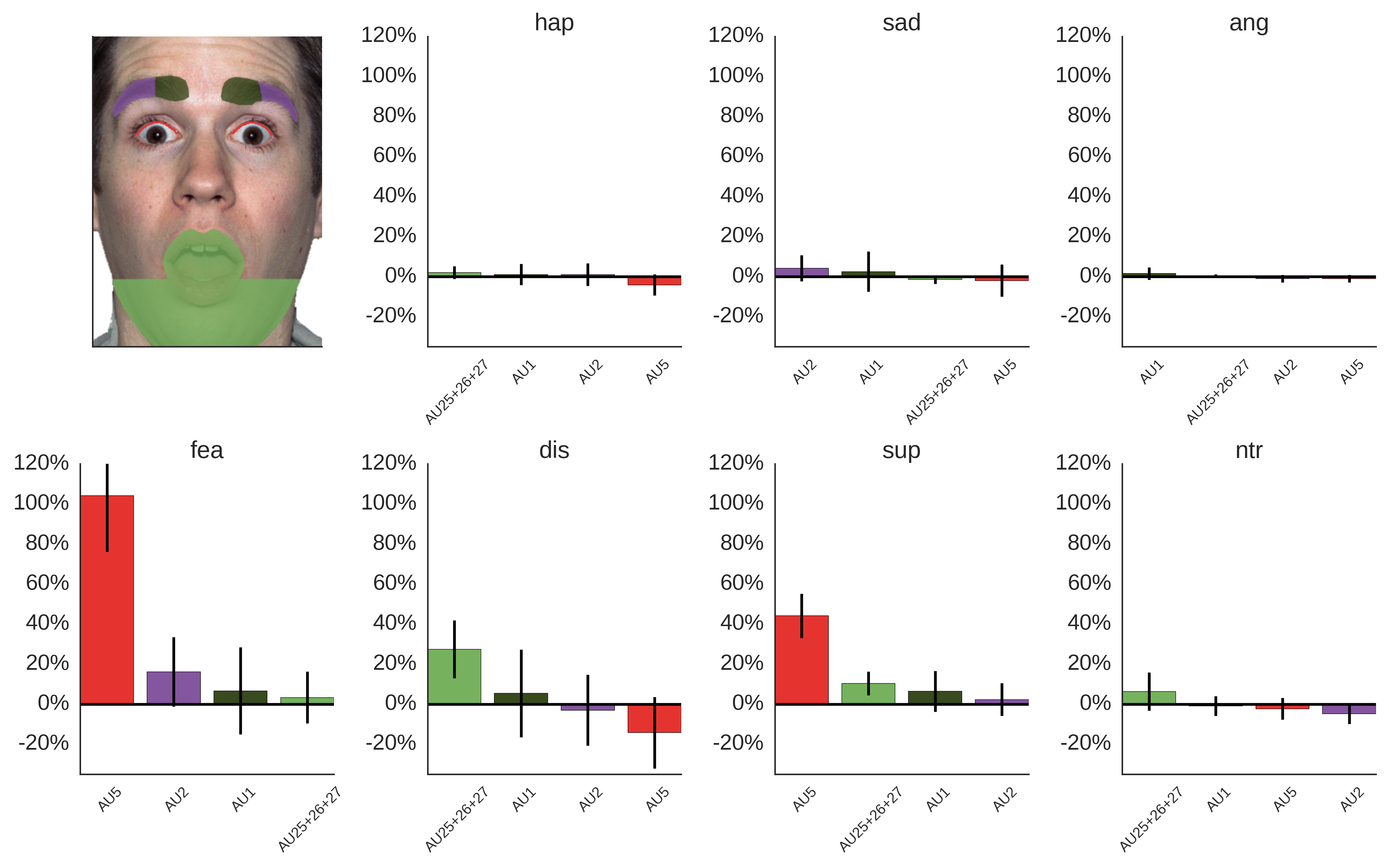

Supplement: S10 Code — (ZIP) [file pone.0177239.s012.zip › mappingTheEmotionalFace-master/auConfusions/confPlot_m_sup.png]

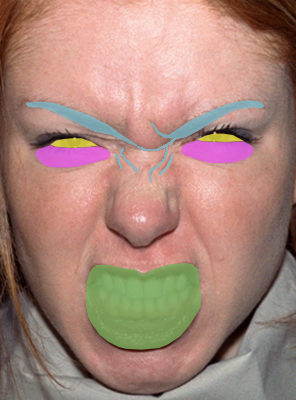

Supplement: S10 Code — (ZIP) [file pone.0177239.s012.zip › mappingTheEmotionalFace-master/auLabels/auVisualisation/f_ang_w_cut.png]

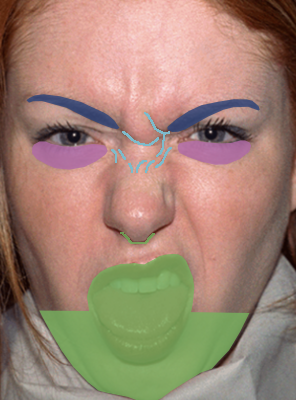

Supplement: S10 Code — (ZIP) [file pone.0177239.s012.zip › mappingTheEmotionalFace-master/auLabels/auVisualisation/f_dis_w_cut.png]

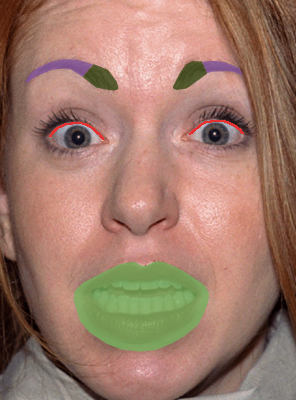

Supplement: S10 Code — (ZIP) [file pone.0177239.s012.zip › mappingTheEmotionalFace-master/auLabels/auVisualisation/f_fea_w_cut.png]

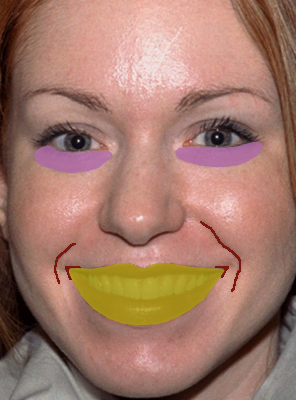

Supplement: S10 Code — (ZIP) [file pone.0177239.s012.zip › mappingTheEmotionalFace-master/auLabels/auVisualisation/f_hap_w_cut.png]

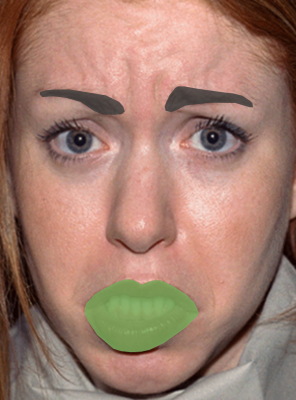

Supplement: S10 Code — (ZIP) [file pone.0177239.s012.zip › mappingTheEmotionalFace-master/auLabels/auVisualisation/f_sad_w_cut.png]

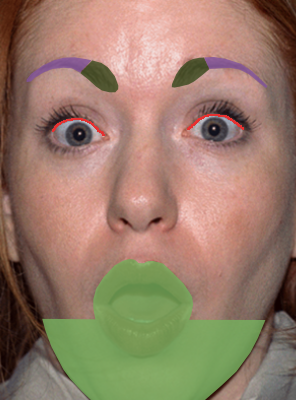

Supplement: S10 Code — (ZIP) [file pone.0177239.s012.zip › mappingTheEmotionalFace-master/auLabels/auVisualisation/f_sup_w_cut.png]

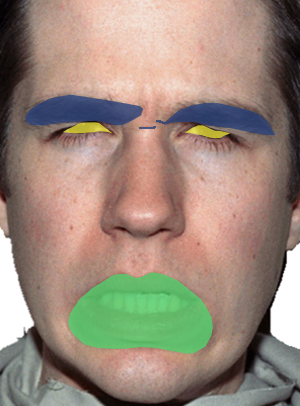

Supplement: S10 Code — (ZIP) [file pone.0177239.s012.zip › mappingTheEmotionalFace-master/auLabels/auVisualisation/m_ang_m_cut.png]

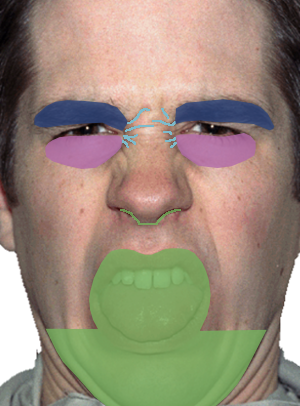

Supplement: S10 Code — (ZIP) [file pone.0177239.s012.zip › mappingTheEmotionalFace-master/auLabels/auVisualisation/m_dis_m_cut.png]

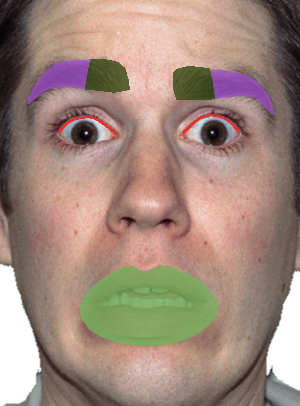

Supplement: S10 Code — (ZIP) [file pone.0177239.s012.zip › mappingTheEmotionalFace-master/auLabels/auVisualisation/m_fea_m_cut.png]

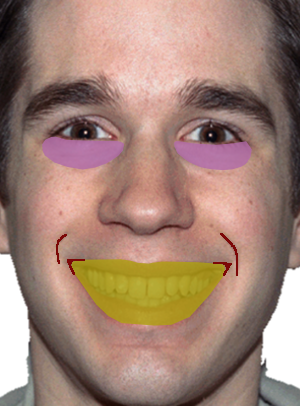

Supplement: S10 Code — (ZIP) [file pone.0177239.s012.zip › mappingTheEmotionalFace-master/auLabels/auVisualisation/m_hap_m_cut.png]

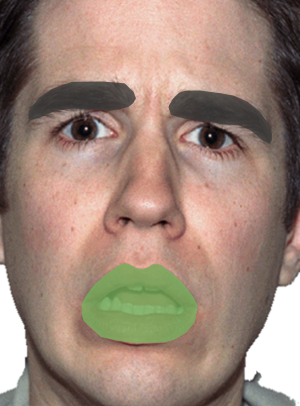

Supplement: S10 Code — (ZIP) [file pone.0177239.s012.zip › mappingTheEmotionalFace-master/auLabels/auVisualisation/m_sad_m_cut.png]

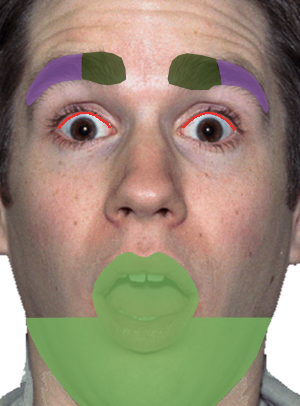

Supplement: S10 Code — (ZIP) [file pone.0177239.s012.zip › mappingTheEmotionalFace-master/auLabels/auVisualisation/m_sup_m_cut.png]

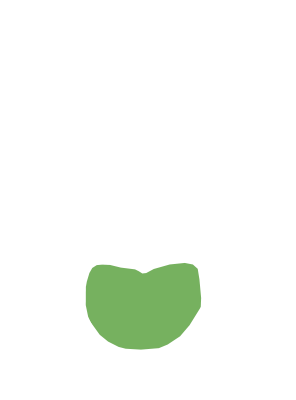

Supplement: S10 Code — (ZIP) [file pone.0177239.s012.zip › mappingTheEmotionalFace-master/auLabels/f_ang_AU22+23+25.png]

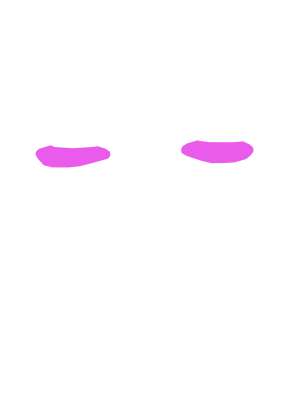

Supplement: S10 Code — (ZIP) [file pone.0177239.s012.zip › mappingTheEmotionalFace-master/auLabels/f_ang_AU6.png]

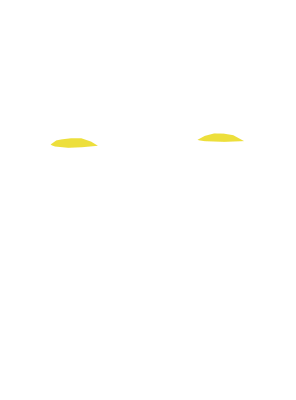

Supplement: S10 Code — (ZIP) [file pone.0177239.s012.zip › mappingTheEmotionalFace-master/auLabels/f_ang_AU7.png]

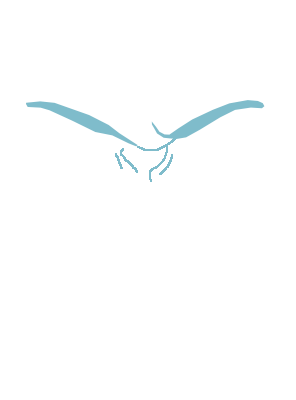

Supplement: S10 Code — (ZIP) [file pone.0177239.s012.zip › mappingTheEmotionalFace-master/auLabels/f_ang_AU9.png]

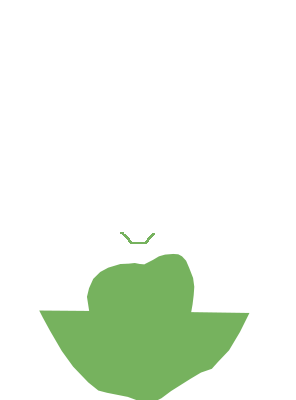

Supplement: S10 Code — (ZIP) [file pone.0177239.s012.zip › mappingTheEmotionalFace-master/auLabels/f_dis_AU10+16+19+25+26.png]

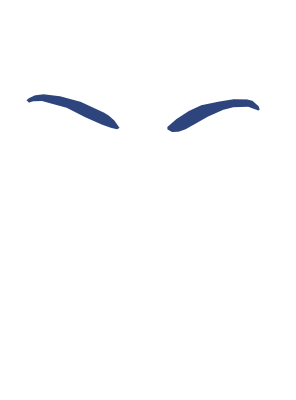

Supplement: S10 Code — (ZIP) [file pone.0177239.s012.zip › mappingTheEmotionalFace-master/auLabels/f_dis_AU4.png]

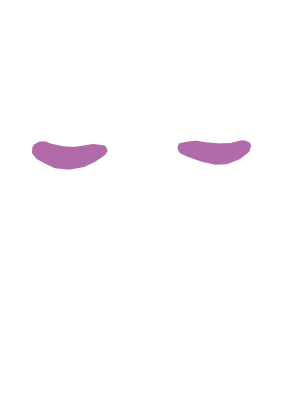

Supplement: S10 Code — (ZIP) [file pone.0177239.s012.zip › mappingTheEmotionalFace-master/auLabels/f_dis_AU6.png]

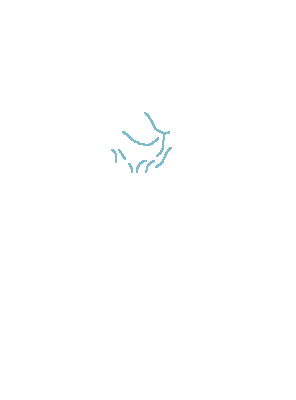

Supplement: S10 Code — (ZIP) [file pone.0177239.s012.zip › mappingTheEmotionalFace-master/auLabels/f_dis_AU9.png]

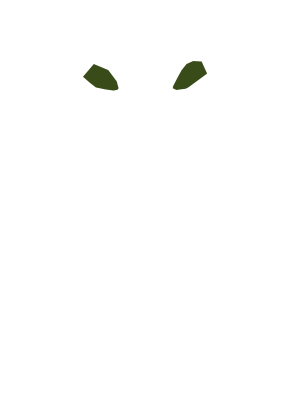

Supplement: S10 Code — (ZIP) [file pone.0177239.s012.zip › mappingTheEmotionalFace-master/auLabels/f_fea_AU1.png]

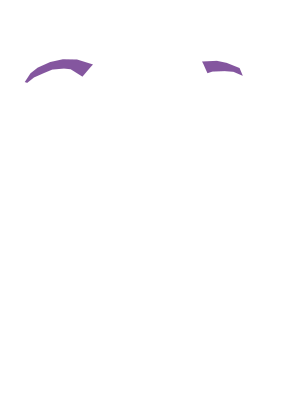

Supplement: S10 Code — (ZIP) [file pone.0177239.s012.zip › mappingTheEmotionalFace-master/auLabels/f_fea_AU2.png]

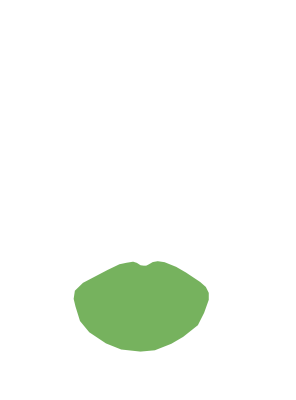

Supplement: S10 Code — (ZIP) [file pone.0177239.s012.zip › mappingTheEmotionalFace-master/auLabels/f_fea_AU20+25.png]

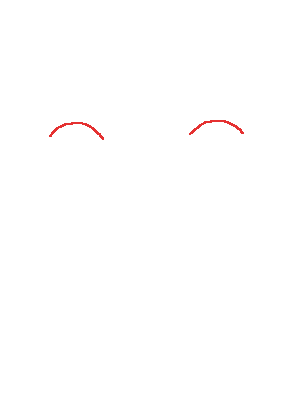

Supplement: S10 Code — (ZIP) [file pone.0177239.s012.zip › mappingTheEmotionalFace-master/auLabels/f_fea_AU5.png]

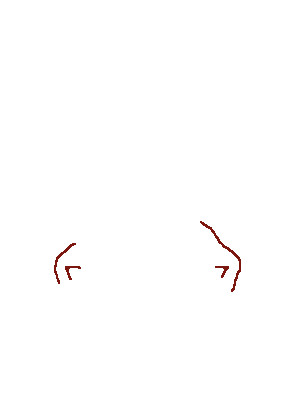

Supplement: S10 Code — (ZIP) [file pone.0177239.s012.zip › mappingTheEmotionalFace-master/auLabels/f_hap_AU12.png]

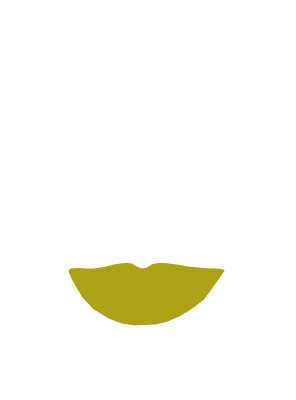

Supplement: S10 Code — (ZIP) [file pone.0177239.s012.zip › mappingTheEmotionalFace-master/auLabels/f_hap_AU25.png]

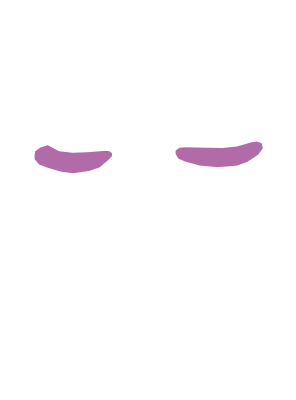

Supplement: S10 Code — (ZIP) [file pone.0177239.s012.zip › mappingTheEmotionalFace-master/auLabels/f_hap_AU6.png]

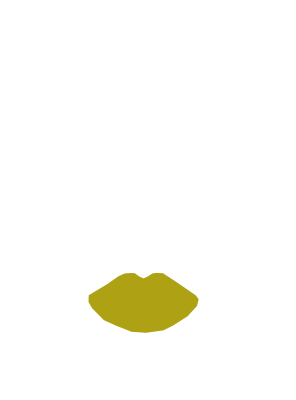

Supplement: S10 Code — (ZIP) [file pone.0177239.s012.zip › mappingTheEmotionalFace-master/auLabels/f_ntr_AU25.png]

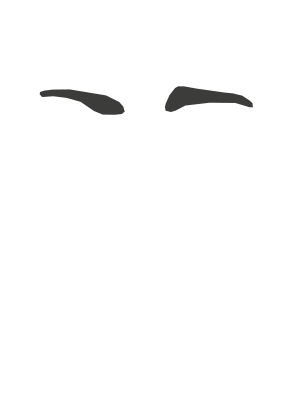

Supplement: S10 Code — (ZIP) [file pone.0177239.s012.zip › mappingTheEmotionalFace-master/auLabels/f_sad_AU1+4.png]

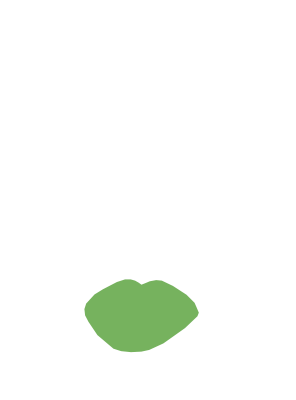

Supplement: S10 Code — (ZIP) [file pone.0177239.s012.zip › mappingTheEmotionalFace-master/auLabels/f_sad_AU15+25.png]

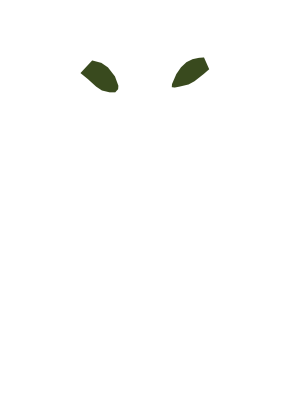

Supplement: S10 Code — (ZIP) [file pone.0177239.s012.zip › mappingTheEmotionalFace-master/auLabels/f_sup_AU1.png]

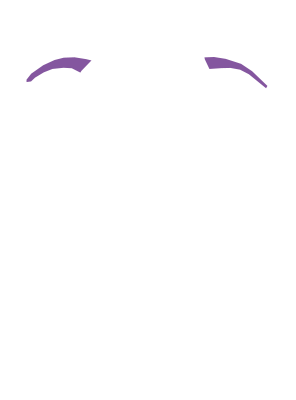

Supplement: S10 Code — (ZIP) [file pone.0177239.s012.zip › mappingTheEmotionalFace-master/auLabels/f_sup_AU2.png]

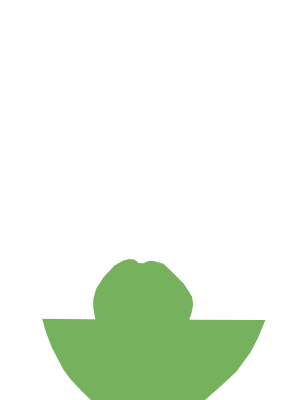

Supplement: S10 Code — (ZIP) [file pone.0177239.s012.zip › mappingTheEmotionalFace-master/auLabels/f_sup_AU25+26+27.png]

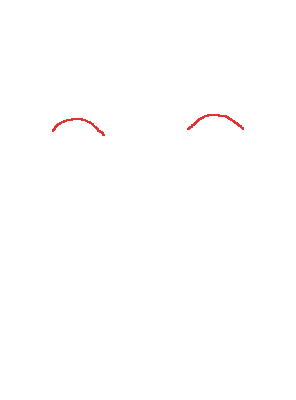

Supplement: S10 Code — (ZIP) [file pone.0177239.s012.zip › mappingTheEmotionalFace-master/auLabels/f_sup_AU5.png]

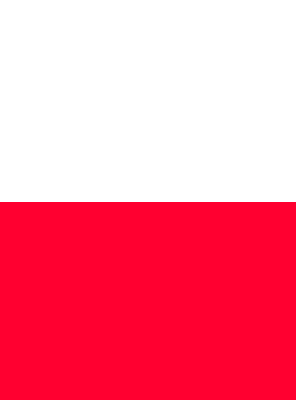

Supplement: S10 Code — (ZIP) [file pone.0177239.s012.zip › mappingTheEmotionalFace-master/auLabels/lower.png]

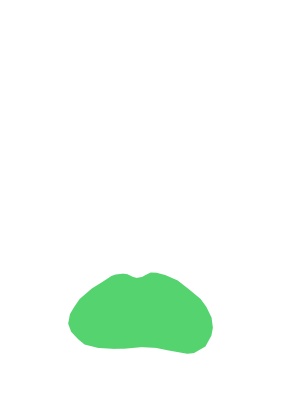

Supplement: S10 Code — (ZIP) [file pone.0177239.s012.zip › mappingTheEmotionalFace-master/auLabels/m_ang_AU25+29.png]

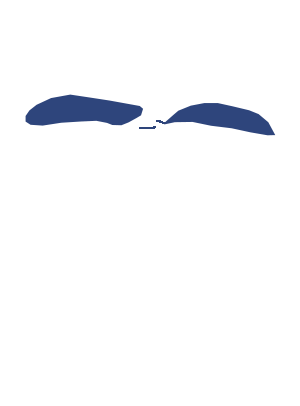

Supplement: S10 Code — (ZIP) [file pone.0177239.s012.zip › mappingTheEmotionalFace-master/auLabels/m_ang_AU4.png]

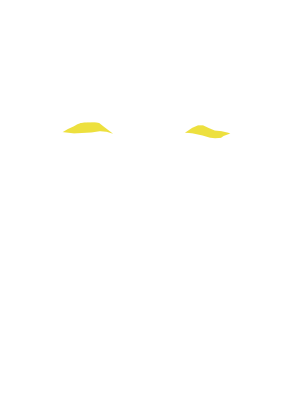

Supplement: S10 Code — (ZIP) [file pone.0177239.s012.zip › mappingTheEmotionalFace-master/auLabels/m_ang_AU7.png]

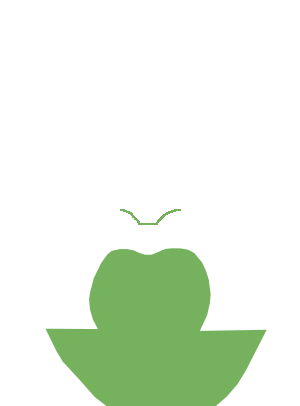

Supplement: S10 Code — (ZIP) [file pone.0177239.s012.zip › mappingTheEmotionalFace-master/auLabels/m_dis_AU10+16+19+25+26.png]

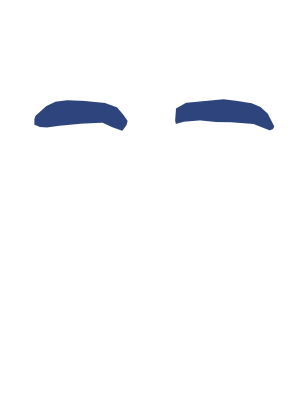

Supplement: S10 Code — (ZIP) [file pone.0177239.s012.zip › mappingTheEmotionalFace-master/auLabels/m_dis_AU4.png]

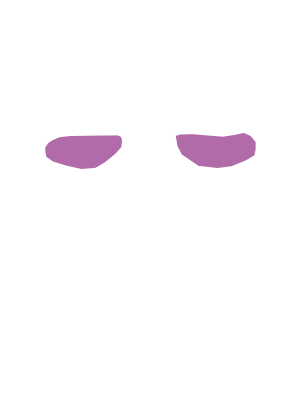

Supplement: S10 Code — (ZIP) [file pone.0177239.s012.zip › mappingTheEmotionalFace-master/auLabels/m_dis_AU6.png]

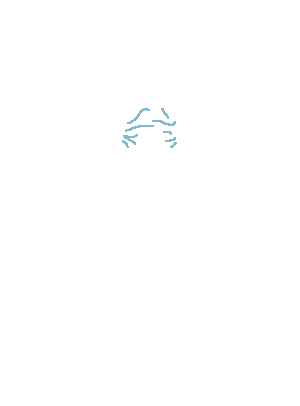

Supplement: S10 Code — (ZIP) [file pone.0177239.s012.zip › mappingTheEmotionalFace-master/auLabels/m_dis_AU9.png]

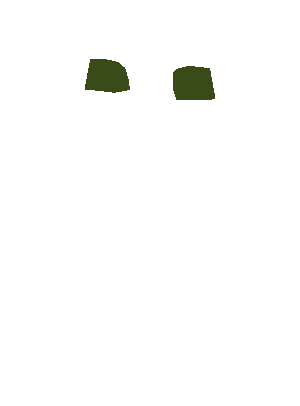

Supplement: S10 Code — (ZIP) [file pone.0177239.s012.zip › mappingTheEmotionalFace-master/auLabels/m_fea_AU1.png]

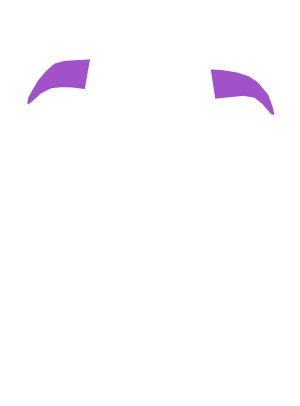

Supplement: S10 Code — (ZIP) [file pone.0177239.s012.zip › mappingTheEmotionalFace-master/auLabels/m_fea_AU2.png]

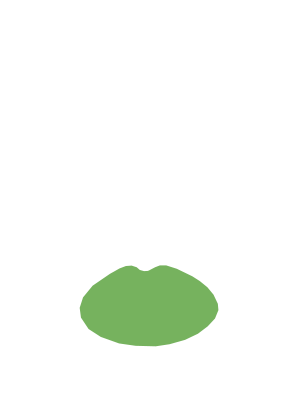

Supplement: S10 Code — (ZIP) [file pone.0177239.s012.zip › mappingTheEmotionalFace-master/auLabels/m_fea_AU20+25.png]

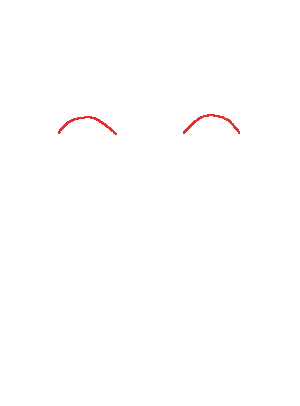

Supplement: S10 Code — (ZIP) [file pone.0177239.s012.zip › mappingTheEmotionalFace-master/auLabels/m_fea_AU5.png]

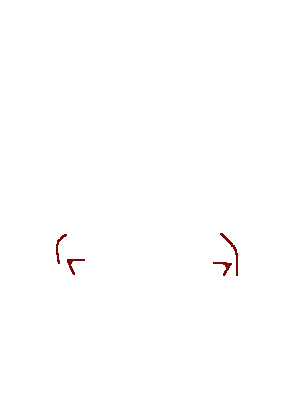

Supplement: S10 Code — (ZIP) [file pone.0177239.s012.zip › mappingTheEmotionalFace-master/auLabels/m_hap_AU12.png]

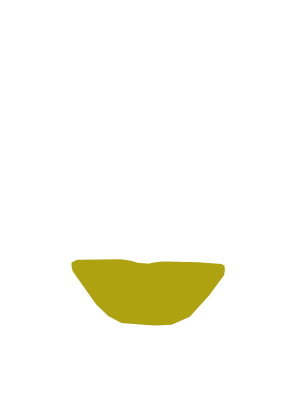

Supplement: S10 Code — (ZIP) [file pone.0177239.s012.zip › mappingTheEmotionalFace-master/auLabels/m_hap_AU25.png]

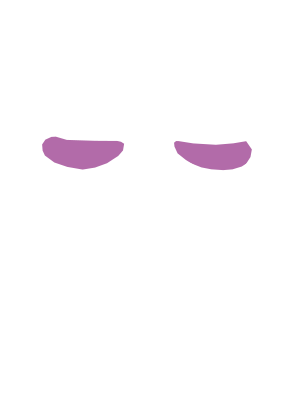

Supplement: S10 Code — (ZIP) [file pone.0177239.s012.zip › mappingTheEmotionalFace-master/auLabels/m_hap_AU6.png]

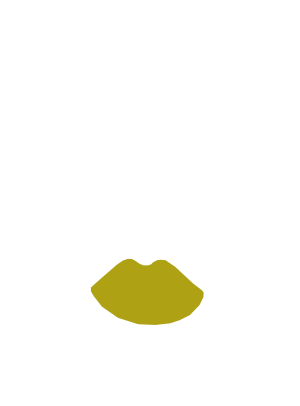

Supplement: S10 Code — (ZIP) [file pone.0177239.s012.zip › mappingTheEmotionalFace-master/auLabels/m_ntr_AU25.png]

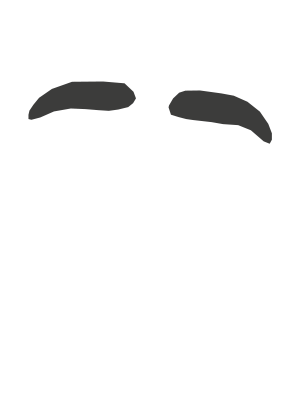

Supplement: S10 Code — (ZIP) [file pone.0177239.s012.zip › mappingTheEmotionalFace-master/auLabels/m_sad_AU1+4.png]

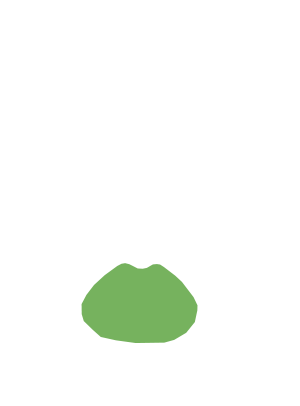

Supplement: S10 Code — (ZIP) [file pone.0177239.s012.zip › mappingTheEmotionalFace-master/auLabels/m_sad_AU15+25.png]

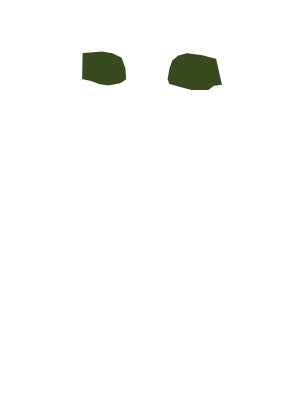

Supplement: S10 Code — (ZIP) [file pone.0177239.s012.zip › mappingTheEmotionalFace-master/auLabels/m_sup_AU1.png]

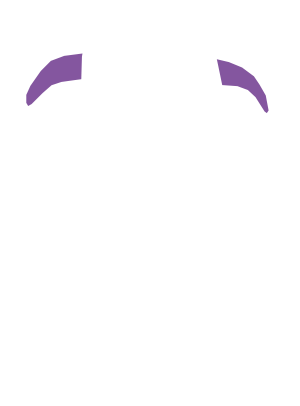

Supplement: S10 Code — (ZIP) [file pone.0177239.s012.zip › mappingTheEmotionalFace-master/auLabels/m_sup_AU2.png]

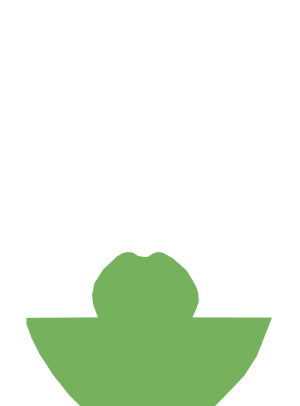

Supplement: S10 Code — (ZIP) [file pone.0177239.s012.zip › mappingTheEmotionalFace-master/auLabels/m_sup_AU25+26+27.png]

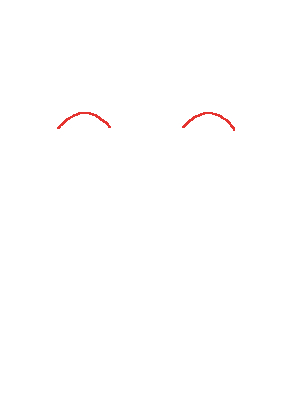

Supplement: S10 Code — (ZIP) [file pone.0177239.s012.zip › mappingTheEmotionalFace-master/auLabels/m_sup_AU5.png]

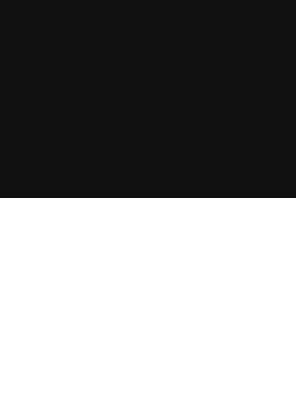

Supplement: S10 Code — (ZIP) [file pone.0177239.s012.zip › mappingTheEmotionalFace-master/auLabels/upper.png]
