# Supplementary material for: Mapping the emotional face. How individual face parts contribute to successful emotion recognition
Source: PLoS One. 2017 May 11;12(5):e0177239. doi: 10.1371/journal.pone.0177239 (PMC5426715; doi:10.1371/journal.pone.0177239)
Supplement: S2 Code — (HTML) [file pone.0177239.s004.html]

code001\_dataImport


# Mapping the emotional face. How individual face parts contribute to successful emotion recognition.

# 1. Reading Logfiles¶

This notebook takes all logfiles from the experiment and transforms them into pandas dataframes. Then these tables are saved as csv files for later re-use in other notebooks where the actual analysis happens.

All aquired data (i.e. the logfiles that are imported in this notebook, located in the folder ./experiment/app/static/logfiles/) are licensed under a creative commons Public Domain Dedication (No Rights Reserved).

## Import modules¶

Modules and global variables needed in most or all notebooks are stored in the file myBasics.py

In [1]:

```
from myBasics import *
```

## Get Logfiles¶

In [2]:

```
def getLogfile(whichFolder, whichExperiment):
    logList = []
    for filename in os.listdir(whichFolder):
        if fnmatch.fnmatch(filename, whichExperiment):
            logList.append(whichFolder+filename)
    return logList
```

In [3]:

```
myFolder = '../experiment/app/static/logfiles/'
myExperiment = 'log*.txt'
logList = getLogfile(myFolder,myExperiment)
logList.sort()
```

These are all the logfiles we have. Note that #10 and #67 are missing, because they were not started (e.g. participant did not come to the appointment). But these are not dropouts and there are no data that were unusable or had to be removed from the analyses.

In [4]:

```
i = 0
for log in logList:
    print i,':\t',log
    i+=1
```

```
0 :	../experiment/app/static/logfiles/logfile1.txt
1 :	../experiment/app/static/logfiles/logfile11.txt
2 :	../experiment/app/static/logfiles/logfile12.txt
3 :	../experiment/app/static/logfiles/logfile13.txt
4 :	../experiment/app/static/logfiles/logfile14.txt
5 :	../experiment/app/static/logfiles/logfile15.txt
6 :	../experiment/app/static/logfiles/logfile16.txt
7 :	../experiment/app/static/logfiles/logfile17.txt
8 :	../experiment/app/static/logfiles/logfile18.txt
9 :	../experiment/app/static/logfiles/logfile19.txt
10 :	../experiment/app/static/logfiles/logfile2.txt
11 :	../experiment/app/static/logfiles/logfile20.txt
12 :	../experiment/app/static/logfiles/logfile21.txt
13 :	../experiment/app/static/logfiles/logfile22.txt
14 :	../experiment/app/static/logfiles/logfile23.txt
15 :	../experiment/app/static/logfiles/logfile24.txt
16 :	../experiment/app/static/logfiles/logfile25.txt
17 :	../experiment/app/static/logfiles/logfile26.txt
18 :	../experiment/app/static/logfiles/logfile27.txt
19 :	../experiment/app/static/logfiles/logfile28.txt
20 :	../experiment/app/static/logfiles/logfile29.txt
21 :	../experiment/app/static/logfiles/logfile3.txt
22 :	../experiment/app/static/logfiles/logfile30.txt
23 :	../experiment/app/static/logfiles/logfile31.txt
24 :	../experiment/app/static/logfiles/logfile32.txt
25 :	../experiment/app/static/logfiles/logfile33.txt
26 :	../experiment/app/static/logfiles/logfile34.txt
27 :	../experiment/app/static/logfiles/logfile35.txt
28 :	../experiment/app/static/logfiles/logfile36.txt
29 :	../experiment/app/static/logfiles/logfile37.txt
30 :	../experiment/app/static/logfiles/logfile38.txt
31 :	../experiment/app/static/logfiles/logfile39.txt
32 :	../experiment/app/static/logfiles/logfile4.txt
33 :	../experiment/app/static/logfiles/logfile40.txt
34 :	../experiment/app/static/logfiles/logfile41.txt
35 :	../experiment/app/static/logfiles/logfile42.txt
36 :	../experiment/app/static/logfiles/logfile43.txt
37 :	../experiment/app/static/logfiles/logfile44.txt
38 :	../experiment/app/static/logfiles/logfile45.txt
39 :	../experiment/app/static/logfiles/logfile46.txt
40 :	../experiment/app/static/logfiles/logfile47.txt
41 :	../experiment/app/static/logfiles/logfile48.txt
42 :	../experiment/app/static/logfiles/logfile49.txt
43 :	../experiment/app/static/logfiles/logfile5.txt
44 :	../experiment/app/static/logfiles/logfile50.txt
45 :	../experiment/app/static/logfiles/logfile51.txt
46 :	../experiment/app/static/logfiles/logfile52.txt
47 :	../experiment/app/static/logfiles/logfile53.txt
48 :	../experiment/app/static/logfiles/logfile54.txt
49 :	../experiment/app/static/logfiles/logfile55.txt
50 :	../experiment/app/static/logfiles/logfile56.txt
51 :	../experiment/app/static/logfiles/logfile57.txt
52 :	../experiment/app/static/logfiles/logfile58.txt
53 :	../experiment/app/static/logfiles/logfile59.txt
54 :	../experiment/app/static/logfiles/logfile6.txt
55 :	../experiment/app/static/logfiles/logfile60.txt
56 :	../experiment/app/static/logfiles/logfile61.txt
57 :	../experiment/app/static/logfiles/logfile62.txt
58 :	../experiment/app/static/logfiles/logfile63.txt
59 :	../experiment/app/static/logfiles/logfile64.txt
60 :	../experiment/app/static/logfiles/logfile65.txt
61 :	../experiment/app/static/logfiles/logfile66.txt
62 :	../experiment/app/static/logfiles/logfile68.txt
63 :	../experiment/app/static/logfiles/logfile69.txt
64 :	../experiment/app/static/logfiles/logfile7.txt
65 :	../experiment/app/static/logfiles/logfile70.txt
66 :	../experiment/app/static/logfiles/logfile71.txt
67 :	../experiment/app/static/logfiles/logfile72.txt
68 :	../experiment/app/static/logfiles/logfile73.txt
69 :	../experiment/app/static/logfiles/logfile74.txt
70 :	../experiment/app/static/logfiles/logfile75.txt
71 :	../experiment/app/static/logfiles/logfile76.txt
72 :	../experiment/app/static/logfiles/logfile77.txt
73 :	../experiment/app/static/logfiles/logfile78.txt
74 :	../experiment/app/static/logfiles/logfile79.txt
75 :	../experiment/app/static/logfiles/logfile8.txt
76 :	../experiment/app/static/logfiles/logfile80.txt
77 :	../experiment/app/static/logfiles/logfile81.txt
78 :	../experiment/app/static/logfiles/logfile82.txt
79 :	../experiment/app/static/logfiles/logfile83.txt
80 :	../experiment/app/static/logfiles/logfile84.txt
81 :	../experiment/app/static/logfiles/logfile85.txt
82 :	../experiment/app/static/logfiles/logfile86.txt
83 :	../experiment/app/static/logfiles/logfile87.txt
84 :	../experiment/app/static/logfiles/logfile88.txt
85 :	../experiment/app/static/logfiles/logfile89.txt
86 :	../experiment/app/static/logfiles/logfile9.txt
87 :	../experiment/app/static/logfiles/logfile90.txt
88 :	../experiment/app/static/logfiles/logfile91.txt
89 :	../experiment/app/static/logfiles/logfile92.txt
90 :	../experiment/app/static/logfiles/logfile93.txt
91 :	../experiment/app/static/logfiles/logfile94.txt
92 :	../experiment/app/static/logfiles/logfile95.txt
93 :	../experiment/app/static/logfiles/logfile96.txt
```

Example of how the head of the logfile looks:

In [5]:

```
for index,entry in enumerate(open(logList[-1],'r')):
    print entry
    if index > 6:
        break
```

```
####### THIS IS A LOGFILE FOR THE DYNAMIC MASKING FACE EXPERIMENT ######

Participant Number: 96

Date and Time: 2016-03-10 15:04:08.090637

age: 20 ,gender: 1 ,environ: 2 ,occup: 0 ,advert: 0

###################################################################


time	cumtime	express	ident	button	filename	evaluation	stopRT	choiceRT	maskNum	maskList

2016-03-10 15:04:53	0.0	2	1	ang	img/m_ang_cut.png	HIT	25384.0	34289.0	26	33-43-3-21-44-34-8-10-36-20-15-1-29-19-38-9-25-40-47-16-32-4-13-23-12-0
```

### Get information about age and gender¶

In [6]:

```
def getDemographics(logList):
    #empty dict to write to
    d = {}
    # loop through the logfiles of all participants
    for log in logList:
        # get the filename of the logfile (clean)
        logName = log[log.rfind('/logfile')+len('/logfile'):log.rfind('.')]
        # loop throught the logfiles content
        for index,entry in enumerate(open(log,'r')):
            # the demographics are stored in the 3rd row
            if index ==3:
                # we get the conents of that row, split it and put in in a list
                thisEntry = entry.split()
                
                d['p'+('000'+logName)[-3:]] = {'age':int(thisEntry[1]) , 'gender':int(thisEntry[3])}
    
    # make a dataframe
    demographicsDf = pd.DataFrame(d).T
    # missing values (99) are turned into nans
    demographicsDf = demographicsDf.replace(99,np.nan)
    return demographicsDf
```

In [7]:

```
demoDf = getDemographics(logList)
```

In [8]:

```
demoDf.head()
```

Out[8]:

|  | age | gender |
| --- | --- | --- |
| p001 | 24 | 0 |
| p002 | 30 | 0 |
| p003 | 25 | 0 |
| p004 | 26 | 0 |
| p005 | 28 | 1 |

In [9]:

```
demoDf.describe()
```

Out[9]:

|  | age | gender |
| --- | --- | --- |
| count | 94.000000 | 94.000000 |
| mean | 23.595745 | 0.361702 |
| std | 3.370712 | 0.483070 |
| min | 18.000000 | 0.000000 |
| 25% | 21.000000 | 0.000000 |
| 50% | 24.000000 | 0.000000 |
| 75% | 25.000000 | 1.000000 |
| max | 36.000000 | 1.000000 |

In [10]:

```
# 0 == female
# 1 == male
# 2 == other
# 99/NaN == no information
demoDf['gender'].value_counts()
```

Out[10]:

```
0    60
1    34
Name: gender, dtype: int64
```

## Some meta-information and sanity checks¶

### Get the times when the first and last trials were shown¶

In [11]:

```
def getClocktimes(logFile):
    timing = []
    for entry in open(logFile,'r'):
        if 'img' in entry:
            timing.append(entry.split()[1])
    print logFile, '; started at %s ; finished at: %s' %(timing[0], timing[-1])
```

In [12]:

```
for logFile in logList:
    getClocktimes(logFile)
```

```
../experiment/app/static/logfiles/logfile1.txt ; started at 15:01:20 ; finished at: 16:02:43
../experiment/app/static/logfiles/logfile11.txt ; started at 11:52:16 ; finished at: 12:35:53
../experiment/app/static/logfiles/logfile12.txt ; started at 11:52:55 ; finished at: 13:05:16
../experiment/app/static/logfiles/logfile13.txt ; started at 14:08:20 ; finished at: 14:56:36
../experiment/app/static/logfiles/logfile14.txt ; started at 15:31:56 ; finished at: 16:26:33
../experiment/app/static/logfiles/logfile15.txt ; started at 15:31:41 ; finished at: 16:38:39
../experiment/app/static/logfiles/logfile16.txt ; started at 11:57:53 ; finished at: 12:44:36
../experiment/app/static/logfiles/logfile17.txt ; started at 12:00:49 ; finished at: 12:56:46
../experiment/app/static/logfiles/logfile18.txt ; started at 14:14:05 ; finished at: 15:25:08
../experiment/app/static/logfiles/logfile19.txt ; started at 14:32:17 ; finished at: 15:22:41
../experiment/app/static/logfiles/logfile2.txt ; started at 17:06:50 ; finished at: 17:44:52
../experiment/app/static/logfiles/logfile20.txt ; started at 16:13:00 ; finished at: 16:58:07
../experiment/app/static/logfiles/logfile21.txt ; started at 16:13:32 ; finished at: 16:56:18
../experiment/app/static/logfiles/logfile22.txt ; started at 09:46:44 ; finished at: 10:47:41
../experiment/app/static/logfiles/logfile23.txt ; started at 09:47:30 ; finished at: 10:57:35
../experiment/app/static/logfiles/logfile24.txt ; started at 10:56:47 ; finished at: 11:59:55
../experiment/app/static/logfiles/logfile25.txt ; started at 10:57:48 ; finished at: 12:02:43
../experiment/app/static/logfiles/logfile26.txt ; started at 11:01:32 ; finished at: 11:48:03
../experiment/app/static/logfiles/logfile27.txt ; started at 15:25:41 ; finished at: 16:04:10
../experiment/app/static/logfiles/logfile28.txt ; started at 15:25:34 ; finished at: 16:18:21
../experiment/app/static/logfiles/logfile29.txt ; started at 10:11:30 ; finished at: 11:02:26
../experiment/app/static/logfiles/logfile3.txt ; started at 14:59:48 ; finished at: 15:48:42
../experiment/app/static/logfiles/logfile30.txt ; started at 12:02:14 ; finished at: 12:35:14
../experiment/app/static/logfiles/logfile31.txt ; started at 15:28:40 ; finished at: 16:06:19
../experiment/app/static/logfiles/logfile32.txt ; started at 08:27:30 ; finished at: 09:13:17
../experiment/app/static/logfiles/logfile33.txt ; started at 10:17:16 ; finished at: 11:03:39
../experiment/app/static/logfiles/logfile34.txt ; started at 10:05:19 ; finished at: 10:58:23
../experiment/app/static/logfiles/logfile35.txt ; started at 12:59:58 ; finished at: 14:12:35
../experiment/app/static/logfiles/logfile36.txt ; started at 13:01:14 ; finished at: 13:50:17
../experiment/app/static/logfiles/logfile37.txt ; started at 13:11:14 ; finished at: 13:53:53
../experiment/app/static/logfiles/logfile38.txt ; started at 08:06:03 ; finished at: 09:03:04
../experiment/app/static/logfiles/logfile39.txt ; started at 11:53:23 ; finished at: 12:48:32
../experiment/app/static/logfiles/logfile4.txt ; started at 16:26:17 ; finished at: 17:21:07
../experiment/app/static/logfiles/logfile40.txt ; started at 12:57:26 ; finished at: 13:48:29
../experiment/app/static/logfiles/logfile41.txt ; started at 12:57:44 ; finished at: 13:49:12
../experiment/app/static/logfiles/logfile42.txt ; started at 16:02:41 ; finished at: 16:54:38
../experiment/app/static/logfiles/logfile43.txt ; started at 12:59:15 ; finished at: 14:06:48
../experiment/app/static/logfiles/logfile44.txt ; started at 13:08:10 ; finished at: 13:57:16
../experiment/app/static/logfiles/logfile45.txt ; started at 16:04:44 ; finished at: 16:58:07
../experiment/app/static/logfiles/logfile46.txt ; started at 10:57:53 ; finished at: 11:51:38
../experiment/app/static/logfiles/logfile47.txt ; started at 12:57:12 ; finished at: 13:36:45
../experiment/app/static/logfiles/logfile48.txt ; started at 12:57:12 ; finished at: 13:38:29
../experiment/app/static/logfiles/logfile49.txt ; started at 13:02:01 ; finished at: 13:52:45
../experiment/app/static/logfiles/logfile5.txt ; started at 15:53:34 ; finished at: 17:01:05
../experiment/app/static/logfiles/logfile50.txt ; started at 17:30:51 ; finished at: 18:21:46
../experiment/app/static/logfiles/logfile51.txt ; started at 17:32:31 ; finished at: 18:29:58
../experiment/app/static/logfiles/logfile52.txt ; started at 11:54:58 ; finished at: 12:32:25
../experiment/app/static/logfiles/logfile53.txt ; started at 11:55:13 ; finished at: 13:06:03
../experiment/app/static/logfiles/logfile54.txt ; started at 16:04:43 ; finished at: 16:49:29
../experiment/app/static/logfiles/logfile55.txt ; started at 17:43:09 ; finished at: 18:32:49
../experiment/app/static/logfiles/logfile56.txt ; started at 12:03:49 ; finished at: 12:49:50
../experiment/app/static/logfiles/logfile57.txt ; started at 12:03:47 ; finished at: 12:47:25
../experiment/app/static/logfiles/logfile58.txt ; started at 12:00:17 ; finished at: 12:36:42
../experiment/app/static/logfiles/logfile59.txt ; started at 12:00:29 ; finished at: 12:42:33
../experiment/app/static/logfiles/logfile6.txt ; started at 10:10:08 ; finished at: 11:06:53
../experiment/app/static/logfiles/logfile60.txt ; started at 12:03:49 ; finished at: 12:41:30
../experiment/app/static/logfiles/logfile61.txt ; started at 12:53:21 ; finished at: 13:40:56
../experiment/app/static/logfiles/logfile62.txt ; started at 13:01:51 ; finished at: 13:58:32
../experiment/app/static/logfiles/logfile63.txt ; started at 11:58:08 ; finished at: 12:38:07
../experiment/app/static/logfiles/logfile64.txt ; started at 10:33:38 ; finished at: 11:25:00
../experiment/app/static/logfiles/logfile65.txt ; started at 13:27:19 ; finished at: 14:10:20
../experiment/app/static/logfiles/logfile66.txt ; started at 11:59:49 ; finished at: 12:53:26
../experiment/app/static/logfiles/logfile68.txt ; started at 16:05:33 ; finished at: 16:49:42
../experiment/app/static/logfiles/logfile69.txt ; started at 10:03:35 ; finished at: 10:45:23
../experiment/app/static/logfiles/logfile7.txt ; started at 10:10:52 ; finished at: 11:12:10
../experiment/app/static/logfiles/logfile70.txt ; started at 11:02:43 ; finished at: 11:59:17
../experiment/app/static/logfiles/logfile71.txt ; started at 14:01:51 ; finished at: 14:50:36
../experiment/app/static/logfiles/logfile72.txt ; started at 10:03:20 ; finished at: 10:47:48
../experiment/app/static/logfiles/logfile73.txt ; started at 12:08:23 ; finished at: 12:52:02
../experiment/app/static/logfiles/logfile74.txt ; started at 10:00:46 ; finished at: 10:57:17
../experiment/app/static/logfiles/logfile75.txt ; started at 10:59:32 ; finished at: 11:45:42
../experiment/app/static/logfiles/logfile76.txt ; started at 11:08:09 ; finished at: 12:03:12
../experiment/app/static/logfiles/logfile77.txt ; started at 10:10:57 ; finished at: 11:18:42
../experiment/app/static/logfiles/logfile78.txt ; started at 15:31:44 ; finished at: 16:12:14
../experiment/app/static/logfiles/logfile79.txt ; started at 15:33:53 ; finished at: 16:36:56
../experiment/app/static/logfiles/logfile8.txt ; started at 10:11:07 ; finished at: 10:59:00
../experiment/app/static/logfiles/logfile80.txt ; started at 15:45:27 ; finished at: 16:32:56
../experiment/app/static/logfiles/logfile81.txt ; started at 11:00:51 ; finished at: 11:56:47
../experiment/app/static/logfiles/logfile82.txt ; started at 10:56:01 ; finished at: 11:33:05
../experiment/app/static/logfiles/logfile83.txt ; started at 10:57:34 ; finished at: 11:37:47
../experiment/app/static/logfiles/logfile84.txt ; started at 11:11:45 ; finished at: 11:46:29
../experiment/app/static/logfiles/logfile85.txt ; started at 11:03:59 ; finished at: 12:00:17
../experiment/app/static/logfiles/logfile86.txt ; started at 13:50:46 ; finished at: 14:38:59
../experiment/app/static/logfiles/logfile87.txt ; started at 13:51:04 ; finished at: 14:41:06
../experiment/app/static/logfiles/logfile88.txt ; started at 13:59:35 ; finished at: 14:41:03
../experiment/app/static/logfiles/logfile89.txt ; started at 10:06:02 ; finished at: 11:08:51
../experiment/app/static/logfiles/logfile9.txt ; started at 11:07:58 ; finished at: 11:56:50
../experiment/app/static/logfiles/logfile90.txt ; started at 13:04:22 ; finished at: 13:48:56
../experiment/app/static/logfiles/logfile91.txt ; started at 09:55:27 ; finished at: 10:55:31
../experiment/app/static/logfiles/logfile92.txt ; started at 11:00:37 ; finished at: 11:49:11
../experiment/app/static/logfiles/logfile93.txt ; started at 11:01:08 ; finished at: 11:49:51
../experiment/app/static/logfiles/logfile94.txt ; started at 13:57:31 ; finished at: 14:52:09
../experiment/app/static/logfiles/logfile95.txt ; started at 14:10:28 ; finished at: 15:17:39
../experiment/app/static/logfiles/logfile96.txt ; started at 15:04:53 ; finished at: 15:52:53
```

### Get the length of each block and the cumulated time of blocks¶

In [13]:

```
def getBlocktimes(logfile):
    cumtime = []
    blocktime = []
    for entry in open(logfile,'r'):
        if 'img' in entry:
            cumtime.append( float(entry.split()[2]) )
            try:
                if cumtime[-2] > cumtime[-1]:
                    blocktime.append(round(cumtime[-2]/60,2))
            except:
                pass
    blocktime.append(round(cumtime[-1]/60,2))
    print logfile[logfile.rfind('/')+1:], '; block lengths: %s ; total length: %smin' %(blocktime, sum(blocktime))
    return sum(blocktime)
```

In [14]:

```
allTimes = []
for logFile in logList:
    allTimes.append( getBlocktimes(logFile) )
```

```
logfile1.txt ; block lengths: [35.99, 24.64] ; total length: 60.63min
logfile11.txt ; block lengths: [20.44, 22.21] ; total length: 42.65min
logfile12.txt ; block lengths: [38.39, 33.4] ; total length: 71.79min
logfile13.txt ; block lengths: [23.76, 23.57] ; total length: 47.33min
logfile14.txt ; block lengths: [31.64, 21.84] ; total length: 53.48min
logfile15.txt ; block lengths: [36.64, 28.59] ; total length: 65.23min
logfile16.txt ; block lengths: [25.37, 20.7] ; total length: 46.07min
logfile17.txt ; block lengths: [30.37, 24.59] ; total length: 54.96min
logfile18.txt ; block lengths: [36.83, 33.6] ; total length: 70.43min
logfile19.txt ; block lengths: [26.08, 23.18] ; total length: 49.26min
logfile2.txt ; block lengths: [20.65, 16.21] ; total length: 36.86min
logfile20.txt ; block lengths: [25.32, 19.44] ; total length: 44.76min
logfile21.txt ; block lengths: [24.72, 17.07] ; total length: 41.79min
logfile22.txt ; block lengths: [43.75, 15.84] ; total length: 59.59min
logfile23.txt ; block lengths: [36.26, 31.1] ; total length: 67.36min
logfile24.txt ; block lengths: [35.06, 26.7] ; total length: 61.76min
logfile25.txt ; block lengths: [34.7, 28.88] ; total length: 63.58min
logfile26.txt ; block lengths: [25.91, 20.03] ; total length: 45.94min
logfile27.txt ; block lengths: [19.39, 17.67] ; total length: 37.06min
logfile28.txt ; block lengths: [29.73, 22.17] ; total length: 51.9min
logfile29.txt ; block lengths: [25.35, 24.67] ; total length: 50.02min
logfile3.txt ; block lengths: [23.66, 21.72] ; total length: 45.38min
logfile30.txt ; block lengths: [18.88, 13.68] ; total length: 32.56min
logfile31.txt ; block lengths: [20.62, 16.44] ; total length: 37.06min
logfile32.txt ; block lengths: [26.04, 19.24] ; total length: 45.28min
logfile33.txt ; block lengths: [25.63, 19.65] ; total length: 45.28min
logfile34.txt ; block lengths: [27.27, 24.98] ; total length: 52.25min
logfile35.txt ; block lengths: [36.24, 35.45] ; total length: 71.69min
logfile36.txt ; block lengths: [24.98, 23.47] ; total length: 48.45min
logfile37.txt ; block lengths: [23.99, 17.94] ; total length: 41.93min
logfile38.txt ; block lengths: [31.46, 24.56] ; total length: 56.02min
logfile39.txt ; block lengths: [28.36, 26.32] ; total length: 54.68min
logfile4.txt ; block lengths: [30.99, 22.16] ; total length: 53.15min
logfile40.txt ; block lengths: [28.93, 21.38] ; total length: 50.31min
logfile41.txt ; block lengths: [24.11, 26.46] ; total length: 50.57min
logfile42.txt ; block lengths: [31.44, 19.6] ; total length: 51.04min
logfile43.txt ; block lengths: [34.79, 30.31] ; total length: 65.1min
logfile44.txt ; block lengths: [28.14, 18.82] ; total length: 46.96min
logfile45.txt ; block lengths: [26.02, 26.49] ; total length: 52.51min
logfile46.txt ; block lengths: [32.81, 20.29] ; total length: 53.1min
logfile47.txt ; block lengths: [21.63, 16.61] ; total length: 38.24min
logfile48.txt ; block lengths: [18.97, 21.55] ; total length: 40.52min
logfile49.txt ; block lengths: [27.79, 22.39] ; total length: 50.18min
logfile5.txt ; block lengths: [38.57, 25.73] ; total length: 64.3min
logfile50.txt ; block lengths: [23.36, 25.76] ; total length: 49.12min
logfile51.txt ; block lengths: [29.07, 28.0] ; total length: 57.07min
logfile52.txt ; block lengths: [23.91, 12.88] ; total length: 36.79min
logfile53.txt ; block lengths: [40.12, 30.07] ; total length: 70.19min
logfile54.txt ; block lengths: [24.39, 19.34] ; total length: 43.73min
logfile55.txt ; block lengths: [27.05, 22.01] ; total length: 49.06min
logfile56.txt ; block lengths: [26.77, 18.54] ; total length: 45.31min
logfile57.txt ; block lengths: [24.97, 17.76] ; total length: 42.73min
logfile58.txt ; block lengths: [20.53, 14.56] ; total length: 35.09min
logfile59.txt ; block lengths: [19.32, 22.05] ; total length: 41.37min
logfile6.txt ; block lengths: [24.63, 30.6] ; total length: 55.23min
logfile60.txt ; block lengths: [16.24, 20.87] ; total length: 37.11min
logfile61.txt ; block lengths: [24.94, 21.8] ; total length: 46.74min
logfile62.txt ; block lengths: [29.89, 25.95] ; total length: 55.84min
logfile63.txt ; block lengths: [21.04, 18.01] ; total length: 39.05min
logfile64.txt ; block lengths: [27.4, 23.46] ; total length: 50.86min
logfile65.txt ; block lengths: [24.71, 17.66] ; total length: 42.37min
logfile66.txt ; block lengths: [32.39, 19.87] ; total length: 52.26min
logfile68.txt ; block lengths: [28.1, 15.57] ; total length: 43.67min
logfile69.txt ; block lengths: [23.05, 18.05] ; total length: 41.1min
logfile7.txt ; block lengths: [33.6, 26.51] ; total length: 60.11min
logfile70.txt ; block lengths: [31.48, 24.41] ; total length: 55.89min
logfile71.txt ; block lengths: [27.58, 20.44] ; total length: 48.02min
logfile72.txt ; block lengths: [25.69, 17.81] ; total length: 43.5min
logfile73.txt ; block lengths: [25.91, 16.93] ; total length: 42.84min
logfile74.txt ; block lengths: [35.31, 20.77] ; total length: 56.08min
logfile75.txt ; block lengths: [26.3, 18.94] ; total length: 45.24min
logfile76.txt ; block lengths: [33.0, 20.69] ; total length: 53.69min
logfile77.txt ; block lengths: [35.01, 31.8] ; total length: 66.81min
logfile78.txt ; block lengths: [22.86, 17.28] ; total length: 40.14min
logfile79.txt ; block lengths: [34.0, 27.21] ; total length: 61.21min
logfile8.txt ; block lengths: [24.59, 21.61] ; total length: 46.2min
logfile80.txt ; block lengths: [27.22, 18.28] ; total length: 45.5min
logfile81.txt ; block lengths: [30.36, 23.79] ; total length: 54.15min
logfile82.txt ; block lengths: [20.87, 15.9] ; total length: 36.77min
logfile83.txt ; block lengths: [21.49, 17.85] ; total length: 39.34min
logfile84.txt ; block lengths: [20.34, 13.55] ; total length: 33.89min
logfile85.txt ; block lengths: [29.28, 26.23] ; total length: 55.51min
logfile86.txt ; block lengths: [34.36, 12.9] ; total length: 47.26min
logfile87.txt ; block lengths: [26.84, 22.74] ; total length: 49.58min
logfile88.txt ; block lengths: [20.99, 19.57] ; total length: 40.56min
logfile89.txt ; block lengths: [36.87, 24.96] ; total length: 61.83min
logfile9.txt ; block lengths: [27.05, 20.64] ; total length: 47.69min
logfile90.txt ; block lengths: [26.37, 16.9] ; total length: 43.27min
logfile91.txt ; block lengths: [31.81, 26.46] ; total length: 58.27min
logfile92.txt ; block lengths: [26.69, 21.28] ; total length: 47.97min
logfile93.txt ; block lengths: [23.13, 24.44] ; total length: 47.57min
logfile94.txt ; block lengths: [28.5, 25.29] ; total length: 53.79min
logfile95.txt ; block lengths: [36.09, 23.99] ; total length: 60.08min
logfile96.txt ; block lengths: [28.45, 19.04] ; total length: 47.49min
```

### Summary statistics for time taken¶

In [15]:

```
pd.DataFrame( allTimes ).describe()
```

Out[15]:

|  | 0 |
| --- | --- |
| count | 94.000000 |
| mean | 49.935638 |
| std | 9.202309 |
| min | 32.560000 |
| 25% | 43.327500 |
| 50% | 48.755000 |
| 75% | 55.440000 |
| max | 71.790000 |

### Transform to Pandas dataFrame¶

In [16]:

```
def makePandas(filename):
    # we load the csv into pandas
    df = pd.read_csv(open(filename,'r'),
                skiprows=6,
                header=0,
                sep='\t')
    
    # the index in passed into a column, so we do not loose it when reindexing
    df['id'] = df.index
    # we sort the data frame by the values we want to use for the (mulit)index
    df = df.sort_values(by='express')
    df = df.sort_values(by='ident')
    # we set which variables are the new multi-index
    df = df.set_index(['ident','express','id'],drop=False)
    # we rename the variables because to avoid ambiguity
    df.rename(columns={'ident': 'i', 'express': 'e','id':'#'}, inplace=True)
    ### ugly hack to make index hierarchical
    df = df.unstack(0).stack(1).unstack(0).stack(1).unstack(0).stack(1)
    
    return df
```

In [17]:

```
df = makePandas(logList[-1])
```

We have a multi-index with three variables: the identity of the face (male/female), the facial expression and the trial number (id)

In [18]:

```
df.head()
```

Out[18]:

|  |  |  | time | cumtime | e | i | button | filename | evaluation | stopRT | choiceRT | maskNum | maskList | # |
| --- | --- | --- | --- | --- | --- | --- | --- | --- | --- | --- | --- | --- | --- | --- |
| ident | express | id |  |  |  |  |  |  |  |  |  |  |  |  |
| 0 | 0 | 9 | 2016-03-10 15:08:11 | 198.14952 | 0.0 | 0.0 | hap | img/f\_hap\_cut.png | HIT | 12275.0 | 16115.0 | 13.0 | 3-28-29-7-24-44-45-22-10-43-30-18-34 | 9.0 |
|  |  | 27 | 2016-03-10 15:15:00 | 607.15364 | 0.0 | 0.0 | hap | img/f\_hap\_cut.png | HIT | 19242.0 | 20994.0 | 20.0 | 40-15-0-37-1-27-45-46-44-23-18-43-22-3-41-29-1... | 27.0 |
|  |  | 37 | 2016-03-10 15:18:36 | 823.46331 | 0.0 | 0.0 | hap | img/f\_hap\_cut.png | HIT | 16303.0 | 17847.0 | 17.0 | 9-44-23-35-39-14-1-40-46-19-28-20-26-37-17-21-30 | 37.0 |
|  |  | 43 | 2016-03-10 15:20:31 | 937.80766 | 0.0 | 0.0 | hap | img/f\_hap\_cut.png | HIT | 5062.0 | 7262.0 | 6.0 | 36-29-47-3-43-20 | 43.0 |
|  |  | 58 | 2016-03-10 15:23:36 | 1123.17332 | 0.0 | 0.0 | hap | img/f\_hap\_cut.png | HIT | 5377.0 | 6801.0 | 6.0 | 19-20-16-39-13-44 | 58.0 |

Given that there are 2 faces times 7 expressions times 16 repetitions, there should be 2x7x16=224 entries in each DataFrame

In [19]:

```
for logFile in logList:
    thisDf = makePandas(logFile)
    assert len(list(thisDf.index)) == 2*7*16, "wrong number of entries in df %s" % logFile
    print logFile, '\tnumber of entries: ', len(list(thisDf.index))
```

```
../experiment/app/static/logfiles/logfile1.txt 	number of entries:  224
../experiment/app/static/logfiles/logfile11.txt 	number of entries:  224
../experiment/app/static/logfiles/logfile12.txt 	number of entries:  224
../experiment/app/static/logfiles/logfile13.txt 	number of entries:  224
../experiment/app/static/logfiles/logfile14.txt 	number of entries:  224
../experiment/app/static/logfiles/logfile15.txt 	number of entries:  224
../experiment/app/static/logfiles/logfile16.txt 	number of entries:  224
../experiment/app/static/logfiles/logfile17.txt 	number of entries:  224
../experiment/app/static/logfiles/logfile18.txt 	number of entries:  224
../experiment/app/static/logfiles/logfile19.txt 	number of entries:  224
../experiment/app/static/logfiles/logfile2.txt 	number of entries:  224
../experiment/app/static/logfiles/logfile20.txt 	number of entries:  224
../experiment/app/static/logfiles/logfile21.txt 	number of entries:  224
../experiment/app/static/logfiles/logfile22.txt 	number of entries:  224
../experiment/app/static/logfiles/logfile23.txt 	number of entries:  224
../experiment/app/static/logfiles/logfile24.txt 	number of entries:  224
../experiment/app/static/logfiles/logfile25.txt 	number of entries:  224
../experiment/app/static/logfiles/logfile26.txt 	number of entries:  224
../experiment/app/static/logfiles/logfile27.txt 	number of entries:  224
../experiment/app/static/logfiles/logfile28.txt 	number of entries:  224
../experiment/app/static/logfiles/logfile29.txt 	number of entries:  224
../experiment/app/static/logfiles/logfile3.txt 	number of entries:  224
../experiment/app/static/logfiles/logfile30.txt 	number of entries:  224
../experiment/app/static/logfiles/logfile31.txt 	number of entries:  224
../experiment/app/static/logfiles/logfile32.txt 	number of entries:  224
../experiment/app/static/logfiles/logfile33.txt 	number of entries:  224
../experiment/app/static/logfiles/logfile34.txt 	number of entries:  224
../experiment/app/static/logfiles/logfile35.txt 	number of entries:  224
../experiment/app/static/logfiles/logfile36.txt 	number of entries:  224
../experiment/app/static/logfiles/logfile37.txt 	number of entries:  224
../experiment/app/static/logfiles/logfile38.txt 	number of entries:  224
../experiment/app/static/logfiles/logfile39.txt 	number of entries:  224
../experiment/app/static/logfiles/logfile4.txt 	number of entries:  224
../experiment/app/static/logfiles/logfile40.txt 	number of entries:  224
../experiment/app/static/logfiles/logfile41.txt 	number of entries:  224
../experiment/app/static/logfiles/logfile42.txt 	number of entries:  224
../experiment/app/static/logfiles/logfile43.txt 	number of entries:  224
../experiment/app/static/logfiles/logfile44.txt 	number of entries:  224
../experiment/app/static/logfiles/logfile45.txt 	number of entries:  224
../experiment/app/static/logfiles/logfile46.txt 	number of entries:  224
../experiment/app/static/logfiles/logfile47.txt 	number of entries:  224
../experiment/app/static/logfiles/logfile48.txt 	number of entries:  224
../experiment/app/static/logfiles/logfile49.txt 	number of entries:  224
../experiment/app/static/logfiles/logfile5.txt 	number of entries:  224
../experiment/app/static/logfiles/logfile50.txt 	number of entries:  224
../experiment/app/static/logfiles/logfile51.txt 	number of entries:  224
../experiment/app/static/logfiles/logfile52.txt 	number of entries:  224
../experiment/app/static/logfiles/logfile53.txt 	number of entries:  224
../experiment/app/static/logfiles/logfile54.txt 	number of entries:  224
../experiment/app/static/logfiles/logfile55.txt 	number of entries:  224
../experiment/app/static/logfiles/logfile56.txt 	number of entries:  224
../experiment/app/static/logfiles/logfile57.txt 	number of entries:  224
../experiment/app/static/logfiles/logfile58.txt 	number of entries:  224
../experiment/app/static/logfiles/logfile59.txt 	number of entries:  224
../experiment/app/static/logfiles/logfile6.txt 	number of entries:  224
../experiment/app/static/logfiles/logfile60.txt 	number of entries:  224
../experiment/app/static/logfiles/logfile61.txt 	number of entries:  224
../experiment/app/static/logfiles/logfile62.txt 	number of entries:  224
../experiment/app/static/logfiles/logfile63.txt 	number of entries:  224
../experiment/app/static/logfiles/logfile64.txt 	number of entries:  224
../experiment/app/static/logfiles/logfile65.txt 	number of entries:  224
../experiment/app/static/logfiles/logfile66.txt 	number of entries:  224
../experiment/app/static/logfiles/logfile68.txt 	number of entries:  224
../experiment/app/static/logfiles/logfile69.txt 	number of entries:  224
../experiment/app/static/logfiles/logfile7.txt 	number of entries:  224
../experiment/app/static/logfiles/logfile70.txt 	number of entries:  224
../experiment/app/static/logfiles/logfile71.txt 	number of entries:  224
../experiment/app/static/logfiles/logfile72.txt 	number of entries:  224
../experiment/app/static/logfiles/logfile73.txt 	number of entries:  224
../experiment/app/static/logfiles/logfile74.txt 	number of entries:  224
../experiment/app/static/logfiles/logfile75.txt 	number of entries:  224
../experiment/app/static/logfiles/logfile76.txt 	number of entries:  224
../experiment/app/static/logfiles/logfile77.txt 	number of entries:  224
../experiment/app/static/logfiles/logfile78.txt 	number of entries:  224
../experiment/app/static/logfiles/logfile79.txt 	number of entries:  224
../experiment/app/static/logfiles/logfile8.txt 	number of entries:  224
../experiment/app/static/logfiles/logfile80.txt 	number of entries:  224
../experiment/app/static/logfiles/logfile81.txt 	number of entries:  224
../experiment/app/static/logfiles/logfile82.txt 	number of entries:  224
../experiment/app/static/logfiles/logfile83.txt 	number of entries:  224
../experiment/app/static/logfiles/logfile84.txt 	number of entries:  224
../experiment/app/static/logfiles/logfile85.txt 	number of entries:  224
../experiment/app/static/logfiles/logfile86.txt 	number of entries:  224
../experiment/app/static/logfiles/logfile87.txt 	number of entries:  224
../experiment/app/static/logfiles/logfile88.txt 	number of entries:  224
../experiment/app/static/logfiles/logfile89.txt 	number of entries:  224
../experiment/app/static/logfiles/logfile9.txt 	number of entries:  224
../experiment/app/static/logfiles/logfile90.txt 	number of entries:  224
../experiment/app/static/logfiles/logfile91.txt 	number of entries:  224
../experiment/app/static/logfiles/logfile92.txt 	number of entries:  224
../experiment/app/static/logfiles/logfile93.txt 	number of entries:  224
../experiment/app/static/logfiles/logfile94.txt 	number of entries:  224
../experiment/app/static/logfiles/logfile95.txt 	number of entries:  224
../experiment/app/static/logfiles/logfile96.txt 	number of entries:  224
```

### Save as csv¶

In [20]:

```
def saveCsv(filelist):
    for filename in filelist:
        df = makePandas(filename)
                
        # define a name for the csv file that is created
        pName = filename[filename.rfind('/logfile')+len('/logfile'):filename.rfind('.')]
        csvName = 'pandas_logfile'+ ('000'+pName)[-3:] + '.csv'
        
        # check if the number of trials is correct
        correctNumber = 7*2*8*2 # there are 7 emotions, 2 identities, 8 repetitions per block and 2 blocks (=224)
        
        if len(df.index.levels[-1]) != correctNumber:
            csvName = 'invalid_'+csvName
        else: 
            pass
        
        print "...saving", csvName
        
        df.to_csv('../rawTables/'+csvName)
```

In [21]:

```
saveCsv(logList)
```

```
...saving pandas_logfile001.csv
...saving pandas_logfile011.csv
...saving pandas_logfile012.csv
...saving pandas_logfile013.csv
...saving pandas_logfile014.csv
...saving pandas_logfile015.csv
...saving pandas_logfile016.csv
...saving pandas_logfile017.csv
...saving pandas_logfile018.csv
...saving pandas_logfile019.csv
...saving pandas_logfile002.csv
...saving pandas_logfile020.csv
...saving pandas_logfile021.csv
...saving pandas_logfile022.csv
...saving pandas_logfile023.csv
...saving pandas_logfile024.csv
...saving pandas_logfile025.csv
...saving pandas_logfile026.csv
...saving pandas_logfile027.csv
...saving pandas_logfile028.csv
...saving pandas_logfile029.csv
...saving pandas_logfile003.csv
...saving pandas_logfile030.csv
...saving pandas_logfile031.csv
...saving pandas_logfile032.csv
...saving pandas_logfile033.csv
...saving pandas_logfile034.csv
...saving pandas_logfile035.csv
...saving pandas_logfile036.csv
...saving pandas_logfile037.csv
...saving pandas_logfile038.csv
...saving pandas_logfile039.csv
...saving pandas_logfile004.csv
...saving pandas_logfile040.csv
...saving pandas_logfile041.csv
...saving pandas_logfile042.csv
...saving pandas_logfile043.csv
...saving pandas_logfile044.csv
...saving pandas_logfile045.csv
...saving pandas_logfile046.csv
...saving pandas_logfile047.csv
...saving pandas_logfile048.csv
...saving pandas_logfile049.csv
...saving pandas_logfile005.csv
...saving pandas_logfile050.csv
...saving pandas_logfile051.csv
...saving pandas_logfile052.csv
...saving pandas_logfile053.csv
...saving pandas_logfile054.csv
...saving pandas_logfile055.csv
...saving pandas_logfile056.csv
...saving pandas_logfile057.csv
...saving pandas_logfile058.csv
...saving pandas_logfile059.csv
...saving pandas_logfile006.csv
...saving pandas_logfile060.csv
...saving pandas_logfile061.csv
...saving pandas_logfile062.csv
...saving pandas_logfile063.csv
...saving pandas_logfile064.csv
...saving pandas_logfile065.csv
...saving pandas_logfile066.csv
...saving pandas_logfile068.csv
...saving pandas_logfile069.csv
...saving pandas_logfile007.csv
...saving pandas_logfile070.csv
...saving pandas_logfile071.csv
...saving pandas_logfile072.csv
...saving pandas_logfile073.csv
...saving pandas_logfile074.csv
...saving pandas_logfile075.csv
...saving pandas_logfile076.csv
...saving pandas_logfile077.csv
...saving pandas_logfile078.csv
...saving pandas_logfile079.csv
...saving pandas_logfile008.csv
...saving pandas_logfile080.csv
...saving pandas_logfile081.csv
...saving pandas_logfile082.csv
...saving pandas_logfile083.csv
...saving pandas_logfile084.csv
...saving pandas_logfile085.csv
...saving pandas_logfile086.csv
...saving pandas_logfile087.csv
...saving pandas_logfile088.csv
...saving pandas_logfile089.csv
...saving pandas_logfile009.csv
...saving pandas_logfile090.csv
...saving pandas_logfile091.csv
...saving pandas_logfile092.csv
...saving pandas_logfile093.csv
...saving pandas_logfile094.csv
...saving pandas_logfile095.csv
...saving pandas_logfile096.csv
```

### Sort the list of logfiles in ascending order¶

In [22]:

```
pandasList = getLogfile('../rawTables/','pandas_*')
pandasList.sort()
```

Now, we have a df with the following multi-index:

- identity (0=female, 1=male)
- expression (0=happy, ... 6=neutral)
- id (ascending number as experiment progresses)

### Load the csv¶

In [23]:

```
df = pd.read_csv('../rawTables/pandas_logfile069.csv',index_col=[0,1,2],header=0)
```

Example:

In [24]:

```
df.head()
```

Out[24]:

|  |  |  | time | cumtime | e | i | button | filename | evaluation | stopRT | choiceRT | maskNum | maskList | # |
| --- | --- | --- | --- | --- | --- | --- | --- | --- | --- | --- | --- | --- | --- | --- |
| ident | express | id |  |  |  |  |  |  |  |  |  |  |  |  |
| 0 | 0 | 13 | 2016-01-19 10:07:51 | 255.73029 | 0.0 | 0.0 | hap | img/f\_hap\_cut.png | HIT | 5152.0 | 7296.0 | 6.0 | 42-44-21-13-33-15 | 13.0 |
|  |  | 26 | 2016-01-19 10:10:36 | 420.75442 | 0.0 | 0.0 | hap | img/f\_hap\_cut.png | HIT | 2742.0 | 4079.0 | 3.0 | 13-30-41 | 26.0 |
|  |  | 35 | 2016-01-19 10:12:38 | 542.74912 | 0.0 | 0.0 | hap | img/f\_hap\_cut.png | HIT | 17895.0 | 19008.0 | 18.0 | 40-46-3-47-32-5-41-0-12-17-19-14-22-34-24-8-2-21 | 35.0 |
|  |  | 44 | 2016-01-19 10:14:27 | 652.15954 | 0.0 | 0.0 | hap | img/f\_hap\_cut.png | HIT | 5905.0 | 7025.0 | 6.0 | 8-12-4-30-40-13 | 44.0 |
|  |  | 62 | 2016-01-19 10:18:11 | 875.79968 | 0.0 | 0.0 | hap | img/f\_hap\_cut.png | HIT | 7089.0 | 8969.0 | 8.0 | 22-36-35-25-39-2-30-15 | 62.0 |

### Check if logfiles are not corrupted¶

In [25]:

```
for pandasFile in pandasList:
    print "checking logfile %s ..." % pandasFile
    # load each stored df into pandas
    thisDf = pd.read_csv(pandasFile,index_col=[0,1,2],header=0)
    #loop through identities
    for ident in thisDf.index.levels[0]:
        # loop through expressions
        for express in thisDf.index.levels[1]:

            # This is to double-check if each condition (expression of a particular face)
            # has always exactly 16 trials (there a two blocks a 8 trials per condition)
            assert len(thisDf.ix[ident].ix[express]) ==16 ,'trial numbers corrupted'

            # This is to double-check whether the number of revealed tiles in the
            # variable maskNum is equal to the number of items in the maskList.
            for entry in thisDf.ix[ident].ix[express].index:
                #print entry,df.ix[ident].ix[express].ix[entry]['maskNum'],len(df.ix[ident].ix[express].ix[entry]['maskList'].split('-'))
                assert thisDf.ix[ident].ix[express].ix[entry]['maskNum']==len(thisDf.ix[ident].ix[express].ix[entry]['maskList'].split('-')),'mask numbers corrupted'
```

```
checking logfile ../rawTables/pandas_logfile001.csv ...
checking logfile ../rawTables/pandas_logfile002.csv ...
checking logfile ../rawTables/pandas_logfile003.csv ...
checking logfile ../rawTables/pandas_logfile004.csv ...
checking logfile ../rawTables/pandas_logfile005.csv ...
checking logfile ../rawTables/pandas_logfile006.csv ...
checking logfile ../rawTables/pandas_logfile007.csv ...
checking logfile ../rawTables/pandas_logfile008.csv ...
checking logfile ../rawTables/pandas_logfile009.csv ...
checking logfile ../rawTables/pandas_logfile011.csv ...
checking logfile ../rawTables/pandas_logfile012.csv ...
checking logfile ../rawTables/pandas_logfile013.csv ...
checking logfile ../rawTables/pandas_logfile014.csv ...
checking logfile ../rawTables/pandas_logfile015.csv ...
checking logfile ../rawTables/pandas_logfile016.csv ...
checking logfile ../rawTables/pandas_logfile017.csv ...
checking logfile ../rawTables/pandas_logfile018.csv ...
checking logfile ../rawTables/pandas_logfile019.csv ...
checking logfile ../rawTables/pandas_logfile020.csv ...
checking logfile ../rawTables/pandas_logfile021.csv ...
checking logfile ../rawTables/pandas_logfile022.csv ...
checking logfile ../rawTables/pandas_logfile023.csv ...
checking logfile ../rawTables/pandas_logfile024.csv ...
checking logfile ../rawTables/pandas_logfile025.csv ...
checking logfile ../rawTables/pandas_logfile026.csv ...
checking logfile ../rawTables/pandas_logfile027.csv ...
checking logfile ../rawTables/pandas_logfile028.csv ...
checking logfile ../rawTables/pandas_logfile029.csv ...
checking logfile ../rawTables/pandas_logfile030.csv ...
checking logfile ../rawTables/pandas_logfile031.csv ...
checking logfile ../rawTables/pandas_logfile032.csv ...
checking logfile ../rawTables/pandas_logfile033.csv ...
checking logfile ../rawTables/pandas_logfile034.csv ...
checking logfile ../rawTables/pandas_logfile035.csv ...
checking logfile ../rawTables/pandas_logfile036.csv ...
checking logfile ../rawTables/pandas_logfile037.csv ...
checking logfile ../rawTables/pandas_logfile038.csv ...
checking logfile ../rawTables/pandas_logfile039.csv ...
checking logfile ../rawTables/pandas_logfile040.csv ...
checking logfile ../rawTables/pandas_logfile041.csv ...
checking logfile ../rawTables/pandas_logfile042.csv ...
checking logfile ../rawTables/pandas_logfile043.csv ...
checking logfile ../rawTables/pandas_logfile044.csv ...
checking logfile ../rawTables/pandas_logfile045.csv ...
checking logfile ../rawTables/pandas_logfile046.csv ...
checking logfile ../rawTables/pandas_logfile047.csv ...
checking logfile ../rawTables/pandas_logfile048.csv ...
checking logfile ../rawTables/pandas_logfile049.csv ...
checking logfile ../rawTables/pandas_logfile050.csv ...
checking logfile ../rawTables/pandas_logfile051.csv ...
checking logfile ../rawTables/pandas_logfile052.csv ...
checking logfile ../rawTables/pandas_logfile053.csv ...
checking logfile ../rawTables/pandas_logfile054.csv ...
checking logfile ../rawTables/pandas_logfile055.csv ...
checking logfile ../rawTables/pandas_logfile056.csv ...
checking logfile ../rawTables/pandas_logfile057.csv ...
checking logfile ../rawTables/pandas_logfile058.csv ...
checking logfile ../rawTables/pandas_logfile059.csv ...
checking logfile ../rawTables/pandas_logfile060.csv ...
checking logfile ../rawTables/pandas_logfile061.csv ...
checking logfile ../rawTables/pandas_logfile062.csv ...
checking logfile ../rawTables/pandas_logfile063.csv ...
checking logfile ../rawTables/pandas_logfile064.csv ...
checking logfile ../rawTables/pandas_logfile065.csv ...
checking logfile ../rawTables/pandas_logfile066.csv ...
checking logfile ../rawTables/pandas_logfile068.csv ...
checking logfile ../rawTables/pandas_logfile069.csv ...
checking logfile ../rawTables/pandas_logfile070.csv ...
checking logfile ../rawTables/pandas_logfile071.csv ...
checking logfile ../rawTables/pandas_logfile072.csv ...
checking logfile ../rawTables/pandas_logfile073.csv ...
checking logfile ../rawTables/pandas_logfile074.csv ...
checking logfile ../rawTables/pandas_logfile075.csv ...
checking logfile ../rawTables/pandas_logfile076.csv ...
checking logfile ../rawTables/pandas_logfile077.csv ...
checking logfile ../rawTables/pandas_logfile078.csv ...
checking logfile ../rawTables/pandas_logfile079.csv ...
checking logfile ../rawTables/pandas_logfile080.csv ...
checking logfile ../rawTables/pandas_logfile081.csv ...
checking logfile ../rawTables/pandas_logfile082.csv ...
checking logfile ../rawTables/pandas_logfile083.csv ...
checking logfile ../rawTables/pandas_logfile084.csv ...
checking logfile ../rawTables/pandas_logfile085.csv ...
checking logfile ../rawTables/pandas_logfile086.csv ...
checking logfile ../rawTables/pandas_logfile087.csv ...
checking logfile ../rawTables/pandas_logfile088.csv ...
checking logfile ../rawTables/pandas_logfile089.csv ...
checking logfile ../rawTables/pandas_logfile090.csv ...
checking logfile ../rawTables/pandas_logfile091.csv ...
checking logfile ../rawTables/pandas_logfile092.csv ...
checking logfile ../rawTables/pandas_logfile093.csv ...
checking logfile ../rawTables/pandas_logfile094.csv ...
checking logfile ../rawTables/pandas_logfile095.csv ...
checking logfile ../rawTables/pandas_logfile096.csv ...
```
