# Supplementary material for: Mapping the emotional face. How individual face parts contribute to successful emotion recognition
Source: PLoS One. 2017 May 11;12(5):e0177239. doi: 10.1371/journal.pone.0177239 (PMC5426715; doi:10.1371/journal.pone.0177239)
Supplement: S3 Code — (HTML) [file pone.0177239.s005.html]

code002\_globalMetrics


# Mapping the emotional face. How individual face parts contribute to successful emotion recognition.

# 2. Global Counting¶

Here, we look at global metrics of each face. The main metrics are %correct and %tiles revealed.

### Basic Imports¶

In [1]:

```
from myBasics import *
%matplotlib inline
```

In [2]:

```
logList = getFile('../rawTables/','pand*.csv')
```

Loading data of one participant for illustration purposes:

In [3]:

```
testDf = pd.read_csv(logList[-1],
            header=0,
            index_col=[0,1,2]
           )
```

In [4]:

```
testDf.head()
```

Out[4]:

|  |  |  | time | cumtime | e | i | button | filename | evaluation | stopRT | choiceRT | maskNum | maskList | # |
| --- | --- | --- | --- | --- | --- | --- | --- | --- | --- | --- | --- | --- | --- | --- |
| ident | express | id |  |  |  |  |  |  |  |  |  |  |  |  |
| 0 | 0 | 9 | 2016-03-10 15:08:11 | 198.14952 | 0.0 | 0.0 | hap | img/f\_hap\_cut.png | HIT | 12275.0 | 16115.0 | 13.0 | 3-28-29-7-24-44-45-22-10-43-30-18-34 | 9.0 |
|  |  | 27 | 2016-03-10 15:15:00 | 607.15364 | 0.0 | 0.0 | hap | img/f\_hap\_cut.png | HIT | 19242.0 | 20994.0 | 20.0 | 40-15-0-37-1-27-45-46-44-23-18-43-22-3-41-29-1... | 27.0 |
|  |  | 37 | 2016-03-10 15:18:36 | 823.46331 | 0.0 | 0.0 | hap | img/f\_hap\_cut.png | HIT | 16303.0 | 17847.0 | 17.0 | 9-44-23-35-39-14-1-40-46-19-28-20-26-37-17-21-30 | 37.0 |
|  |  | 43 | 2016-03-10 15:20:31 | 937.80766 | 0.0 | 0.0 | hap | img/f\_hap\_cut.png | HIT | 5062.0 | 7262.0 | 6.0 | 36-29-47-3-43-20 | 43.0 |
|  |  | 58 | 2016-03-10 15:23:36 | 1123.17332 | 0.0 | 0.0 | hap | img/f\_hap\_cut.png | HIT | 5377.0 | 6801.0 | 6.0 | 19-20-16-39-13-44 | 58.0 |

### get number of revealed tiles¶

In [5]:

```
def getMaskNums(df):
    # pre-formatted dict to write to
    d = {0:{'hap':{}, 'sad':{}, 'ang':{}, 'fea':{}, 'dis':{}, 'sup':{}, 'ntr':{} },
         1:{'hap':{}, 'sad':{}, 'ang':{}, 'fea':{}, 'dis':{}, 'sup':{}, 'ntr':{} }
        }

    # loop trough all rows
    for i,ident in enumerate(df.index.labels[0]):
        # get the current emotion and trial number
        express = df.index.labels[1][i]
        trial =  df.index.labels[2][i]
        # get the string emotion name corresponding to the number
        actual = myLabels[express]
        # get the decision
        decision = df.ix[ident].ix[express].ix[trial]["button"]
        # get the number of revealed tiles
        maskNum = df.ix[ident].ix[express].ix[trial]["maskNum"]
    
        # put number of revealed tiles into dict, according to what face it
        # belongs to and what answer was given
        try:
            d[ident][actual][decision].append(maskNum)
        except:
            d[ident][actual][decision] = [maskNum]

    return d
```

Example:

Get all the number of revealed tiles for each trial for the male (1) fearful (fea) face. Here we see that we not only get the number of revealed tiles for each trial, but also information about what answer was given in that trial. In the present example, most of the 16 trials of the male fearful face were (correctly) labelled as fearful and in three cases, fear was misclassified as surprised (a plausible mistake)

In [6]:

```
getMaskNums(testDf)[1]['fea']
```

Out[6]:

```
{'dis': [8.0],
 'fea': [19.0, 21.0, 18.0, 5.0, 9.0, 20.0, 9.0, 7.0, 6.0, 8.0],
 'sad': [4.0, 6.0, 4.0],
 'sup': [18.0, 12.0]}
```

### average across conditions¶

In [7]:

```
def getAvgMaskNums(d,pName):
    # i
    newD = {0:{'hap':{}, 'sad':{}, 'ang':{}, 'fea':{}, 'dis':{}, 'sup':{}, 'ntr':{} },
            1:{'hap':{}, 'sad':{}, 'ang':{}, 'fea':{}, 'dis':{}, 'sup':{}, 'ntr':{} }
        }

    for ident in newD:
        # what emotion does the face have
        for actual in newD[ident]:
            # here, we can loop through the same keys for the type of answer
            for decision in newD[ident]:
                # since some keys may not be there, we have to try:
                try:
                    newD[ident][actual][decision] = np.array(d[ident][actual][decision]).mean()
                except:
                    newD[ident][actual][decision] = np.nan
    
    # seperately for the female and male face
    fDf = pd.DataFrame(newD[0])
    fDf.index = [[pName]*7,['f']*7, fDf.index]
    mDf = pd.DataFrame(newD[1])
    mDf.index = [[pName]*7,['m']*7,mDf.index]

    rawDf = pd.concat([ fDf,mDf])
    return rawDf
```

Example:

Show the average number of revealed tiles for one participant, across all trial for the fearful face

In [8]:

```
getAvgMaskNums(getMaskNums(testDf),'test')['fea']
```

Out[8]:

```
test  f  ang    16.800000
         dis          NaN
         fea    15.000000
         hap          NaN
         ntr          NaN
         sad     5.500000
         sup    11.250000
      m  ang          NaN
         dis     8.000000
         fea    12.200000
         hap          NaN
         ntr          NaN
         sad     4.666667
         sup    15.000000
Name: fea, dtype: float64
```

Do this for all participants:

In [9]:

```
def makeBigMask(logList):
    
    bigDf = pd.DataFrame()

    for logFile in logList:
        pName = "p"+logFile[logFile.rfind('e')+1:logFile.rfind('.')]

        df = pd.read_csv(logFile,
                header=0,
                index_col=[0,1,2]
               )

        countDf = getMaskNums(df)

        thisDf = getAvgMaskNums(countDf,pName)

        bigDf = pd.concat([bigDf,thisDf])
    
    # output as percentage
    return bigDf/48.*100
```

In [10]:

```
bigMask = makeBigMask(logList)
```

Example:

Show the average number of revealed tiles for all participants

In [11]:

```
bigMask.tail()
```

Out[11]:

|  |  |  | ang | dis | fea | hap | ntr | sad | sup |
| --- | --- | --- | --- | --- | --- | --- | --- | --- | --- |
| p096 | m | fea | 33.333333 | 16.666667 | 25.416667 | NaN | 41.250000 | 23.437500 | 33.333333 |
|  |  | hap | NaN | NaN | NaN | 21.354167 | NaN | NaN | NaN |
|  |  | ntr | 4.166667 | NaN | NaN | NaN | 37.916667 | NaN | NaN |
|  |  | sad | 39.583333 | NaN | 9.722222 | NaN | 29.513889 | 30.416667 | NaN |
|  |  | sup | 39.583333 | NaN | 31.250000 | NaN | NaN | 10.000000 | 24.107143 |

Restructure the DataFrame:

In [12]:

```
stackMask = bigMask.stack(0).unstack(1).unstack(-1).unstack(1)
```

Example:

In [13]:

```
stackMask.tail()
```

Out[13]:

|  | f | | | | | | | | | | | | | | | | | | | | | | | | | | | | | | | | | | | | | | | | | | | | | | | | | m | | | | | | | | | | | | | | | | | | | | | | | | | | | | | | | | | | | | | | | | | | | | | | | | |
| --- | --- | --- | --- | --- | --- | --- | --- | --- | --- | --- | --- | --- | --- | --- | --- | --- | --- | --- | --- | --- | --- | --- | --- | --- | --- | --- | --- | --- | --- | --- | --- | --- | --- | --- | --- | --- | --- | --- | --- | --- | --- | --- | --- | --- | --- | --- | --- | --- | --- | --- | --- | --- | --- | --- | --- | --- | --- | --- | --- | --- | --- | --- | --- | --- | --- | --- | --- | --- | --- | --- | --- | --- | --- | --- | --- | --- | --- | --- | --- | --- | --- | --- | --- | --- | --- | --- | --- | --- | --- | --- | --- | --- | --- | --- | --- | --- | --- | --- |
|  | ang | | | | | | | dis | | | | | | | fea | | | | | | | hap | | | | | | | ntr | | | | | | | sad | | | | | | | sup | | | | | | | ang | | | | | | | dis | | | | | | | fea | | | | | | | hap | | | | | | | ntr | | | | | | | sad | | | | | | | sup | | | | | | |
|  | ang | dis | fea | hap | ntr | sad | sup | ang | dis | fea | hap | ntr | sad | sup | ang | dis | fea | hap | ntr | sad | sup | ang | dis | fea | hap | ntr | sad | sup | ang | dis | fea | hap | ntr | sad | sup | ang | dis | fea | hap | ntr | sad | sup | ang | dis | fea | hap | ntr | sad | sup | ang | dis | fea | hap | ntr | sad | sup | ang | dis | fea | hap | ntr | sad | sup | ang | dis | fea | hap | ntr | sad | sup | ang | dis | fea | hap | ntr | sad | sup | ang | dis | fea | hap | ntr | sad | sup | ang | dis | fea | hap | ntr | sad | sup | ang | dis | fea | hap | ntr | sad | sup |
| p092 | 16.666667 | 52.083333 | NaN | NaN | NaN | NaN | NaN | 14.583333 | 20.370370 | NaN | NaN | NaN | NaN | NaN | 19.791667 | 30.208333 | 25.000000 | 28.125000 | NaN | NaN | 45.833333 | NaN | NaN | NaN | 18.472222 | 29.166667 | NaN | NaN | NaN | 40.625000 | 27.083333 | NaN | 23.674242 | 10.416667 | NaN | 17.708333 | 24.583333 | 36.979167 | NaN | 28.125000 | 33.333333 | 37.5 | NaN | NaN | 16.666667 | 20.833333 | 25.0 | NaN | 31.410256 | 26.488095 | 23.697917 | NaN | NaN | 16.666667 | NaN | NaN | 17.187500 | 18.229167 | NaN | NaN | NaN | NaN | NaN | NaN | 33.333333 | 25.260417 | NaN | 25.0 | 32.291667 | 35.416667 | NaN | NaN | 10.416667 | 17.777778 | NaN | NaN | NaN | NaN | 39.583333 | 29.166667 | NaN | 23.106061 | 22.916667 | 45.833333 | 14.583333 | 23.750000 | 33.333333 | NaN | 31.944444 | NaN | 10.416667 | NaN | 11.111111 | 35.416667 | NaN | NaN | NaN | 35.714286 |
| p093 | 20.000000 | NaN | NaN | 8.333333 | NaN | NaN | NaN | 19.791667 | 18.958333 | NaN | NaN | NaN | NaN | NaN | NaN | 31.770833 | 32.407407 | NaN | NaN | 25.000000 | 25.000000 | NaN | NaN | NaN | 12.916667 | 16.666667 | NaN | NaN | NaN | NaN | 25.000000 | NaN | 18.750000 | NaN | 16.666667 | NaN | 31.250000 | 22.916667 | NaN | 16.666667 | 21.955128 | NaN | NaN | NaN | 40.277778 | 12.500000 | NaN | NaN | 19.097222 | 25.000000 | 28.472222 | NaN | NaN | 29.166667 | 32.638889 | NaN | NaN | 21.354167 | NaN | NaN | NaN | NaN | NaN | NaN | 14.583333 | 28.720238 | NaN | NaN | NaN | 18.750000 | NaN | NaN | NaN | 10.807292 | NaN | NaN | NaN | NaN | NaN | 25.000000 | NaN | 21.041667 | 27.500000 | NaN | 8.333333 | 20.833333 | 29.687500 | NaN | NaN | 20.833333 | 10.763889 | NaN | 18.750000 | 25.000000 | NaN | NaN | NaN | 28.787879 |
| p094 | 20.982143 | 6.250000 | 12.50 | NaN | NaN | NaN | NaN | 21.875000 | 20.138889 | NaN | NaN | NaN | NaN | NaN | NaN | NaN | 44.166667 | 14.583333 | 16.666667 | 6.250000 | 37.152778 | NaN | NaN | NaN | 17.317708 | NaN | NaN | NaN | NaN | 54.166667 | NaN | NaN | 30.833333 | NaN | NaN | 30.208333 | 35.416667 | 40.885417 | NaN | 23.958333 | 43.750000 | NaN | NaN | NaN | 39.583333 | NaN | NaN | NaN | 24.444444 | 33.750000 | 27.083333 | 22.916667 | NaN | 29.166667 | 29.861111 | NaN | 13.194444 | 20.512821 | NaN | NaN | NaN | NaN | NaN | 12.5 | 22.916667 | 30.833333 | NaN | NaN | NaN | 29.427083 | NaN | NaN | NaN | 20.963542 | NaN | NaN | NaN | NaN | NaN | 16.666667 | NaN | 29.166667 | 22.916667 | NaN | NaN | NaN | 39.880952 | NaN | 26.388889 | 36.805556 | NaN | NaN | NaN | NaN | NaN | NaN | NaN | 19.401042 |
| p095 | 21.614583 | NaN | NaN | NaN | NaN | NaN | NaN | 12.500000 | 19.940476 | NaN | NaN | NaN | NaN | NaN | NaN | NaN | 40.000000 | 22.916667 | NaN | NaN | 33.958333 | NaN | NaN | NaN | 12.760417 | NaN | NaN | NaN | NaN | NaN | NaN | NaN | 37.361111 | NaN | 60.416667 | NaN | 62.500000 | 42.500000 | NaN | NaN | 31.875000 | NaN | NaN | NaN | NaN | NaN | NaN | NaN | 18.750000 | 32.142857 | NaN | 39.583333 | NaN | NaN | 45.486111 | NaN | 27.083333 | 19.618056 | NaN | NaN | NaN | NaN | 8.333333 | NaN | NaN | 43.269231 | NaN | NaN | NaN | 36.805556 | NaN | NaN | NaN | 12.890625 | NaN | NaN | NaN | NaN | NaN | 50.000000 | NaN | 34.935897 | 72.916667 | 39.583333 | 25.000000 | 64.583333 | 53.958333 | NaN | NaN | 20.833333 | NaN | NaN | NaN | 33.333333 | NaN | NaN | NaN | 26.666667 |
| p096 | 14.791667 | 12.083333 | 6.25 | NaN | NaN | NaN | NaN | 22.500000 | 16.856061 | NaN | NaN | NaN | NaN | NaN | 35.000000 | NaN | 31.250000 | NaN | NaN | 11.458333 | 23.437500 | NaN | NaN | NaN | 18.333333 | 22.916667 | NaN | NaN | NaN | NaN | NaN | 16.666667 | 31.666667 | NaN | NaN | 18.333333 | NaN | 22.916667 | NaN | NaN | 23.437500 | NaN | NaN | NaN | 30.208333 | NaN | NaN | NaN | 21.279762 | 25.925926 | 36.458333 | 33.333333 | NaN | 4.166667 | 39.583333 | 39.583333 | 21.726190 | 15.104167 | 16.666667 | NaN | NaN | NaN | NaN | NaN | 16.666667 | 25.416667 | NaN | NaN | 9.722222 | 31.250000 | NaN | NaN | NaN | 21.354167 | NaN | NaN | NaN | NaN | NaN | 41.250000 | NaN | 37.916667 | 29.513889 | NaN | 40.625000 | NaN | 23.437500 | NaN | NaN | 30.416667 | 10.000000 | NaN | 10.416667 | 33.333333 | NaN | NaN | NaN | 24.107143 |

In [14]:

```
stackMask.to_csv('../outputs/stackCount.csv')
```

## Get % of correct responses:¶

### get response for each trial¶

In [15]:

```
def getFullCount(df,myLabels=myLabels):
    
    d = {0:{},1:{}}

    for i,ident in enumerate(df.index.labels[0]):
        express = df.index.labels[1][i]
        trial =  df.index.labels[2][i]

        actual = myLabels[express]
        decision = df.ix[ident].ix[express].ix[trial]["button"]

        try:
            d[ident][actual].append(decision)
        except:
            d[ident][actual] = [decision]

    fDf = pd.DataFrame(d[0])
    fDf.index = [['f']*16, fDf.index]
    mDf = pd.DataFrame(d[1])
    mDf.index = [['m']*16, mDf.index]
    rawDf = pd.concat([ fDf,mDf])
    
    return rawDf
```

Example for one participant:

In [16]:

```
getFullCount(testDf).head()
```

Out[16]:

|  |  | ang | dis | fea | hap | ntr | sad | sup |
| --- | --- | --- | --- | --- | --- | --- | --- | --- |
| f | 0 | ang | dis | sup | hap | ntr | sad | sup |
|  | 1 | ang | dis | ang | hap | ntr | ang | sup |
|  | 2 | ang | ang | ang | hap | ntr | ang | sup |
|  | 3 | ang | dis | ang | hap | ntr | fea | sup |
|  | 4 | ang | dis | fea | hap | ntr | ang | sup |

### get % count for one participant¶

In [17]:

```
def makeCountParticipant(df,pName):
    bigDf = pd.DataFrame()

    for ident in df.index.levels[0]:
        outDf = pd.DataFrame(index =  list( df.columns ))
        for entry in df.columns:
            outDf[entry] = df.ix[ident][entry].value_counts()

        outDf = (outDf.fillna(0)/16.)*100
        outDf.index = [[ident]*len(outDf.index),outDf.index]

        bigDf = pd.concat([bigDf,outDf],axis=0)

    #return bigDf
    stackDf = bigDf.unstack(0).stack(0).unstack(1)
    stackDf.index = [[pName]*len(stackDf.index),stackDf.index]
    stackDf = stackDf.sortlevel()
    
    return stackDf
```

Example:

In [18]:

```
makeCountParticipant(getFullCount(testDf),'test')
```

Out[18]:

|  |  | f | | | | | | | m | | | | | | |
| --- | --- | --- | --- | --- | --- | --- | --- | --- | --- | --- | --- | --- | --- | --- | --- |
|  |  | ang | dis | fea | hap | ntr | sad | sup | ang | dis | fea | hap | ntr | sad | sup |
| test | ang | 62.50 | 31.25 | 31.25 | 0.00 | 0.00 | 62.5 | 0.0 | 56.25 | 43.75 | 0.00 | 0.0 | 0.00 | 12.50 | 0.00 |
|  | dis | 31.25 | 68.75 | 0.00 | 0.00 | 0.00 | 0.0 | 0.0 | 12.50 | 50.00 | 6.25 | 0.0 | 0.00 | 0.00 | 6.25 |
|  | fea | 6.25 | 0.00 | 31.25 | 0.00 | 0.00 | 12.5 | 12.5 | 6.25 | 6.25 | 62.50 | 0.0 | 31.25 | 25.00 | 6.25 |
|  | hap | 0.00 | 0.00 | 0.00 | 93.75 | 6.25 | 0.0 | 0.0 | 0.00 | 0.00 | 0.00 | 100.0 | 0.00 | 0.00 | 0.00 |
|  | ntr | 0.00 | 0.00 | 0.00 | 6.25 | 93.75 | 0.0 | 0.0 | 12.50 | 0.00 | 0.00 | 0.0 | 31.25 | 0.00 | 0.00 |
|  | sad | 0.00 | 0.00 | 12.50 | 0.00 | 0.00 | 25.0 | 0.0 | 6.25 | 0.00 | 18.75 | 0.0 | 37.50 | 31.25 | 0.00 |
|  | sup | 0.00 | 0.00 | 25.00 | 0.00 | 0.00 | 0.0 | 87.5 | 6.25 | 0.00 | 12.50 | 0.0 | 0.00 | 31.25 | 87.50 |

### Do this for all participants:¶

In [19]:

```
def makeBig(logList):
    bigDf = pd.DataFrame()

    for logFile in logList:

        pName = "p"+logFile[logFile.rfind('e')+1:logFile.rfind('.')]

        df = pd.read_csv(logFile,
                header=0,
                index_col=[0,1,2]
               )

        countDf = getFullCount(df)

        thisDf = makeCountParticipant(countDf,pName)

        bigDf = pd.concat([bigDf,thisDf])
        
    bigDf = bigDf.sortlevel()
    
    return bigDf
```

In [20]:

```
bigDf = makeBig(logList)
```

Example of how the entry of one participant looks like:

In [21]:

```
bigDf.ix[bigDf.index.levels[0][-1]]
```

Out[21]:

|  | f | | | | | | | m | | | | | | |
| --- | --- | --- | --- | --- | --- | --- | --- | --- | --- | --- | --- | --- | --- | --- |
|  | ang | dis | fea | hap | ntr | sad | sup | ang | dis | fea | hap | ntr | sad | sup |
| ang | 62.50 | 31.25 | 31.25 | 0.00 | 0.00 | 62.5 | 0.0 | 56.25 | 43.75 | 0.00 | 0.0 | 0.00 | 12.50 | 0.00 |
| dis | 31.25 | 68.75 | 0.00 | 0.00 | 0.00 | 0.0 | 0.0 | 12.50 | 50.00 | 6.25 | 0.0 | 0.00 | 0.00 | 6.25 |
| fea | 6.25 | 0.00 | 31.25 | 0.00 | 0.00 | 12.5 | 12.5 | 6.25 | 6.25 | 62.50 | 0.0 | 31.25 | 25.00 | 6.25 |
| hap | 0.00 | 0.00 | 0.00 | 93.75 | 6.25 | 0.0 | 0.0 | 0.00 | 0.00 | 0.00 | 100.0 | 0.00 | 0.00 | 0.00 |
| ntr | 0.00 | 0.00 | 0.00 | 6.25 | 93.75 | 0.0 | 0.0 | 12.50 | 0.00 | 0.00 | 0.0 | 31.25 | 0.00 | 0.00 |
| sad | 0.00 | 0.00 | 12.50 | 0.00 | 0.00 | 25.0 | 0.0 | 6.25 | 0.00 | 18.75 | 0.0 | 37.50 | 31.25 | 0.00 |
| sup | 0.00 | 0.00 | 25.00 | 0.00 | 0.00 | 0.0 | 87.5 | 6.25 | 0.00 | 12.50 | 0.0 | 0.00 | 31.25 | 87.50 |

Restructure:

In [22]:

```
stackHits = bigDf.unstack(1)
```

In [23]:

```
stackHits.tail()
```

Out[23]:

|  | f | | | | | | | | | | | | | | | | | | | | | | | | | | | | | | | | | | | | | | | | | | | | | | | | | m | | | | | | | | | | | | | | | | | | | | | | | | | | | | | | | | | | | | | | | | | | | | | | | | |
| --- | --- | --- | --- | --- | --- | --- | --- | --- | --- | --- | --- | --- | --- | --- | --- | --- | --- | --- | --- | --- | --- | --- | --- | --- | --- | --- | --- | --- | --- | --- | --- | --- | --- | --- | --- | --- | --- | --- | --- | --- | --- | --- | --- | --- | --- | --- | --- | --- | --- | --- | --- | --- | --- | --- | --- | --- | --- | --- | --- | --- | --- | --- | --- | --- | --- | --- | --- | --- | --- | --- | --- | --- | --- | --- | --- | --- | --- | --- | --- | --- | --- | --- | --- | --- | --- | --- | --- | --- | --- | --- | --- | --- | --- | --- | --- | --- | --- | --- |
|  | ang | | | | | | | dis | | | | | | | fea | | | | | | | hap | | | | | | | ntr | | | | | | | sad | | | | | | | sup | | | | | | | ang | | | | | | | dis | | | | | | | fea | | | | | | | hap | | | | | | | ntr | | | | | | | sad | | | | | | | sup | | | | | | |
|  | ang | dis | fea | hap | ntr | sad | sup | ang | dis | fea | hap | ntr | sad | sup | ang | dis | fea | hap | ntr | sad | sup | ang | dis | fea | hap | ntr | sad | sup | ang | dis | fea | hap | ntr | sad | sup | ang | dis | fea | hap | ntr | sad | sup | ang | dis | fea | hap | ntr | sad | sup | ang | dis | fea | hap | ntr | sad | sup | ang | dis | fea | hap | ntr | sad | sup | ang | dis | fea | hap | ntr | sad | sup | ang | dis | fea | hap | ntr | sad | sup | ang | dis | fea | hap | ntr | sad | sup | ang | dis | fea | hap | ntr | sad | sup | ang | dis | fea | hap | ntr | sad | sup |
| p092 | 87.50 | 12.50 | 0.00 | 0.00 | 0.0 | 0.0 | 0.0 | 43.75 | 56.25 | 0.0 | 0.0 | 0.0 | 0.0 | 0.0 | 12.50 | 12.5 | 37.50 | 12.50 | 0.0 | 0.00 | 25.0 | 0.0 | 0.0 | 0.0 | 93.75 | 6.25 | 0.0 | 0.0 | 0.0 | 12.50 | 12.50 | 0.00 | 68.75 | 6.25 | 0.00 | 12.5 | 31.25 | 25.00 | 0.0 | 12.50 | 12.50 | 6.25 | 0.0 | 0.0 | 6.25 | 6.25 | 6.25 | 0.0 | 81.25 | 43.75 | 50.00 | 0.00 | 0.0 | 6.25 | 0.00 | 0.00 | 25.00 | 75.00 | 0.00 | 0.0 | 0.0 | 0.0 | 0.00 | 0.00 | 6.25 | 50.00 | 0.0 | 12.5 | 12.50 | 18.75 | 0.0 | 0.0 | 6.25 | 93.75 | 0.0 | 0.0 | 0.0 | 0.0 | 6.25 | 12.50 | 0.0 | 68.75 | 6.25 | 6.25 | 12.50 | 31.25 | 31.25 | 0.0 | 18.75 | 0.00 | 6.25 | 0.0 | 18.75 | 37.50 | 0.0 | 0.0 | 0.0 | 43.75 |
| p093 | 93.75 | 0.00 | 0.00 | 6.25 | 0.0 | 0.0 | 0.0 | 37.50 | 62.50 | 0.0 | 0.0 | 0.0 | 0.0 | 0.0 | 0.00 | 25.0 | 56.25 | 0.00 | 0.0 | 6.25 | 12.5 | 0.0 | 0.0 | 0.0 | 93.75 | 6.25 | 0.0 | 0.0 | 0.0 | 0.00 | 6.25 | 0.00 | 87.50 | 0.00 | 6.25 | 0.0 | 6.25 | 6.25 | 0.0 | 6.25 | 81.25 | 0.00 | 0.0 | 0.0 | 18.75 | 6.25 | 0.00 | 0.0 | 75.00 | 56.25 | 18.75 | 0.00 | 0.0 | 6.25 | 18.75 | 0.00 | 0.00 | 100.00 | 0.00 | 0.0 | 0.0 | 0.0 | 0.00 | 0.00 | 6.25 | 87.50 | 0.0 | 0.0 | 0.00 | 6.25 | 0.0 | 0.0 | 0.00 | 100.00 | 0.0 | 0.0 | 0.0 | 0.0 | 0.00 | 6.25 | 0.0 | 62.50 | 31.25 | 0.00 | 6.25 | 18.75 | 25.00 | 0.0 | 0.00 | 12.50 | 37.50 | 0.0 | 6.25 | 25.00 | 0.0 | 0.0 | 0.0 | 68.75 |
| p094 | 87.50 | 6.25 | 6.25 | 0.00 | 0.0 | 0.0 | 0.0 | 25.00 | 75.00 | 0.0 | 0.0 | 0.0 | 0.0 | 0.0 | 0.00 | 0.0 | 31.25 | 12.50 | 12.5 | 6.25 | 37.5 | 0.0 | 0.0 | 0.0 | 100.00 | 0.00 | 0.0 | 0.0 | 0.0 | 6.25 | 0.00 | 0.00 | 93.75 | 0.00 | 0.00 | 12.5 | 6.25 | 50.00 | 0.0 | 12.50 | 18.75 | 0.00 | 0.0 | 0.0 | 6.25 | 0.00 | 0.00 | 0.0 | 93.75 | 31.25 | 6.25 | 18.75 | 0.0 | 6.25 | 37.50 | 0.00 | 18.75 | 81.25 | 0.00 | 0.0 | 0.0 | 0.0 | 0.00 | 6.25 | 12.50 | 31.25 | 0.0 | 0.0 | 0.00 | 50.00 | 0.0 | 0.0 | 0.00 | 100.00 | 0.0 | 0.0 | 0.0 | 0.0 | 0.00 | 6.25 | 0.0 | 87.50 | 6.25 | 0.00 | 0.00 | 0.00 | 43.75 | 0.0 | 37.50 | 18.75 | 0.00 | 0.0 | 0.00 | 0.00 | 0.0 | 0.0 | 0.0 | 100.00 |
| p095 | 100.00 | 0.00 | 0.00 | 0.00 | 0.0 | 0.0 | 0.0 | 12.50 | 87.50 | 0.0 | 0.0 | 0.0 | 0.0 | 0.0 | 0.00 | 0.0 | 31.25 | 6.25 | 0.0 | 0.00 | 62.5 | 0.0 | 0.0 | 0.0 | 100.00 | 0.00 | 0.0 | 0.0 | 0.0 | 0.00 | 0.00 | 0.00 | 93.75 | 0.00 | 6.25 | 0.0 | 6.25 | 31.25 | 0.0 | 0.00 | 62.50 | 0.00 | 0.0 | 0.0 | 0.00 | 0.00 | 0.00 | 0.0 | 100.00 | 43.75 | 0.00 | 18.75 | 0.0 | 0.00 | 37.50 | 0.00 | 18.75 | 75.00 | 0.00 | 0.0 | 0.0 | 0.0 | 6.25 | 0.00 | 0.00 | 81.25 | 0.0 | 0.0 | 0.00 | 18.75 | 0.0 | 0.0 | 0.00 | 100.00 | 0.0 | 0.0 | 0.0 | 0.0 | 0.00 | 6.25 | 0.0 | 81.25 | 6.25 | 6.25 | 6.25 | 12.50 | 62.50 | 0.0 | 0.00 | 18.75 | 0.00 | 0.0 | 0.00 | 6.25 | 0.0 | 0.0 | 0.0 | 93.75 |
| p096 | 62.50 | 31.25 | 6.25 | 0.00 | 0.0 | 0.0 | 0.0 | 31.25 | 68.75 | 0.0 | 0.0 | 0.0 | 0.0 | 0.0 | 31.25 | 0.0 | 31.25 | 0.00 | 0.0 | 12.50 | 25.0 | 0.0 | 0.0 | 0.0 | 93.75 | 6.25 | 0.0 | 0.0 | 0.0 | 0.00 | 0.00 | 6.25 | 93.75 | 0.00 | 0.00 | 62.5 | 0.00 | 12.50 | 0.0 | 0.00 | 25.00 | 0.00 | 0.0 | 0.0 | 12.50 | 0.00 | 0.00 | 0.0 | 87.50 | 56.25 | 12.50 | 6.25 | 0.0 | 12.50 | 6.25 | 6.25 | 43.75 | 50.00 | 6.25 | 0.0 | 0.0 | 0.0 | 0.00 | 0.00 | 6.25 | 62.50 | 0.0 | 0.0 | 18.75 | 12.50 | 0.0 | 0.0 | 0.00 | 100.00 | 0.0 | 0.0 | 0.0 | 0.0 | 0.00 | 31.25 | 0.0 | 31.25 | 37.50 | 0.00 | 12.50 | 0.00 | 25.00 | 0.0 | 0.00 | 31.25 | 31.25 | 0.0 | 6.25 | 6.25 | 0.0 | 0.0 | 0.0 | 87.50 |

In [24]:

```
stackHits.to_csv('../outputs/stackHits.csv')
```

## Plotting all responses¶

In [25]:

```
sns.palplot(stackColors)
```

The following function is overly complicated, but it's supposed to make the plot look good.

In [26]:

```
def makeConfPlot(confDf,ax):
    
    # order of face expressions is defined by hand here, instead of
    # using the columns, so the order is as we wish it to be
    faceExpressions = ['hap','ang','sup','ntr','dis','fea','sad']
    faceAnswers = ['hap','ang','sup','ntr','dis','fea','sad']
    
    # we loop through all expressions
    for i,emo in enumerate( faceExpressions ):
        
        # since we build a stacked plot, we have to initialize a value that
        # tells us where to start. We start at the very bottom, hence 0
        sumSoFar = 0
        
        # we get the values of all conditions, and their names, as defined
        # in the index
        thisMean = confDf[emo].mean()
        thisIndex = thisMean.index
        
        # firstly, we are interested in the correct response, which will be
        # always at the bottom of the stack plot and printed in a strong color
        
        # corrPos is the position of the current expression in the list of all expressions
        corrPos = faceExpressions.index(emo)
        corrCol = emoReverse[emo]
        # and we also get the name of the answer at this position (cave: the order of lists
        # must match!)
        corrEntry = faceAnswers[corrPos]

        # we get the positions of all the other answers
        incorrAnswers = faceAnswers[:corrPos]+faceAnswers[corrPos+1:]
        
        # we get the value of the correct answer
        entry = thisMean[corrEntry]
        # we get the name of the correct answer
        answer = faceAnswers[corrPos]
        
        # we take the color list and extract the color for the correct position
        thisColor = stackColors[corrCol]
        thisHex = rgb2hex( (thisColor[0]*255,thisColor[1]*255,thisColor[2]*255) )

        # this is the first bar we generate, it starts at the bottom (sumSoFar=0) and
        # has a strong color (alpha=1)
        ax.bar(i,
                entry,
                bottom=sumSoFar,
                color=thisHex,
                label=answer,
                alpha=1.)      
        
        # to stack the rest of the bars on top, we update the sum to be
        # the value (% correct) of the current condition
        sumSoFar = entry
        
        # we loop through the remaining (incorrect answers)
        for j,answer in enumerate(incorrAnswers):
            entry = thisMean[answer]

            thisColor = stackColors[emoReverse[answer]]
            thisHex = rgb2hex( (thisColor[0]*255,thisColor[1]*255,thisColor[2]*255) )
            
            # same thing as above, but without the legend and with transparency
            ax.bar(i,
                    entry,
                    bottom=sumSoFar,
                    color=thisHex,
                    alpha=.6
                        )           
            # for each run of the loop, we update the sumSoFar so we
            # always stack each expression at the top
            sumSoFar+=entry
    
    
    # x labels for each plot
    ax.set_xlabel('Basic Expression',fontsize=14)
    # y labels only for the first plot
    ax.set_ylabel('% of responses')
    
    # axes annotation for all plots
    ax.set_xticks(np.arange(0,7.1,1)+0.4,  )
    ax.set_xticklabels(faceExpressions)
    ax.set_yticks(np.arange(0,101,10))
    ax.set_yticklabels([str(a)+'%' for a in  np.arange(0,101,10)] )
    ax.set_ylim(0,100)

    plt.legend(loc='best',bbox_to_anchor=[1.1,0.9])
    
    return ax
```

In [27]:

```
sns.set_style("white")
```

In [28]:

```
f, ( ax1,ax2 ) = plt.subplots( 1,2,figsize=(16,6) );
makeConfPlot(bigDf['f'].unstack(1),ax1);
makeConfPlot(bigDf['m'].unstack(1),ax2);
sns.despine()
plt.savefig('../figures/confusionPlot.png',dpi=300)
```

```
/opt/anaconda2/lib/python2.7/site-packages/matplotlib/axes/_axes.py:519: UserWarning: No labelled objects found. Use label='...' kwarg on individual plots.
  warnings.warn("No labelled objects found. "
```

## Get only the correct responses¶

In [29]:

```
def makeDiagonal(df):
    diagDf = pd.DataFrame()

    for ident in df.columns.levels[0]:
        for real in df.columns.levels[1]:
            for resp in df.columns.levels[2]:
                if real == resp:
                    thisCond = pd.DataFrame(df[ident][real][resp])
                    thisCond.columns=pd.MultiIndex.from_tuples( [(ident,resp)] )
                    diagDf = pd.concat([diagDf,thisCond],axis=1)
    return  diagDf
```

### For the % of correct responses:¶

In [30]:

```
hitCount = makeDiagonal(stackHits)
```

In [31]:

```
hitCount.tail()
```

Out[31]:

|  | f | | | | | | | m | | | | | | |
| --- | --- | --- | --- | --- | --- | --- | --- | --- | --- | --- | --- | --- | --- | --- |
|  | ang | dis | fea | hap | ntr | sad | sup | ang | dis | fea | hap | ntr | sad | sup |
| p092 | 87.50 | 56.25 | 37.50 | 93.75 | 68.75 | 12.50 | 81.25 | 43.75 | 75.00 | 50.00 | 93.75 | 68.75 | 0.00 | 43.75 |
| p093 | 93.75 | 62.50 | 56.25 | 93.75 | 87.50 | 81.25 | 75.00 | 56.25 | 100.00 | 87.50 | 100.00 | 62.50 | 12.50 | 68.75 |
| p094 | 87.50 | 75.00 | 31.25 | 100.00 | 93.75 | 18.75 | 93.75 | 31.25 | 81.25 | 31.25 | 100.00 | 87.50 | 18.75 | 100.00 |
| p095 | 100.00 | 87.50 | 31.25 | 100.00 | 93.75 | 62.50 | 100.00 | 43.75 | 75.00 | 81.25 | 100.00 | 81.25 | 18.75 | 93.75 |
| p096 | 62.50 | 68.75 | 31.25 | 93.75 | 93.75 | 25.00 | 87.50 | 56.25 | 50.00 | 62.50 | 100.00 | 31.25 | 31.25 | 87.50 |

### For the number of revealed tiles:¶

In [32]:

```
hitMasks = makeDiagonal(stackMask)
```

In [33]:

```
hitMasks.tail()
```

Out[33]:

|  | f | | | | | | | m | | | | | | |
| --- | --- | --- | --- | --- | --- | --- | --- | --- | --- | --- | --- | --- | --- | --- |
|  | ang | dis | fea | hap | ntr | sad | sup | ang | dis | fea | hap | ntr | sad | sup |
| p092 | 16.666667 | 20.370370 | 25.000000 | 18.472222 | 23.674242 | 33.333333 | 31.410256 | 26.488095 | 18.229167 | 25.260417 | 17.777778 | 23.106061 | NaN | 35.714286 |
| p093 | 20.000000 | 18.958333 | 32.407407 | 12.916667 | 18.750000 | 21.955128 | 19.097222 | 25.000000 | 21.354167 | 28.720238 | 10.807292 | 21.041667 | 20.833333 | 28.787879 |
| p094 | 20.982143 | 20.138889 | 44.166667 | 17.317708 | 30.833333 | 43.750000 | 24.444444 | 33.750000 | 20.512821 | 30.833333 | 20.963542 | 29.166667 | 36.805556 | 19.401042 |
| p095 | 21.614583 | 19.940476 | 40.000000 | 12.760417 | 37.361111 | 31.875000 | 18.750000 | 32.142857 | 19.618056 | 43.269231 | 12.890625 | 34.935897 | 20.833333 | 26.666667 |
| p096 | 14.791667 | 16.856061 | 31.250000 | 18.333333 | 31.666667 | 23.437500 | 21.279762 | 25.925926 | 15.104167 | 25.416667 | 21.354167 | 37.916667 | 30.416667 | 24.107143 |

## Plotting the relationship of hits and speed¶

Restructuring again

In [34]:

```
bigCount = hitMasks.stack(0).unstack(1)
bigHits = hitCount.stack(0).unstack(1)
```

How to plot:

In [35]:

```
def makeMyScatter(bigCount,bigHits,stackColors=stackColors):
    
    # order of face expressions is defined by hand here, instead of
    # using the columns, so the order is as we wish it to be
    for e,emo in enumerate(['hap','ang','sup','ntr','dis','fea','sad']):
        thisColor = stackColors[emoReverse[emo]]
        thisHex = rgb2hex( (thisColor[0]*255,thisColor[1]*255,thisColor[2]*255) )
        
        for i,ident in enumerate(bigCount.mean().index.levels[1]):

            assert bigCount.shape[0] == bigHits.shape[0], 'number of participants in both metric dicts not equal!'
            n = bigCount.shape[0]

            countMean = bigCount.mean()[emo][ident]
            countCI = bigCount.std(ddof=1)[emo][ident]/np.sqrt(n)*1.96

            hitMean = bigHits.mean()[emo][ident]
            hitCI = bigHits.std(ddof=1)[emo][ident]/np.sqrt(n)*1.96

            if i == 0:
                myFmt = 'o'
            elif i == 1:
                myFmt = 'v'

            plt.errorbar(countMean,
                         hitMean,
                         xerr=countCI,
                         yerr=hitCI,
                         fmt=myFmt,
                         c=thisColor,
                         ecolor='gray',
                         label= str(ident)+'-'+str(emo),
                         markersize=15,
                         markeredgecolor='gray',
                         markeredgewidth=.5
                        )
    sns.despine()
    plt.xlabel('% Tiles Revealed')
    plt.ylabel('% Correct Responses')

    plt.legend(loc='right', bbox_to_anchor=(1.2,.5));
    
    plt.savefig('../figures/globalScatter.png',dpi=300)
    plt.show()
```

In [36]:

```
fig = plt.figure(figsize=(10,7))
makeMyScatter(bigCount,bigHits)
```

## Violin Plots for correct responses¶

In [37]:

```
def scriptToGetViolinPlot(df,myYscale):
    # restructure so that the the cases move to the index and there is only one column left
    violinDf = pd.DataFrame( df.stack(0).stack(0) )

    violinDf['g'] = violinDf.index.labels[2]
    violinDf['e'] = violinDf.index.labels[1]

    # change the emoname-number mapping
    emoList = list(violinDf['e'])
    for i,entry in enumerate(emoList):
        thisName = violinDf.index.levels[1][entry]
        thisNumber = myLabels.values().index(thisName)
        emoList[i] = thisNumber
    violinDf['e'] = emoList
    # change the order to be like the new mapping
    violinDf = violinDf.sort_values(by='g')
    violinDf = violinDf.sort_values(by='e')
    fig = plt.figure(figsize=(14,6))

    sns.violinplot(x="e",
                      y=0,
                      hue="g",
                      data=violinDf,
                      split=True,
                      inner=None)

    sns.stripplot(x="e",
                      y=0,
                      hue="g",
                      data=violinDf,
                      split=True,
                      jitter=True,
                      alpha=1.,
                      linewidth=.5,
                      edgecolor='white')

    plt.xticks(range(7),myLabels.values())
    plt.ylim(0,myYscale)
    sns.despine()
    plt.show()
```

### % Hits¶

The blue parts of the plot are the female face and the green part are the male face.

In [38]:

```
scriptToGetViolinPlot(bigHits,105)
```

### % Tiles Revealed¶

In [39]:

```
scriptToGetViolinPlot(bigCount,myYscale=70)
```

## Inferential Statistics¶

Make mean values (averaged over face gender)

In [40]:

```
def makeAverage(df,myLabels=myLabels):
    
    meanDf = pd.DataFrame()
    
    for index,entry in enumerate( df.columns.levels[0] ):
        thisDf = pd.DataFrame( df[ entry ].T.mean() )
        thisDf.columns = [myLabels[index]]
        meanDf = pd.concat([meanDf, thisDf],axis=1)
        
    return meanDf
```

In [41]:

```
meanHits = makeAverage(bigHits)
```

In [42]:

```
meanCount = makeAverage(bigCount)
```

In [43]:

```
from scipy import stats
```

In [44]:

```
def ttestTable(df):

    d = {}

    for entry1 in df.columns:
        firstSample = df[entry1]
        for entry2 in df.columns:
            if entry1 != entry2:
                secondSample = df[entry2]
                thisT,thisP =  stats.ttest_rel(firstSample,secondSample)

                newName = [entry1,entry2]
                newName.sort()

                d[newName[0]+'-'+newName[1]] = {'t':round(thisT,2),'p':round(thisP,5)}
    outDf = pd.DataFrame(d).T
    
    return outDf
```

In [45]:

```
meanHits['sad'][:5]
```

Out[45]:

```
p001    93.750
p002    46.875
p003    53.125
p004    87.500
p005    90.625
Name: sad, dtype: float64
```

In [46]:

```
ttestTable(meanHits)
```

Out[46]:

|  | p | t |
| --- | --- | --- |
| ang-dis | 0.00000 | 8.05 |
| ang-fea | 0.00000 | 19.83 |
| ang-hap | 0.00000 | -7.83 |
| ang-ntr | 0.00000 | 9.59 |
| ang-sad | 0.00000 | -11.38 |
| ang-sup | 0.00000 | -7.76 |
| dis-fea | 0.00000 | -13.33 |
| dis-hap | 0.06618 | 1.86 |
| dis-ntr | 0.00980 | 2.64 |
| dis-sad | 0.95641 | -0.05 |
| dis-sup | 0.00000 | -16.10 |
| fea-hap | 0.00000 | 18.53 |
| fea-ntr | 0.00000 | -12.47 |
| fea-sad | 0.00000 | 17.00 |
| fea-sup | 0.00000 | -30.59 |
| hap-ntr | 0.00002 | 4.49 |
| hap-sad | 0.02760 | 2.24 |
| hap-sup | 0.00000 | -16.33 |
| ntr-sad | 0.01629 | 2.45 |
| ntr-sup | 0.00000 | 18.37 |
| sad-sup | 0.00000 | -18.10 |

In [47]:

```
ttestTable(meanCount)
```

Out[47]:

|  | p | t |
| --- | --- | --- |
| ang-dis | 0.38042 | -0.88 |
| ang-fea | 0.00000 | -16.53 |
| ang-hap | 0.00000 | 11.27 |
| ang-ntr | 0.00000 | -7.89 |
| ang-sad | 0.00000 | 15.76 |
| ang-sup | NaN | NaN |
| dis-fea | 0.00000 | 21.30 |
| dis-hap | 0.00000 | 11.19 |
| dis-ntr | 0.00000 | -9.28 |
| dis-sad | 0.00000 | 17.73 |
| dis-sup | NaN | NaN |
| fea-hap | 0.00000 | -11.56 |
| fea-ntr | 0.00000 | 14.65 |
| fea-sad | 0.00023 | -3.83 |
| fea-sup | NaN | NaN |
| hap-ntr | 0.00038 | 3.69 |
| hap-sad | 0.00000 | -8.54 |
| hap-sup | NaN | NaN |
| ntr-sad | 0.00000 | 10.38 |
| ntr-sup | NaN | NaN |
| sad-sup | NaN | NaN |

In [48]:

```
ttestTable(meanCount.fillna(0))
```

Out[48]:

|  | p | t |
| --- | --- | --- |
| ang-dis | 0.38042 | -0.88 |
| ang-fea | 0.00000 | -16.53 |
| ang-hap | 0.00000 | 11.27 |
| ang-ntr | 0.00000 | -7.89 |
| ang-sad | 0.00000 | 15.76 |
| ang-sup | 0.26350 | -1.12 |
| dis-fea | 0.00000 | 21.30 |
| dis-hap | 0.00000 | 11.19 |
| dis-ntr | 0.00000 | -9.28 |
| dis-sad | 0.00000 | 17.73 |
| dis-sup | 0.58878 | -0.54 |
| fea-hap | 0.00000 | -11.56 |
| fea-ntr | 0.00000 | 14.65 |
| fea-sad | 0.00023 | -3.83 |
| fea-sup | 0.00000 | 12.93 |
| hap-ntr | 0.00038 | 3.69 |
| hap-sad | 0.00000 | -8.54 |
| hap-sup | 0.00000 | 7.70 |
| ntr-sad | 0.00000 | 10.38 |
| ntr-sup | 0.00000 | -5.43 |
| sad-sup | 0.00000 | 11.45 |
