# Supplementary material for: Mapping the emotional face. How individual face parts contribute to successful emotion recognition
Source: PLoS One. 2017 May 11;12(5):e0177239. doi: 10.1371/journal.pone.0177239 (PMC5426715; doi:10.1371/journal.pone.0177239)
Supplement: S4 Code — (HTML) [file pone.0177239.s006.html]

code003\_weighting


# Mapping the emotional face. How individual face parts contribute to successful emotion recognition.

# 3. Applying Weights¶

Here, a weight factor is computed for each tile in each trial. This is the basic metric which serves as input to all the following notebooks.

In [1]:

```
from myBasics import *

%matplotlib inline
```

In [2]:

```
logList = getFile('../rawTables/','pand*.csv')
```

### The formula:¶

Here, each tile gets a value assigned. This works according to the following formula:

$$weight = \frac{1}{n}\sum\_{i}^{n}({1-\frac{list\ lenght}{max\ length}})$$

This means that a weight is assigned to each tile belonging to trial i.
The weigth is computed by:

- taking the lenght of the trial and dividing it by the maximum possible length;  
  this gives small numbers for long trials and large numbers for short trials
- since we want to have a higher weight for short trials we do 1 minus this term
- since very long trials are non-diagnostic, we only do this for trials that contain 16 revealed tiles or less. This number was derived by taking the 75th percentile of all trial length. Using this cutoff makes our formula much more sensitive, because if we divided the list length by 48 (maximal possible number of tiles revealed), it would make less of a difference e.g. if a trial had length 4 or 8 (4/48 and 8/48 gives only a difference of 1/12) then if we scale by the more stringent cutoff (where a difference of 4 tiles translates into a weight difference of 1/4th).

What results is a weight factor for each tile, which is conditional on the length of the trial the tile was part of. If a tile is often part of short trials, we assume that it is more diagnostic. The most extreme case would be if the tile alone can give rise to a correct answer.

We have 16 trials per face, but since we use a cutoff for the maximal allowed trial length, there can be fewer.  
The lowest number of zero would be achieved, if a tile was never part of any trial.
The maximal number of one can actually not be achieved, because it would mean that a participant always gives a correct answer without having seen any tile revealed (1-0 for all trials). Even if that would happen, there would be no tile that could get that weight assigned.
In theory, a tile could be always the first one revealed and always sufficient by itself to trigger a correct response. Then its weight would be 1-1/16 = 15/16. This is in so far very unrealistic, as the chance to be the first one revealed is 1/48 in the first place.

Therefore, given the amount of tiles and the low probabilities associated, very low weight numbers are to be expected.

### get distribution of trial lengths¶

Here, we take all trial lengths (= number of revealed tiles) form all participants, put them in one big list and then inspect its distribution to decide on a cutoff value. We come to the conclusion that a cutoff of 16 is reasonable.

In [3]:

```
allMasks = []
for logFile in logList:
    thisDf = pd.read_csv(logFile,header=0,index_col=[0,1,2])
    thisMasks = list( thisDf['maskNum'] )
    allMasks = allMasks+thisMasks
```

In [4]:

```
sns.distplot( allMasks );
sns.despine()
print '75% percentile:', np.percentile(allMasks, 75+1)
```

```
75% percentile: 16.0
```

In [5]:

```
def getWeight(df,thisName,ident,express,n=16,identDict=identDict,emoDict=emoDict):
    
    d = {}
    
    # get the current condition (face identity and emotion expression)
    thisDf = pd.DataFrame(df.ix[ident].ix[express])

    # loop through all trials of that condition
    for i in thisDf.index:
        # we only consider correct trials, so on incorrect trials,
        # we don't do nothing
        if thisDf.ix[i]['evaluation'] != 'HIT':
            pass
            
        # if the a correct answer was given in the current trial
        else:
            # we get the list of revealed tiles
            maskList = thisDf.ix[i]['maskList']
            thisSplit = maskList.split('-')
            # if the current trial is short enough
            if len(thisSplit) <= n:
                    
                # we apply the above formula to get a weight for the importance of that trial
                    
                ####
                thisFormula = 1. - (len(thisSplit)/float(n) ) 
                ####
                    
                # now each tile of that trial gets that weight assigned, because it was
                # part of this trial
                for num in thisSplit:
                    # we try to append, if this does not work, we create the entry
                    try:
                        d[num].append(thisFormula)
                    except:
                        d[num] = [thisFormula]
                            
                # everyone else not present gets a zero
                # this works by having a list of all possible tile indices (0-47)
                # and removing indeces of all trial that were actually present
                unassigned = range(48)
                for num in thisSplit: unassigned.remove( int(num) )
                # now each tile that was not in the trial gets a zero for having been absent
                for num in unassigned:
                    try:
                        d[str(num)].append(0)
                    except:
                        d[str(num)] = [0]
    
    # note that if a trial contained an incorrect answer or was not short enough, it is
    # entierly ignored. This might not be desireable, however. E.g. one might argue that
    # if a tile is often part of an incorrect trial, it should get a negative weight
    # so there are certainly many possibilities to make the formula more sophisticated
    
    #average over all trials for each tile 
    outDf = pd.DataFrame( pd.DataFrame( d ).mean() )
    # make the index nice and restructure
    outDf.index = [int(x) for x in outDf.index]
    outDf = outDf.sort_index()
    outDf = outDf.T
    outDf.index = [[thisName],[identDict[ident]],[emoDict[express]]]
    outDf = outDf.sortlevel()
    # return a row with all values of the current expression
    return outDf
```

Example with one face of one participant:

In [6]:

```
df = pd.read_csv(logList[-1],
                 header=0,
                 index_col=[0,1,2]
                )
```

In [7]:

```
getWeight(df,'test',0,0)
```

Out[7]:

|  |  |  | 0 | 1 | 2 | 3 | 4 | 5 | 6 | 7 | 8 | 9 | 10 | 11 | 12 | 13 | 14 | 15 | 16 | 17 | 18 | 19 | 20 | 21 | 22 | 23 | 24 | 25 | 26 | 27 | 28 | 29 | 30 | 31 | 32 | 33 | 34 | 35 | 36 | 37 | 38 | 39 | 40 | 41 | 42 | 43 | 44 | 45 | 46 | 47 |
| --- | --- | --- | --- | --- | --- | --- | --- | --- | --- | --- | --- | --- | --- | --- | --- | --- | --- | --- | --- | --- | --- | --- | --- | --- | --- | --- | --- | --- | --- | --- | --- | --- | --- | --- | --- | --- | --- | --- | --- | --- | --- | --- | --- | --- | --- | --- | --- | --- | --- | --- |
| test | f | hap | 0.0 | 0.0 | 0.052083 | 0.25 | 0.041667 | 0.0 | 0.0 | 0.015625 | 0.0625 | 0.0 | 0.015625 | 0.052083 | 0.0625 | 0.171875 | 0.0 | 0.057292 | 0.0625 | 0.098958 | 0.015625 | 0.052083 | 0.114583 | 0.067708 | 0.067708 | 0.057292 | 0.015625 | 0.0 | 0.041667 | 0.052083 | 0.067708 | 0.265625 | 0.015625 | 0.052083 | 0.0 | 0.010417 | 0.130208 | 0.0625 | 0.119792 | 0.166667 | 0.057292 | 0.161458 | 0.010417 | 0.046875 | 0.0 | 0.078125 | 0.130208 | 0.119792 | 0.046875 | 0.09375 |

### get all faces of one participant¶

In [8]:

```
def getParticipantWeights(logfile):
    
    # read the contents of the logfile into a dataframe
    df = pd.read_csv(logfile,
                     header=0,
                     index_col=[0,1,2])
    
    # get the naming of the current participant right
    thisName = 'p'+logfile[logfile.rfind('e')+1:logfile.rfind('.')]
    
    # make a df with all faces for that participant
    bigWeight = pd.DataFrame()
    for ident in range(2):
        for emo in  range(7):
            thisWeight = getWeight(df,thisName,ident,emo)
            bigWeight = pd.concat([bigWeight,thisWeight])
    bigWeight = bigWeight.sortlevel()
    return bigWeight
```

Example:

In [9]:

```
getParticipantWeights(logList[-1])
```

Out[9]:

|  |  |  | 0 | 1 | 2 | 3 | 4 | 5 | 6 | 7 | 8 | 9 | 10 | 11 | 12 | 13 | 14 | 15 | 16 | 17 | 18 | 19 | 20 | 21 | 22 | 23 | 24 | 25 | 26 | 27 | 28 | 29 | 30 | 31 | 32 | 33 | 34 | 35 | 36 | 37 | 38 | 39 | 40 | 41 | 42 | 43 | 44 | 45 | 46 | 47 |
| --- | --- | --- | --- | --- | --- | --- | --- | --- | --- | --- | --- | --- | --- | --- | --- | --- | --- | --- | --- | --- | --- | --- | --- | --- | --- | --- | --- | --- | --- | --- | --- | --- | --- | --- | --- | --- | --- | --- | --- | --- | --- | --- | --- | --- | --- | --- | --- | --- | --- | --- |
| p096 | f | ang | 0.000000 | 0.150000 | 0.087500 | 0.056250 | 0.056250 | 0.137500 | 0.093750 | 0.056250 | 0.087500 | 0.050000 | 0.000000 | 0.000000 | 0.106250 | 0.137500 | 0.043750 | 0.093750 | 0.168750 | 0.093750 | 0.131250 | 0.000000 | 0.050000 | 0.075000 | 0.056250 | 0.175000 | 0.000000 | 0.056250 | 0.000000 | 0.087500 | 0.150000 | 0.081250 | 0.062500 | 0.081250 | 0.050000 | 0.037500 | 0.050000 | 0.093750 | 0.156250 | 0.131250 | 0.043750 | 0.106250 | 0.000000 | 0.106250 | 0.143750 | 0.000000 | 0.081250 | 0.137500 | 0.106250 | 0.000000 |
|  |  | dis | 0.051136 | 0.045455 | 0.045455 | 0.204545 | 0.130682 | 0.142045 | 0.000000 | 0.107955 | 0.039773 | 0.056818 | 0.068182 | 0.096591 | 0.022727 | 0.045455 | 0.005682 | 0.079545 | 0.062500 | 0.085227 | 0.068182 | 0.068182 | 0.062500 | 0.102273 | 0.113636 | 0.000000 | 0.136364 | 0.062500 | 0.045455 | 0.034091 | 0.005682 | 0.130682 | 0.107955 | 0.119318 | 0.073864 | 0.034091 | 0.073864 | 0.051136 | 0.113636 | 0.142045 | 0.170455 | 0.022727 | 0.000000 | 0.034091 | 0.005682 | 0.000000 | 0.068182 | 0.147727 | 0.022727 | 0.039773 |
|  |  | fea | 0.078125 | 0.000000 | 0.000000 | 0.031250 | 0.078125 | 0.000000 | 0.000000 | 0.078125 | 0.046875 | 0.031250 | 0.000000 | 0.046875 | 0.000000 | 0.031250 | 0.000000 | 0.046875 | 0.062500 | 0.000000 | 0.031250 | 0.062500 | 0.046875 | 0.000000 | 0.000000 | 0.031250 | 0.031250 | 0.031250 | 0.031250 | 0.000000 | 0.031250 | 0.000000 | 0.000000 | 0.078125 | 0.109375 | 0.031250 | 0.109375 | 0.031250 | 0.031250 | 0.000000 | 0.000000 | 0.000000 | 0.046875 | 0.000000 | 0.078125 | 0.031250 | 0.078125 | 0.000000 | 0.031250 | 0.000000 |
|  |  | hap | 0.000000 | 0.000000 | 0.052083 | 0.250000 | 0.041667 | 0.000000 | 0.000000 | 0.015625 | 0.062500 | 0.000000 | 0.015625 | 0.052083 | 0.062500 | 0.171875 | 0.000000 | 0.057292 | 0.062500 | 0.098958 | 0.015625 | 0.052083 | 0.114583 | 0.067708 | 0.067708 | 0.057292 | 0.015625 | 0.000000 | 0.041667 | 0.052083 | 0.067708 | 0.265625 | 0.015625 | 0.052083 | 0.000000 | 0.010417 | 0.130208 | 0.062500 | 0.119792 | 0.166667 | 0.057292 | 0.161458 | 0.010417 | 0.046875 | 0.000000 | 0.078125 | 0.130208 | 0.119792 | 0.046875 | 0.093750 |
|  |  | ntr | 0.085938 | 0.054688 | 0.054688 | 0.054688 | 0.031250 | 0.093750 | 0.054688 | 0.171875 | 0.109375 | 0.046875 | 0.164062 | 0.000000 | 0.062500 | 0.062500 | 0.078125 | 0.000000 | 0.070312 | 0.031250 | 0.039062 | 0.078125 | 0.125000 | 0.187500 | 0.062500 | 0.054688 | 0.070312 | 0.070312 | 0.031250 | 0.187500 | 0.187500 | 0.039062 | 0.000000 | 0.070312 | 0.109375 | 0.039062 | 0.070312 | 0.000000 | 0.062500 | 0.000000 | 0.000000 | 0.031250 | 0.000000 | 0.085938 | 0.117188 | 0.210938 | 0.085938 | 0.031250 | 0.109375 | 0.054688 |
|  |  | sad | 0.062500 | 0.125000 | 0.171875 | 0.000000 | 0.000000 | 0.000000 | 0.000000 | 0.000000 | 0.000000 | 0.000000 | 0.109375 | 0.125000 | 0.062500 | 0.062500 | 0.000000 | 0.109375 | 0.000000 | 0.234375 | 0.000000 | 0.187500 | 0.062500 | 0.000000 | 0.000000 | 0.109375 | 0.000000 | 0.234375 | 0.109375 | 0.062500 | 0.000000 | 0.234375 | 0.000000 | 0.000000 | 0.187500 | 0.062500 | 0.062500 | 0.000000 | 0.000000 | 0.000000 | 0.109375 | 0.000000 | 0.000000 | 0.062500 | 0.187500 | 0.000000 | 0.000000 | 0.000000 | 0.000000 | 0.000000 |
|  |  | sup | 0.039773 | 0.119318 | 0.096591 | 0.062500 | 0.056818 | 0.000000 | 0.250000 | 0.000000 | 0.096591 | 0.000000 | 0.079545 | 0.028409 | 0.000000 | 0.102273 | 0.028409 | 0.079545 | 0.000000 | 0.028409 | 0.039773 | 0.113636 | 0.085227 | 0.119318 | 0.085227 | 0.079545 | 0.056818 | 0.000000 | 0.000000 | 0.068182 | 0.039773 | 0.187500 | 0.221591 | 0.000000 | 0.039773 | 0.000000 | 0.039773 | 0.096591 | 0.125000 | 0.000000 | 0.102273 | 0.056818 | 0.227273 | 0.000000 | 0.068182 | 0.102273 | 0.000000 | 0.000000 | 0.028409 | 0.056818 |
|  | m | ang | 0.104167 | 0.000000 | 0.000000 | 0.000000 | 0.052083 | 0.000000 | 0.000000 | 0.000000 | 0.125000 | 0.000000 | 0.104167 | 0.000000 | 0.072917 | 0.125000 | 0.052083 | 0.000000 | 0.000000 | 0.104167 | 0.125000 | 0.000000 | 0.000000 | 0.312500 | 0.052083 | 0.104167 | 0.052083 | 0.000000 | 0.000000 | 0.052083 | 0.072917 | 0.000000 | 0.177083 | 0.000000 | 0.000000 | 0.000000 | 0.177083 | 0.000000 | 0.000000 | 0.000000 | 0.125000 | 0.135417 | 0.000000 | 0.333333 | 0.000000 | 0.125000 | 0.125000 | 0.000000 | 0.052083 | 0.000000 |
|  |  | dis | 0.000000 | 0.156250 | 0.132812 | 0.054688 | 0.148438 | 0.156250 | 0.148438 | 0.078125 | 0.000000 | 0.226562 | 0.125000 | 0.078125 | 0.015625 | 0.000000 | 0.148438 | 0.093750 | 0.140625 | 0.054688 | 0.179688 | 0.000000 | 0.000000 | 0.093750 | 0.078125 | 0.054688 | 0.015625 | 0.000000 | 0.156250 | 0.101562 | 0.015625 | 0.156250 | 0.078125 | 0.101562 | 0.000000 | 0.000000 | 0.000000 | 0.054688 | 0.054688 | 0.000000 | 0.085938 | 0.000000 | 0.015625 | 0.156250 | 0.070312 | 0.000000 | 0.000000 | 0.000000 | 0.164062 | 0.015625 |
|  |  | fea | 0.000000 | 0.093750 | 0.093750 | 0.177083 | 0.083333 | 0.177083 | 0.114583 | 0.072917 | 0.000000 | 0.000000 | 0.187500 | 0.083333 | 0.104167 | 0.145833 | 0.208333 | 0.000000 | 0.072917 | 0.000000 | 0.083333 | 0.083333 | 0.000000 | 0.083333 | 0.187500 | 0.000000 | 0.083333 | 0.000000 | 0.187500 | 0.083333 | 0.000000 | 0.093750 | 0.072917 | 0.072917 | 0.072917 | 0.072917 | 0.250000 | 0.093750 | 0.072917 | 0.000000 | 0.104167 | 0.093750 | 0.093750 | 0.000000 | 0.177083 | 0.000000 | 0.072917 | 0.083333 | 0.000000 | 0.000000 |
|  |  | hap | 0.062500 | 0.066964 | 0.075893 | 0.093750 | 0.035714 | 0.026786 | 0.026786 | 0.026786 | 0.000000 | 0.053571 | 0.129464 | 0.071429 | 0.098214 | 0.022321 | 0.000000 | 0.102679 | 0.000000 | 0.062500 | 0.116071 | 0.026786 | 0.116071 | 0.107143 | 0.022321 | 0.026786 | 0.004464 | 0.142857 | 0.044643 | 0.098214 | 0.031250 | 0.129464 | 0.008929 | 0.058036 | 0.080357 | 0.017857 | 0.089286 | 0.044643 | 0.062500 | 0.263393 | 0.053571 | 0.040179 | 0.049107 | 0.049107 | 0.000000 | 0.035714 | 0.053571 | 0.093750 | 0.066964 | 0.035714 |
|  |  | ntr | 0.312500 | 0.000000 | 0.000000 | 0.000000 | 0.000000 | 0.000000 | 0.000000 | 0.281250 | 0.000000 | 0.312500 | 0.281250 | 0.000000 | 0.281250 | 0.593750 | 0.000000 | 0.000000 | 0.000000 | 0.000000 | 0.000000 | 0.000000 | 0.000000 | 0.000000 | 0.000000 | 0.000000 | 0.000000 | 0.000000 | 0.000000 | 0.000000 | 0.000000 | 0.000000 | 0.312500 | 0.281250 | 0.000000 | 0.000000 | 0.000000 | 0.000000 | 0.000000 | 0.000000 | 0.000000 | 0.281250 | 0.000000 | 0.000000 | 0.281250 | 0.000000 | 0.312500 | 0.000000 | 0.312500 | 0.000000 |
|  |  | sad | 0.000000 | 0.062500 | 0.125000 | 0.000000 | 0.000000 | 0.046875 | 0.078125 | 0.046875 | 0.250000 | 0.125000 | 0.000000 | 0.140625 | 0.062500 | 0.000000 | 0.000000 | 0.000000 | 0.078125 | 0.187500 | 0.109375 | 0.062500 | 0.000000 | 0.000000 | 0.125000 | 0.000000 | 0.125000 | 0.046875 | 0.250000 | 0.000000 | 0.000000 | 0.078125 | 0.062500 | 0.140625 | 0.000000 | 0.046875 | 0.062500 | 0.000000 | 0.000000 | 0.046875 | 0.062500 | 0.078125 | 0.046875 | 0.125000 | 0.062500 | 0.187500 | 0.046875 | 0.000000 | 0.000000 | 0.000000 |
|  |  | sup | 0.022727 | 0.085227 | 0.062500 | 0.022727 | 0.000000 | 0.000000 | 0.000000 | 0.096591 | 0.068182 | 0.107955 | 0.000000 | 0.056818 | 0.159091 | 0.000000 | 0.306818 | 0.090909 | 0.119318 | 0.022727 | 0.073864 | 0.062500 | 0.181818 | 0.176136 | 0.142045 | 0.000000 | 0.113636 | 0.107955 | 0.034091 | 0.090909 | 0.102273 | 0.181818 | 0.039773 | 0.034091 | 0.096591 | 0.000000 | 0.119318 | 0.051136 | 0.068182 | 0.096591 | 0.062500 | 0.073864 | 0.045455 | 0.000000 | 0.056818 | 0.096591 | 0.000000 | 0.159091 | 0.062500 | 0.079545 |

### do a transformation to percent signal change¶

like in fMRI, we use a formula like so:

$$psc = \frac{value}{mean}\*100-100$$

In [10]:

```
def makePSC(logfile):
    
    # get the raw weights
    bigWeight = getParticipantWeights(logfile)
    
    # normalize
    byMean = bigWeight.T/bigWeight.T.mean()
    scaledDf = (( ( byMean ).T )*100) -100 # that's the PSC formula
    
    return scaledDf
```

Example:

In [11]:

```
getParticipantWeights(logList[-1]).head()
```

Out[11]:

|  |  |  | 0 | 1 | 2 | 3 | 4 | 5 | 6 | 7 | 8 | 9 | 10 | 11 | 12 | 13 | 14 | 15 | 16 | 17 | 18 | 19 | 20 | 21 | 22 | 23 | 24 | 25 | 26 | 27 | 28 | 29 | 30 | 31 | 32 | 33 | 34 | 35 | 36 | 37 | 38 | 39 | 40 | 41 | 42 | 43 | 44 | 45 | 46 | 47 |
| --- | --- | --- | --- | --- | --- | --- | --- | --- | --- | --- | --- | --- | --- | --- | --- | --- | --- | --- | --- | --- | --- | --- | --- | --- | --- | --- | --- | --- | --- | --- | --- | --- | --- | --- | --- | --- | --- | --- | --- | --- | --- | --- | --- | --- | --- | --- | --- | --- | --- | --- |
| p096 | f | ang | 0.000000 | 0.150000 | 0.087500 | 0.056250 | 0.056250 | 0.137500 | 0.093750 | 0.056250 | 0.087500 | 0.050000 | 0.000000 | 0.000000 | 0.106250 | 0.137500 | 0.043750 | 0.093750 | 0.168750 | 0.093750 | 0.131250 | 0.000000 | 0.050000 | 0.075000 | 0.056250 | 0.175000 | 0.000000 | 0.056250 | 0.000000 | 0.087500 | 0.150000 | 0.081250 | 0.062500 | 0.081250 | 0.050000 | 0.037500 | 0.050000 | 0.093750 | 0.156250 | 0.131250 | 0.043750 | 0.106250 | 0.000000 | 0.106250 | 0.143750 | 0.000000 | 0.081250 | 0.137500 | 0.106250 | 0.000000 |
|  |  | dis | 0.051136 | 0.045455 | 0.045455 | 0.204545 | 0.130682 | 0.142045 | 0.000000 | 0.107955 | 0.039773 | 0.056818 | 0.068182 | 0.096591 | 0.022727 | 0.045455 | 0.005682 | 0.079545 | 0.062500 | 0.085227 | 0.068182 | 0.068182 | 0.062500 | 0.102273 | 0.113636 | 0.000000 | 0.136364 | 0.062500 | 0.045455 | 0.034091 | 0.005682 | 0.130682 | 0.107955 | 0.119318 | 0.073864 | 0.034091 | 0.073864 | 0.051136 | 0.113636 | 0.142045 | 0.170455 | 0.022727 | 0.000000 | 0.034091 | 0.005682 | 0.000000 | 0.068182 | 0.147727 | 0.022727 | 0.039773 |
|  |  | fea | 0.078125 | 0.000000 | 0.000000 | 0.031250 | 0.078125 | 0.000000 | 0.000000 | 0.078125 | 0.046875 | 0.031250 | 0.000000 | 0.046875 | 0.000000 | 0.031250 | 0.000000 | 0.046875 | 0.062500 | 0.000000 | 0.031250 | 0.062500 | 0.046875 | 0.000000 | 0.000000 | 0.031250 | 0.031250 | 0.031250 | 0.031250 | 0.000000 | 0.031250 | 0.000000 | 0.000000 | 0.078125 | 0.109375 | 0.031250 | 0.109375 | 0.031250 | 0.031250 | 0.000000 | 0.000000 | 0.000000 | 0.046875 | 0.000000 | 0.078125 | 0.031250 | 0.078125 | 0.000000 | 0.031250 | 0.000000 |
|  |  | hap | 0.000000 | 0.000000 | 0.052083 | 0.250000 | 0.041667 | 0.000000 | 0.000000 | 0.015625 | 0.062500 | 0.000000 | 0.015625 | 0.052083 | 0.062500 | 0.171875 | 0.000000 | 0.057292 | 0.062500 | 0.098958 | 0.015625 | 0.052083 | 0.114583 | 0.067708 | 0.067708 | 0.057292 | 0.015625 | 0.000000 | 0.041667 | 0.052083 | 0.067708 | 0.265625 | 0.015625 | 0.052083 | 0.000000 | 0.010417 | 0.130208 | 0.062500 | 0.119792 | 0.166667 | 0.057292 | 0.161458 | 0.010417 | 0.046875 | 0.000000 | 0.078125 | 0.130208 | 0.119792 | 0.046875 | 0.093750 |
|  |  | ntr | 0.085938 | 0.054688 | 0.054688 | 0.054688 | 0.031250 | 0.093750 | 0.054688 | 0.171875 | 0.109375 | 0.046875 | 0.164062 | 0.000000 | 0.062500 | 0.062500 | 0.078125 | 0.000000 | 0.070312 | 0.031250 | 0.039062 | 0.078125 | 0.125000 | 0.187500 | 0.062500 | 0.054688 | 0.070312 | 0.070312 | 0.031250 | 0.187500 | 0.187500 | 0.039062 | 0.000000 | 0.070312 | 0.109375 | 0.039062 | 0.070312 | 0.000000 | 0.062500 | 0.000000 | 0.000000 | 0.031250 | 0.000000 | 0.085938 | 0.117188 | 0.210938 | 0.085938 | 0.031250 | 0.109375 | 0.054688 |

In [12]:

```
makePSC(logList[-1]).head()
```

Out[12]:

|  |  |  | 0 | 1 | 2 | 3 | 4 | 5 | 6 | 7 | 8 | 9 | 10 | 11 | 12 | 13 | 14 | 15 | 16 | 17 | 18 | 19 | 20 | 21 | 22 | 23 | 24 | 25 | 26 | 27 | 28 | 29 | 30 | 31 | 32 | 33 | 34 | 35 | 36 | 37 | 38 | 39 | 40 | 41 | 42 | 43 | 44 | 45 | 46 | 47 |
| --- | --- | --- | --- | --- | --- | --- | --- | --- | --- | --- | --- | --- | --- | --- | --- | --- | --- | --- | --- | --- | --- | --- | --- | --- | --- | --- | --- | --- | --- | --- | --- | --- | --- | --- | --- | --- | --- | --- | --- | --- | --- | --- | --- | --- | --- | --- | --- | --- | --- | --- |
| p096 | f | ang | -100.000000 | 96.252129 | 14.480409 | -26.405451 | -26.405451 | 79.897785 | 22.657581 | -26.405451 | 14.480409 | -34.582624 | -100.000000 | -100.000000 | 39.011925 | 79.897785 | -42.759796 | 22.657581 | 120.783646 | 22.657581 | 71.720613 | -100.000000 | -34.582624 | -1.873935 | -26.405451 | 128.960818 | -100.000000 | -26.405451 | -100.000000 | 14.480409 | 96.252129 | 6.303237 | -18.228279 | 6.303237 | -34.582624 | -50.936968 | -34.582624 | 22.657581 | 104.429302 | 71.720613 | -42.759796 | 39.011925 | -100.000000 | 39.011925 | 88.074957 | -100.000000 | 6.303237 | 79.897785 | 39.011925 | -100.000000 |
|  |  | dis | -26.655348 | -34.804754 | -34.804754 | 193.378608 | 87.436333 | 103.735144 | -100.000000 | 54.838710 | -42.954160 | -18.505942 | -2.207131 | 38.539898 | -67.402377 | -34.804754 | -91.850594 | 14.091681 | -10.356537 | 22.241087 | -2.207131 | -2.207131 | -10.356537 | 46.689304 | 62.988115 | -100.000000 | 95.585739 | -10.356537 | -34.804754 | -51.103565 | -91.850594 | 87.436333 | 54.838710 | 71.137521 | 5.942275 | -51.103565 | 5.942275 | -26.655348 | 62.988115 | 103.735144 | 144.482173 | -67.402377 | -100.000000 | -51.103565 | -91.850594 | -100.000000 | -2.207131 | 111.884550 | -67.402377 | -42.954160 |
|  |  | fea | 152.631579 | -100.000000 | -100.000000 | 1.052632 | 152.631579 | -100.000000 | -100.000000 | 152.631579 | 51.578947 | 1.052632 | -100.000000 | 51.578947 | -100.000000 | 1.052632 | -100.000000 | 51.578947 | 102.105263 | -100.000000 | 1.052632 | 102.105263 | 51.578947 | -100.000000 | -100.000000 | 1.052632 | 1.052632 | 1.052632 | 1.052632 | -100.000000 | 1.052632 | -100.000000 | -100.000000 | 152.631579 | 253.684211 | 1.052632 | 253.684211 | 1.052632 | 1.052632 | -100.000000 | -100.000000 | -100.000000 | 51.578947 | -100.000000 | 152.631579 | 1.052632 | 152.631579 | -100.000000 | 1.052632 | -100.000000 |
|  |  | hap | -100.000000 | -100.000000 | -18.367347 | 291.836735 | -34.693878 | -100.000000 | -100.000000 | -75.510204 | -2.040816 | -100.000000 | -75.510204 | -18.367347 | -2.040816 | 169.387755 | -100.000000 | -10.204082 | -2.040816 | 55.102041 | -75.510204 | -18.367347 | 79.591837 | 6.122449 | 6.122449 | -10.204082 | -75.510204 | -100.000000 | -34.693878 | -18.367347 | 6.122449 | 316.326531 | -75.510204 | -18.367347 | -100.000000 | -83.673469 | 104.081633 | -2.040816 | 87.755102 | 161.224490 | -10.204082 | 153.061224 | -83.673469 | -26.530612 | -100.000000 | 22.448980 | 104.081633 | 87.755102 | -26.530612 | 46.938776 |
|  |  | ntr | 20.000000 | -23.636364 | -23.636364 | -23.636364 | -56.363636 | 30.909091 | -23.636364 | 140.000000 | 52.727273 | -34.545455 | 129.090909 | -100.000000 | -12.727273 | -12.727273 | 9.090909 | -100.000000 | -1.818182 | -56.363636 | -45.454545 | 9.090909 | 74.545455 | 161.818182 | -12.727273 | -23.636364 | -1.818182 | -1.818182 | -56.363636 | 161.818182 | 161.818182 | -45.454545 | -100.000000 | -1.818182 | 52.727273 | -45.454545 | -1.818182 | -100.000000 | -12.727273 | -100.000000 | -100.000000 | -56.363636 | -100.000000 | 20.000000 | 63.636364 | 194.545455 | 20.000000 | -56.363636 | 52.727273 | -23.636364 |

### Do this for the whole sample¶

In [13]:

```
def getAllWeights(logList,func=makePSC):
    bigBig = pd.DataFrame()
    for logFile in logList:
        thisDf = func(logFile)
        bigBig = pd.concat([bigBig,thisDf],axis=0)
    bigBig = bigBig.sortlevel()
    return bigBig
```

In [14]:

```
bigWeightDf = getAllWeights(logList)
```

In [15]:

```
bigWeightDf.head()
```

Out[15]:

|  |  |  | 0 | 1 | 2 | 3 | 4 | 5 | 6 | 7 | 8 | 9 | 10 | 11 | 12 | 13 | 14 | 15 | 16 | 17 | 18 | 19 | 20 | 21 | 22 | 23 | 24 | 25 | 26 | 27 | 28 | 29 | 30 | 31 | 32 | 33 | 34 | 35 | 36 | 37 | 38 | 39 | 40 | 41 | 42 | 43 | 44 | 45 | 46 | 47 |
| --- | --- | --- | --- | --- | --- | --- | --- | --- | --- | --- | --- | --- | --- | --- | --- | --- | --- | --- | --- | --- | --- | --- | --- | --- | --- | --- | --- | --- | --- | --- | --- | --- | --- | --- | --- | --- | --- | --- | --- | --- | --- | --- | --- | --- | --- | --- | --- | --- | --- | --- |
| p001 | f | ang | -75.129534 | -0.518135 | 132.124352 | -100.000000 | -100.000000 | -25.388601 | -75.129534 | 32.642487 | -100.000000 | -75.129534 | 82.383420 | 115.544041 | -100.000000 | 7.772021 | 74.093264 | -100.000000 | -75.129534 | 32.642487 | -100.000000 | 32.642487 | 82.383420 | -100.000000 | -0.518135 | -25.388601 | -25.388601 | -66.839378 | 190.155440 | 32.642487 | 107.253886 | 32.642487 | 115.544041 | -0.518135 | -100.000000 | -100.000000 | -100.000000 | 32.642487 | 82.383420 | 7.772021 | -25.388601 | -25.388601 | 7.772021 | 7.772021 | 7.772021 | 107.253886 | -100.000000 | 82.383420 | 107.253886 | 82.383420 |
|  |  | dis | -22.330097 | 94.174757 | -6.796117 | -100.000000 | -100.000000 | 39.805825 | 140.776699 | 78.640777 | -22.330097 | -100.000000 | 101.941748 | -100.000000 | 78.640777 | 70.873786 | 55.339806 | -100.000000 | 55.339806 | -53.398058 | -6.796117 | -100.000000 | -100.000000 | 164.077670 | 171.844660 | 39.805825 | -61.165049 | 32.038835 | 8.737864 | -100.000000 | -6.796117 | -100.000000 | 296.116505 | -53.398058 | 47.572816 | -6.796117 | 117.475728 | 32.038835 | -100.000000 | -100.000000 | 133.009709 | -6.796117 | -100.000000 | -53.398058 | -6.796117 | -6.796117 | -53.398058 | -53.398058 | -100.000000 | -37.864078 |
|  |  | fea | 55.956679 | -100.000000 | -100.000000 | -100.000000 | 38.628159 | -100.000000 | 38.628159 | 177.256318 | 38.628159 | 55.956679 | 177.256318 | 55.956679 | 21.299639 | 55.956679 | 159.927798 | 55.956679 | 55.956679 | -100.000000 | 21.299639 | 55.956679 | -100.000000 | 107.942238 | 21.299639 | -100.000000 | -100.000000 | -100.000000 | 21.299639 | 38.628159 | 21.299639 | 194.584838 | -100.000000 | -100.000000 | -100.000000 | 38.628159 | 177.256318 | 55.956679 | -100.000000 | -100.000000 | -100.000000 | -100.000000 | -100.000000 | 194.584838 | -100.000000 | -100.000000 | 21.299639 | -100.000000 | 21.299639 | 21.299639 |
|  |  | hap | 25.925926 | 77.777778 | -11.111111 | -62.962963 | -25.925926 | -100.000000 | 107.407407 | -33.333333 | -100.000000 | 107.407407 | 55.555556 | -25.925926 | 40.740741 | -40.740741 | -62.962963 | -48.148148 | -3.703704 | 11.111111 | -100.000000 | -70.370370 | 18.518519 | 77.777778 | -70.370370 | -70.370370 | -33.333333 | 18.518519 | -55.555556 | -25.925926 | 3.703704 | 18.518519 | -55.555556 | -92.592593 | 85.185185 | 11.111111 | 40.740741 | 129.629630 | -100.000000 | 129.629630 | -25.925926 | 85.185185 | -25.925926 | 151.851852 | -40.740741 | -100.000000 | 137.037037 | -100.000000 | 18.518519 | 129.629630 |
|  |  | ntr | -82.385321 | -82.385321 | 14.495413 | 67.339450 | 32.110092 | -100.000000 | -100.000000 | -73.577982 | -100.000000 | -29.541284 | 111.376147 | -29.541284 | 14.495413 | 67.339450 | 14.495413 | 14.495413 | -82.385321 | -55.963303 | 67.339450 | -100.000000 | -100.000000 | -47.155963 | 5.688073 | -29.541284 | 40.917431 | -3.119266 | 14.495413 | -100.000000 | -64.770642 | 40.917431 | 32.110092 | 190.642202 | 49.724771 | 181.834862 | 76.146789 | -100.000000 | -64.770642 | -29.541284 | 76.146789 | -100.000000 | 14.495413 | -100.000000 | 340.366972 | 5.688073 | -38.348624 | -64.770642 | 181.834862 | 23.302752 |

In [16]:

```
bigWeightDf.to_csv('../outputs/weightDf.csv')
```

### do a standardization with mean zero, standard deviation 1¶

This is better for the PCA analyses we do later

In [17]:

```
from sklearn.preprocessing import StandardScaler
```

In [18]:

```
def makeStandard(logfile):
    
    # get the raw weights
    weightDf = getParticipantWeights(logfile)
    
    # initialise the scaler
    scaler = StandardScaler()
    # transfrom to numpy array, transpose because we want to
    # scale by observation and not by feature
    weightArray = np.array(weightDf.fillna(0)).T
    # scale
    scaledArray = scaler.fit( weightArray ).transform(  weightArray )
    # transform back to pandas, get the original structure back (transposing and using the old index of weight df)
    scaledDf = pd.DataFrame(scaledArray.T,index=weightDf.index)
    
    return scaledDf
```

In [19]:

```
bigWeightStandardDf = getAllWeights(logList,func=makeStandard)
```

In [20]:

```
bigWeightStandardDf.head()
```

Out[20]:

|  |  |  | 0 | 1 | 2 | 3 | 4 | 5 | 6 | 7 | 8 | 9 | 10 | 11 | 12 | 13 | 14 | 15 | 16 | 17 | 18 | 19 | 20 | 21 | 22 | 23 | 24 | 25 | 26 | 27 | 28 | 29 | 30 | 31 | 32 | 33 | 34 | 35 | 36 | 37 | 38 | 39 | 40 | 41 | 42 | 43 | 44 | 45 | 46 | 47 |
| --- | --- | --- | --- | --- | --- | --- | --- | --- | --- | --- | --- | --- | --- | --- | --- | --- | --- | --- | --- | --- | --- | --- | --- | --- | --- | --- | --- | --- | --- | --- | --- | --- | --- | --- | --- | --- | --- | --- | --- | --- | --- | --- | --- | --- | --- | --- | --- | --- | --- | --- |
| p001 | f | ang | -0.948401 | -0.006541 | 1.667878 | -1.262355 | -1.262355 | -0.320494 | -0.948401 | 0.412064 | -1.262355 | -0.948401 | 1.039971 | 1.458576 | -1.262355 | 0.098110 | 0.935320 | -1.262355 | -0.948401 | 0.412064 | -1.262355 | 0.412064 | 1.039971 | -1.262355 | -0.006541 | -0.320494 | -0.320494 | -0.843750 | 2.400437 | 0.412064 | 1.353925 | 0.412064 | 1.458576 | -0.006541 | -1.262355 | -1.262355 | -1.262355 | 0.412064 | 1.039971 | 0.098110 | -0.320494 | -0.320494 | 0.098110 | 0.098110 | 0.098110 | 1.353925 | -1.262355 | 1.039971 | 1.353925 | 1.039971 |
|  |  | dis | -0.245679 | 1.036125 | -0.074772 | -1.100216 | -1.100216 | 0.437950 | 1.548847 | 0.865218 | -0.245679 | -1.100216 | 1.121579 | -1.100216 | 0.865218 | 0.779764 | 0.608857 | -1.100216 | 0.608857 | -0.587494 | -0.074772 | -1.100216 | -1.100216 | 1.805208 | 1.890662 | 0.437950 | -0.672947 | 0.352496 | 0.096135 | -1.100216 | -0.074772 | -1.100216 | 3.257920 | -0.587494 | 0.523404 | -0.074772 | 1.292486 | 0.352496 | -1.100216 | -1.100216 | 1.463394 | -0.074772 | -1.100216 | -0.587494 | -0.074772 | -0.074772 | -0.587494 | -0.587494 | -1.100216 | -0.416586 |
|  |  | fea | 0.582234 | -1.040508 | -1.040508 | -1.040508 | 0.401929 | -1.040508 | 0.401929 | 1.844366 | 0.401929 | 0.582234 | 1.844366 | 0.582234 | 0.221624 | 0.582234 | 1.664062 | 0.582234 | 0.582234 | -1.040508 | 0.221624 | 0.582234 | -1.040508 | 1.123148 | 0.221624 | -1.040508 | -1.040508 | -1.040508 | 0.221624 | 0.401929 | 0.221624 | 2.024671 | -1.040508 | -1.040508 | -1.040508 | 0.401929 | 1.844366 | 0.582234 | -1.040508 | -1.040508 | -1.040508 | -1.040508 | -1.040508 | 2.024671 | -1.040508 | -1.040508 | 0.221624 | -1.040508 | 0.221624 | 0.221624 |
|  |  | hap | 0.352580 | 1.057741 | -0.151106 | -0.856267 | -0.352580 | -1.359953 | 1.460690 | -0.453318 | -1.359953 | 1.460690 | 0.755529 | -0.352580 | 0.554055 | -0.554055 | -0.856267 | -0.654792 | -0.050369 | 0.151106 | -1.359953 | -0.957004 | 0.251843 | 1.057741 | -0.957004 | -0.957004 | -0.453318 | 0.251843 | -0.755529 | -0.352580 | 0.050369 | 0.251843 | -0.755529 | -1.259216 | 1.158478 | 0.151106 | 0.554055 | 1.762902 | -1.359953 | 1.762902 | -0.352580 | 1.158478 | -0.352580 | 2.065113 | -0.554055 | -1.359953 | 1.863639 | -1.359953 | 0.251843 | 1.762902 |
|  |  | ntr | -0.897600 | -0.897600 | 0.157930 | 0.733673 | 0.349844 | -1.089514 | -1.089514 | -0.801643 | -1.089514 | -0.321856 | 1.213459 | -0.321856 | 0.157930 | 0.733673 | 0.157930 | 0.157930 | -0.897600 | -0.609728 | 0.733673 | -1.089514 | -1.089514 | -0.513771 | 0.061972 | -0.321856 | 0.445801 | -0.033985 | 0.157930 | -1.089514 | -0.705685 | 0.445801 | 0.349844 | 2.077074 | 0.541758 | 1.981117 | 0.829630 | -1.089514 | -0.705685 | -0.321856 | 0.829630 | -1.089514 | 0.157930 | -1.089514 | 3.708346 | 0.061972 | -0.417814 | -0.705685 | 1.981117 | 0.253887 |

In [21]:

```
bigWeightStandardDf.to_csv('../outputs/weightStdDf.csv')
```

### Do an average with the PSC weights¶

In [22]:

```
def getAverageWeight(bigWeightDf):
    bigWeightAvgDf = bigWeightDf.groupby(level=[1,2]).mean()
    return bigWeightAvgDf
```

In [23]:

```
bigAvgWeightDf = getAverageWeight(bigWeightDf)
```

In [24]:

```
bigAvgWeightDf
```

Out[24]:

|  |  | 0 | 1 | 2 | 3 | 4 | 5 | 6 | 7 | 8 | 9 | 10 | 11 | 12 | 13 | 14 | 15 | 16 | 17 | 18 | 19 | 20 | 21 | 22 | 23 | 24 | 25 | 26 | 27 | 28 | 29 | 30 | 31 | 32 | 33 | 34 | 35 | 36 | 37 | 38 | 39 | 40 | 41 | 42 | 43 | 44 | 45 | 46 | 47 |
| --- | --- | --- | --- | --- | --- | --- | --- | --- | --- | --- | --- | --- | --- | --- | --- | --- | --- | --- | --- | --- | --- | --- | --- | --- | --- | --- | --- | --- | --- | --- | --- | --- | --- | --- | --- | --- | --- | --- | --- | --- | --- | --- | --- | --- | --- | --- | --- | --- | --- |
| f | ang | -7.354947 | -7.805702 | 3.006315 | -13.034124 | -18.757770 | 8.792441 | -11.307107 | -11.584066 | -17.873372 | -7.605425 | 38.741684 | 0.621276 | -4.682816 | 2.405409 | -18.616791 | -11.215989 | -11.366503 | 10.950412 | 4.784300 | 28.271390 | 12.864789 | 18.467653 | 25.671236 | -5.595345 | -6.680401 | -12.362859 | 45.086895 | -3.527955 | 4.442992 | 77.227735 | 37.500220 | -17.032651 | -18.001299 | -11.063549 | 51.660520 | -1.913773 | -18.908472 | -1.292978 | -9.854979 | -20.863736 | -18.816326 | -19.218831 | -8.636762 | -7.564247 | -14.487180 | -13.065846 | -12.555611 | -7.847855 |
|  | dis | -18.041983 | 3.937812 | -22.837764 | -15.128121 | -13.700682 | -22.043449 | -12.648803 | -1.704436 | -13.620330 | -18.142441 | -9.516321 | 4.598998 | -1.107215 | 6.897731 | 9.686928 | -3.056760 | -16.188043 | -2.422285 | -6.733543 | -2.594785 | 1.705939 | 84.376752 | 100.801659 | -10.657749 | -21.081638 | -16.242838 | -34.212029 | -4.970155 | -16.704939 | 108.099014 | 135.119257 | 0.854224 | -21.793195 | -19.486129 | -21.509443 | 1.218984 | -16.476697 | 2.232947 | -10.681631 | 6.629351 | -0.504315 | -14.804957 | -10.364359 | -25.697650 | -10.233583 | -7.724368 | -15.547729 | -7.979228 |
|  | fea | 10.195612 | -10.988335 | -15.378811 | -4.282311 | -2.614065 | -35.234560 | -17.069579 | 6.199092 | -3.946539 | 8.085170 | 86.206851 | -19.193472 | -28.581906 | 2.563412 | -18.414976 | -8.453057 | -7.780731 | -3.063234 | -9.667070 | -9.747472 | -17.832253 | 19.944842 | 48.062710 | -34.231356 | -2.304950 | 15.550239 | 6.277398 | -7.678257 | -0.643989 | 107.042096 | 15.583183 | -11.391457 | -28.853170 | 5.480473 | 106.283975 | 4.953362 | -5.982936 | -25.016441 | -14.957941 | -23.423207 | 11.441346 | -21.123724 | -18.351739 | -6.545945 | -2.923676 | -17.097610 | 5.543373 | -26.638367 |
|  | hap | -5.777886 | -12.469828 | -5.674466 | -0.271513 | -13.049266 | -9.012192 | -13.553572 | -13.973723 | -9.404425 | -9.615958 | 2.244235 | -11.425391 | 5.510863 | 60.292422 | -0.234158 | -9.703615 | -10.783914 | -8.213909 | -0.366007 | -8.005888 | -0.299749 | 54.484599 | 16.402121 | -15.229307 | -20.567930 | -3.120583 | -5.919911 | -11.758969 | -2.171551 | 67.870283 | 3.810133 | -18.963780 | -1.792285 | -8.329770 | 12.706033 | 6.315357 | 14.370916 | 53.492159 | -11.364492 | -15.752295 | 4.270272 | 3.037072 | 0.102659 | -12.495154 | -5.793642 | 0.082287 | -15.062133 | -14.834149 |
|  | ntr | -8.954666 | -4.624652 | 12.442949 | -6.284468 | -1.687176 | -2.295681 | -12.147113 | -18.806401 | -14.510065 | -1.804427 | 57.633289 | -20.977364 | -3.796857 | 36.693662 | -4.369154 | -3.340330 | -10.724021 | 6.498163 | 4.314535 | -20.171530 | -7.437683 | 33.133762 | 56.070559 | -31.294721 | -10.342349 | -9.871887 | -16.858616 | 8.547333 | 17.819798 | 4.384095 | 27.131933 | -13.171331 | 1.502108 | -0.715810 | 91.060952 | 0.747891 | 0.511342 | -28.999534 | -12.166192 | -12.451590 | -20.105985 | 1.682182 | -0.641653 | -19.010636 | -17.481034 | -10.177632 | -7.322358 | -7.631636 |
|  | sad | 31.736929 | -18.446084 | -0.598245 | -22.573608 | -9.589268 | -5.164410 | -24.323279 | -10.299428 | 4.528918 | -18.763549 | 3.741830 | 10.316811 | -35.059796 | 5.922767 | 11.270499 | -26.336099 | 3.563097 | 8.932118 | 4.527828 | -11.666332 | -11.352183 | 13.939496 | -16.918325 | 5.357360 | -0.324169 | 83.830671 | 71.585743 | -7.914067 | 10.407456 | 30.008601 | -31.593286 | -13.306234 | -1.623850 | 22.643256 | 104.076658 | 15.100395 | -31.837752 | -13.587049 | -14.986946 | -4.859449 | -12.776357 | 13.284640 | 4.306044 | -14.779278 | -8.502192 | -29.343218 | -25.156903 | -37.399759 |
|  | sup | -19.162343 | 2.434593 | -19.036665 | -15.455387 | 7.602216 | -21.741832 | -7.972270 | -9.273324 | -0.174529 | -5.816131 | 24.494960 | -0.241916 | -15.288116 | -7.240079 | 11.478878 | -15.567426 | -16.829169 | -6.161394 | -10.639315 | -22.919173 | 4.514318 | 84.639107 | 40.494351 | -3.964504 | -14.409693 | -15.534037 | 18.448613 | -10.035833 | -17.285552 | 70.766004 | 57.299820 | 12.209205 | -2.955496 | -17.836333 | 50.485445 | -9.102044 | -6.532143 | -12.006783 | -6.841070 | -10.764172 | -12.477339 | -18.467339 | 2.793473 | -10.629182 | -3.194589 | -8.706514 | -7.785981 | -5.613311 |
| m | ang | -17.663058 | -7.649570 | 2.524516 | -10.906288 | -2.929056 | -13.779441 | -14.268044 | -41.735764 | -7.903068 | -11.083180 | 112.432564 | 6.025099 | -13.826049 | 2.008854 | -35.089346 | -15.428037 | -20.616905 | -8.504673 | 109.390032 | 21.136687 | -15.928711 | 52.255455 | 25.687165 | -8.634572 | 10.462437 | -7.203207 | 71.685566 | -10.013597 | -15.198523 | 35.846717 | 35.100870 | -27.646914 | -28.604684 | -33.055257 | 30.175350 | -15.991809 | -12.063863 | -20.293922 | -34.509698 | -0.984792 | 1.021012 | -11.059040 | 0.894651 | -9.915567 | -0.494704 | -20.365456 | -12.953386 | -10.346798 |
|  | dis | -18.482278 | 0.282525 | -5.944005 | 0.979984 | -19.151919 | -5.696637 | -6.781033 | -15.761245 | -7.380828 | -4.889681 | -17.184474 | -14.638870 | -2.207433 | -14.438903 | 5.485438 | 14.017410 | -6.999195 | -24.234363 | -7.075831 | -8.342200 | -10.560651 | 76.943898 | 88.202910 | 1.129730 | -12.364243 | -2.087663 | -7.960632 | -10.745553 | -5.459240 | 90.565915 | 66.034190 | -11.251260 | -22.996891 | -1.930320 | -7.831769 | 0.697307 | -26.037138 | 7.910946 | -12.689830 | 21.395265 | -31.670490 | -15.189221 | -11.450339 | -0.126010 | -23.335529 | -4.785251 | -3.548539 | 27.583943 |
|  | fea | -10.099872 | -25.409541 | -5.964443 | -14.840034 | -3.721446 | -14.381879 | -0.518063 | -3.905476 | 0.693834 | -8.298492 | 75.718228 | 0.521190 | -21.617385 | 4.917647 | 14.197242 | 16.056415 | -39.360988 | 3.009171 | 14.998443 | -12.783217 | -14.203733 | 41.939787 | 25.434083 | -25.710421 | -13.111412 | -23.718081 | 52.655279 | -5.276821 | -15.860051 | 21.664701 | 31.424657 | -17.343976 | -0.032562 | -15.850102 | 114.314228 | 3.451942 | 6.323916 | -20.338768 | 16.396038 | -9.156165 | -9.482092 | -34.211555 | 1.513722 | -16.699008 | -14.541543 | -8.518552 | -27.994495 | -12.280350 |
|  | hap | -4.659852 | -20.685601 | -4.239779 | -7.556644 | -13.644443 | -8.153146 | -2.697878 | -15.817821 | -12.245609 | -12.144300 | 19.154388 | 3.800027 | 11.059609 | 46.074150 | -3.309115 | -13.860971 | -18.059792 | -18.462172 | -5.464579 | -4.214569 | 5.372723 | 58.582397 | 3.465367 | -15.322516 | -9.324975 | -21.123046 | -4.485627 | -0.368592 | -8.316060 | 68.959204 | 25.778719 | -2.201520 | -18.523168 | -16.310742 | 1.743523 | -4.057643 | 11.825179 | 70.311343 | 5.271624 | -17.996169 | -2.735678 | -2.694451 | -6.425586 | -0.888636 | -7.113647 | -2.112741 | -15.202955 | -10.978230 |
|  | ntr | -16.366721 | -10.237236 | -5.728343 | -16.153067 | -4.213632 | 10.906716 | -9.751179 | 8.246358 | 0.778722 | 1.340415 | 17.864153 | -18.769981 | -6.873185 | 54.583664 | -6.194222 | -15.762371 | -15.751746 | -2.102904 | 6.617723 | -8.973064 | 24.875007 | 33.442057 | 5.889421 | -2.032198 | -14.900391 | -2.267001 | 2.241122 | 0.302698 | 12.986474 | 39.972239 | 28.878816 | 1.720198 | -20.753405 | -28.521118 | 12.439456 | -10.612688 | -5.737669 | 11.798930 | 5.922485 | -10.881295 | -25.989944 | -11.183909 | 11.670238 | -15.817533 | 4.417427 | -10.820877 | -7.714487 | 7.215845 |
|  | sad | -13.938410 | -11.938944 | -17.795451 | -37.381813 | -9.654425 | -12.775000 | -16.716372 | -19.635504 | -19.552301 | -7.869316 | 18.203488 | 3.016253 | -1.137024 | 12.426479 | -6.539003 | 6.052890 | -23.820698 | 11.664872 | -3.164216 | -5.021492 | 20.876684 | -10.598687 | 9.050630 | -8.437051 | -20.863338 | 50.097088 | 24.788589 | 47.654755 | -24.530534 | 15.329786 | 37.218296 | -8.078543 | -35.318726 | -4.038455 | 178.326270 | 38.121394 | -31.452052 | -33.094149 | -46.635487 | 9.254653 | -15.498880 | -22.762840 | -1.883445 | -2.271320 | -5.402293 | 4.571600 | -10.154791 | 1.306835 |
|  | sup | -6.663781 | -11.952487 | 3.328154 | -2.854515 | -3.976193 | -22.930099 | -19.472121 | -15.648956 | -1.350623 | -1.772803 | 55.015682 | -16.820610 | -5.621167 | -10.271319 | -13.154675 | -5.771151 | 11.587691 | 8.250532 | 16.902900 | -24.670604 | 12.246045 | 47.576409 | 32.251379 | -10.818276 | 2.924360 | -1.585859 | 19.904238 | -13.096919 | -7.087560 | 78.954684 | 17.890613 | -18.305083 | 15.222024 | -9.955520 | 54.120386 | -16.870222 | -4.361058 | -16.580632 | 1.897671 | -20.899896 | -20.728216 | -0.630846 | -11.278262 | -4.412029 | -15.714577 | -18.794030 | -12.160547 | -11.862130 |

In [25]:

```
bigAvgWeightDf.to_csv('../outputs/weightAvgDf.csv')
```

### Do an average with the normalized weights¶

In [26]:

```
bigAvgStdWeightDf = getAverageWeight(bigWeightStandardDf)
```

In [27]:

```
bigAvgStdWeightDf.to_csv('../outputs/weightAvgStdDf.csv')
```

### Functions for Tile plotting:¶

Get a list of all possible coordinates:

In [28]:

```
def makeCoordinates(xNum,yNum,squareSize):
    myArray = []
    xDim=xNum*squareSize
    yDim=yNum*squareSize
    for x in np.arange(0,xDim,squareSize):
        for y in np.arange(0,yDim,squareSize):
            myArray.append( (x,y) )
    
    return myArray
```

Example:

In [29]:

```
coords =  makeCoordinates(8,6,50)

print coords
```

```
[(0, 0), (0, 50), (0, 100), (0, 150), (0, 200), (0, 250), (50, 0), (50, 50), (50, 100), (50, 150), (50, 200), (50, 250), (100, 0), (100, 50), (100, 100), (100, 150), (100, 200), (100, 250), (150, 0), (150, 50), (150, 100), (150, 150), (150, 200), (150, 250), (200, 0), (200, 50), (200, 100), (200, 150), (200, 200), (200, 250), (250, 0), (250, 50), (250, 100), (250, 150), (250, 200), (250, 250), (300, 0), (300, 50), (300, 100), (300, 150), (300, 200), (300, 250), (350, 0), (350, 50), (350, 100), (350, 150), (350, 200), (350, 250)]
```

Cut out a tile and change its red value:

In [30]:

```
def getCut( im, pValue, h, v, squareSize ):
    
    # cut out a part
    cut = im.crop((h,v,h+squareSize,v+squareSize))
    pixdata=cut.load()
        
    # change its transparency by looping through all the pixels of the cutout
    for y in xrange(cut.size[1]):
        for x in xrange(cut.size[0]):
            r,g,b,a =  pixdata[x, y]
            pixdata[x, y] = (int(255*pValue),g,b,a) # alternative visualisation

    return cut
```

Example:

In [31]:

```
getCut(Image.open(picList[0][0],'r'),0.,coords[0][0],coords[0][1],50 )
```

Out[31]:

In [32]:

```
getCut(Image.open(picList[0][0],'r'),1.,coords[1][0],coords[1][1],50 )
```

Out[32]:

Weights are rescaled to be between 0 and 1 for plotting purposes only:

In [33]:

```
def minMaxScale(d):
    
    # turn into pandas df
    df = pd.DataFrame(d,index=["num"]).T
    # we only do this for one column of data
    assert df.shape[1] == 1, "function not specified for multiple columns"
    
    # we apply min/max scaling in the standard way
    outDf = ( df-df.min() ) / ( df.max()-df.min() +.000000000001 ) # if min=max, we dont want to divide by zero
    
    # return the rescaled data (0-1) as a dictionary
    outDict = outDf.to_dict()
    # get rid of the redundant hierarchy of the dict (i.e. return the values of the first and only key)
    return outDict[ outDict.keys()[-1] ]
```

Example for the happy female face:

In [34]:

```
thisDict = bigWeightDf.ix['p001'].ix['f'].ix['hap'].to_dict()
print 'min:', float( pd.DataFrame(thisDict,index=['val']).T.min() )
print 'max:', float( pd.DataFrame(thisDict,index=['val']).T.max() )
```

```
min: -100.0
max: 151.851851852
```

In [35]:

```
minMaxDict = minMaxScale(thisDict)
print 'min:', float( pd.DataFrame(minMaxDict,index=['val']).T.min() )
print 'max:', float( pd.DataFrame(minMaxDict,index=['val']).T.max() )
```

```
min: 0.0
max: 1.0
```

Apply to one whole image:

In [36]:

```
def applyTransparency(im,pDict,dims,squareSize):
    
    #rescale the dict using min/max scaling
    pDict = minMaxScale(pDict)
    
    # dimension of the image
    width=dims[1]*squareSize
    height=dims[0]*squareSize
    # make all x,y coordinates for the image
    thisCoord = makeCoordinates(dims[1],dims[0],squareSize)
    # take the original image and convert it, so it has an alpha channel
    im = im.convert("RGBA")
    im = im.resize((width,height), PIL.Image.ANTIALIAS)
    imDim = im.getdata.im_self.size
    pixdata = im.load()
    
    # create an empty output image to which we will write
    imOut = Image.new("RGBA",
                      imDim,
                      (0,0,0))

    # for each position of a tile and its probability of being shown
    for key,pValue in pDict.iteritems():
        # get the position of the current square

        h,v = thisCoord[int(key)]
        # cut that square and apply the proability value
        cut = getCut( im, pValue, h, v, squareSize )
        # add the edited cutout to the output image
        imOut.paste(cut, (h,v))
        
    return imOut
```

Example:

In [37]:

```
applyTransparency(Image.open(picList[0][0],'r'),
                  bigWeightDf.ix['p001'].ix['f'].ix['hap'].to_dict(),
                  (8,6),
                  50)
```

Out[37]:

## The big plot¶

In [38]:

```
sns.set_style("white")
```

In [39]:

```
def plotAll(bigWeightDf,picList=picList,identDict=identDict,emoDict=emoDict):
    
    fig = plt.figure( figsize=(16,8) )

    i = 1 # counter for subplots
    for ident in range(2):
        for emo in range(7):
            pDict = bigWeightDf.ix[identDict[ident] ].ix[emoDict[emo] ].to_dict()
            im=Image.open(picList[ident][emo],'r')
            dims=(8,6);squareSize=50
            thisIm = applyTransparency(im,pDict,dims,squareSize)
            ax = plt.subplot(2,7,i)
            ax.imshow(thisIm)
            ax.set_yticks([]); ax.set_xticks([])
            i+=1
    plt.tight_layout()
    plt.savefig('../figures/mainWeightPlot.png',dpi=100)
    plt.show()
```

In [40]:

```
plotAll(bigAvgWeightDf)
```
