# Supplementary material for: Mapping the emotional face. How individual face parts contribute to successful emotion recognition
Source: PLoS One. 2017 May 11;12(5):e0177239. doi: 10.1371/journal.pone.0177239 (PMC5426715; doi:10.1371/journal.pone.0177239)
Supplement: S5 Code — (HTML) [file pone.0177239.s007.html]

code004\_actionUnits\_and\_upperLower


# Mapping the emotional face. How individual face parts contribute to successful emotion recognition.

# 4a. Plotting Results by Action Units¶

This notebook aggregates the tiles in accordance to the Ekman Action Units (AUs) they fall into.

The AUs have been manually defined on each individual face and each AU has a color code that identifies it.
Now, the algorithm assigns each tile one or a number of AUs that are contained in it, so each tile has either an AU label, or it represents no AU.

### importing the basic modules and files¶

In [1]:

```
from myBasics import *
%matplotlib inline
```

In [2]:

```
logList = getFile('../rawTables/','pand*.csv')
```

## Visualize the hand-drawn Action Units (AUs)¶

#### Example:¶

Get all the AUs for one picture. This is a list of single images, one per AU, where each AU is drawn in a different color.

In [3]:

```
myPicList = getFile('../auLabels/','f_ang*.png')
```

Now we have an array with all AU's that belong to the female angry face (f\_ang):

In [4]:

```
myPicList
```

Out[4]:

```
['../auLabels/f_ang_AU22+23+25.png',
 '../auLabels/f_ang_AU6.png',
 '../auLabels/f_ang_AU7.png',
 '../auLabels/f_ang_AU9.png']
```

For illustration purposes, here we plot all the hand-drawn AU's of that face

In [5]:

```
fig = plt.figure(figsize=(12,4))
for i,pic in enumerate(myPicList):
    im = Image.open(pic)
    ax = plt.subplot(1,len(myPicList),i+1)
    ax.set_title(pic[pic.rfind('/')+1:],fontsize=12)
    ax.set_xticks([]);ax.set_yticks([])
    ax.imshow(im);
```

### Simple visualisation of all AUs on the faces¶

In [6]:

```
myPaintList =  []
for fGender in ['f','m']:
    thisList = []
    for fEmo in myLabels.values()[:-1]:
        thisFace = getFile('../auLabels/auVisualisation/',fGender+'_'+fEmo+'*.png')[-1]
        thisList.append(thisFace)
    myPaintList.append(thisList)
```

In [7]:

```
myPaintList
```

Out[7]:

```
[['../auLabels/auVisualisation/f_hap_w_cut.png',
  '../auLabels/auVisualisation/f_sad_w_cut.png',
  '../auLabels/auVisualisation/f_ang_w_cut.png',
  '../auLabels/auVisualisation/f_fea_w_cut.png',
  '../auLabels/auVisualisation/f_dis_w_cut.png',
  '../auLabels/auVisualisation/f_sup_w_cut.png'],
 ['../auLabels/auVisualisation/m_hap_m_cut.png',
  '../auLabels/auVisualisation/m_sad_m_cut.png',
  '../auLabels/auVisualisation/m_ang_m_cut.png',
  '../auLabels/auVisualisation/m_fea_m_cut.png',
  '../auLabels/auVisualisation/m_dis_m_cut.png',
  '../auLabels/auVisualisation/m_sup_m_cut.png']]
```

In [8]:

```
def makePicturePlot(myPaintList):
    
    fig = plt.figure( figsize=(16,8) )
    i = 1 # counter for subplots
    for ident in range(2):
        for emo in range(6):

            im=Image.open(myPaintList[ident][emo],'r')

            ax = plt.subplot(2,6,i)
            ax.imshow(im)
            ax.set_yticks([]); ax.set_xticks([])

            i+=1
    plt.savefig('../figures/auIllustration.png',dpi=300)
    plt.show()
```

In [9]:

```
makePicturePlot(myPaintList)
```

## Assign AUs to tiles¶

Get a list of all possible tile coordinates

In [10]:

```
def makeCoordinates(xNum,yNum,squareSize):
    myArray = []
    xDim=xNum*squareSize
    yDim=yNum*squareSize
    for x in np.arange(0,xDim,squareSize):
        for y in np.arange(0,yDim,squareSize):
            myArray.append( (x,y) )
    
    return myArray
```

In [11]:

```
thisCoord = makeCoordinates(6,8,50)
print thisCoord
print "\nNumber of Coordinates:", len(makeCoordinates(6,8,50))
```

```
[(0, 0), (0, 50), (0, 100), (0, 150), (0, 200), (0, 250), (0, 300), (0, 350), (50, 0), (50, 50), (50, 100), (50, 150), (50, 200), (50, 250), (50, 300), (50, 350), (100, 0), (100, 50), (100, 100), (100, 150), (100, 200), (100, 250), (100, 300), (100, 350), (150, 0), (150, 50), (150, 100), (150, 150), (150, 200), (150, 250), (150, 300), (150, 350), (200, 0), (200, 50), (200, 100), (200, 150), (200, 200), (200, 250), (200, 300), (200, 350), (250, 0), (250, 50), (250, 100), (250, 150), (250, 200), (250, 250), (250, 300), (250, 350)]

Number of Coordinates: 48
```

### Small script to get the rgb color of each mask¶

In [12]:

```
def getColor(imName):

    im = Image.open(imName)
    # we loop through all pixels
    for y in xrange(im.size[1]):
        for x in xrange(im.size[0]):
            # we get the RGB+alpha values of each pixel
            r,g,b,a =  im.load()[x, y]
            # we are only intereset in colored pixels
            if (r,g,b) != (0,0,0):
                # store the present color for later re-use
                thisColor = (r,g,b)
                # and stop here
                break
    return thisColor
```

In [13]:

```
getColor( myPicList[-1] )
```

Out[13]:

```
(127, 188, 203)
```

### Determining for each tile, whether it contains some colored pixels¶

We loop through the image, tile by tile. For this, we use For each tile, count the occurence of each color on a pixel-by-pixel basis. We use the thisCoord list of all tile coordinates (where each coordinate tuple denotes the upper left corner of the 50x50 tile).  
The first output is a dictionary, with tile coordinates as keys and number of colored pixels as values.  
The second output is the RGB value of the color used for that AU. Since each image contains only one AU and should have only one color, this works by taking the color of any non-black (0,0,0) pixel.

In [14]:

```
def getTiles(im,thisCoord=thisCoord,squareSize=50):
    
    # resize the image, if it is not exactly 300x400 (as it was presented in the experiment),
    # this should not be necessary in general
    im = im.resize((300,400))
    
    # the dictionary where we write the occurence of colored pixels for each tile
    thisCut = {}

    # we loop through the coordinate list
    for coord in thisCoord:
        # we get the (h)orizontal and (v)ertical coordinates from the tuple
        h,v = coord

        # we cut out the tile
        cut = im.crop((h,v,h+squareSize,v+squareSize))
        pixdata=cut.load()

        # we loop through all pixels of that tile
        for y in xrange(cut.size[1]):
            for x in xrange(cut.size[0]):
                # we get the RGB+alpha values of each pixel
                r,g,b,a =  pixdata[x, y]
                # we are only intereset in colored pixels
                if (r,g,b) != (0,0,0):
                    # store the present color for later re-use
                    thisColor = (r,g,b)
                    # count the occurence of colored pixels for that tile/coordinate pair
                    try:
                        thisCut[coord]+=1
                    except:
                        thisCut[coord]=1

        # if the tile remained empty, assign zero
        if coord not in thisCut:
            thisCut[coord] = 0
    
    # return the dict and a tuple with RGB values of that Action Unit
    return thisCut,thisColor
```

Example:

In [15]:

```
print getTiles(im)
```

```
({(150, 100): 517, (150, 250): 0, (0, 250): 0, (250, 250): 0, (100, 50): 0, (50, 100): 649, (50, 250): 0, (100, 300): 0, (250, 100): 143, (0, 350): 0, (0, 200): 0, (100, 350): 0, (200, 100): 587, (50, 0): 0, (50, 150): 0, (100, 200): 0, (200, 150): 0, (0, 50): 0, (0, 300): 0, (100, 250): 0, (200, 200): 0, (150, 200): 0, (50, 50): 0, (250, 150): 0, (250, 300): 0, (150, 350): 0, (0, 150): 0, (0, 0): 0, (200, 300): 0, (250, 350): 0, (200, 0): 0, (200, 350): 0, (200, 50): 0, (0, 100): 160, (150, 0): 0, (250, 200): 0, (250, 50): 0, (50, 300): 0, (150, 150): 166, (100, 100): 354, (150, 50): 0, (100, 150): 128, (250, 0): 0, (150, 300): 0, (50, 200): 0, (50, 350): 0, (200, 250): 0, (100, 0): 0}, (127, 170, 212))
```

### get tiles for all AUs¶

In [16]:

```
def makeAUTable(myPicList):
    
    # get number of colored pixels in each tile for all AUs of a face
    d = {}
    for pic in myPicList:
        d[pic] = getTiles( Image.open(pic) )
        
    # transform this into a dataframe
    bigDf = pd.DataFrame()
    for key in d:
        thisDf = pd.DataFrame(d[key][0],index=[key]).T
        bigDf = pd.concat([bigDf,thisDf],axis=1)

    return bigDf
```

In [17]:

```
makeAUTable(myPicList).head()
```

Out[17]:

|  |  | ../auLabels/f\_ang\_AU9.png | ../auLabels/f\_ang\_AU7.png | ../auLabels/f\_ang\_AU6.png | ../auLabels/f\_ang\_AU22+23+25.png |
| --- | --- | --- | --- | --- | --- |
| 0 | 0 | 0 | 0 | 0 | 0 |
|  | 50 | 0 | 0 | 0 | 0 |
|  | 100 | 160 | 0 | 36 | 0 |
|  | 150 | 0 | 0 | 185 | 0 |
|  | 200 | 0 | 0 | 0 | 0 |

Binarize in a way that multiple AUs can belong to a tile, but only if they pass a defined cutoff

In [18]:

```
def makeBinAUs(bigDf,cutOff=25):
         
    return (bigDf>cutOff).astype(int)
```

In [19]:

```
makeBinAUs( makeAUTable(myPicList) ).head()
```

Out[19]:

|  |  | ../auLabels/f\_ang\_AU9.png | ../auLabels/f\_ang\_AU7.png | ../auLabels/f\_ang\_AU6.png | ../auLabels/f\_ang\_AU22+23+25.png |
| --- | --- | --- | --- | --- | --- |
| 0 | 0 | 0 | 0 | 0 | 0 |
|  | 50 | 0 | 0 | 0 | 0 |
|  | 100 | 1 | 0 | 1 | 0 |
|  | 150 | 0 | 0 | 1 | 0 |
|  | 200 | 0 | 0 | 0 | 0 |

In [20]:

```
d = makeBinAUs( makeAUTable(myPicList) ).to_dict()
```

### visualize the result¶

In [21]:

```
def showTiles(bgIm,d,squareSize=50):

    imDict = {}

    # loop through the action units
    for au in d:
        
        # get the face image as a background to visualize on
        imOut = Image.open(bgIm)
        imOut = imOut.resize((300,400))
    
        # loop through the tile coordinates
        for coords in d[au]:

            h,v = coords
            if d[au][coords] == 1:
                thisColor = getColor(au)
            else:
                thisColor = 0

            faceTile = imOut.crop((h,v,h+squareSize,v+squareSize))

            if thisColor == 0:

                imOut.paste(faceTile, (h,v))

            else:
                pixdata=faceTile.load()

                # change its color by looping through all the pixels of the cutout
                for y in xrange(faceTile.size[1]):
                    for x in xrange(faceTile.size[0]):
                        pixdata[x, y] = thisColor
                    # paste the fully colored tile onto the background image
                    imOut.paste(faceTile, (h,v))
                    
        imDict[au] = imOut
        
    return imDict
```

In [22]:

```
d = makeBinAUs( makeAUTable(myPicList) ).to_dict()
imDict = showTiles(picList[0][2],d)
```

In [23]:

```
fig = plt.figure(figsize=(16,6))
for i,im in enumerate(imDict):
    ax = plt.subplot( 1, len(imDict.keys()), i+1 )
    ax.set_title(im[im.rfind('/')+1:],fontsize=12)
    ax = plt.imshow(imDict[im])
    plt.xticks([]); plt.yticks([])
plt.show()
```

### Get all the pictures and their respective AU lists¶

Here we define a dictionary *picDict*, wich is used to store the filenames of the original image and the AU images for each face.

In [24]:

```
def makePicDict(picList):
    
    picDict = {}
    for i,ident in enumerate(picList):
        for e,emo in enumerate(picList[i]):
            thisPic = picList[i][e]

            thisName = thisPic[thisPic.find('_')-1:thisPic.rfind('_')]

            myPicList = getFile('../auLabels/',thisName+'*.png')

            picDict[thisName] = {'auList':myPicList,
                                 'picFile':thisPic}
    return picDict
```

In [25]:

```
picDict = makePicDict(picList)
```

In [26]:

```
picDict
```

Out[26]:

```
{'f_ang': {'auList': ['../auLabels/f_ang_AU22+23+25.png',
   '../auLabels/f_ang_AU6.png',
   '../auLabels/f_ang_AU7.png',
   '../auLabels/f_ang_AU9.png'],
  'picFile': '../experiment/app/static/img/f_ang_cut.png'},
 'f_dis': {'auList': ['../auLabels/f_dis_AU10+16+19+25+26.png',
   '../auLabels/f_dis_AU4.png',
   '../auLabels/f_dis_AU6.png',
   '../auLabels/f_dis_AU9.png'],
  'picFile': '../experiment/app/static/img/f_dis_cut.png'},
 'f_fea': {'auList': ['../auLabels/f_fea_AU1.png',
   '../auLabels/f_fea_AU2.png',
   '../auLabels/f_fea_AU20+25.png',
   '../auLabels/f_fea_AU5.png'],
  'picFile': '../experiment/app/static/img/f_fea_cut.png'},
 'f_hap': {'auList': ['../auLabels/f_hap_AU12.png',
   '../auLabels/f_hap_AU25.png',
   '../auLabels/f_hap_AU6.png'],
  'picFile': '../experiment/app/static/img/f_hap_cut.png'},
 'f_ntr': {'auList': ['../auLabels/f_ntr_AU25.png'],
  'picFile': '../experiment/app/static/img/f_ntr_cut.png'},
 'f_sad': {'auList': ['../auLabels/f_sad_AU1+4.png',
   '../auLabels/f_sad_AU15+25.png'],
  'picFile': '../experiment/app/static/img/f_sad_cut.png'},
 'f_sup': {'auList': ['../auLabels/f_sup_AU1.png',
   '../auLabels/f_sup_AU2.png',
   '../auLabels/f_sup_AU25+26+27.png',
   '../auLabels/f_sup_AU5.png'],
  'picFile': '../experiment/app/static/img/f_sup_cut.png'},
 'm_ang': {'auList': ['../auLabels/m_ang_AU25+29.png',
   '../auLabels/m_ang_AU4.png',
   '../auLabels/m_ang_AU7.png'],
  'picFile': '../experiment/app/static/img/m_ang_cut.png'},
 'm_dis': {'auList': ['../auLabels/m_dis_AU10+16+19+25+26.png',
   '../auLabels/m_dis_AU4.png',
   '../auLabels/m_dis_AU6.png',
   '../auLabels/m_dis_AU9.png'],
  'picFile': '../experiment/app/static/img/m_dis_cut.png'},
 'm_fea': {'auList': ['../auLabels/m_fea_AU1.png',
   '../auLabels/m_fea_AU2.png',
   '../auLabels/m_fea_AU20+25.png',
   '../auLabels/m_fea_AU5.png'],
  'picFile': '../experiment/app/static/img/m_fea_cut.png'},
 'm_hap': {'auList': ['../auLabels/m_hap_AU12.png',
   '../auLabels/m_hap_AU25.png',
   '../auLabels/m_hap_AU6.png'],
  'picFile': '../experiment/app/static/img/m_hap_cut.png'},
 'm_ntr': {'auList': ['../auLabels/m_ntr_AU25.png'],
  'picFile': '../experiment/app/static/img/m_ntr_cut.png'},
 'm_sad': {'auList': ['../auLabels/m_sad_AU1+4.png',
   '../auLabels/m_sad_AU15+25.png'],
  'picFile': '../experiment/app/static/img/m_sad_cut.png'},
 'm_sup': {'auList': ['../auLabels/m_sup_AU1.png',
   '../auLabels/m_sup_AU2.png',
   '../auLabels/m_sup_AU25+26+27.png',
   '../auLabels/m_sup_AU5.png'],
  'picFile': '../experiment/app/static/img/m_sup_cut.png'}}
```

make a list with face names, which is sorted in a nice way, so we can use it for looping

In [27]:

```
picSort = []
for entry in picList:
    for subentry in entry:
        picSort.append(subentry[subentry.rfind('/')+1:subentry.rfind('_')])
print picSort
```

```
['f_hap', 'f_sad', 'f_ang', 'f_fea', 'f_dis', 'f_sup', 'f_ntr', 'm_hap', 'm_sad', 'm_ang', 'm_fea', 'm_dis', 'm_sup', 'm_ntr']
```

### show all the tile-assignments of action units¶

In [28]:

```
picDict[picSort[0]]
```

Out[28]:

```
{'auList': ['../auLabels/f_hap_AU12.png',
  '../auLabels/f_hap_AU25.png',
  '../auLabels/f_hap_AU6.png'],
 'picFile': '../experiment/app/static/img/f_hap_cut.png'}
```

In [29]:

```
for entry in picSort:
    
    d = makeBinAUs( makeAUTable(picDict[entry]['auList'])).to_dict()
    imDict = showTiles(picDict[entry]['picFile'],d)

    fig = plt.figure(figsize=(16,6))
    for i,im in enumerate(imDict):
        ax = plt.subplot( 1, 4, i+1 )
        imName = im[im.rfind('_')+1:im.rfind('.')]
        ax.set_title(imName)
        ax.set_xticks([]);ax.set_yticks([])
        ax = plt.imshow(imDict[im])
    saveName = '../figures/auAssignments/'+str(im[im.rfind('/')+1:im.rfind('_')])
    plt.savefig(saveName,dpi=300)
    plt.show()
```

## Derive behavioral metric¶

These are the acutal behavioral data, which we now want to apply not to single tiles, but to groups of tiles which all belong to the same action unit. The algorithms to derive the values are esentially the same as in the previous notebooks.

In [30]:

```
metricDf = pd.read_csv('../outputs/weightDf.csv',index_col=[0,1,2])
metricDf.index.names = ['p','ident','emo']
metricDf = metricDf.sortlevel()
```

In [31]:

```
metricDf.tail()
```

Out[31]:

|  |  |  | 0 | 1 | 2 | 3 | 4 | 5 | 6 | 7 | 8 | 9 | 10 | 11 | 12 | 13 | 14 | 15 | 16 | 17 | 18 | 19 | 20 | 21 | 22 | 23 | 24 | 25 | 26 | 27 | 28 | 29 | 30 | 31 | 32 | 33 | 34 | 35 | 36 | 37 | 38 | 39 | 40 | 41 | 42 | 43 | 44 | 45 | 46 | 47 |
| --- | --- | --- | --- | --- | --- | --- | --- | --- | --- | --- | --- | --- | --- | --- | --- | --- | --- | --- | --- | --- | --- | --- | --- | --- | --- | --- | --- | --- | --- | --- | --- | --- | --- | --- | --- | --- | --- | --- | --- | --- | --- | --- | --- | --- | --- | --- | --- | --- | --- | --- |
| p | ident | emo |  |  |  |  |  |  |  |  |  |  |  |  |  |  |  |  |  |  |  |  |  |  |  |  |  |  |  |  |  |  |  |  |  |  |  |  |  |  |  |  |  |  |  |  |  |  |  |  |
| p096 | m | fea | -100.000000 | 17.391304 | 17.391304 | 121.739130 | 4.347826 | 121.739130 | 43.478261 | -8.695652 | -100.000000 | -100.000000 | 134.782609 | 4.347826 | 30.434783 | 82.608696 | 160.869565 | -100.000000 | -8.695652 | -100.000000 | 4.347826 | 4.347826 | -100.000000 | 4.347826 | 134.782609 | -100.000000 | 4.347826 | -100.000000 | 134.782609 | 4.347826 | -100.000000 | 17.391304 | -8.695652 | -8.695652 | -8.695652 | -8.695652 | 213.043478 | 17.391304 | -8.695652 | -100.000000 | 30.434783 | 17.391304 | 17.391304 | -100.000000 | 121.739130 | -100.000000 | -8.695652 | 4.347826 | -100.000000 | -100.000000 |
|  |  | hap | 2.595420 | 9.923664 | 24.580153 | 53.893130 | -41.374046 | -56.030534 | -56.030534 | -56.030534 | -100.000000 | -12.061069 | 112.519084 | 17.251908 | 61.221374 | -63.358779 | -100.000000 | 68.549618 | -100.000000 | 2.595420 | 90.534351 | -56.030534 | 90.534351 | 75.877863 | -63.358779 | -56.030534 | -92.671756 | 134.503817 | -26.717557 | 61.221374 | -48.702290 | 112.519084 | -85.343511 | -4.732824 | 31.908397 | -70.687023 | 46.564885 | -26.717557 | 2.595420 | 332.366412 | -12.061069 | -34.045802 | -19.389313 | -19.389313 | -100.000000 | -41.374046 | -12.061069 | 53.893130 | 9.923664 | -41.374046 |
|  |  | ntr | 290.243902 | -100.000000 | -100.000000 | -100.000000 | -100.000000 | -100.000000 | -100.000000 | 251.219512 | -100.000000 | 290.243902 | 251.219512 | -100.000000 | 251.219512 | 641.463415 | -100.000000 | -100.000000 | -100.000000 | -100.000000 | -100.000000 | -100.000000 | -100.000000 | -100.000000 | -100.000000 | -100.000000 | -100.000000 | -100.000000 | -100.000000 | -100.000000 | -100.000000 | -100.000000 | 290.243902 | 251.219512 | -100.000000 | -100.000000 | -100.000000 | -100.000000 | -100.000000 | -100.000000 | -100.000000 | 251.219512 | -100.000000 | -100.000000 | 251.219512 | -100.000000 | 290.243902 | -100.000000 | 290.243902 | -100.000000 |
|  |  | sad | -100.000000 | 1.052632 | 102.105263 | -100.000000 | -100.000000 | -24.210526 | 26.315789 | -24.210526 | 304.210526 | 102.105263 | -100.000000 | 127.368421 | 1.052632 | -100.000000 | -100.000000 | -100.000000 | 26.315789 | 203.157895 | 76.842105 | 1.052632 | -100.000000 | -100.000000 | 102.105263 | -100.000000 | 102.105263 | -24.210526 | 304.210526 | -100.000000 | -100.000000 | 26.315789 | 1.052632 | 127.368421 | -100.000000 | -24.210526 | 1.052632 | -100.000000 | -100.000000 | -24.210526 | 1.052632 | 26.315789 | -24.210526 | 102.105263 | 1.052632 | 203.157895 | -24.210526 | -100.000000 | -100.000000 | -100.000000 |
|  |  | sup | -69.953052 | 12.676056 | -17.370892 | -69.953052 | -100.000000 | -100.000000 | -100.000000 | 27.699531 | -9.859155 | 42.723005 | -100.000000 | -24.882629 | 110.328638 | -100.000000 | 305.633803 | 20.187793 | 57.746479 | -69.953052 | -2.347418 | -17.370892 | 140.375587 | 132.863850 | 87.793427 | -100.000000 | 50.234742 | 42.723005 | -54.929577 | 20.187793 | 35.211268 | 140.375587 | -47.417840 | -54.929577 | 27.699531 | -100.000000 | 57.746479 | -32.394366 | -9.859155 | 27.699531 | -17.370892 | -2.347418 | -39.906103 | -100.000000 | -24.882629 | 27.699531 | -100.000000 | 110.328638 | -17.370892 | 5.164319 |

In [32]:

```
def getWeight(metricDf,ident,emo,identDict=identDict,emoDict=emoDict):
    outDf = pd.DataFrame()
    for entry in metricDf.index.levels[0]:
        thisDf = pd.DataFrame( metricDf.ix[entry].ix[identDict[ident]].ix[emoDict[emo]] ).T
        thisDf.index = [entry]
        outDf = pd.concat([outDf,thisDf])
    outDf = outDf.sortlevel()
    return outDf
```

Table of that condition, with all participants

In [33]:

```
getWeight(metricDf,0,0).head()
```

Out[33]:

|  | 0 | 1 | 2 | 3 | 4 | 5 | 6 | 7 | 8 | 9 | 10 | 11 | 12 | 13 | 14 | 15 | 16 | 17 | 18 | 19 | 20 | 21 | 22 | 23 | 24 | 25 | 26 | 27 | 28 | 29 | 30 | 31 | 32 | 33 | 34 | 35 | 36 | 37 | 38 | 39 | 40 | 41 | 42 | 43 | 44 | 45 | 46 | 47 |
| --- | --- | --- | --- | --- | --- | --- | --- | --- | --- | --- | --- | --- | --- | --- | --- | --- | --- | --- | --- | --- | --- | --- | --- | --- | --- | --- | --- | --- | --- | --- | --- | --- | --- | --- | --- | --- | --- | --- | --- | --- | --- | --- | --- | --- | --- | --- | --- | --- |
| p001 | 25.925926 | 77.777778 | -11.111111 | -62.962963 | -25.925926 | -100.000000 | 107.407407 | -33.333333 | -100.000000 | 107.407407 | 55.555556 | -25.925926 | 40.740741 | -40.740741 | -62.962963 | -48.148148 | -3.703704 | 11.111111 | -100.000000 | -70.370370 | 18.518519 | 77.777778 | -70.370370 | -70.370370 | -33.333333 | 18.518519 | -55.555556 | -25.925926 | 3.703704 | 18.518519 | -55.555556 | -92.592593 | 85.185185 | 11.111111 | 40.740741 | 129.629630 | -100.000000 | 129.629630 | -25.925926 | 85.185185 | -25.925926 | 151.851852 | -40.740741 | -100.000000 | 137.037037 | -100.000000 | 18.518519 | 129.629630 |
| p002 | 64.928910 | 64.928910 | 59.241706 | -26.066351 | -20.379147 | -100.000000 | 70.616114 | -43.127962 | -43.127962 | 30.805687 | 64.928910 | -14.691943 | -14.691943 | -100.000000 | -100.000000 | -26.066351 | -60.189573 | 2.369668 | -94.312796 | -37.440758 | 8.056872 | 110.426540 | -100.000000 | -100.000000 | -100.000000 | 87.677725 | -26.066351 | 36.492891 | -20.379147 | 30.805687 | -26.066351 | -100.000000 | 93.364929 | 30.805687 | 76.303318 | 138.862559 | -100.000000 | 47.867299 | -20.379147 | 64.928910 | -20.379147 | 195.734597 | -94.312796 | -100.000000 | 81.990521 | -94.312796 | 53.554502 | 167.298578 |
| p003 | -30.012151 | -53.341434 | 39.975699 | -100.000000 | -70.838396 | 80.801944 | 10.814095 | -24.179830 | 39.975699 | 4.981774 | -0.850547 | -100.000000 | 104.131227 | 4.981774 | -82.503038 | -0.850547 | -41.676792 | -100.000000 | 45.808019 | -59.173755 | -35.844471 | 80.801944 | 28.311057 | -47.509113 | -18.347509 | -53.341434 | 150.789793 | -76.670717 | -70.838396 | 104.131227 | 45.808019 | 39.975699 | 74.969623 | -47.509113 | 109.963548 | 255.771567 | -30.012151 | -30.012151 | -53.341434 | -30.012151 | -30.012151 | -47.509113 | 4.981774 | -47.509113 | -35.844471 | 57.472661 | -76.670717 | 109.963548 |
| p004 | -2.040816 | -91.836735 | -91.836735 | -51.020408 | -34.693878 | 144.897959 | 95.918367 | -100.000000 | -26.530612 | 6.122449 | -42.857143 | 210.204082 | 22.448980 | 185.714286 | 14.285714 | 14.285714 | 38.775510 | -100.000000 | -51.020408 | -100.000000 | -100.000000 | 120.408163 | -91.836735 | 14.285714 | -34.693878 | -100.000000 | 169.387755 | -100.000000 | 6.122449 | 357.142857 | -18.367347 | -34.693878 | -51.020408 | 14.285714 | 22.448980 | 63.265306 | -26.530612 | 79.591837 | 46.938776 | -26.530612 | 6.122449 | -34.693878 | -34.693878 | -91.836735 | -100.000000 | -42.857143 | 46.938776 | -100.000000 |
| p005 | 290.921228 | -10.280374 | 8.945260 | -61.548732 | -29.506008 | 8.945260 | 53.805073 | -48.731642 | 8.945260 | -10.280374 | 8.945260 | -29.506008 | 85.847797 | 21.762350 | -67.957276 | -29.506008 | -23.097463 | -16.688919 | -61.548732 | 28.170895 | -55.140187 | -100.000000 | 60.213618 | -93.591455 | 15.353805 | -100.000000 | 85.847797 | 53.805073 | -67.957276 | 233.244326 | -48.731642 | 8.945260 | 28.170895 | -16.688919 | 105.073431 | -42.323097 | -42.323097 | 98.664887 | -100.000000 | -100.000000 | -48.731642 | -16.688919 | 15.353805 | 8.945260 | -55.140187 | -48.731642 | 8.945260 | 85.847797 |

## Combining The AU assigments and the tile metrics¶

In [34]:

```
def writeMetric(metricDf,facePic,p,picDict=picDict,thisCoord=thisCoord):
    
    # names of action units 
    auList = picDict[facePic]['auList'] 

    # transforming the coordinates to indices from 0 to 47
    cCodes = {}
    for c,coord in enumerate(thisCoord):
        cCodes[ c ] = coord
    
    # dict to write to
    auDict = {float(np.nan):[]}
    
    # tracker for tiles belonging to no au
    notNan = []
    
    for au in auList:
        
        auDict[au] = []
        
        # mapping of coordinates to AUs
        d = makeBinAUs( makeAUTable(auList) ).to_dict()
        
        # looping through the metrics
        for key in metricDf.ix[p].to_dict():
            thisCoord = d[au][cCodes[int(key)]]
            # get the metric
            thisMetric = metricDf.ix[p].to_dict()[key]
            if d[au][tuple(cCodes[int(key)])] == 1:
                # adding value to 
                auDict[au].append( thisMetric)
                
                # keep track that this is not a nan
                notNan.append(key)

    # do that for the remaining nans
    for key in metricDf.ix[p].to_dict():
        if key not in notNan:
            thisCoord = d[au][cCodes[int(key)]]
            # get the metric
            thisMetric = metricDf.ix[p].to_dict()[key]
            # adding value to 
            auDict[float(np.nan)].append( thisMetric)

    # transform to df
    auDf = pd.DataFrame(index=[p])

    for entry in auDict:
        auDf[entry] = np.mean(auDict[entry])

    # cleaning up columns names
    cleanCols = []
    for e in auDf.columns:
        if type(e) == str:
            cleanCols.append(e[e.rfind('_')+1:e.rfind('.')] )
        else:
            cleanCols.append(e)

    auDf.columns = cleanCols
    return auDf
```

Example:

In [35]:

```
writeMetric( getWeight(metricDf,0,3),
            'f_fea',
            'p001'
           )
```

Out[35]:

|  | nan | AU2 | AU1 | AU20+25 | AU5 |
| --- | --- | --- | --- | --- | --- |
| p001 | -10.003494 | -1.805054 | -26.353791 | 15.523466 | 125.270758 |

### Do this for all participants¶

In [36]:

```
def writeAllMetrics(metricDf,ident,emo,picList=picList,picDict=picDict):

    facePic = picList[ident][emo]
    faceName = facePic[facePic.rfind('/')+1:facePic.rfind('_')]
    thisMetric = getWeight(metricDf,ident,emo)

    bigDf = pd.DataFrame()
    for p in thisMetric.index:
        thisDf = writeMetric(thisMetric,faceName,p,picDict=picDict)
        bigDf = pd.concat([bigDf,thisDf])
    
    return bigDf,faceName
```

Example: fearful female face (0,3)

In [37]:

```
fFeaDf,dummy = writeAllMetrics(metricDf,0,3)
```

In [38]:

```
fFeaDf.head()
```

Out[38]:

|  | nan | AU2 | AU1 | AU20+25 | AU5 |
| --- | --- | --- | --- | --- | --- |
| p001 | -10.003494 | -1.805054 | -26.353791 | 15.523466 | 125.270758 |
| p002 | -9.201378 | -3.689320 | -13.786408 | -3.689320 | 157.864078 |
| p003 | -11.520737 | 37.142857 | 105.714286 | -46.666667 | 151.428571 |
| p004 | -11.966701 | -22.580645 | 16.129032 | 56.989247 | -35.483871 |
| p005 | -4.403927 | -30.434783 | -47.826087 | 6.666667 | 53.043478 |

### Do this for all faces¶

In [39]:

```
def writeAllFaces(metricDf,picList=picList,picDict=picDict):
    bigDf = pd.DataFrame()
    for ident in range(2):
        for emo in range(7):
            thisDf,faceName = writeAllMetrics(metricDf,ident,emo,picList=picList,picDict=picDict)
            thisDf.columns = [ [faceName]*len(thisDf.columns),thisDf.columns]
            bigDf = pd.concat([bigDf,thisDf],axis=1)
    return bigDf
```

In [40]:

```
bigAU = writeAllFaces(metricDf)
```

In [41]:

```
bigAU.head()
```

Out[41]:

|  | f\_hap | | | | f\_sad | | | f\_ang | | | | | f\_fea | | | | | f\_dis | | | | | f\_sup | | | | | f\_ntr | | m\_hap | | | | m\_sad | | | m\_ang | | | | m\_fea | | | | | m\_dis | | | | | m\_sup | | | | | m\_ntr | |
| --- | --- | --- | --- | --- | --- | --- | --- | --- | --- | --- | --- | --- | --- | --- | --- | --- | --- | --- | --- | --- | --- | --- | --- | --- | --- | --- | --- | --- | --- | --- | --- | --- | --- | --- | --- | --- | --- | --- | --- | --- | --- | --- | --- | --- | --- | --- | --- | --- | --- | --- | --- | --- | --- | --- | --- | --- | --- |
|  | NaN | AU12 | AU25 | AU6 | NaN | AU15+25 | AU1+4 | NaN | AU9 | AU7 | AU6 | AU22+23+25 | NaN | AU2 | AU1 | AU20+25 | AU5 | NaN | AU10+16+19+25+26 | AU9 | AU6 | AU4 | NaN | AU2 | AU1 | AU25+26+27 | AU5 | NaN | AU25 | NaN | AU12 | AU25 | AU6 | NaN | AU1+4 | AU15+25 | NaN | AU25+29 | AU7 | AU4 | NaN | AU1 | AU2 | AU20+25 | AU5 | NaN | AU10+16+19+25+26 | AU4 | AU6 | AU9 | NaN | AU1 | AU2 | AU5 | AU25+26+27 | NaN | AU25 |
| p001 | 9.833972 | 7.407407 | -0.529101 | -22.222222 | -19.420671 | -2.040816 | 61.632653 | -19.467061 | 34.715026 | -8.808290 | 36.096718 | 13.989637 | -10.003494 | -1.805054 | -26.353791 | 15.523466 | 125.270758 | -25.658807 | 50.346741 | -49.514563 | -14.563107 | 25.242718 | 0.651982 | -15.418502 | -25.991189 | 14.285714 | -64.757709 | -2.699869 | 18.899083 | -21.095890 | -59.817352 | 57.077626 | 37.351598 | NaN | NaN | NaN | NaN | NaN | NaN | NaN | 17.679888 | 17.919075 | -22.312139 | -20.231214 | -44.508671 | 13.506301 | 3.943662 | -58.470825 | -18.309859 | -29.014085 | 18.692618 | -81.491003 | -52.699229 | 63.496144 | -13.624679 | -5.833042 | 40.831296 |
| p002 | 8.252983 | -41.706161 | -19.566689 | 1.232227 | -19.782355 | 1.204819 | 60.481928 | -37.074247 | 68.782161 | -9.433962 | 65.351630 | 31.732419 | -9.201378 | -3.689320 | -13.786408 | -3.689320 | 157.864078 | -41.200828 | 75.155280 | -100.000000 | -39.920949 | 30.434783 | 9.136842 | -46.947368 | -64.631579 | 11.157895 | -36.000000 | -3.454715 | 24.183007 | -24.236760 | -43.925234 | 79.439252 | 30.841121 | -14.590747 | 61.501132 | -37.366548 | -6.348282 | -6.647808 | 33.663366 | 37.482320 | 5.747126 | 64.705882 | 12.941176 | -30.392157 | 7.843137 | 3.711911 | 32.000000 | -84.120301 | -40.210526 | -100.000000 | 26.007326 | -53.846154 | -36.752137 | -25.641026 | -11.111111 | -2.570586 | 17.994100 |
| p003 | -13.922990 | 12.272175 | 21.645548 | 17.812880 | -6.571087 | -8.994709 | 26.666667 | -28.035105 | 21.843003 | -1.706485 | 22.866894 | 63.822526 | -11.520737 | 37.142857 | 105.714286 | -46.666667 | 151.428571 | -43.111111 | 49.333333 | -4.000000 | 37.696970 | 4.000000 | -14.233503 | -0.507614 | 11.675127 | 10.224801 | 48.900169 | -10.379165 | 72.654155 | 1.768707 | 78.231293 | 35.147392 | -29.251701 | 3.225806 | -27.272727 | 33.333333 | -19.191919 | 26.984127 | 85.858586 | 66.233766 | 12.551724 | 63.200000 | -46.240000 | -23.200000 | -42.400000 | -1.999833 | -17.965024 | 50.442880 | 10.015898 | -16.057234 | -31.506849 | -26.027397 | -8.219178 | 4.794521 | 48.493151 | -9.407666 | 65.853659 |
| p004 | -22.026742 | 65.306122 | 92.419825 | -0.408163 | -9.136566 | 43.712575 | -2.275449 | -8.777084 | -2.056555 | 57.326478 | 7.969152 | 18.766067 | -11.966701 | -22.580645 | 16.129032 | 56.989247 | -35.483871 | -33.445378 | 49.747899 | -36.470588 | -1.176471 | -2.941176 | -22.292467 | 13.441654 | 27.621861 | 33.192657 | -14.918759 | 7.711757 | -53.982301 | -7.976879 | -16.763006 | 19.075145 | 8.208092 | NaN | NaN | NaN | -20.204604 | 26.708075 | 173.913043 | 71.428571 | -3.532896 | -70.552147 | -52.883436 | 28.834356 | 69.325153 | 6.567303 | -16.371681 | 9.228824 | 9.144543 | 9.734513 | 4.310652 | 4.738155 | -16.209476 | 19.700748 | -0.249377 | -5.980066 | 41.860465 |
| p005 | -14.258091 | 40.987984 | 28.170895 | 17.276368 | 19.106700 | 5.494505 | -63.076923 | -3.970528 | -33.810888 | -34.670487 | -23.209169 | 48.710602 | -4.403927 | -30.434783 | -47.826087 | 6.666667 | 53.043478 | -14.235145 | 50.493047 | -21.415929 | -28.173773 | -40.530973 | -17.338552 | 64.383562 | 62.035225 | 20.771596 | -46.771037 | 0.348432 | -2.439024 | 0.295791 | -24.914676 | 11.035267 | -0.068259 | -15.542522 | -20.661157 | 118.181818 | -13.459621 | 14.285714 | 69.491525 | 51.089588 | -27.893479 | 60.396040 | 18.811881 | 6.930693 | 107.920792 | -2.416987 | 23.605150 | -41.140405 | -21.888412 | -48.497854 | -21.951220 | 93.902439 | 34.146341 | -74.390244 | 16.097561 | -5.946685 | 41.626794 |

### Taking the nan (no Action Unit) as baseline¶

In [42]:

```
def baselineCorrection(df,cond):
    # select the condition
    thisCorr = df[cond]
    diffDf = pd.DataFrame()

    # for all action units
    for actionUnit in thisCorr.columns:
        # we subtract the baseline
        thisDiff = thisCorr[actionUnit]-thisCorr[np.nan]
        diffDf[actionUnit] = thisDiff
    diffDf = diffDf.drop(np.nan,axis=1)
    
    # restore the original structure of the multicolumns
    diffDf.columns = [[cond]*len(diffDf.columns),diffDf.columns]
    
    return diffDf
```

Example:

In [43]:

```
bigAU['f_fea'].head()
```

Out[43]:

|  | nan | AU2 | AU1 | AU20+25 | AU5 |
| --- | --- | --- | --- | --- | --- |
| p001 | -10.003494 | -1.805054 | -26.353791 | 15.523466 | 125.270758 |
| p002 | -9.201378 | -3.689320 | -13.786408 | -3.689320 | 157.864078 |
| p003 | -11.520737 | 37.142857 | 105.714286 | -46.666667 | 151.428571 |
| p004 | -11.966701 | -22.580645 | 16.129032 | 56.989247 | -35.483871 |
| p005 | -4.403927 | -30.434783 | -47.826087 | 6.666667 | 53.043478 |

In [44]:

```
baselineCorrection(bigAU,'f_fea').head()
```

Out[44]:

|  | f\_fea | | | |
| --- | --- | --- | --- | --- |
|  | AU2 | AU1 | AU20+25 | AU5 |
| p001 | 8.198440 | -16.350297 | 25.526959 | 135.274252 |
| p002 | 5.512058 | -4.585030 | 5.512058 | 167.065456 |
| p003 | 48.663594 | 117.235023 | -35.145929 | 162.949309 |
| p004 | -10.613944 | 28.095734 | 68.955949 | -23.517170 |
| p005 | -26.030856 | -43.422160 | 11.070594 | 57.447405 |

### Do this for the whole big table¶

In [45]:

```
def makeBaseline(bigAU):
    baselineDf = pd.DataFrame()
    for face in bigAU.columns.levels[0]:
        thisDf = baselineCorrection(bigAU,face)
        baselineDf = pd.concat([baselineDf,thisDf],axis=1)
    
    return baselineDf
```

In [46]:

```
baselineDf = makeBaseline(bigAU)
```

Example:

In [47]:

```
baselineDf['f_fea'].head()
```

Out[47]:

|  | AU2 | AU1 | AU20+25 | AU5 |
| --- | --- | --- | --- | --- |
| p001 | 8.198440 | -16.350297 | 25.526959 | 135.274252 |
| p002 | 5.512058 | -4.585030 | 5.512058 | 167.065456 |
| p003 | 48.663594 | 117.235023 | -35.145929 | 162.949309 |
| p004 | -10.613944 | 28.095734 | 68.955949 | -23.517170 |
| p005 | -26.030856 | -43.422160 | 11.070594 | 57.447405 |

In [48]:

```
baselineDf.to_csv('../outputs/actionUnitTable.csv')
```

### Example of re-loading¶

In [49]:

```
baselineDf = pd.read_csv('../outputs/actionUnitTable.csv',index_col=[0],header=[0,1])
```

In [50]:

```
baselineDf.head()
```

Out[50]:

|  | f\_ang | | | | f\_dis | | | | f\_fea | | | | f\_hap | | | f\_ntr | f\_sad | | f\_sup | | | | m\_ang | | | m\_dis | | | | m\_fea | | | | m\_hap | | | m\_ntr | m\_sad | | m\_sup | | | |
| --- | --- | --- | --- | --- | --- | --- | --- | --- | --- | --- | --- | --- | --- | --- | --- | --- | --- | --- | --- | --- | --- | --- | --- | --- | --- | --- | --- | --- | --- | --- | --- | --- | --- | --- | --- | --- | --- | --- | --- | --- | --- | --- | --- |
|  | AU9 | AU7 | AU6 | AU22+23+25 | AU10+16+19+25+26 | AU9 | AU6 | AU4 | AU2 | AU1 | AU20+25 | AU5 | AU12 | AU25 | AU6 | AU25 | AU15+25 | AU1+4 | AU2 | AU1 | AU25+26+27 | AU5 | AU25+29 | AU7 | AU4 | AU10+16+19+25+26 | AU4 | AU6 | AU9 | AU1 | AU2 | AU20+25 | AU5 | AU12 | AU25 | AU6 | AU25 | AU1+4 | AU15+25 | AU1 | AU2 | AU5 | AU25+26+27 |
| p001 | 54.182087 | 10.658771 | 55.563780 | 33.456699 | 76.005548 | -23.855756 | 11.095700 | 50.901526 | 8.198440 | -16.350297 | 25.526959 | 135.274252 | -2.426564 | -10.363072 | -32.056194 | 21.598952 | 17.379855 | 81.053325 | -16.070485 | -26.643172 | 13.633732 | -65.409692 | NaN | NaN | NaN | -9.562639 | -71.977126 | -31.816160 | -42.520385 | 0.239187 | -39.992027 | -37.911102 | -62.188559 | -38.721461 | 78.173516 | 58.447489 | 46.664338 | NaN | NaN | -100.183621 | -71.391847 | 44.803526 | -32.317297 |
| p002 | 105.856408 | 27.640284 | 102.425876 | 68.806665 | 116.356108 | -58.799172 | 1.279880 | 71.635611 | 5.512058 | -4.585030 | 5.512058 | 167.065456 | -49.959144 | -27.819672 | -7.020755 | 27.637722 | 20.987175 | 80.264283 | -56.084211 | -73.768421 | 2.021053 | -45.136842 | -0.299526 | 40.011648 | 43.830602 | 28.288089 | -87.832212 | -43.922438 | -103.711911 | 58.958756 | 7.194050 | -36.139283 | 2.096011 | -19.688474 | 103.676012 | 55.077882 | 20.564686 | 76.091880 | -22.775801 | -79.853480 | -62.759463 | -51.648352 | -37.118437 |
| p003 | 49.878108 | 26.328620 | 50.901999 | 91.857630 | 92.444444 | 39.111111 | 80.808081 | 47.111111 | 48.663594 | 117.235023 | -35.145929 | 162.949309 | 26.195165 | 35.568538 | 31.735870 | 83.033321 | -2.423622 | 33.237754 | 13.725888 | 25.908629 | 24.458303 | 63.133672 | 46.176046 | 105.050505 | 85.425685 | -15.965191 | 52.442713 | 12.015731 | -14.057401 | 50.648276 | -58.791724 | -35.751724 | -54.951724 | 76.462585 | 33.378685 | -31.020408 | 75.261324 | -30.498534 | 30.107527 | 5.479452 | 23.287671 | 36.301370 | 80.000000 |
| p004 | 6.720529 | 66.103562 | 16.746236 | 27.543151 | 83.193277 | -3.025210 | 32.268908 | 30.504202 | -10.613944 | 28.095734 | 68.955949 | -23.517170 | 87.332864 | 114.446567 | 21.618578 | -61.694058 | 52.849140 | 6.861116 | 35.734121 | 49.914328 | 55.485123 | 7.373708 | 46.912678 | 194.117647 | 91.633175 | -22.938985 | 2.661521 | 2.577240 | 3.167210 | -67.019251 | -49.350539 | 32.367252 | 72.858050 | -8.786127 | 27.052023 | 16.184971 | 47.840532 | NaN | NaN | 0.427503 | -20.520128 | 15.390096 | -4.560029 |
| p005 | -29.840360 | -30.699959 | -19.238641 | 52.681130 | 64.728192 | -7.180784 | -13.938628 | -26.295828 | -26.030856 | -43.422160 | 11.070594 | 57.447405 | 55.246075 | 42.428986 | 31.534460 | -2.787456 | -13.612194 | -82.183623 | 81.722114 | 79.373777 | 38.110148 | -29.432485 | 27.745335 | 82.951147 | 64.549210 | 26.022137 | -38.723418 | -19.471425 | -46.080867 | 88.289519 | 46.705360 | 34.824172 | 135.814271 | -25.210466 | 10.739477 | -0.364050 | 47.573479 | -5.118635 | 133.724340 | 115.853659 | 56.097561 | -52.439024 | 38.048780 |

## Basic plotting¶

In [51]:

```
def makeActionUnitPlot(baselineDf,face):
    # select the face condition
    thisDf = baselineDf[face]
    
    colNames = thisDf.columns
    # get number of participants (with values)
    n = int(thisDf.describe().ix['count'][-1])
    
    # get all metrics into a df which we sort descending
    plotDf = pd.DataFrame()
    plotDf['mean'] = thisDf.mean()
    #print float( thisDf.mean() ), float( thisDf.describe().ix['mean'] )
    plotDf['ci'] = thisDf.std()/np.sqrt(n)*1.96
 
    plotDf['color'] = [rgb2hex(getColor('../auLabels/'+face+'_'+colName+'.png')) for colName in colNames]
    plotDf = plotDf.sort_values(by="mean",ascending=False)
   
    # plot this bar at the correct position and using the correct color
    # this is done in a 
    plt.xticks(np.arange(len(plotDf.index))+0.45,plotDf.index,rotation=45)
    plt.xlim(-0.05,4.05)
    plt.yticks(range(0,101,20),['0%','20%','40%','60%','80%','100%'])
    plt.ylim(-10,100)
    plt.axhline(0,color='k')
    plt.title(face,fontsize=20)
    im = plt.bar(range(len(plotDf.index)) ,
            plotDf['mean'],
            yerr=plotDf['ci'],
            color= plotDf['color'],
            ecolor='k')
    
    return im
```

In [52]:

```
makeActionUnitPlot(baselineDf,'f_sad');
```

In [53]:

```
fig = plt.figure(figsize=(14,28))
count = 1
for pic in picSort:
    if 'ntr' not in pic:
        ax = plt.subplot(4,3,count)
        ax = makeActionUnitPlot(baselineDf,pic)
        plt.ylabel('Percent Signal Change')
        count+=1
        sns.despine()
plt.tight_layout()
plt.savefig('../figures/allAUs.png',dpi=300)
plt.show()
```

# 4b. Plotting Results by Face Half¶

This basically re-uses the scripts above, but there are only two regions and together they make up the whole face. Therfore, a difference score can be computed which summarizes for each face whether the upper face half (positive values) or the lower face half (negative values) is more important.

### get the up/low mask images¶

In [54]:

```
myPicList = getFile('../auLabels/','*er.png')
```

In [55]:

```
myPicList
```

Out[55]:

```
['../auLabels/lower.png', '../auLabels/upper.png']
```

### assign tiles to upper/lower half¶

In [56]:

```
makeBinAUs( makeAUTable(myPicList) ).head()
```

Out[56]:

|  |  | ../auLabels/lower.png | ../auLabels/upper.png |
| --- | --- | --- | --- |
| 0 | 0 | 0 | 1 |
|  | 50 | 0 | 1 |
|  | 100 | 0 | 1 |
|  | 150 | 0 | 1 |
|  | 200 | 1 | 0 |

In [57]:

```
d = makeBinAUs( makeAUTable(myPicList) ).to_dict()
```

### here, each face has the same two masks¶

In [58]:

```
def makePicDict(picList,myPicList):
    
    picDict = {}
    for i,ident in enumerate(picList):
        for e,emo in enumerate(picList[i]):
            thisPic = picList[i][e]

            thisName = thisPic[thisPic.find('_')-1:thisPic.rfind('_')]

            picDict[thisName] = {'auList':myPicList,
                                 'picFile':thisPic}
    return picDict
```

In [59]:

```
picDict = makePicDict(picList,myPicList)
```

In [60]:

```
picDict
```

Out[60]:

```
{'f_ang': {'auList': ['../auLabels/lower.png', '../auLabels/upper.png'],
  'picFile': '../experiment/app/static/img/f_ang_cut.png'},
 'f_dis': {'auList': ['../auLabels/lower.png', '../auLabels/upper.png'],
  'picFile': '../experiment/app/static/img/f_dis_cut.png'},
 'f_fea': {'auList': ['../auLabels/lower.png', '../auLabels/upper.png'],
  'picFile': '../experiment/app/static/img/f_fea_cut.png'},
 'f_hap': {'auList': ['../auLabels/lower.png', '../auLabels/upper.png'],
  'picFile': '../experiment/app/static/img/f_hap_cut.png'},
 'f_ntr': {'auList': ['../auLabels/lower.png', '../auLabels/upper.png'],
  'picFile': '../experiment/app/static/img/f_ntr_cut.png'},
 'f_sad': {'auList': ['../auLabels/lower.png', '../auLabels/upper.png'],
  'picFile': '../experiment/app/static/img/f_sad_cut.png'},
 'f_sup': {'auList': ['../auLabels/lower.png', '../auLabels/upper.png'],
  'picFile': '../experiment/app/static/img/f_sup_cut.png'},
 'm_ang': {'auList': ['../auLabels/lower.png', '../auLabels/upper.png'],
  'picFile': '../experiment/app/static/img/m_ang_cut.png'},
 'm_dis': {'auList': ['../auLabels/lower.png', '../auLabels/upper.png'],
  'picFile': '../experiment/app/static/img/m_dis_cut.png'},
 'm_fea': {'auList': ['../auLabels/lower.png', '../auLabels/upper.png'],
  'picFile': '../experiment/app/static/img/m_fea_cut.png'},
 'm_hap': {'auList': ['../auLabels/lower.png', '../auLabels/upper.png'],
  'picFile': '../experiment/app/static/img/m_hap_cut.png'},
 'm_ntr': {'auList': ['../auLabels/lower.png', '../auLabels/upper.png'],
  'picFile': '../experiment/app/static/img/m_ntr_cut.png'},
 'm_sad': {'auList': ['../auLabels/lower.png', '../auLabels/upper.png'],
  'picFile': '../experiment/app/static/img/m_sad_cut.png'},
 'm_sup': {'auList': ['../auLabels/lower.png', '../auLabels/upper.png'],
  'picFile': '../experiment/app/static/img/m_sup_cut.png'}}
```

### illustrate the masking¶

In [61]:

```
for entry in picSort:
    
    d = makeBinAUs( makeAUTable(picDict[entry]['auList'])).to_dict()
    imDict = showTiles(picDict[entry]['picFile'],d)

    fig = plt.figure(figsize=(12,6))
    for i,im in enumerate(imDict):
        ax = plt.subplot( 1, 2, i+1 )
        imName = im[im.rfind('_')+1:im.rfind('.')]
        ax.set_title(imName[imName.rfind('/')+1:])
        ax.set_xticks([]);ax.set_yticks([])
        ax = plt.imshow(imDict[im])

    plt.savefig('../figures/auAssignments/upDownExample.png',dpi=300)
    plt.show()
    break # do this only for the first image
```

### get the metric for each half¶

Here, there is no nan, because each tile must belong to one of the two masks

Example:

In [62]:

```
writeMetric( getWeight(metricDf,0,3),
            'f_fea',
            'p001',
            picDict=picDict
           )
```

```
/opt/anaconda2/lib/python2.7/site-packages/numpy/core/_methods.py:59: RuntimeWarning: Mean of empty slice.
  warnings.warn("Mean of empty slice.", RuntimeWarning)
```

Out[62]:

|  | nan | ../auLabels/lower | ../auLabels/upper |
| --- | --- | --- | --- |
| p001 | NaN | -1.805054 | 1.805054 |

### get the metric for all faces and all participants¶

In [63]:

```
bigUpperLower = writeAllFaces(metricDf,picDict=picDict)
```

In [64]:

```
bigUpperLower.head()
```

Out[64]:

|  | f\_hap | | | f\_sad | | | f\_ang | | | f\_fea | | | f\_dis | | | f\_sup | | | f\_ntr | | | m\_hap | | | m\_sad | | | m\_ang | | | m\_fea | | | m\_dis | | | m\_sup | | | m\_ntr | | |
| --- | --- | --- | --- | --- | --- | --- | --- | --- | --- | --- | --- | --- | --- | --- | --- | --- | --- | --- | --- | --- | --- | --- | --- | --- | --- | --- | --- | --- | --- | --- | --- | --- | --- | --- | --- | --- | --- | --- | --- | --- | --- | --- |
|  | NaN | ../auLabels/lower | ../auLabels/upper | NaN | ../auLabels/lower | ../auLabels/upper | NaN | ../auLabels/lower | ../auLabels/upper | NaN | ../auLabels/lower | ../auLabels/upper | NaN | ../auLabels/lower | ../auLabels/upper | NaN | ../auLabels/lower | ../auLabels/upper | NaN | ../auLabels/lower | ../auLabels/upper | NaN | ../auLabels/lower | ../auLabels/upper | NaN | ../auLabels/lower | ../auLabels/upper | NaN | ../auLabels/lower | ../auLabels/upper | NaN | ../auLabels/lower | ../auLabels/upper | NaN | ../auLabels/lower | ../auLabels/upper | NaN | ../auLabels/lower | ../auLabels/upper | NaN | ../auLabels/lower | ../auLabels/upper |
| p001 | NaN | -2.469136 | 2.469136 | NaN | -9.523810 | 9.523810 | NaN | 5.699482 | -5.699482 | NaN | -1.805054 | 1.805054 | NaN | 14.886731 | -14.886731 | NaN | 7.488987 | -7.488987 | NaN | -4.954128 | 4.954128 | NaN | -8.675799 | 8.675799 | NaN | NaN | NaN | NaN | NaN | NaN | NaN | -17.919075 | 17.919075 | NaN | 7.323944 | -7.323944 | NaN | -4.884319 | 4.884319 | NaN | 3.667482 | -3.667482 |
| p002 | NaN | -13.744076 | 13.744076 | NaN | -7.228916 | 7.228916 | NaN | 7.718696 | -7.718696 | NaN | 7.961165 | -7.961165 | NaN | 28.260870 | -28.260870 | NaN | 7.789474 | -7.789474 | NaN | 5.555556 | -5.555556 | NaN | -2.180685 | 2.180685 | NaN | -25.266904 | 25.266904 | NaN | 3.465347 | -3.465347 | NaN | -15.359477 | 15.359477 | NaN | 22.105263 | -22.105263 | NaN | -1.282051 | 1.282051 | NaN | -15.044248 | 15.044248 |
| p003 | NaN | 3.280680 | -3.280680 | NaN | -2.469136 | 2.469136 | NaN | 9.215017 | -9.215017 | NaN | 2.857143 | -2.857143 | NaN | 16.444444 | -16.444444 | NaN | 8.967851 | -8.967851 | NaN | 6.970509 | -6.970509 | NaN | 2.947846 | -2.947846 | NaN | 33.333333 | -33.333333 | NaN | 13.131313 | -13.131313 | NaN | -20.000000 | 20.000000 | NaN | -12.559618 | 12.559618 | NaN | 16.438356 | -16.438356 | NaN | 15.447154 | -15.447154 |
| p004 | NaN | 19.727891 | -19.727891 | NaN | -5.988024 | 5.988024 | NaN | 1.028278 | -1.028278 | NaN | 14.516129 | -14.516129 | NaN | 10.294118 | -10.294118 | NaN | 14.918759 | -14.918759 | NaN | -13.274336 | 13.274336 | NaN | 19.653179 | -19.653179 | NaN | NaN | NaN | NaN | -10.144928 | 10.144928 | NaN | 0.613497 | -0.613497 | NaN | -14.749263 | 14.749263 | NaN | 10.723192 | -10.723192 | NaN | -5.232558 | 5.232558 |
| p005 | NaN | -9.212283 | 9.212283 | NaN | -38.461538 | 38.461538 | NaN | 5.157593 | -5.157593 | NaN | -30.434783 | 30.434783 | NaN | 21.769912 | -21.769912 | NaN | 8.806262 | -8.806262 | NaN | -12.195122 | 12.195122 | NaN | -5.119454 | 5.119454 | NaN | -27.272727 | 27.272727 | NaN | 9.039548 | -9.039548 | NaN | -16.831683 | 16.831683 | NaN | 4.291845 | -4.291845 | NaN | 4.878049 | -4.878049 | NaN | 23.444976 | -23.444976 |

### compute a difference score up minus down¶

In [65]:

```
def makeDiffDf(bigUpperLower):
    bigDiff = pd.DataFrame()
    for entry in bigUpperLower.columns.levels[0]:
        thisDiff = pd.DataFrame( bigUpperLower[entry]['../auLabels/upper']- bigUpperLower[entry]['../auLabels/lower'] )
        thisDiff.columns = [ [entry[0]] , [entry[2:]] ]
        bigDiff = pd.concat([bigDiff,thisDiff],axis=1)
    return bigDiff
```

In [66]:

```
bigDiff = makeDiffDf(bigUpperLower)
```

Example:

In [67]:

```
bigDiff.head()
```

Out[67]:

|  | f | | | | | | | m | | | | | | |
| --- | --- | --- | --- | --- | --- | --- | --- | --- | --- | --- | --- | --- | --- | --- |
|  | ang | dis | fea | hap | ntr | sad | sup | ang | dis | fea | hap | ntr | sad | sup |
| p001 | -11.398964 | -29.773463 | 3.610108 | 4.938272 | 9.908257 | 19.047619 | -14.977974 | NaN | -14.647887 | 35.838150 | 17.351598 | -7.334963 | NaN | 9.768638 |
| p002 | -15.437393 | -56.521739 | -15.922330 | 27.488152 | -11.111111 | 14.457831 | -15.578947 | -6.930693 | -44.210526 | 30.718954 | 4.361371 | 30.088496 | 50.533808 | 2.564103 |
| p003 | -18.430034 | -32.888889 | -5.714286 | -6.561361 | -13.941019 | 4.938272 | -17.935702 | -26.262626 | 25.119237 | 40.000000 | -5.895692 | -30.894309 | -66.666667 | -32.876712 |
| p004 | -2.056555 | -20.588235 | -29.032258 | -39.455782 | 26.548673 | 11.976048 | -29.837518 | 20.289855 | 29.498525 | -1.226994 | -39.306358 | 10.465116 | NaN | -21.446384 |
| p005 | -10.315186 | -43.539823 | 60.869565 | 18.424566 | 24.390244 | 76.923077 | -17.612524 | -18.079096 | -8.583691 | 33.663366 | 10.238908 | -46.889952 | 54.545455 | -9.756098 |

## Make a figure¶

In [68]:

```
sns.set_style('white')
sns.palplot(stackColors)
```

In [69]:

```
def makeUpLowPlot(bigDiff,emoReverse=emoReverse,stackColors=stackColors):
    fig = plt.figure(figsize=(16,6))
    ax = plt.subplot(1,2,1)
    # hard-code the order to please us
    myOrder=['dis','sup','hap','ang','ntr','fea','sad']
    for e,entry in enumerate(myOrder):
        thisCond = bigDiff['f'][entry]

        ax.bar([e],
                 thisCond.mean(),
                 color=stackColors[emoReverse[entry]],
                 yerr=thisCond.std()/np.sqrt(len(thisCond))*1.96,
                 ecolor='k'
                )
    ax.set_xlim(-0.1,7.1)
    ax.set_xticks(np.arange(len(myOrder))+0.4)
    ax.set_xticklabels(myOrder );
    ax.axhline(0,color='k')
    ax.set_ylabel('Upper-Lower % Difference')
    ax.set_xlabel('emotion expression')
    ax.set_title('Female Face')
    ax.set_ylim(-33,30)
    sns.despine()

    ax = plt.subplot(1,2,2)
    myOrder=['dis','hap','ntr','sup','fea','sad','ang']
    for e,entry in enumerate(myOrder):
        thisCond = bigDiff['m'][entry]

        ax.bar([e],
                 thisCond.mean(),
                 color=stackColors[emoReverse[entry]],
                 yerr=thisCond.std()/np.sqrt(len(thisCond))*1.96,
                 ecolor='k'
                )
    ax.set_xlim(-0.1,7.1)
    ax.set_xticks(np.arange(len(myOrder))+0.4)
    ax.set_xticklabels(myOrder );
    ax.axhline(0,color='k')
    ax.set_ylabel('Upper-Lower % Difference')
    ax.set_xlabel('emotion expression')
    ax.set_title('Male Face')
    ax.set_ylim(-33,30)
    sns.despine()
    
    plt.savefig('../figures/upDownPlot.png',dpi=300)
    plt.show()
```

In [70]:

```
makeUpLowPlot(bigDiff)
```

In [ ]:

```

```
