# Supplementary material for: Mapping the emotional face. How individual face parts contribute to successful emotion recognition
Source: PLoS One. 2017 May 11;12(5):e0177239. doi: 10.1371/journal.pone.0177239 (PMC5426715; doi:10.1371/journal.pone.0177239)
Supplement: S6 Code — (HTML) [file pone.0177239.s008.html]

code005\_principalComponentAnalysis


# Mapping the emotional face. How individual face parts contribute to successful emotion recognition.

# 5. Principal Component Analysis (PCA)¶

### Import modules¶

In [1]:

```
from myBasics import *
%matplotlib inline

from sklearn.decomposition import PCA as sklearnPCA
```

### get data¶

Get the big table, with values of each participant

In [2]:

```
participantDf = pd.read_csv('../outputs/weightStdDf.csv',index_col=[0,1,2])
participantDf.index.names = ['p','ident','emo']
```

In [3]:

```
participantDf.head()
```

Out[3]:

|  |  |  | 0 | 1 | 2 | 3 | 4 | 5 | 6 | 7 | 8 | 9 | 10 | 11 | 12 | 13 | 14 | 15 | 16 | 17 | 18 | 19 | 20 | 21 | 22 | 23 | 24 | 25 | 26 | 27 | 28 | 29 | 30 | 31 | 32 | 33 | 34 | 35 | 36 | 37 | 38 | 39 | 40 | 41 | 42 | 43 | 44 | 45 | 46 | 47 |
| --- | --- | --- | --- | --- | --- | --- | --- | --- | --- | --- | --- | --- | --- | --- | --- | --- | --- | --- | --- | --- | --- | --- | --- | --- | --- | --- | --- | --- | --- | --- | --- | --- | --- | --- | --- | --- | --- | --- | --- | --- | --- | --- | --- | --- | --- | --- | --- | --- | --- | --- |
| p | ident | emo |  |  |  |  |  |  |  |  |  |  |  |  |  |  |  |  |  |  |  |  |  |  |  |  |  |  |  |  |  |  |  |  |  |  |  |  |  |  |  |  |  |  |  |  |  |  |  |  |
| p001 | f | ang | -0.948401 | -0.006541 | 1.667878 | -1.262355 | -1.262355 | -0.320494 | -0.948401 | 0.412064 | -1.262355 | -0.948401 | 1.039971 | 1.458576 | -1.262355 | 0.098110 | 0.935320 | -1.262355 | -0.948401 | 0.412064 | -1.262355 | 0.412064 | 1.039971 | -1.262355 | -0.006541 | -0.320494 | -0.320494 | -0.843750 | 2.400437 | 0.412064 | 1.353925 | 0.412064 | 1.458576 | -0.006541 | -1.262355 | -1.262355 | -1.262355 | 0.412064 | 1.039971 | 0.098110 | -0.320494 | -0.320494 | 0.098110 | 0.098110 | 0.098110 | 1.353925 | -1.262355 | 1.039971 | 1.353925 | 1.039971 |
|  |  | dis | -0.245679 | 1.036125 | -0.074772 | -1.100216 | -1.100216 | 0.437950 | 1.548847 | 0.865218 | -0.245679 | -1.100216 | 1.121579 | -1.100216 | 0.865218 | 0.779764 | 0.608857 | -1.100216 | 0.608857 | -0.587494 | -0.074772 | -1.100216 | -1.100216 | 1.805208 | 1.890662 | 0.437950 | -0.672947 | 0.352496 | 0.096135 | -1.100216 | -0.074772 | -1.100216 | 3.257920 | -0.587494 | 0.523404 | -0.074772 | 1.292486 | 0.352496 | -1.100216 | -1.100216 | 1.463394 | -0.074772 | -1.100216 | -0.587494 | -0.074772 | -0.074772 | -0.587494 | -0.587494 | -1.100216 | -0.416586 |
|  |  | fea | 0.582234 | -1.040508 | -1.040508 | -1.040508 | 0.401929 | -1.040508 | 0.401929 | 1.844366 | 0.401929 | 0.582234 | 1.844366 | 0.582234 | 0.221624 | 0.582234 | 1.664062 | 0.582234 | 0.582234 | -1.040508 | 0.221624 | 0.582234 | -1.040508 | 1.123148 | 0.221624 | -1.040508 | -1.040508 | -1.040508 | 0.221624 | 0.401929 | 0.221624 | 2.024671 | -1.040508 | -1.040508 | -1.040508 | 0.401929 | 1.844366 | 0.582234 | -1.040508 | -1.040508 | -1.040508 | -1.040508 | -1.040508 | 2.024671 | -1.040508 | -1.040508 | 0.221624 | -1.040508 | 0.221624 | 0.221624 |
|  |  | hap | 0.352580 | 1.057741 | -0.151106 | -0.856267 | -0.352580 | -1.359953 | 1.460690 | -0.453318 | -1.359953 | 1.460690 | 0.755529 | -0.352580 | 0.554055 | -0.554055 | -0.856267 | -0.654792 | -0.050369 | 0.151106 | -1.359953 | -0.957004 | 0.251843 | 1.057741 | -0.957004 | -0.957004 | -0.453318 | 0.251843 | -0.755529 | -0.352580 | 0.050369 | 0.251843 | -0.755529 | -1.259216 | 1.158478 | 0.151106 | 0.554055 | 1.762902 | -1.359953 | 1.762902 | -0.352580 | 1.158478 | -0.352580 | 2.065113 | -0.554055 | -1.359953 | 1.863639 | -1.359953 | 0.251843 | 1.762902 |
|  |  | ntr | -0.897600 | -0.897600 | 0.157930 | 0.733673 | 0.349844 | -1.089514 | -1.089514 | -0.801643 | -1.089514 | -0.321856 | 1.213459 | -0.321856 | 0.157930 | 0.733673 | 0.157930 | 0.157930 | -0.897600 | -0.609728 | 0.733673 | -1.089514 | -1.089514 | -0.513771 | 0.061972 | -0.321856 | 0.445801 | -0.033985 | 0.157930 | -1.089514 | -0.705685 | 0.445801 | 0.349844 | 2.077074 | 0.541758 | 1.981117 | 0.829630 | -1.089514 | -0.705685 | -0.321856 | 0.829630 | -1.089514 | 0.157930 | -1.089514 | 3.708346 | 0.061972 | -0.417814 | -0.705685 | 1.981117 | 0.253887 |

Get the averaged data

In [4]:

```
weightDf = pd.read_csv('../outputs/weightAvgStdDf.csv',
                      index_col=[0,1])
weightDf.index.names = ['ident','emo']
weightDf = weightDf.sortlevel()
```

In [5]:

```
weightDf.head()
```

Out[5]:

|  |  | 0 | 1 | 2 | 3 | 4 | 5 | 6 | 7 | 8 | 9 | 10 | 11 | 12 | 13 | 14 | 15 | 16 | 17 | 18 | 19 | 20 | 21 | 22 | 23 | 24 | 25 | 26 | 27 | 28 | 29 | 30 | 31 | 32 | 33 | 34 | 35 | 36 | 37 | 38 | 39 | 40 | 41 | 42 | 43 | 44 | 45 | 46 | 47 |
| --- | --- | --- | --- | --- | --- | --- | --- | --- | --- | --- | --- | --- | --- | --- | --- | --- | --- | --- | --- | --- | --- | --- | --- | --- | --- | --- | --- | --- | --- | --- | --- | --- | --- | --- | --- | --- | --- | --- | --- | --- | --- | --- | --- | --- | --- | --- | --- | --- | --- |
| ident | emo |  |  |  |  |  |  |  |  |  |  |  |  |  |  |  |  |  |  |  |  |  |  |  |  |  |  |  |  |  |  |  |  |  |  |  |  |  |  |  |  |  |  |  |  |  |  |  |  |
| f | ang | -0.107068 | -0.090944 | 0.021346 | -0.140837 | -0.231097 | 0.120188 | -0.101582 | -0.155755 | -0.212338 | -0.097965 | 0.484374 | 0.035292 | -0.089919 | 0.065455 | -0.239384 | -0.122578 | -0.118693 | 0.146498 | 0.044670 | 0.344048 | 0.145883 | 0.255147 | 0.364067 | -0.061439 | -0.129267 | -0.147019 | 0.547444 | -0.020446 | 0.031519 | 0.969053 | 0.477879 | -0.226840 | -0.233784 | -0.146768 | 0.651934 | -0.001234 | -0.250892 | -0.034475 | -0.133708 | -0.271287 | -0.215705 | -0.245044 | -0.127644 | -0.094971 | -0.183468 | -0.170725 | -0.187689 | -0.114234 |
|  | dis | -0.197694 | 0.061481 | -0.234455 | -0.156955 | -0.116681 | -0.229027 | -0.159949 | 0.004103 | -0.161257 | -0.209212 | -0.077736 | -0.004602 | 0.010296 | 0.081108 | 0.119742 | -0.017898 | -0.184315 | -0.036822 | -0.108139 | -0.043717 | 0.015189 | 0.921797 | 1.060071 | -0.102794 | -0.215771 | -0.190616 | -0.372721 | -0.040695 | -0.193600 | 1.098819 | 1.436805 | 0.007758 | -0.214569 | -0.222747 | -0.209609 | -0.005113 | -0.151673 | 0.039613 | -0.103777 | 0.084613 | 0.003531 | -0.158570 | -0.139650 | -0.259681 | -0.120911 | -0.063685 | -0.158616 | -0.081669 |
|  | fea | 0.077753 | -0.066480 | -0.141368 | -0.121815 | -0.012927 | -0.287902 | -0.147046 | -0.017016 | -0.061568 | 0.102944 | 0.736890 | -0.114484 | -0.240084 | 0.035541 | -0.134807 | -0.049666 | -0.032427 | -0.049715 | -0.082939 | -0.058181 | -0.169369 | 0.198205 | 0.376390 | -0.238898 | -0.011844 | 0.122511 | 0.026201 | -0.099165 | -0.007230 | 0.956243 | 0.122989 | -0.072396 | -0.224578 | 0.057937 | 0.839029 | 0.044465 | -0.080408 | -0.194371 | -0.158883 | -0.196069 | 0.083162 | -0.133403 | -0.140680 | -0.028346 | -0.066560 | -0.174366 | 0.049001 | -0.214271 |
|  | hap | -0.083151 | -0.176273 | -0.072354 | -0.014752 | -0.192790 | -0.121763 | -0.207403 | -0.184612 | -0.123583 | -0.118115 | 0.042775 | -0.155732 | 0.079402 | 0.812469 | 0.009954 | -0.149744 | -0.148304 | -0.109140 | -0.002345 | -0.099668 | -0.000368 | 0.759900 | 0.242644 | -0.197336 | -0.270702 | -0.031605 | -0.083610 | -0.197457 | -0.012785 | 0.837379 | 0.064816 | -0.261093 | 0.001064 | -0.101332 | 0.171326 | 0.087721 | 0.196049 | 0.715466 | -0.149423 | -0.221160 | 0.064848 | 0.033526 | 0.002885 | -0.177391 | -0.076576 | 0.016847 | -0.201242 | -0.197262 |
|  | ntr | -0.123786 | -0.018737 | 0.109495 | -0.083957 | -0.030939 | -0.008957 | -0.170157 | -0.192194 | -0.176686 | 0.000314 | 0.661145 | -0.199530 | -0.004440 | 0.459792 | -0.032845 | -0.071983 | -0.134917 | 0.030365 | 0.063706 | -0.197195 | -0.052643 | 0.393009 | 0.604447 | -0.353003 | -0.125066 | -0.119348 | -0.151487 | 0.079267 | 0.216065 | 0.114921 | 0.265603 | -0.147549 | -0.005426 | -0.022756 | 0.952202 | -0.034270 | 0.063469 | -0.343780 | -0.107934 | -0.172750 | -0.253165 | 0.041902 | 0.018439 | -0.266517 | -0.219938 | -0.091623 | -0.082322 | -0.078240 |

## Perform the PCA¶

In [6]:

```
sklearn_pca = sklearnPCA(n_components= 5 )
sklearn_pca_avg = sklearnPCA(n_components= 5 )
```

In [7]:

```
Y_sklearn = sklearn_pca.fit_transform(participantDf)
Y_sklearn_avg = sklearn_pca_avg.fit_transform(weightDf)
```

### Eigenvectors¶

These are the n extracted principal components. Each PC is composed of as many values as there are dimensions/features. Each value of the PC tells us the weight of the original dimension/feature.

In [8]:

```
sklearn_pca.explained_variance_ratio_
```

Out[8]:

```
array([ 0.04527311,  0.04126415,  0.03624892,  0.03417348,  0.03285534])
```

In [9]:

```
sklearn_pca_avg.explained_variance_ratio_
```

Out[9]:

```
array([ 0.37123251,  0.17716699,  0.11721804,  0.07849316,  0.05756008])
```

In [10]:

```
fig = plt.figure(figsize=(8,6))

expVar = sklearn_pca.explained_variance_ratio_

plt.plot(expVar*100, '-o',linewidth=7,markersize=16)
plt.xlim(-0.5,4.5)
plt.yticks(fontsize=20)
plt.xticks(np.arange(5),np.arange(1,5+1),fontsize=20)

plt.xlabel('Principal Component #',fontsize=25)
plt.ylabel('% Explained Variance',fontsize=25)

sns.despine()

plt.savefig('../figures/pcaExpVar.png',dpi=300)
plt.show()
```

### Project the weigths of each PC onto the original feature space¶

In [11]:

```
def plotPCWeights(Y_sklearn,components,explained_variance,figName='../figures/pcaCbar.png'):

    # set basic figure properties
    plt.figure(figsize=(16,6))

    w,h = (6,8)
    # loop through all eigenvectors
    for index,thisPC in enumerate(components):

        # make an empty w*h matrix with zeros
        a = np.zeros(shape=(w,h))

        # counter for the features
        count = 0
        
        # we loop through the matrix in a structured way
        for x in range(w):
            for y in range(h):
                a[x][y] = thisPC[count]
                count+=1

        # make a subplot
        ax = plt.subplot(1,len(components),index+1)

        # the heatmap: we have to transpose the matrix so it is oriented
        # as the picture was; we write the weights into each tile and get
        # rid of all other annotations
        sns.heatmap(a.T,
                    square=True,
                    cbar=False,
                    vmin=-0.4,vmax=0.4,
                    xticklabels=False,
                    yticklabels=False,
                    cmap=None)

        # the % explained variance is computed from the eigenvalue*100
        # we round for plotting reasons
        varExp = round(explained_variance[index]*100,1)
        
        # title with explained variance for each PC
        plt.title('PC #'+str(index+1)+'\nExplained. Var: '+str(varExp)+'%') 
        
    # shows all subplots
    plt.savefig(figName,dpi=300)
    plt.show()
```

In [12]:

```
def plotPCWeights(Y_sklearn,components,explained_variance,ax,figName='../figures/pcaCbar.png'):

    w,h = (6,8)
    # loop through all eigenvectors
    for index,thisPC in enumerate(components):

        # make an empty w*h matrix with zeros
        a = np.zeros(shape=(w,h))

        # counter for the features
        count = 0
        
        # we loop through the matrix in a structured way
        for x in range(w):
            for y in range(h):
                a[x][y] = thisPC[count]
                count+=1

        # make a subplot
        myAx = plt.subplot(1,len(components),index+1)

        # the heatmap: we have to transpose the matrix so it is oriented
        # as the picture was; we write the weights into each tile and get
        # rid of all other annotations
        ax = sns.heatmap(a.T,
                    square=True,
                    cbar=False,
                    vmin=-0.4,vmax=0.4,
                    xticklabels=False,
                    yticklabels=False,
                    cmap=None,
                    ax=myAx)

        # the % explained variance is computed from the eigenvalue*100
        # we round for plotting reasons
        varExp = round(explained_variance[index]*100,1)
        
        # title with explained variance for each PC
        myAx.set_title('PC #'+str(index+1)+'\nExplained. Var: '+str(varExp)+'%',fontsize=18) 
        
    return ax;
```

With all data:

In [13]:

```
f, ( ax1 ) = plt.subplots( 1,2,figsize=(16,8) );

plotPCWeights(Y_sklearn,
              sklearn_pca.components_,
              sklearn_pca.explained_variance_ratio_,
              ax1);
plt.savefig('../figures/pcaWeightsFaces.png',dpi=300)
```

With data averaged by condition (just out of curisity):

obviously, with only 14 data points, we can explain much more variance...

In [14]:

```
f, ( ax1 ) = plt.subplots( 1,2,figsize=(16,8) );

plotPCWeights(Y_sklearn,
              sklearn_pca_avg.components_,
              sklearn_pca_avg.explained_variance_ratio_,
              ax1);
```

### Projecting the faces back into the PCA-space¶

In [15]:

```
from matplotlib.offsetbox import OffsetImage, AnnotationBbox
from matplotlib._png import read_png
```

### Transform the values of each participant into the space¶

Project the data of each participant (48 tiles each) to the PCA space, where there are only 5 dimensions, each a combination of the 48 tiles

In [16]:

```
def makeParticipantPCA(participantDf,sklearn_pca=sklearn_pca):
    pcaDf = pd.DataFrame()
    for p in participantDf.index.levels[0]:
        thisEntry = sklearn_pca.transform( participantDf.ix[p] )
        thisDf = pd.DataFrame(thisEntry)
        thisDf.index = participantDf.ix[p].index
        # add participant name
        thisDf['p'] = p
        thisDf.set_index('p', append=True, inplace=True)
        thisDf = thisDf.reorder_levels(['p', 'ident', 'emo'])
        # add to group df
        pcaDf = pd.concat([pcaDf,thisDf])
    return pcaDf
```

In [17]:

```
pcaDf = makeParticipantPCA(participantDf)
```

In [18]:

```
pcaDf.head()
```

Out[18]:

|  |  |  | 0 | 1 | 2 | 3 | 4 |
| --- | --- | --- | --- | --- | --- | --- | --- |
| p | ident | emo |  |  |  |  |  |
| p001 | f | ang | 1.037068 | -0.412058 | -2.594713 | -0.848840 | -0.719657 |
|  |  | dis | -0.657484 | -2.868062 | 0.989515 | 1.414985 | 1.985290 |
|  |  | fea | -1.093147 | 2.201858 | 0.527650 | 1.874243 | -0.414441 |
|  |  | hap | -0.403686 | 1.290129 | 0.843942 | 0.691269 | -0.864649 |
|  |  | ntr | -0.874690 | 0.397866 | -0.769708 | 0.110667 | -0.323192 |

### Plotting¶

In [19]:

```
sns.palplot(stackColors)
```

Function to plot the data as a scatterplot

In [20]:

```
def makeScatterPCA(pcaDf,ax,identDict=identDict,emoDict=emoDict):

    # average over all participants
    meanPca = pcaDf.groupby(level=[1,2]).mean()
    n = len(pcaDf.index.levels[0])
    stdPca = pcaDf.groupby(level=[1,2]).std()/np.sqrt(n)*1.96

    # make the scatterplot
    for ident,emo in meanPca.index:
        
        thisMean = meanPca.ix[ident].ix[emo]
        thisStd = stdPca.ix[ident].ix[emo]

        myAx = ax.errorbar(thisMean[0], thisMean[1],
                           xerr = thisStd[0],yerr=thisStd[1],
                           c=stackColors[emoReverse[emo]],
                           ecolor='gray',
                           marker='ov'[identReverse[ident]],
                           markersize=15,
                           markeredgecolor='gray',
                           markeredgewidth=.5,
                           label=ident+' '+emo
                           );

    sns.despine()
    ax.set_xlim(-1.5,2);ax.set_ylim(-1,1)
    ax.set_ylabel("PC 2"); ax.set_xlabel("PC 1")
    #ax.legend(loc='upper right')

    return myAx
```

Function to plot the pictures

In [21]:

```
def makePicturePCA(pcaDf,ax,identDict=identDict,emoDict=emoDict,myZoom=.15):
    
    # average over all participants
    meanPca = pcaDf.groupby(level=[1,2]).mean()
    # make the scatterplot
    myAx = ax.scatter(meanPca[0], meanPca[1])

    # annoate it with the index labels from the dataframe
    for label, x, y in zip(meanPca.index, meanPca[0], meanPca[1]):

        imagebox = OffsetImage(read_png(picList[identReverse[label[0]] ][emoReverse[label[1]] ]), zoom=myZoom)
        ab = AnnotationBbox(imagebox, [x,y],pad=0)
        ax.add_artist(ab)

    sns.despine()        
    ax.set_ylabel("PC 2"); ax.set_xlabel("PC 1")
    ax.set_xlim(-1.5,2);ax.set_ylim(-1,1)
    
    return myAx;
```

In [22]:

```
f, (ax1,ax2 ) = plt.subplots( 1,2,figsize=(16,8) );
makeScatterPCA(pcaDf,ax1);
makePicturePCA(pcaDf,ax2,myZoom=.15);
#plt.savefig('../figures/pcaScatter.png',dpi=300)
```

Out[22]:

```
<matplotlib.collections.PathCollection at 0x7f16a5eeb910>
```
