# Supplementary material for: Mapping the emotional face. How individual face parts contribute to successful emotion recognition
Source: PLoS One. 2017 May 11;12(5):e0177239. doi: 10.1371/journal.pone.0177239 (PMC5426715; doi:10.1371/journal.pone.0177239)
Supplement: S7 Code — (HTML) [file pone.0177239.s009.html]

code006\_representationalSimilarityAnalysis


# Mapping the emotional face. How individual face parts contribute to successful emotion recognition.

# 6. Representational Similarity Analysis (RSA)¶

We take the probability matrices of each picture and correlate them with each other. Then we can use the correlation coefficient as a similarity metric.  
To plot the ratings in a space where high similartiy means close proximity and high dissimilarty is translated to high distances, we need to compute a dissimilarty metric as 1-r. Then we can use MDS to plot the dissimilarities. We do this in a simple scatterplot and by plotting the images themselves instead of points representing them. The whole approach is adapted from Kriegeskorte et al. 2008 Neuron; see also Edelman 1998 BBS.

### Import modules¶

In [1]:

```
from myBasics import *
%matplotlib inline

from itertools import chain
from sklearn import manifold
from sklearn.metrics import euclidean_distances
from sklearn.decomposition import PCA
from sklearn.preprocessing import StandardScaler
```

### get data¶

In [2]:

```
weightDf = pd.read_csv('../outputs/weightStdDf.csv',
                      index_col=[0,1,2])
weightDf.index.names = ['p','ident','emo']
weightDf = weightDf.sortlevel()
```

In [3]:

```
weightDf.head()
```

Out[3]:

|  |  |  | 0 | 1 | 2 | 3 | 4 | 5 | 6 | 7 | 8 | 9 | 10 | 11 | 12 | 13 | 14 | 15 | 16 | 17 | 18 | 19 | 20 | 21 | 22 | 23 | 24 | 25 | 26 | 27 | 28 | 29 | 30 | 31 | 32 | 33 | 34 | 35 | 36 | 37 | 38 | 39 | 40 | 41 | 42 | 43 | 44 | 45 | 46 | 47 |
| --- | --- | --- | --- | --- | --- | --- | --- | --- | --- | --- | --- | --- | --- | --- | --- | --- | --- | --- | --- | --- | --- | --- | --- | --- | --- | --- | --- | --- | --- | --- | --- | --- | --- | --- | --- | --- | --- | --- | --- | --- | --- | --- | --- | --- | --- | --- | --- | --- | --- | --- |
| p | ident | emo |  |  |  |  |  |  |  |  |  |  |  |  |  |  |  |  |  |  |  |  |  |  |  |  |  |  |  |  |  |  |  |  |  |  |  |  |  |  |  |  |  |  |  |  |  |  |  |  |
| p001 | f | ang | -0.948401 | -0.006541 | 1.667878 | -1.262355 | -1.262355 | -0.320494 | -0.948401 | 0.412064 | -1.262355 | -0.948401 | 1.039971 | 1.458576 | -1.262355 | 0.098110 | 0.935320 | -1.262355 | -0.948401 | 0.412064 | -1.262355 | 0.412064 | 1.039971 | -1.262355 | -0.006541 | -0.320494 | -0.320494 | -0.843750 | 2.400437 | 0.412064 | 1.353925 | 0.412064 | 1.458576 | -0.006541 | -1.262355 | -1.262355 | -1.262355 | 0.412064 | 1.039971 | 0.098110 | -0.320494 | -0.320494 | 0.098110 | 0.098110 | 0.098110 | 1.353925 | -1.262355 | 1.039971 | 1.353925 | 1.039971 |
|  |  | dis | -0.245679 | 1.036125 | -0.074772 | -1.100216 | -1.100216 | 0.437950 | 1.548847 | 0.865218 | -0.245679 | -1.100216 | 1.121579 | -1.100216 | 0.865218 | 0.779764 | 0.608857 | -1.100216 | 0.608857 | -0.587494 | -0.074772 | -1.100216 | -1.100216 | 1.805208 | 1.890662 | 0.437950 | -0.672947 | 0.352496 | 0.096135 | -1.100216 | -0.074772 | -1.100216 | 3.257920 | -0.587494 | 0.523404 | -0.074772 | 1.292486 | 0.352496 | -1.100216 | -1.100216 | 1.463394 | -0.074772 | -1.100216 | -0.587494 | -0.074772 | -0.074772 | -0.587494 | -0.587494 | -1.100216 | -0.416586 |
|  |  | fea | 0.582234 | -1.040508 | -1.040508 | -1.040508 | 0.401929 | -1.040508 | 0.401929 | 1.844366 | 0.401929 | 0.582234 | 1.844366 | 0.582234 | 0.221624 | 0.582234 | 1.664062 | 0.582234 | 0.582234 | -1.040508 | 0.221624 | 0.582234 | -1.040508 | 1.123148 | 0.221624 | -1.040508 | -1.040508 | -1.040508 | 0.221624 | 0.401929 | 0.221624 | 2.024671 | -1.040508 | -1.040508 | -1.040508 | 0.401929 | 1.844366 | 0.582234 | -1.040508 | -1.040508 | -1.040508 | -1.040508 | -1.040508 | 2.024671 | -1.040508 | -1.040508 | 0.221624 | -1.040508 | 0.221624 | 0.221624 |
|  |  | hap | 0.352580 | 1.057741 | -0.151106 | -0.856267 | -0.352580 | -1.359953 | 1.460690 | -0.453318 | -1.359953 | 1.460690 | 0.755529 | -0.352580 | 0.554055 | -0.554055 | -0.856267 | -0.654792 | -0.050369 | 0.151106 | -1.359953 | -0.957004 | 0.251843 | 1.057741 | -0.957004 | -0.957004 | -0.453318 | 0.251843 | -0.755529 | -0.352580 | 0.050369 | 0.251843 | -0.755529 | -1.259216 | 1.158478 | 0.151106 | 0.554055 | 1.762902 | -1.359953 | 1.762902 | -0.352580 | 1.158478 | -0.352580 | 2.065113 | -0.554055 | -1.359953 | 1.863639 | -1.359953 | 0.251843 | 1.762902 |
|  |  | ntr | -0.897600 | -0.897600 | 0.157930 | 0.733673 | 0.349844 | -1.089514 | -1.089514 | -0.801643 | -1.089514 | -0.321856 | 1.213459 | -0.321856 | 0.157930 | 0.733673 | 0.157930 | 0.157930 | -0.897600 | -0.609728 | 0.733673 | -1.089514 | -1.089514 | -0.513771 | 0.061972 | -0.321856 | 0.445801 | -0.033985 | 0.157930 | -1.089514 | -0.705685 | 0.445801 | 0.349844 | 2.077074 | 0.541758 | 1.981117 | 0.829630 | -1.089514 | -0.705685 | -0.321856 | 0.829630 | -1.089514 | 0.157930 | -1.089514 | 3.708346 | 0.061972 | -0.417814 | -0.705685 | 1.981117 | 0.253887 |

### average¶

In [4]:

```
weightAvgDf = weightDf.groupby(level=[1,2]).mean()
```

In [5]:

```
weightAvgDf
```

Out[5]:

|  |  | 0 | 1 | 2 | 3 | 4 | 5 | 6 | 7 | 8 | 9 | 10 | 11 | 12 | 13 | 14 | 15 | 16 | 17 | 18 | 19 | 20 | 21 | 22 | 23 | 24 | 25 | 26 | 27 | 28 | 29 | 30 | 31 | 32 | 33 | 34 | 35 | 36 | 37 | 38 | 39 | 40 | 41 | 42 | 43 | 44 | 45 | 46 | 47 |
| --- | --- | --- | --- | --- | --- | --- | --- | --- | --- | --- | --- | --- | --- | --- | --- | --- | --- | --- | --- | --- | --- | --- | --- | --- | --- | --- | --- | --- | --- | --- | --- | --- | --- | --- | --- | --- | --- | --- | --- | --- | --- | --- | --- | --- | --- | --- | --- | --- | --- |
| ident | emo |  |  |  |  |  |  |  |  |  |  |  |  |  |  |  |  |  |  |  |  |  |  |  |  |  |  |  |  |  |  |  |  |  |  |  |  |  |  |  |  |  |  |  |  |  |  |  |  |
| f | ang | -0.107068 | -0.090944 | 0.021346 | -0.140837 | -0.231097 | 0.120188 | -0.101582 | -0.155755 | -0.212338 | -0.097965 | 0.484374 | 0.035292 | -0.089919 | 0.065455 | -0.239384 | -0.122578 | -0.118693 | 0.146498 | 0.044670 | 0.344048 | 0.145883 | 0.255147 | 0.364067 | -0.061439 | -0.129267 | -0.147019 | 0.547444 | -0.020446 | 0.031519 | 0.969053 | 0.477879 | -0.226840 | -0.233784 | -0.146768 | 0.651934 | -0.001234 | -0.250892 | -0.034475 | -0.133708 | -0.271287 | -0.215705 | -0.245044 | -0.127644 | -0.094971 | -0.183468 | -0.170725 | -0.187689 | -0.114234 |
|  | dis | -0.197694 | 0.061481 | -0.234455 | -0.156955 | -0.116681 | -0.229027 | -0.159949 | 0.004103 | -0.161257 | -0.209212 | -0.077736 | -0.004602 | 0.010296 | 0.081108 | 0.119742 | -0.017898 | -0.184315 | -0.036822 | -0.108139 | -0.043717 | 0.015189 | 0.921797 | 1.060071 | -0.102794 | -0.215771 | -0.190616 | -0.372721 | -0.040695 | -0.193600 | 1.098819 | 1.436805 | 0.007758 | -0.214569 | -0.222747 | -0.209609 | -0.005113 | -0.151673 | 0.039613 | -0.103777 | 0.084613 | 0.003531 | -0.158570 | -0.139650 | -0.259681 | -0.120911 | -0.063685 | -0.158616 | -0.081669 |
|  | fea | 0.077753 | -0.066480 | -0.141368 | -0.121815 | -0.012927 | -0.287902 | -0.147046 | -0.017016 | -0.061568 | 0.102944 | 0.736890 | -0.114484 | -0.240084 | 0.035541 | -0.134807 | -0.049666 | -0.032427 | -0.049715 | -0.082939 | -0.058181 | -0.169369 | 0.198205 | 0.376390 | -0.238898 | -0.011844 | 0.122511 | 0.026201 | -0.099165 | -0.007230 | 0.956243 | 0.122989 | -0.072396 | -0.224578 | 0.057937 | 0.839029 | 0.044465 | -0.080408 | -0.194371 | -0.158883 | -0.196069 | 0.083162 | -0.133403 | -0.140680 | -0.028346 | -0.066560 | -0.174366 | 0.049001 | -0.214271 |
|  | hap | -0.083151 | -0.176273 | -0.072354 | -0.014752 | -0.192790 | -0.121763 | -0.207403 | -0.184612 | -0.123583 | -0.118115 | 0.042775 | -0.155732 | 0.079402 | 0.812469 | 0.009954 | -0.149744 | -0.148304 | -0.109140 | -0.002345 | -0.099668 | -0.000368 | 0.759900 | 0.242644 | -0.197336 | -0.270702 | -0.031605 | -0.083610 | -0.197457 | -0.012785 | 0.837379 | 0.064816 | -0.261093 | 0.001064 | -0.101332 | 0.171326 | 0.087721 | 0.196049 | 0.715466 | -0.149423 | -0.221160 | 0.064848 | 0.033526 | 0.002885 | -0.177391 | -0.076576 | 0.016847 | -0.201242 | -0.197262 |
|  | ntr | -0.123786 | -0.018737 | 0.109495 | -0.083957 | -0.030939 | -0.008957 | -0.170157 | -0.192194 | -0.176686 | 0.000314 | 0.661145 | -0.199530 | -0.004440 | 0.459792 | -0.032845 | -0.071983 | -0.134917 | 0.030365 | 0.063706 | -0.197195 | -0.052643 | 0.393009 | 0.604447 | -0.353003 | -0.125066 | -0.119348 | -0.151487 | 0.079267 | 0.216065 | 0.114921 | 0.265603 | -0.147549 | -0.005426 | -0.022756 | 0.952202 | -0.034270 | 0.063469 | -0.343780 | -0.107934 | -0.172750 | -0.253165 | 0.041902 | 0.018439 | -0.266517 | -0.219938 | -0.091623 | -0.082322 | -0.078240 |
|  | sad | 0.184882 | -0.127680 | -0.060984 | -0.158955 | -0.057357 | -0.021647 | -0.145245 | -0.084124 | -0.059754 | -0.122693 | 0.052222 | 0.053129 | -0.223741 | 0.074674 | 0.087649 | -0.172451 | -0.018550 | 0.107360 | 0.070615 | -0.133233 | -0.104635 | 0.109380 | -0.144394 | 0.053951 | 0.004425 | 0.706575 | 0.514547 | -0.047641 | 0.051109 | 0.252733 | -0.252668 | -0.077862 | -0.006967 | 0.136566 | 0.712770 | 0.059842 | -0.249183 | -0.163925 | -0.086291 | -0.004512 | -0.093885 | 0.052982 | 0.051361 | -0.106941 | -0.049065 | -0.166603 | -0.151522 | -0.244265 |
|  | sup | -0.200397 | 0.061519 | -0.204223 | -0.190441 | 0.003277 | -0.248203 | -0.105989 | -0.133349 | 0.019298 | -0.028401 | 0.351475 | 0.010811 | -0.186709 | -0.105716 | 0.171615 | -0.190557 | -0.199700 | -0.049054 | -0.122689 | -0.269113 | -0.016272 | 1.022867 | 0.514906 | -0.061519 | -0.159245 | -0.191216 | 0.214639 | -0.132690 | -0.186214 | 0.824110 | 0.655651 | 0.157623 | -0.111842 | -0.206953 | 0.576101 | -0.124684 | -0.047287 | -0.124257 | -0.100276 | -0.103540 | -0.165803 | -0.186152 | 0.023175 | -0.140398 | -0.034845 | -0.098798 | -0.115898 | -0.064637 |
| m | ang | -0.147810 | -0.090815 | 0.079869 | -0.067013 | -0.003200 | -0.111989 | -0.116224 | -0.349456 | -0.063999 | -0.086909 | 1.079562 | -0.024076 | -0.129015 | 0.049692 | -0.293144 | -0.141947 | -0.157273 | -0.137862 | 0.883143 | 0.031603 | -0.135489 | 0.534249 | 0.233757 | -0.115687 | -0.018857 | -0.053334 | 0.672953 | -0.063929 | -0.151923 | 0.365126 | 0.182592 | -0.262725 | -0.272455 | -0.329753 | 0.324460 | -0.104426 | -0.062720 | -0.171180 | -0.262166 | -0.004625 | 0.052623 | -0.114931 | 0.007077 | -0.071521 | -0.010132 | -0.191861 | -0.054781 | -0.123480 |
|  | dis | -0.183420 | -0.016911 | -0.073400 | 0.024479 | -0.234697 | -0.063476 | -0.084005 | -0.191738 | -0.090126 | -0.077726 | -0.187415 | -0.190714 | -0.026003 | -0.206813 | 0.067961 | 0.172052 | -0.076957 | -0.304362 | -0.062975 | -0.059768 | -0.104016 | 0.825245 | 0.957210 | 0.011119 | -0.152729 | -0.005959 | -0.076461 | -0.127019 | -0.064348 | 1.033164 | 0.791417 | -0.132160 | -0.285825 | -0.009192 | -0.066448 | 0.007000 | -0.296288 | 0.092349 | -0.133612 | 0.257393 | -0.340888 | -0.162663 | -0.127511 | 0.004255 | -0.253271 | -0.051498 | -0.038230 | 0.314981 |
|  | fea | -0.077231 | -0.200512 | -0.074860 | -0.140877 | -0.066320 | -0.126886 | 0.017264 | -0.107285 | 0.007130 | -0.091135 | 0.777351 | -0.017429 | -0.177906 | 0.069966 | 0.113103 | 0.132541 | -0.337640 | 0.021904 | 0.120930 | -0.100200 | -0.101916 | 0.377112 | 0.251506 | -0.255817 | -0.108004 | -0.208893 | 0.525016 | -0.038400 | -0.129689 | 0.212091 | 0.269984 | -0.190473 | -0.020385 | -0.178000 | 1.073120 | 0.018291 | 0.040373 | -0.233335 | 0.150789 | -0.113870 | -0.090264 | -0.290962 | 0.029329 | -0.157190 | -0.138786 | -0.107487 | -0.226428 | -0.099618 |
|  | hap | -0.050525 | -0.290847 | -0.085458 | -0.092325 | -0.189344 | -0.080268 | -0.042586 | -0.213514 | -0.164511 | -0.152753 | 0.223567 | 0.059805 | 0.176611 | 0.617089 | -0.046032 | -0.169695 | -0.247911 | -0.235120 | -0.028841 | -0.054830 | 0.058468 | 0.744898 | 0.044865 | -0.199634 | -0.115767 | -0.290798 | -0.060222 | -0.005151 | -0.093014 | 0.867706 | 0.336744 | -0.046914 | -0.249483 | -0.198457 | 0.007031 | -0.055391 | 0.177247 | 0.942656 | 0.074705 | -0.253048 | -0.019530 | -0.040397 | -0.059653 | 0.002774 | -0.095966 | -0.053750 | -0.206256 | -0.146175 |
|  | ntr | -0.218679 | -0.120495 | -0.053487 | -0.140802 | -0.027873 | 0.054360 | -0.109542 | -0.004019 | -0.036447 | -0.032446 | 0.106896 | -0.162269 | -0.088113 | 0.503841 | -0.018245 | -0.156115 | -0.131595 | -0.010068 | 0.052106 | -0.028120 | 0.320970 | 0.338143 | 0.080319 | -0.077805 | -0.116970 | -0.009940 | 0.068886 | -0.036699 | 0.169568 | 0.488858 | 0.257322 | -0.006068 | -0.177739 | -0.271038 | 0.174192 | -0.124064 | -0.055240 | 0.169659 | 0.058271 | -0.113255 | -0.279233 | -0.066863 | 0.044466 | -0.185020 | 0.054816 | -0.104298 | -0.035720 | 0.055597 |
|  | sad | -0.076002 | -0.054205 | -0.080972 | -0.177725 | -0.033782 | -0.041417 | -0.107863 | -0.139478 | -0.114452 | -0.050734 | 0.111651 | 0.006293 | 0.011445 | 0.023983 | -0.055468 | -0.047892 | -0.107585 | 0.109009 | -0.007500 | 0.016354 | 0.088728 | -0.057744 | 0.052238 | -0.066769 | -0.091346 | 0.233597 | 0.133486 | 0.253875 | -0.152544 | 0.087752 | 0.261514 | -0.073964 | -0.216595 | 0.013719 | 1.037263 | 0.157944 | -0.174235 | -0.145792 | -0.250436 | 0.049275 | -0.065697 | -0.102701 | -0.016207 | -0.054510 | 0.008708 | 0.003894 | -0.048377 | -0.048736 |
|  | sup | -0.054429 | -0.149024 | 0.035815 | -0.022497 | -0.062488 | -0.261285 | -0.173190 | -0.167149 | -0.030314 | -0.017201 | 0.637808 | -0.175138 | -0.090473 | -0.110422 | -0.156938 | -0.056181 | 0.116634 | 0.111349 | 0.192082 | -0.279182 | 0.099153 | 0.550366 | 0.350242 | -0.098910 | -0.008794 | 0.010870 | 0.251621 | -0.148239 | -0.091247 | 0.832018 | 0.214856 | -0.191427 | 0.170664 | -0.108776 | 0.609113 | -0.178041 | -0.037244 | -0.167970 | 0.008821 | -0.227562 | -0.241198 | 0.009626 | -0.114197 | -0.067991 | -0.206543 | -0.197860 | -0.157284 | -0.151846 |

### Transform to dissimilarities¶

In [6]:

```
dissWeight = 1 - weightAvgDf.T.corr()
```

In [7]:

```
dissWeight
```

Out[7]:

|  | ident | f | | | | | | | m | | | | | | |
| --- | --- | --- | --- | --- | --- | --- | --- | --- | --- | --- | --- | --- | --- | --- | --- |
|  | emo | ang | dis | fea | hap | ntr | sad | sup | ang | dis | fea | hap | ntr | sad | sup |
| ident | emo |  |  |  |  |  |  |  |  |  |  |  |  |  |  |
| f | ang | 0.000000 | 0.476491 | 0.262901 | 0.526918 | 0.426292 | 0.606374 | 0.310539 | 0.353957 | 0.463615 | 0.306928 | 0.498845 | 0.367775 | 0.433686 | 0.241164 |
|  | dis | 0.476491 | 0.000000 | 0.584528 | 0.465495 | 0.636283 | 1.134458 | 0.238734 | 0.739679 | 0.134840 | 0.734699 | 0.433183 | 0.470010 | 0.856303 | 0.529047 |
|  | fea | 0.262901 | 0.584528 | 0.000000 | 0.561444 | 0.330490 | 0.517788 | 0.317090 | 0.423827 | 0.583472 | 0.305420 | 0.630176 | 0.575863 | 0.423399 | 0.200198 |
|  | hap | 0.526918 | 0.465495 | 0.561444 | 0.000000 | 0.575025 | 0.838546 | 0.471021 | 0.681756 | 0.526483 | 0.693391 | 0.113149 | 0.304991 | 0.879547 | 0.512600 |
|  | ntr | 0.426292 | 0.636283 | 0.330490 | 0.575025 | 0.000000 | 0.696674 | 0.408133 | 0.474981 | 0.688317 | 0.251699 | 0.704923 | 0.491758 | 0.413829 | 0.325824 |
|  | sad | 0.606374 | 1.134458 | 0.517788 | 0.838546 | 0.696674 | 0.000000 | 0.747662 | 0.673415 | 1.024298 | 0.548052 | 1.019425 | 0.811255 | 0.397864 | 0.563475 |
|  | sup | 0.310539 | 0.238734 | 0.317090 | 0.471021 | 0.408133 | 0.747662 | 0.000000 | 0.459503 | 0.288438 | 0.324296 | 0.457873 | 0.395880 | 0.588767 | 0.226280 |
| m | ang | 0.353957 | 0.739679 | 0.423827 | 0.681756 | 0.474981 | 0.673415 | 0.459503 | 0.000000 | 0.723499 | 0.312079 | 0.659540 | 0.601791 | 0.650644 | 0.315321 |
|  | dis | 0.463615 | 0.134840 | 0.583472 | 0.526483 | 0.688317 | 1.024298 | 0.288438 | 0.723499 | 0.000000 | 0.754335 | 0.520312 | 0.534927 | 0.854830 | 0.495952 |
|  | fea | 0.306928 | 0.734699 | 0.305420 | 0.693391 | 0.251699 | 0.548052 | 0.324296 | 0.312079 | 0.754335 | 0.000000 | 0.700505 | 0.585824 | 0.361441 | 0.277235 |
|  | hap | 0.498845 | 0.433183 | 0.630176 | 0.113149 | 0.704923 | 1.019425 | 0.457873 | 0.659540 | 0.520312 | 0.700505 | 0.000000 | 0.305368 | 0.946475 | 0.569456 |
|  | ntr | 0.367775 | 0.470010 | 0.575863 | 0.304991 | 0.491758 | 0.811255 | 0.395880 | 0.601791 | 0.534927 | 0.585824 | 0.305368 | 0.000000 | 0.725656 | 0.473879 |
|  | sad | 0.433686 | 0.856303 | 0.423399 | 0.879547 | 0.413829 | 0.397864 | 0.588767 | 0.650644 | 0.854830 | 0.361441 | 0.946475 | 0.725656 | 0.000000 | 0.580746 |
|  | sup | 0.241164 | 0.529047 | 0.200198 | 0.512600 | 0.325824 | 0.563475 | 0.226280 | 0.315321 | 0.495952 | 0.277235 | 0.569456 | 0.473879 | 0.580746 | 0.000000 |

### show clustering of correlations¶

In [8]:

```
sns.clustermap(dissWeight,figsize=(6, 6));
```

### Multidimensional Scaling¶

Set parameters for mds

In [9]:

```
mds = manifold.MDS(n_components=2,
                   max_iter=3000,
                   eps=1e-9,
                   dissimilarity="precomputed",
                   n_jobs=1)
```

Apply parameters to the data

In [10]:

```
mdsPositions = mds.fit( dissWeight ).embedding_
```

We get positions in a 2d-space, close proximity means large similarity

Cave: This will give different values each time, as the distances do not fully determine the position (so each time the space is generated, it is rotated in a different way!)

In [11]:

```
print mdsPositions
```

```
[[-0.03842474 -0.0431317 ]
 [ 0.45703209  0.01421987]
 [-0.17096342 -0.00257937]
 [ 0.25136673 -0.34541343]
 [-0.16292284  0.24270857]
 [-0.61147792 -0.17826308]
 [ 0.16211755  0.07147817]
 [-0.02547554  0.39233151]
 [ 0.45739541  0.10164672]
 [-0.24117376  0.17401417]
 [ 0.34061738 -0.32549723]
 [ 0.09493259 -0.30728485]
 [-0.50260977  0.10025367]
 [-0.01041377  0.10551699]]
```

put into DataFrame

In [12]:

```
mdsDf = pd.DataFrame(mdsPositions,index=dissWeight.index)
mdsDf
```

Out[12]:

|  |  | 0 | 1 |
| --- | --- | --- | --- |
| ident | emo |  |  |
| f | ang | -0.038425 | -0.043132 |
|  | dis | 0.457032 | 0.014220 |
|  | fea | -0.170963 | -0.002579 |
|  | hap | 0.251367 | -0.345413 |
|  | ntr | -0.162923 | 0.242709 |
|  | sad | -0.611478 | -0.178263 |
|  | sup | 0.162118 | 0.071478 |
| m | ang | -0.025476 | 0.392332 |
|  | dis | 0.457395 | 0.101647 |
|  | fea | -0.241174 | 0.174014 |
|  | hap | 0.340617 | -0.325497 |
|  | ntr | 0.094933 | -0.307285 |
|  | sad | -0.502610 | 0.100254 |
|  | sup | -0.010414 | 0.105517 |

### Make a function to wrap this all together¶

In [13]:

```
def makeRsaDf(weightDf,mds=mds):
    # average over all participants
    weightAvgDf = weightDf.groupby(level=[1,2]).mean()
    # make dissimiarity matrix
    dissWeight = 1 - weightAvgDf.T.corr().fillna(0)
    # do the MDS
    mdsPositions = mds.fit( dissWeight ).embedding_
    # put into dataframe, annotate
    mdsDf = pd.DataFrame(mdsPositions,index=dissWeight.index)
    
    return mdsDf
```

In [14]:

```
mdsDf = makeRsaDf(weightDf)
mdsDf
```

Out[14]:

|  |  | 0 | 1 |
| --- | --- | --- | --- |
| ident | emo |  |  |
| f | ang | 0.088620 | -0.000163 |
|  | dis | -0.333937 | 0.315532 |
|  | fea | -0.041643 | -0.172794 |
|  | hap | 0.053524 | 0.423787 |
|  | ntr | -0.191002 | -0.245852 |
|  | sad | 0.374319 | -0.527642 |
|  | sup | -0.160831 | 0.071757 |
| m | ang | 0.347168 | -0.063976 |
|  | dis | -0.395457 | 0.251178 |
|  | fea | 0.104866 | -0.249121 |
|  | hap | 0.023343 | 0.481422 |
|  | ntr | 0.154762 | 0.292120 |
|  | sad | -0.014422 | -0.520012 |
|  | sup | -0.009310 | -0.056237 |

### Annotated Scatterplot¶

In [15]:

```
from matplotlib.offsetbox import OffsetImage, AnnotationBbox
from matplotlib._png import read_png
```

In [16]:

```
def makeScatter(mdsDf,ax,identDict=identDict,emoDict=emoDict):

    # make the scatterplot
    for ident,emo in mdsDf.index:
        thisDf = mdsDf.ix[ident].ix[emo]
        myAx = ax.errorbar(thisDf[0], thisDf[1],
                           c=stackColors[emoReverse[emo]],
                           marker='ov'[identReverse[ident]],
                           markersize=35,
                           markeredgewidth=.5,
                           #label=ident+' '+emo
                           );

    sns.despine()   
    ax.set_ylim(-0.5,0.4);ax.set_xlim(-0.6,0.8)
    #ax.legend(loc='upper right')
    ax.set_yticks([]); ax.set_xticks([])
    ax.set_ylabel(''); ax.set_xlabel('')
    
    return myAx
```

In [17]:

```
def makePicture(mdsDf,ax,myZoom=0.15):
    mdsDf.plot(0,1, kind='scatter', s=50,ax=ax)
    for label, x, y in zip(mdsDf.index, mdsDf[0], mdsDf[1]):
        imagebox = OffsetImage(read_png(picList[identReverse[label[0]] ][emoReverse[label[1]] ]), zoom=myZoom)
        ab = AnnotationBbox(imagebox, [x,y],pad=0)
        ax.add_artist(ab)
    ax.set_ylim(-0.5,0.4);ax.set_xlim(-0.6,0.8)
    ax.set_yticks([]); ax.set_xticks([])
    ax.set_ylabel(''); ax.set_xlabel('')
    return ax;
```

In [18]:

```
f, ( ax1, ax2 ) = plt.subplots( 1,2,figsize=(16,8) );
makeScatter(mdsDf,ax1);
makePicture(mdsDf,ax2,myZoom=0.15);
sns.despine()
#plt.savefig('../figures/mdsBehavior.png',dpi=300)
```

## RSA with raw pixel values¶

This is done with greyscale images for simplicity...

In [19]:

```
def makePixel(picList=picList,identDict=identDict,emoDict=emoDict):
    bigDf = pd.DataFrame()
    for i,ident in enumerate(picList):
        for e,express in enumerate(ident):
            # open image
            thisImg = Image.open(express)
            # convert to greyscale
            thisBw = thisImg.convert('L')
            # convert to array
            thisArray = np.array(thisBw)
            # convert to 1d list
            thisList = list(chain(*thisArray))
            # standardize
            floatList = map(float,thisList)
            thisStd = StandardScaler(with_std=False).fit_transform(floatList)

            # convert data frame
            thisDf = pd.DataFrame(thisStd).T
            idx = pd.MultiIndex.from_tuples( [(identDict[i],emoDict[e])] )
            thisDf.index = idx

            # append to big one
            try:
                bigDf = pd.concat([bigDf,thisDf])
            except:
                bigDf = thisDf
                
    bigDf = bigDf.sortlevel()
    
    return bigDf
```

In [20]:

```
bigPixelDf = makePixel()
```

```
/opt/anaconda2/lib/python2.7/site-packages/sklearn/preprocessing/data.py:583: DeprecationWarning: Passing 1d arrays as data is deprecated in 0.17 and will raise ValueError in 0.19. Reshape your data either using X.reshape(-1, 1) if your data has a single feature or X.reshape(1, -1) if it contains a single sample.
  warnings.warn(DEPRECATION_MSG_1D, DeprecationWarning)
/opt/anaconda2/lib/python2.7/site-packages/sklearn/preprocessing/data.py:646: DeprecationWarning: Passing 1d arrays as data is deprecated in 0.17 and will raise ValueError in 0.19. Reshape your data either using X.reshape(-1, 1) if your data has a single feature or X.reshape(1, -1) if it contains a single sample.
  warnings.warn(DEPRECATION_MSG_1D, DeprecationWarning)
/opt/anaconda2/lib/python2.7/site-packages/sklearn/preprocessing/data.py:583: DeprecationWarning: Passing 1d arrays as data is deprecated in 0.17 and will raise ValueError in 0.19. Reshape your data either using X.reshape(-1, 1) if your data has a single feature or X.reshape(1, -1) if it contains a single sample.
  warnings.warn(DEPRECATION_MSG_1D, DeprecationWarning)
/opt/anaconda2/lib/python2.7/site-packages/sklearn/preprocessing/data.py:646: DeprecationWarning: Passing 1d arrays as data is deprecated in 0.17 and will raise ValueError in 0.19. Reshape your data either using X.reshape(-1, 1) if your data has a single feature or X.reshape(1, -1) if it contains a single sample.
  warnings.warn(DEPRECATION_MSG_1D, DeprecationWarning)
/opt/anaconda2/lib/python2.7/site-packages/sklearn/preprocessing/data.py:583: DeprecationWarning: Passing 1d arrays as data is deprecated in 0.17 and will raise ValueError in 0.19. Reshape your data either using X.reshape(-1, 1) if your data has a single feature or X.reshape(1, -1) if it contains a single sample.
  warnings.warn(DEPRECATION_MSG_1D, DeprecationWarning)
/opt/anaconda2/lib/python2.7/site-packages/sklearn/preprocessing/data.py:646: DeprecationWarning: Passing 1d arrays as data is deprecated in 0.17 and will raise ValueError in 0.19. Reshape your data either using X.reshape(-1, 1) if your data has a single feature or X.reshape(1, -1) if it contains a single sample.
  warnings.warn(DEPRECATION_MSG_1D, DeprecationWarning)
/opt/anaconda2/lib/python2.7/site-packages/sklearn/preprocessing/data.py:583: DeprecationWarning: Passing 1d arrays as data is deprecated in 0.17 and will raise ValueError in 0.19. Reshape your data either using X.reshape(-1, 1) if your data has a single feature or X.reshape(1, -1) if it contains a single sample.
  warnings.warn(DEPRECATION_MSG_1D, DeprecationWarning)
/opt/anaconda2/lib/python2.7/site-packages/sklearn/preprocessing/data.py:646: DeprecationWarning: Passing 1d arrays as data is deprecated in 0.17 and will raise ValueError in 0.19. Reshape your data either using X.reshape(-1, 1) if your data has a single feature or X.reshape(1, -1) if it contains a single sample.
  warnings.warn(DEPRECATION_MSG_1D, DeprecationWarning)
/opt/anaconda2/lib/python2.7/site-packages/sklearn/preprocessing/data.py:583: DeprecationWarning: Passing 1d arrays as data is deprecated in 0.17 and will raise ValueError in 0.19. Reshape your data either using X.reshape(-1, 1) if your data has a single feature or X.reshape(1, -1) if it contains a single sample.
  warnings.warn(DEPRECATION_MSG_1D, DeprecationWarning)
/opt/anaconda2/lib/python2.7/site-packages/sklearn/preprocessing/data.py:646: DeprecationWarning: Passing 1d arrays as data is deprecated in 0.17 and will raise ValueError in 0.19. Reshape your data either using X.reshape(-1, 1) if your data has a single feature or X.reshape(1, -1) if it contains a single sample.
  warnings.warn(DEPRECATION_MSG_1D, DeprecationWarning)
/opt/anaconda2/lib/python2.7/site-packages/sklearn/preprocessing/data.py:583: DeprecationWarning: Passing 1d arrays as data is deprecated in 0.17 and will raise ValueError in 0.19. Reshape your data either using X.reshape(-1, 1) if your data has a single feature or X.reshape(1, -1) if it contains a single sample.
  warnings.warn(DEPRECATION_MSG_1D, DeprecationWarning)
/opt/anaconda2/lib/python2.7/site-packages/sklearn/preprocessing/data.py:646: DeprecationWarning: Passing 1d arrays as data is deprecated in 0.17 and will raise ValueError in 0.19. Reshape your data either using X.reshape(-1, 1) if your data has a single feature or X.reshape(1, -1) if it contains a single sample.
  warnings.warn(DEPRECATION_MSG_1D, DeprecationWarning)
/opt/anaconda2/lib/python2.7/site-packages/sklearn/preprocessing/data.py:583: DeprecationWarning: Passing 1d arrays as data is deprecated in 0.17 and will raise ValueError in 0.19. Reshape your data either using X.reshape(-1, 1) if your data has a single feature or X.reshape(1, -1) if it contains a single sample.
  warnings.warn(DEPRECATION_MSG_1D, DeprecationWarning)
/opt/anaconda2/lib/python2.7/site-packages/sklearn/preprocessing/data.py:646: DeprecationWarning: Passing 1d arrays as data is deprecated in 0.17 and will raise ValueError in 0.19. Reshape your data either using X.reshape(-1, 1) if your data has a single feature or X.reshape(1, -1) if it contains a single sample.
  warnings.warn(DEPRECATION_MSG_1D, DeprecationWarning)
/opt/anaconda2/lib/python2.7/site-packages/sklearn/preprocessing/data.py:583: DeprecationWarning: Passing 1d arrays as data is deprecated in 0.17 and will raise ValueError in 0.19. Reshape your data either using X.reshape(-1, 1) if your data has a single feature or X.reshape(1, -1) if it contains a single sample.
  warnings.warn(DEPRECATION_MSG_1D, DeprecationWarning)
/opt/anaconda2/lib/python2.7/site-packages/sklearn/preprocessing/data.py:646: DeprecationWarning: Passing 1d arrays as data is deprecated in 0.17 and will raise ValueError in 0.19. Reshape your data either using X.reshape(-1, 1) if your data has a single feature or X.reshape(1, -1) if it contains a single sample.
  warnings.warn(DEPRECATION_MSG_1D, DeprecationWarning)
/opt/anaconda2/lib/python2.7/site-packages/sklearn/preprocessing/data.py:583: DeprecationWarning: Passing 1d arrays as data is deprecated in 0.17 and will raise ValueError in 0.19. Reshape your data either using X.reshape(-1, 1) if your data has a single feature or X.reshape(1, -1) if it contains a single sample.
  warnings.warn(DEPRECATION_MSG_1D, DeprecationWarning)
/opt/anaconda2/lib/python2.7/site-packages/sklearn/preprocessing/data.py:646: DeprecationWarning: Passing 1d arrays as data is deprecated in 0.17 and will raise ValueError in 0.19. Reshape your data either using X.reshape(-1, 1) if your data has a single feature or X.reshape(1, -1) if it contains a single sample.
  warnings.warn(DEPRECATION_MSG_1D, DeprecationWarning)
/opt/anaconda2/lib/python2.7/site-packages/sklearn/preprocessing/data.py:583: DeprecationWarning: Passing 1d arrays as data is deprecated in 0.17 and will raise ValueError in 0.19. Reshape your data either using X.reshape(-1, 1) if your data has a single feature or X.reshape(1, -1) if it contains a single sample.
  warnings.warn(DEPRECATION_MSG_1D, DeprecationWarning)
/opt/anaconda2/lib/python2.7/site-packages/sklearn/preprocessing/data.py:646: DeprecationWarning: Passing 1d arrays as data is deprecated in 0.17 and will raise ValueError in 0.19. Reshape your data either using X.reshape(-1, 1) if your data has a single feature or X.reshape(1, -1) if it contains a single sample.
  warnings.warn(DEPRECATION_MSG_1D, DeprecationWarning)
/opt/anaconda2/lib/python2.7/site-packages/sklearn/preprocessing/data.py:583: DeprecationWarning: Passing 1d arrays as data is deprecated in 0.17 and will raise ValueError in 0.19. Reshape your data either using X.reshape(-1, 1) if your data has a single feature or X.reshape(1, -1) if it contains a single sample.
  warnings.warn(DEPRECATION_MSG_1D, DeprecationWarning)
/opt/anaconda2/lib/python2.7/site-packages/sklearn/preprocessing/data.py:646: DeprecationWarning: Passing 1d arrays as data is deprecated in 0.17 and will raise ValueError in 0.19. Reshape your data either using X.reshape(-1, 1) if your data has a single feature or X.reshape(1, -1) if it contains a single sample.
  warnings.warn(DEPRECATION_MSG_1D, DeprecationWarning)
/opt/anaconda2/lib/python2.7/site-packages/sklearn/preprocessing/data.py:583: DeprecationWarning: Passing 1d arrays as data is deprecated in 0.17 and will raise ValueError in 0.19. Reshape your data either using X.reshape(-1, 1) if your data has a single feature or X.reshape(1, -1) if it contains a single sample.
  warnings.warn(DEPRECATION_MSG_1D, DeprecationWarning)
/opt/anaconda2/lib/python2.7/site-packages/sklearn/preprocessing/data.py:646: DeprecationWarning: Passing 1d arrays as data is deprecated in 0.17 and will raise ValueError in 0.19. Reshape your data either using X.reshape(-1, 1) if your data has a single feature or X.reshape(1, -1) if it contains a single sample.
  warnings.warn(DEPRECATION_MSG_1D, DeprecationWarning)
/opt/anaconda2/lib/python2.7/site-packages/sklearn/preprocessing/data.py:583: DeprecationWarning: Passing 1d arrays as data is deprecated in 0.17 and will raise ValueError in 0.19. Reshape your data either using X.reshape(-1, 1) if your data has a single feature or X.reshape(1, -1) if it contains a single sample.
  warnings.warn(DEPRECATION_MSG_1D, DeprecationWarning)
/opt/anaconda2/lib/python2.7/site-packages/sklearn/preprocessing/data.py:646: DeprecationWarning: Passing 1d arrays as data is deprecated in 0.17 and will raise ValueError in 0.19. Reshape your data either using X.reshape(-1, 1) if your data has a single feature or X.reshape(1, -1) if it contains a single sample.
  warnings.warn(DEPRECATION_MSG_1D, DeprecationWarning)
/opt/anaconda2/lib/python2.7/site-packages/sklearn/preprocessing/data.py:583: DeprecationWarning: Passing 1d arrays as data is deprecated in 0.17 and will raise ValueError in 0.19. Reshape your data either using X.reshape(-1, 1) if your data has a single feature or X.reshape(1, -1) if it contains a single sample.
  warnings.warn(DEPRECATION_MSG_1D, DeprecationWarning)
/opt/anaconda2/lib/python2.7/site-packages/sklearn/preprocessing/data.py:646: DeprecationWarning: Passing 1d arrays as data is deprecated in 0.17 and will raise ValueError in 0.19. Reshape your data either using X.reshape(-1, 1) if your data has a single feature or X.reshape(1, -1) if it contains a single sample.
  warnings.warn(DEPRECATION_MSG_1D, DeprecationWarning)
```

In [21]:

```
bigPixelDf.head()
```

Out[21]:

|  |  | 0 | 1 | 2 | 3 | 4 | 5 | 6 | 7 | 8 | 9 | 10 | 11 | 12 | 13 | 14 | 15 | 16 | 17 | 18 | 19 | 20 | 21 | 22 | 23 | 24 | 25 | 26 | 27 | 28 | 29 | 30 | 31 | 32 | 33 | 34 | 35 | 36 | 37 | 38 | 39 | 40 | 41 | 42 | 43 | 44 | 45 | 46 | 47 | 48 | 49 | ... | 121750 | 121751 | 121752 | 121753 | 121754 | 121755 | 121756 | 121757 | 121758 | 121759 | 121760 | 121761 | 121762 | 121763 | 121764 | 121765 | 121766 | 121767 | 121768 | 121769 | 121770 | 121771 | 121772 | 121773 | 121774 | 121775 | 121776 | 121777 | 121778 | 121779 | 121780 | 121781 | 121782 | 121783 | 121784 | 121785 | 121786 | 121787 | 121788 | 121789 | 121790 | 121791 | 121792 | 121793 | 121794 | 121795 | 121796 | 121797 | 121798 | 121799 |
| --- | --- | --- | --- | --- | --- | --- | --- | --- | --- | --- | --- | --- | --- | --- | --- | --- | --- | --- | --- | --- | --- | --- | --- | --- | --- | --- | --- | --- | --- | --- | --- | --- | --- | --- | --- | --- | --- | --- | --- | --- | --- | --- | --- | --- | --- | --- | --- | --- | --- | --- | --- | --- | --- | --- | --- | --- | --- | --- | --- | --- | --- | --- | --- | --- | --- | --- | --- | --- | --- | --- | --- | --- | --- | --- | --- | --- | --- | --- | --- | --- | --- | --- | --- | --- | --- | --- | --- | --- | --- | --- | --- | --- | --- | --- | --- | --- | --- | --- | --- | --- | --- | --- |
| f | ang | -67.147280 | -66.147280 | -71.147280 | -74.147280 | -76.147280 | -76.147280 | -71.147280 | -72.147280 | -73.147280 | -71.147280 | -67.147280 | -69.147280 | -51.147280 | -48.147280 | -67.147280 | -73.147280 | -68.147280 | -69.147280 | -63.147280 | -59.147280 | -50.147280 | -26.147280 | -23.147280 | -34.147280 | -37.147280 | -39.147280 | -41.147280 | -36.147280 | -22.147280 | -23.147280 | -27.147280 | -17.147280 | -4.147280 | -2.147280 | -5.147280 | -7.147280 | -7.147280 | -9.147280 | -10.147280 | -0.147280 | 12.852720 | 15.852720 | 13.852720 | 5.852720 | -3.147280 | 1.852720 | 2.852720 | -0.147280 | 6.852720 | 10.852720 | ... | NaN | NaN | NaN | NaN | NaN | NaN | NaN | NaN | NaN | NaN | NaN | NaN | NaN | NaN | NaN | NaN | NaN | NaN | NaN | NaN | NaN | NaN | NaN | NaN | NaN | NaN | NaN | NaN | NaN | NaN | NaN | NaN | NaN | NaN | NaN | NaN | NaN | NaN | NaN | NaN | NaN | NaN | NaN | NaN | NaN | NaN | NaN | NaN | NaN | NaN |
|  | dis | -80.495422 | -78.495422 | -80.495422 | -82.495422 | -76.495422 | -70.495422 | -73.495422 | -79.495422 | -79.495422 | -81.495422 | -83.495422 | -82.495422 | -77.495422 | -75.495422 | -74.495422 | -78.495422 | -88.495422 | -91.495422 | -84.495422 | -75.495422 | -64.495422 | -58.495422 | -58.495422 | -52.495422 | -53.495422 | -55.495422 | -54.495422 | -49.495422 | -47.495422 | -47.495422 | -42.495422 | -38.495422 | -31.495422 | -24.495422 | -21.495422 | -20.495422 | -19.495422 | -16.495422 | -19.495422 | -25.495422 | -28.495422 | -27.495422 | -29.495422 | -29.495422 | -23.495422 | -14.495422 | -13.495422 | -16.495422 | -12.495422 | -2.495422 | ... | NaN | NaN | NaN | NaN | NaN | NaN | NaN | NaN | NaN | NaN | NaN | NaN | NaN | NaN | NaN | NaN | NaN | NaN | NaN | NaN | NaN | NaN | NaN | NaN | NaN | NaN | NaN | NaN | NaN | NaN | NaN | NaN | NaN | NaN | NaN | NaN | NaN | NaN | NaN | NaN | NaN | NaN | NaN | NaN | NaN | NaN | NaN | NaN | NaN | NaN |
|  | fea | -81.921512 | -83.921512 | -84.921512 | -82.921512 | -75.921512 | -78.921512 | -85.921512 | -84.921512 | -84.921512 | -84.921512 | -83.921512 | -84.921512 | -81.921512 | -70.921512 | -74.921512 | -84.921512 | -84.921512 | -85.921512 | -83.921512 | -72.921512 | -71.921512 | -87.921512 | -85.921512 | -83.921512 | -71.921512 | -57.921512 | -57.921512 | -62.921512 | -51.921512 | -41.921512 | -47.921512 | -54.921512 | -58.921512 | -55.921512 | -48.921512 | -35.921512 | -18.921512 | -25.921512 | -30.921512 | -23.921512 | -29.921512 | -42.921512 | -50.921512 | -50.921512 | -44.921512 | -47.921512 | -37.921512 | -23.921512 | -10.921512 | -1.921512 | ... | NaN | NaN | NaN | NaN | NaN | NaN | NaN | NaN | NaN | NaN | NaN | NaN | NaN | NaN | NaN | NaN | NaN | NaN | NaN | NaN | NaN | NaN | NaN | NaN | NaN | NaN | NaN | NaN | NaN | NaN | NaN | NaN | NaN | NaN | NaN | NaN | NaN | NaN | NaN | NaN | NaN | NaN | NaN | NaN | NaN | NaN | NaN | NaN | NaN | NaN |
|  | hap | -87.162660 | -83.162660 | -77.162660 | -82.162660 | -80.162660 | -81.162660 | -72.162660 | -78.162660 | -87.162660 | -87.162660 | -78.162660 | -72.162660 | -76.162660 | -90.162660 | -88.162660 | -84.162660 | -91.162660 | -90.162660 | -88.162660 | -91.162660 | -93.162660 | -81.162660 | -56.162660 | -50.162660 | -58.162660 | -63.162660 | -69.162660 | -70.162660 | -73.162660 | -72.162660 | -66.162660 | -58.162660 | -56.162660 | -64.162660 | -65.162660 | -39.162660 | -21.162660 | -33.162660 | -63.162660 | -64.162660 | -54.162660 | -53.162660 | -46.162660 | -34.162660 | -24.162660 | -22.162660 | -23.162660 | -20.162660 | -11.162660 | -9.162660 | ... | NaN | NaN | NaN | NaN | NaN | NaN | NaN | NaN | NaN | NaN | NaN | NaN | NaN | NaN | NaN | NaN | NaN | NaN | NaN | NaN | NaN | NaN | NaN | NaN | NaN | NaN | NaN | NaN | NaN | NaN | NaN | NaN | NaN | NaN | NaN | NaN | NaN | NaN | NaN | NaN | NaN | NaN | NaN | NaN | NaN | NaN | NaN | NaN | NaN | NaN |
|  | ntr | -81.018184 | -82.018184 | -81.018184 | -79.018184 | -85.018184 | -88.018184 | -79.018184 | -77.018184 | -83.018184 | -82.018184 | -78.018184 | -79.018184 | -77.018184 | -78.018184 | -92.018184 | -102.018184 | -95.018184 | -82.018184 | -72.018184 | -68.018184 | -64.018184 | -64.018184 | -69.018184 | -75.018184 | -72.018184 | -63.018184 | -54.018184 | -49.018184 | -44.018184 | -38.018184 | -38.018184 | -45.018184 | -58.018184 | -66.018184 | -63.018184 | -51.018184 | -35.018184 | -25.018184 | -29.018184 | -36.018184 | -30.018184 | -20.018184 | -13.018184 | -7.018184 | 0.981816 | 9.981816 | 17.981816 | 26.981816 | 28.981816 | 28.981816 | ... | NaN | NaN | NaN | NaN | NaN | NaN | NaN | NaN | NaN | NaN | NaN | NaN | NaN | NaN | NaN | NaN | NaN | NaN | NaN | NaN | NaN | NaN | NaN | NaN | NaN | NaN | NaN | NaN | NaN | NaN | NaN | NaN | NaN | NaN | NaN | NaN | NaN | NaN | NaN | NaN | NaN | NaN | NaN | NaN | NaN | NaN | NaN | NaN | NaN | NaN |

5 rows × 121800 columns

In [22]:

```
dissImg = 1 - bigPixelDf.T.corr()
```

In [23]:

```
sns.clustermap(dissImg,figsize=(6, 6));
```

In [24]:

```
def makeRsaImgDf(weightDf,mds=mds):
    # make dissimiarity matrix
    dissWeight = 1 - weightDf.T.corr().fillna(0)
    # do the MDS
    mdsPositions = mds.fit( dissWeight ).embedding_
    # put into dataframe, annotate
    mdsDf = pd.DataFrame(mdsPositions,index=dissWeight.index)
    
    return mdsDf
```

In [25]:

```
mdsImgDf = makeRsaImgDf(bigPixelDf)
```

In [26]:

```
mdsImgDf.head()
```

Out[26]:

|  |  | 0 | 1 |
| --- | --- | --- | --- |
| f | ang | -0.538891 | 0.166276 |
|  | dis | -0.477310 | 0.251867 |
|  | fea | -0.239341 | 0.489169 |
|  | hap | -0.137641 | 0.446497 |
|  | ntr | -0.351184 | 0.272551 |

In [27]:

```
def makeScatter(mdsDf,ax,identDict=identDict,emoDict=emoDict):

    # make the scatterplot
    for ident,emo in mdsDf.index:
        thisDf = mdsDf.ix[ident].ix[emo]
        myAx = ax.errorbar(thisDf[0], thisDf[1],
                           c=stackColors[emoReverse[emo]],
                           marker='ov'[identReverse[ident]],
                           markersize=35,
                           markeredgewidth=.5,
                           #label=ident+' '+emo
                           );

    sns.despine()   
    ax.set_ylim(-0.75,0.6);ax.set_xlim(-0.6,0.6)
    #ax.legend(loc='upper right')
    ax.set_yticks([]); ax.set_xticks([])
    ax.set_ylabel(''); ax.set_xlabel('')
    
    return myAx

def makePicture(mdsDf,ax,myZoom=0.15):
    mdsDf.plot(0,1, kind='scatter', s=50,ax=ax)
    for label, x, y in zip(mdsDf.index, mdsDf[0], mdsDf[1]):
        imagebox = OffsetImage(read_png(picList[identReverse[label[0]] ][emoReverse[label[1]] ]), zoom=myZoom)
        ab = AnnotationBbox(imagebox, [x,y],pad=0)
        ax.add_artist(ab)
    ax.set_ylim(-0.75,0.6);ax.set_xlim(-0.6,0.6)
    ax.set_yticks([]); ax.set_xticks([])
    ax.set_ylabel(''); ax.set_xlabel('')
    return ax;


f, ( ax1, ax2 ) = plt.subplots( 1,2,figsize=(16,8) );
makeScatter(mdsImgDf,ax1);
makePicture(mdsImgDf,ax2,myZoom=.15);
sns.despine()

#plt.savefig('../figures/mdsPixel.png',dpi=300)
```
