# Supplementary material for: Mapping the emotional face. How individual face parts contribute to successful emotion recognition
Source: PLoS One. 2017 May 11;12(5):e0177239. doi: 10.1371/journal.pone.0177239 (PMC5426715; doi:10.1371/journal.pone.0177239)
Supplement: S8 Code — (HTML) [file pone.0177239.s010.html]

code007\_interactivePlots


# Mapping the emotional face. How individual face parts contribute to successful emotion recognition.

# 7. Interactive Plotting of Trials¶

This notebook creates tables which contain information about which tiles were revealed in each trial.

In [1]:

```
from myBasics import *
%matplotlib inline
```

Example of the first female face:

In [2]:

```
filename = picF[0]
im = Image.open(filename, 'r')
plt.imshow(im)
plt.show()
```

## Load all logfiles¶

Here we load the dataframes that have been generated in the 'DataImport' notebook.

In [3]:

```
logList = getFile('../rawTables/','pand*.csv')
```

### Make a table with all revealed tiles¶

In [4]:

```
def getRevealedTable(df,ident,express,dims,thisAns):
    '''
    df: the pandas dataframe of a particular participant, as stored in the rawTables folder
    ident: the identity of the face (0:female or 1:male)
    express: the expression of the face (0:happy, 1:sad, 2:angry, 3:fearful, 4:disgusted, 5:surprised, 6:neutral)
    dims: in how many tiles is the face divided into ( in this experiment, this is 8x6 (height x width) )
    thisAns: the answer given (e.g. 'hap' for a happy face would be the correct response)
    '''
    # df to write to
    bigDf = pd.DataFrame()
    
    # we go through all items of one specific identity showing one specific expression
    for whichItem in df.ix[ident].ix[express].index:
        
        # if the answer for the current item is the one we are looking for (e.g. correct or a certain
        # kind of mistake)
        if df.ix[ident].ix[express].ix[whichItem]['button'] == thisAns:
            
            # we create a dict with zeroes, one for each possible position
            d = {}
            for num in range(dims[0]*dims[1]):
                d[num] = 0
            
            # we take all the tile numbers and assign each tile that was present
            # the number one
            for num in df.ix[ident].ix[express].ix[whichItem]['maskList'].split('-'):
                d[int(num)] = 1

            # for each item, we create a df
            thisDf = pd.DataFrame(d,index=[whichItem])
            # we append to the big df
            try:
                bigDf = pd.concat([bigDf,thisDf])
            except:
                bigDf = thisDf
                
    return bigDf
```

Example: we know that there are 8\*6=48 tiles, so we create a table with columns from 0 to 47. All positions are created with default values of zero, indicating that this part of the face was hidden. Then for each item we ask whether a tile was revealed; if so, its value is changed to 1.  
In the following example, a single subject dataframe is loaded (df), the female face is selected (first 0), the happy expression is selected (second 0), the tile dimensions are specified (8,6) and the type of answer given is selected ('hap').

In [5]:

```
df = pd.read_csv(logList[-1],index_col=[0,1,2])
df.head()
```

Out[5]:

|  |  |  | time | cumtime | e | i | button | filename | evaluation | stopRT | choiceRT | maskNum | maskList | # |
| --- | --- | --- | --- | --- | --- | --- | --- | --- | --- | --- | --- | --- | --- | --- |
| ident | express | id |  |  |  |  |  |  |  |  |  |  |  |  |
| 0 | 0 | 9 | 2016-03-10 15:08:11 | 198.14952 | 0.0 | 0.0 | hap | img/f\_hap\_cut.png | HIT | 12275.0 | 16115.0 | 13.0 | 3-28-29-7-24-44-45-22-10-43-30-18-34 | 9.0 |
|  |  | 27 | 2016-03-10 15:15:00 | 607.15364 | 0.0 | 0.0 | hap | img/f\_hap\_cut.png | HIT | 19242.0 | 20994.0 | 20.0 | 40-15-0-37-1-27-45-46-44-23-18-43-22-3-41-29-1... | 27.0 |
|  |  | 37 | 2016-03-10 15:18:36 | 823.46331 | 0.0 | 0.0 | hap | img/f\_hap\_cut.png | HIT | 16303.0 | 17847.0 | 17.0 | 9-44-23-35-39-14-1-40-46-19-28-20-26-37-17-21-30 | 37.0 |
|  |  | 43 | 2016-03-10 15:20:31 | 937.80766 | 0.0 | 0.0 | hap | img/f\_hap\_cut.png | HIT | 5062.0 | 7262.0 | 6.0 | 36-29-47-3-43-20 | 43.0 |
|  |  | 58 | 2016-03-10 15:23:36 | 1123.17332 | 0.0 | 0.0 | hap | img/f\_hap\_cut.png | HIT | 5377.0 | 6801.0 | 6.0 | 19-20-16-39-13-44 | 58.0 |

In [6]:

```
exampleRevealed = getRevealedTable(df,0,0,(8,6),'hap')
```

This gives us a table with trial number as index and the numbering of the tiles as columns. Each 1 indicates that this tile was revealed in the respective trial.

In [7]:

```
exampleRevealed.head()
```

Out[7]:

|  | 0 | 1 | 2 | 3 | 4 | 5 | 6 | 7 | 8 | 9 | 10 | 11 | 12 | 13 | 14 | 15 | 16 | 17 | 18 | 19 | 20 | 21 | 22 | 23 | 24 | 25 | 26 | 27 | 28 | 29 | 30 | 31 | 32 | 33 | 34 | 35 | 36 | 37 | 38 | 39 | 40 | 41 | 42 | 43 | 44 | 45 | 46 | 47 |
| --- | --- | --- | --- | --- | --- | --- | --- | --- | --- | --- | --- | --- | --- | --- | --- | --- | --- | --- | --- | --- | --- | --- | --- | --- | --- | --- | --- | --- | --- | --- | --- | --- | --- | --- | --- | --- | --- | --- | --- | --- | --- | --- | --- | --- | --- | --- | --- | --- |
| 9 | 0 | 0 | 0 | 1 | 0 | 0 | 0 | 1 | 0 | 0 | 1 | 0 | 0 | 0 | 0 | 0 | 0 | 0 | 1 | 0 | 0 | 0 | 1 | 0 | 1 | 0 | 0 | 0 | 1 | 1 | 1 | 0 | 0 | 0 | 1 | 0 | 0 | 0 | 0 | 0 | 0 | 0 | 0 | 1 | 1 | 1 | 0 | 0 |
| 27 | 1 | 1 | 0 | 1 | 0 | 0 | 0 | 0 | 0 | 0 | 1 | 1 | 0 | 0 | 0 | 1 | 0 | 0 | 1 | 0 | 0 | 0 | 1 | 1 | 1 | 0 | 0 | 1 | 0 | 1 | 0 | 0 | 0 | 0 | 0 | 0 | 1 | 1 | 0 | 0 | 1 | 1 | 0 | 1 | 1 | 1 | 1 | 0 |
| 37 | 0 | 1 | 0 | 0 | 0 | 0 | 0 | 0 | 0 | 1 | 0 | 0 | 0 | 0 | 1 | 0 | 0 | 1 | 0 | 1 | 1 | 1 | 0 | 1 | 0 | 0 | 1 | 0 | 1 | 0 | 1 | 0 | 0 | 0 | 0 | 1 | 0 | 1 | 0 | 1 | 1 | 0 | 0 | 0 | 1 | 0 | 1 | 0 |
| 43 | 0 | 0 | 0 | 1 | 0 | 0 | 0 | 0 | 0 | 0 | 0 | 0 | 0 | 0 | 0 | 0 | 0 | 0 | 0 | 0 | 1 | 0 | 0 | 0 | 0 | 0 | 0 | 0 | 0 | 1 | 0 | 0 | 0 | 0 | 0 | 0 | 1 | 0 | 0 | 0 | 0 | 0 | 0 | 1 | 0 | 0 | 0 | 1 |
| 58 | 0 | 0 | 0 | 0 | 0 | 0 | 0 | 0 | 0 | 0 | 0 | 0 | 0 | 1 | 0 | 0 | 1 | 0 | 0 | 1 | 1 | 0 | 0 | 0 | 0 | 0 | 0 | 0 | 0 | 0 | 0 | 0 | 0 | 0 | 0 | 0 | 0 | 0 | 0 | 1 | 0 | 0 | 0 | 0 | 1 | 0 | 0 | 0 |

## Visualise each trial in the face space¶

As we have a table that codes the presence/absence of each tile in every trial, we can use this information to reconstruct each trial of each participant. This might allow for better visualization of results and an intuitive inspection of how the participants approached the experiment.

### Make a list of all possible coordinates¶

These coordinates (x,y) must be created in the same way as they are in the JavaScript of the experiment. We start at the top left corner and work down the first column, then the 2nd column etc.

In [8]:

```
def makeCoordinates(xNum,yNum,squareSize):
    myArray = []
    xDim=xNum*squareSize
    yDim=yNum*squareSize
    for x in np.arange(0,xDim,squareSize):
        for y in np.arange(0,yDim,squareSize):
            myArray.append( (x,y) )
    
    return myArray
```

### For each coordinate a square of a certain size can be draw upon the original image¶

In [9]:

```
def makeSquare(xStart,yStart,squareSize,im):
    # the face image is used for drawing upon
    draw  =  ImageDraw.Draw(im)
    # for each coordinate, a square is drawn
    draw.polygon([(xStart,yStart),
                  (xStart+squareSize,yStart),
                  (xStart+squareSize,yStart+squareSize),
                  (xStart,yStart+squareSize)],
                 
                 fill="white",
                 outline=None
                )
    return im
```

### Apply the masking for one trial¶

In [10]:

```
def makeOneTrial(pic,thisDf,trial,hDim,vDim,squareSize):
    # first we rescale the image
    width = hDim*squareSize
    height = vDim*squareSize
    outIm = Image.open(pic,'r')
    outIm = outIm.resize((width,height), PIL.Image.ANTIALIAS)
    
    # generate all tile positions
    thisCoords = makeCoordinates(hDim,vDim,squareSize)
    # go through all tiles of the trial
    for i,tile in enumerate(thisDf.ix[trial]):
        # each time the tile was not revealed
        if tile == 0:
            # we mask the image at that position
            xPos,yPos = thisCoords[i]
            outIm = makeSquare(xPos,yPos,squareSize,outIm)

    return outIm
```

Example:

Here we reconstruct the trial of the first correct resonse to the famale happy face.

In [11]:

```
exampleRevealed.ix[ exampleRevealed.index[0] ]
```

Out[11]:

```
0     0
1     0
2     0
3     1
4     0
5     0
6     0
7     1
8     0
9     0
10    1
11    0
12    0
13    0
14    0
15    0
16    0
17    0
18    1
19    0
20    0
21    0
22    1
23    0
24    1
25    0
26    0
27    0
28    1
29    1
30    1
31    0
32    0
33    0
34    1
35    0
36    0
37    0
38    0
39    0
40    0
41    0
42    0
43    1
44    1
45    1
46    0
47    0
Name: 9, dtype: int64
```

In [12]:

```
# get the list of present/absent tiles
exampleRevealed = getRevealedTable(df,0,0,(8,6),'hap')
# show the first trial of that condition in the face space
makeOneTrial(picList[0][0],exampleRevealed,exampleRevealed.index[0],6,8,50)
```

Out[12]:

We might like to contrast this with the very last trial of that condition:

In [13]:

```
# get the list of present/absent tiles
exampleRevealed = getRevealedTable(df,0,0,(8,6),'hap')
# show the first trial of that condition in the face space
makeOneTrial(picList[0][0],exampleRevealed,exampleRevealed.index[-1],6,8,50)
```

Out[13]:

These two examples alone suggest that participants perfomance improved (fewer information needed for a correct response) as the experiment progressed.

## Allow for interactive exploration of each trial¶

Import modules for interactive widgets

In [14]:

```
from IPython.html import widgets
from IPython.html.widgets import interact
from IPython.display import display
```

```
/opt/anaconda2/lib/python2.7/site-packages/IPython/html.py:14: ShimWarning: The `IPython.html` package has been deprecated. You should import from `notebook` instead. `IPython.html.widgets` has moved to `ipywidgets`.
  "`IPython.html.widgets` has moved to `ipywidgets`.", ShimWarning)
```

In [15]:

```
def myInteract(p,trialNumber,ident,express,thisAns):    
    
    # load df
    thisFile = logList[int(p)] 
    
    df = pd.read_csv(thisFile,
                 header=0,
                 index_col=[0,1,2]
                )
    
    # this is hard-coded into the function, because
    # we do not want to interact with these values
    # set them correctly here, otherwise results will be invalid!
    dims=(8,6)
    squareSize=50
    # this must be identical to the lists used in the widgets, otherwise
    # the results will be invalid
    i = ['female','male']
    e = ['happy','sad','angry','fearful','disgusted','surprised','neutral']
    
    try:
        # since we need the input to be a list of strings for formatting reasons
        # we must take an extra step to transform back to numbers by getting the 
        # position of the input in the list
        iNum = i.index(ident)
        eNum = e.index(express)

        # get the current condition
        thisDf = getRevealedTable(df,iNum,eNum,dims,thisAns)
        # get the real trial number
        thisNum = thisDf.index[trialNumber]

        # reconstruct the item

        thisTest = makeOneTrial(picList[iNum][eNum],
                                thisDf,
                                thisNum,
                                dims[1],
                                dims[0],
                                squareSize)

        # what answer was given?
        thisAns = df.ix[iNum].ix[eNum].ix[thisNum]['button']

        # what is the name of the logfile?
        pName = 'p'+logList[int(p)][-7:-4]

        # show the reconstructed item
        plt.imshow(thisTest)
        plt.title(pName+'\nTrial Number: '+str(thisDf.index[trialNumber]))
        plt.xticks([]);plt.yticks([])
        plt.xlabel('Participant\'s answer:\n'+thisAns,
                   size=15)
        plt.show()
        
    except:
        print "Sorry. No such item"
```

In [16]:

```
sns.set_style('dark')
```

In [17]:

```
participant = (0, len(logList)-1 )
identity = ['female','male']
emo = ['happy','sad','angry','fearful','disgusted','surprised','neutral']
answer = ['hap','sad','ang','fea','dis','sup','ntr']
```

In [18]:

```
trialReconstruction = interact(myInteract,
                               p=participant,
                               trialNumber=(0,15),
                               ident=identity,
                               express=emo,
                               thisAns=answer
                              )
```
