# Supplementary material for: Mapping the emotional face. How individual face parts contribute to successful emotion recognition
Source: PLoS One. 2017 May 11;12(5):e0177239. doi: 10.1371/journal.pone.0177239 (PMC5426715; doi:10.1371/journal.pone.0177239)
Supplement: S9 Code — (HTML) [file pone.0177239.s011.html]

code008\_actionUnits\_interactive


# Mapping the emotional face. How individual face parts contribute to successful emotion recognition.

# 8. Interactive plotting of action unit weights¶

This notebook aggregates the tiles in accordance to the Ekman Action Units (AUs) they fall into.  
Unlike notebook 4, here also mistakes are being considered. I.e. while previously the weights were given only for correct responses, now we can also visualize which areas in the face drive incorrect labeling, like seeing the eyes in a fearful face but thinking it represents surprise.  
Due to the richness of the data that emerge (14 faces x 7 response types), this is done interactively, so figures are generated on the fly.

### importing the basic modules and files¶

In [1]:

```
from myBasics import *
%matplotlib inline
```

In [2]:

```
logList = getFile('../rawTables/','pand*.csv')
```

## Assign AUs to tiles¶

Get a list of all possible tile coordinates

In [3]:

```
def makeCoordinates(xNum,yNum,squareSize):
    myArray = []
    xDim=xNum*squareSize
    yDim=yNum*squareSize
    for x in np.arange(0,xDim,squareSize):
        for y in np.arange(0,yDim,squareSize):
            myArray.append( (x,y) )
    
    return myArray
```

In [4]:

```
thisCoord = makeCoordinates(6,8,50)
print thisCoord
print "\nNumber of Coordinates:", len(makeCoordinates(6,8,50))
```

```
[(0, 0), (0, 50), (0, 100), (0, 150), (0, 200), (0, 250), (0, 300), (0, 350), (50, 0), (50, 50), (50, 100), (50, 150), (50, 200), (50, 250), (50, 300), (50, 350), (100, 0), (100, 50), (100, 100), (100, 150), (100, 200), (100, 250), (100, 300), (100, 350), (150, 0), (150, 50), (150, 100), (150, 150), (150, 200), (150, 250), (150, 300), (150, 350), (200, 0), (200, 50), (200, 100), (200, 150), (200, 200), (200, 250), (200, 300), (200, 350), (250, 0), (250, 50), (250, 100), (250, 150), (250, 200), (250, 250), (250, 300), (250, 350)]

Number of Coordinates: 48
```

### Small script to get the rgb color of each mask¶

In [5]:

```
def getColor(imName):

    im = Image.open(imName)
    # we loop through all pixels
    for y in xrange(im.size[1]):
        for x in xrange(im.size[0]):
            # we get the RGB+alpha values of each pixel
            r,g,b,a =  im.load()[x, y]
            # we are only intereset in colored pixels
            if (r,g,b) != (0,0,0):
                # store the present color for later re-use
                thisColor = (r,g,b)
                # and stop here
                break
    return thisColor
```

### Determining for each tile, whether it contains some colored pixels¶

We loop through the image, tile by tile. For this, we use For each tile, count the occurence of each color on a pixel-by-pixel basis. We use the thisCoord list of all tile coordinates (where each coordinate tuple denotes the upper left corner of the 50x50 tile).  
The first output is a dictionary, with tile coordinates as keys and number of colored pixels as values.  
The second output is the RGB value of the color used for that AU. Since each image contains only one AU and should have only one color, this works by taking the color of any non-black (0,0,0) pixel.

In [6]:

```
def getTiles(im,thisCoord=thisCoord,squareSize=50):
    
    # resize the image, if it is not exactly 300x400 (as it was presented in the experiment),
    # this should not be necessary in general
    im = im.resize((300,400))
    
    # the dictionary where we write the occurence of colored pixels for each tile
    thisCut = {}

    # we loop through the coordinate list
    for coord in thisCoord:
        # we get the (h)orizontal and (v)ertical coordinates from the tuple
        h,v = coord

        # we cut out the tile
        cut = im.crop((h,v,h+squareSize,v+squareSize))
        pixdata=cut.load()

        # we loop through all pixels of that tile
        for y in xrange(cut.size[1]):
            for x in xrange(cut.size[0]):
                # we get the RGB+alpha values of each pixel
                r,g,b,a =  pixdata[x, y]
                # we are only intereset in colored pixels
                if (r,g,b) != (0,0,0):
                    # store the present color for later re-use
                    thisColor = (r,g,b)
                    # count the occurence of colored pixels for that tile/coordinate pair
                    try:
                        thisCut[coord]+=1
                    except:
                        thisCut[coord]=1

        # if the tile remained empty, assign zero
        if coord not in thisCut:
            thisCut[coord] = 0
    
    # return the dict and a tuple with RGB values of that Action Unit
    return thisCut,thisColor
```

### get tiles for all AUs¶

In [7]:

```
def makeAUTable(myPicList):
    
    # get number of colored pixels in each tile for all AUs of a face
    d = {}
    for pic in myPicList:
        d[pic] = getTiles( Image.open(pic) )
        
    # transform this into a dataframe
    bigDf = pd.DataFrame()
    for key in d:
        thisDf = pd.DataFrame(d[key][0],index=[key]).T
        bigDf = pd.concat([bigDf,thisDf],axis=1)

    return bigDf
```

Binarize in a way that multiple AUs can belong to a tile, but only if they pass a defined cutoff

In [8]:

```
def makeBinAUs(bigDf,cutOff=25):
         
    return (bigDf>cutOff).astype(int)
```

### visualize the result¶

In [9]:

```
def showTiles(bgIm,d,squareSize=50):

    imDict = {}

    # loop through the action units
    for au in d:
        
        # get the face image as a background to visualize on
        imOut = Image.open(bgIm)
        imOut = imOut.resize((300,400))
    
        # loop through the tile coordinates
        for coords in d[au]:

            h,v = coords
            if d[au][coords] == 1:
                thisColor = getColor(au)
            else:
                thisColor = 0

            faceTile = imOut.crop((h,v,h+squareSize,v+squareSize))

            if thisColor == 0:

                imOut.paste(faceTile, (h,v))

            else:
                pixdata=faceTile.load()

                # change its color by looping through all the pixels of the cutout
                for y in xrange(faceTile.size[1]):
                    for x in xrange(faceTile.size[0]):
                        pixdata[x, y] = thisColor
                    # paste the fully colored tile onto the background image
                    imOut.paste(faceTile, (h,v))
                    
        imDict[au] = imOut
        
    return imDict
```

In [10]:

```
myPicList = getFile('../auLabels/','f_ang*.png')
```

In [11]:

```
d = makeBinAUs( makeAUTable(myPicList) ).to_dict()
imDict = showTiles(picList[0][2],d)
```

In [12]:

```
fig = plt.figure(figsize=(16,6))
for i,im in enumerate(imDict):
    ax = plt.subplot( 1, len(imDict.keys()), i+1 )
    ax.set_title(im[im.rfind('/')+1:],fontsize=12)
    ax = plt.imshow(imDict[im])
    plt.xticks([]); plt.yticks([])
plt.show()
```

### Get all the pictures and their respective AU lists¶

Here we define a dictionary *picDict*, wich is used to store the filenames of the original image and the AU images for each face.

In [13]:

```
def makePicDict(picList):
    
    picDict = {}
    for i,ident in enumerate(picList):
        for e,emo in enumerate(picList[i]):
            thisPic = picList[i][e]

            thisName = thisPic[thisPic.find('_')-1:thisPic.rfind('_')]

            myPicList = getFile('../auLabels/',thisName+'*.png')

            picDict[thisName] = {'auList':myPicList,
                                 'picFile':thisPic}
    return picDict
```

In [14]:

```
picDict = makePicDict(picList)
```

In [15]:

```
picDict
```

Out[15]:

```
{'f_ang': {'auList': ['../auLabels/f_ang_AU22+23+25.png',
   '../auLabels/f_ang_AU6.png',
   '../auLabels/f_ang_AU7.png',
   '../auLabels/f_ang_AU9.png'],
  'picFile': '../experiment/app/static/img/f_ang_cut.png'},
 'f_dis': {'auList': ['../auLabels/f_dis_AU10+16+19+25+26.png',
   '../auLabels/f_dis_AU4.png',
   '../auLabels/f_dis_AU6.png',
   '../auLabels/f_dis_AU9.png'],
  'picFile': '../experiment/app/static/img/f_dis_cut.png'},
 'f_fea': {'auList': ['../auLabels/f_fea_AU1.png',
   '../auLabels/f_fea_AU2.png',
   '../auLabels/f_fea_AU20+25.png',
   '../auLabels/f_fea_AU5.png'],
  'picFile': '../experiment/app/static/img/f_fea_cut.png'},
 'f_hap': {'auList': ['../auLabels/f_hap_AU12.png',
   '../auLabels/f_hap_AU25.png',
   '../auLabels/f_hap_AU6.png'],
  'picFile': '../experiment/app/static/img/f_hap_cut.png'},
 'f_ntr': {'auList': ['../auLabels/f_ntr_AU25.png'],
  'picFile': '../experiment/app/static/img/f_ntr_cut.png'},
 'f_sad': {'auList': ['../auLabels/f_sad_AU1+4.png',
   '../auLabels/f_sad_AU15+25.png'],
  'picFile': '../experiment/app/static/img/f_sad_cut.png'},
 'f_sup': {'auList': ['../auLabels/f_sup_AU1.png',
   '../auLabels/f_sup_AU2.png',
   '../auLabels/f_sup_AU25+26+27.png',
   '../auLabels/f_sup_AU5.png'],
  'picFile': '../experiment/app/static/img/f_sup_cut.png'},
 'm_ang': {'auList': ['../auLabels/m_ang_AU25+29.png',
   '../auLabels/m_ang_AU4.png',
   '../auLabels/m_ang_AU7.png'],
  'picFile': '../experiment/app/static/img/m_ang_cut.png'},
 'm_dis': {'auList': ['../auLabels/m_dis_AU10+16+19+25+26.png',
   '../auLabels/m_dis_AU4.png',
   '../auLabels/m_dis_AU6.png',
   '../auLabels/m_dis_AU9.png'],
  'picFile': '../experiment/app/static/img/m_dis_cut.png'},
 'm_fea': {'auList': ['../auLabels/m_fea_AU1.png',
   '../auLabels/m_fea_AU2.png',
   '../auLabels/m_fea_AU20+25.png',
   '../auLabels/m_fea_AU5.png'],
  'picFile': '../experiment/app/static/img/m_fea_cut.png'},
 'm_hap': {'auList': ['../auLabels/m_hap_AU12.png',
   '../auLabels/m_hap_AU25.png',
   '../auLabels/m_hap_AU6.png'],
  'picFile': '../experiment/app/static/img/m_hap_cut.png'},
 'm_ntr': {'auList': ['../auLabels/m_ntr_AU25.png'],
  'picFile': '../experiment/app/static/img/m_ntr_cut.png'},
 'm_sad': {'auList': ['../auLabels/m_sad_AU1+4.png',
   '../auLabels/m_sad_AU15+25.png'],
  'picFile': '../experiment/app/static/img/m_sad_cut.png'},
 'm_sup': {'auList': ['../auLabels/m_sup_AU1.png',
   '../auLabels/m_sup_AU2.png',
   '../auLabels/m_sup_AU25+26+27.png',
   '../auLabels/m_sup_AU5.png'],
  'picFile': '../experiment/app/static/img/m_sup_cut.png'}}
```

make a list with face names, which is sorted in a nice way, so we can use it for looping

In [16]:

```
picSort = []
for entry in picList:
    for subentry in entry:
        picSort.append(subentry[subentry.rfind('/')+1:subentry.rfind('_')])
print picSort
```

```
['f_hap', 'f_sad', 'f_ang', 'f_fea', 'f_dis', 'f_sup', 'f_ntr', 'm_hap', 'm_sad', 'm_ang', 'm_fea', 'm_dis', 'm_sup', 'm_ntr']
```

### show all the tile-assignments of action units¶

In [17]:

```
picDict[picSort[0]]
```

Out[17]:

```
{'auList': ['../auLabels/f_hap_AU12.png',
  '../auLabels/f_hap_AU25.png',
  '../auLabels/f_hap_AU6.png'],
 'picFile': '../experiment/app/static/img/f_hap_cut.png'}
```

## Derive behavioral metric¶

These are the acutal behavioral data, which we now want to apply not to single tiles, but to groups of tiles which all belong to the same action unit. The algorithms to derive the values are esentially the same as in the previous notebooks.

In [18]:

```
df = pd.read_csv(logList[-2],
                 header=0,
                 index_col=[0,1,2]
                )
```

In [19]:

```
df.head()
```

Out[19]:

|  |  |  | time | cumtime | e | i | button | filename | evaluation | stopRT | choiceRT | maskNum | maskList | # |
| --- | --- | --- | --- | --- | --- | --- | --- | --- | --- | --- | --- | --- | --- | --- |
| ident | express | id |  |  |  |  |  |  |  |  |  |  |  |  |
| 0 | 0 | 8 | 2016-03-10 14:14:11 | 223.38838 | 0.0 | 0.0 | hap | img/f\_hap\_cut.png | HIT | 7837.0 | 12277.0 | 8.0 | 11-29-31-37-27-15-5-45 | 8.0 |
|  |  | 14 | 2016-03-10 14:16:22 | 353.89436 | 0.0 | 0.0 | hap | img/f\_hap\_cut.png | HIT | 7182.0 | 9014.0 | 8.0 | 36-5-42-22-30-15-29-13 | 14.0 |
|  |  | 38 | 2016-03-10 14:25:14 | 886.06328 | 0.0 | 0.0 | hap | img/f\_hap\_cut.png | HIT | 5245.0 | 7773.0 | 6.0 | 38-11-21-36-1-41 | 38.0 |
|  |  | 45 | 2016-03-10 14:28:01 | 1052.94504 | 0.0 | 0.0 | hap | img/f\_hap\_cut.png | HIT | 5855.0 | 7047.0 | 6.0 | 10-25-22-30-38-0 | 45.0 |
|  |  | 60 | 2016-03-10 14:33:00 | 1352.16195 | 0.0 | 0.0 | hap | img/f\_hap\_cut.png | HIT | 6383.0 | 8055.0 | 7.0 | 17-25-22-15-12-35-41 | 60.0 |

In [20]:

```
def getWeight(df,thisName,identName,expressName,answer,n=16,identDict=identDict,emoDict=emoDict):
    
    d = {}
    
    # code to number
    ident = identReverse[identName]
    express = emoReverse[expressName]
    
    # get the current condition (face identity and emotion expression)
    thisCondDf = df.ix[ident].ix[express]
    # get only the answers as specified
    thisDf = thisCondDf.loc[thisCondDf['button'] == answer]

    # loop through all trials of that condition
    for i in thisDf.index:
            
        # we get the list of revealed tiles
        maskList = thisDf.ix[i]['maskList']
        thisSplit = maskList.split('-')
        # if the current trial is short enough
        if len(thisSplit) <= n:
                    
            # we apply the above formula to get a weight for the importance of that trial
                    
            ####
            thisFormula = 1. - (len(thisSplit)/float(n) ) 
            ####
                    
            # now each tile of that trial gets that weight assigned, because it was
            # part of this trial
            for num in thisSplit:
                # we try to append, if this does not work, we create the entry
                try:
                    d[num].append(thisFormula)
                except:
                    d[num] = [thisFormula]
                            
            # everyone else not present gets a zero
            # this works by having a list of all possible tile indices (0-47)
            # and removing indeces of all trial that were actually present
            unassigned = range(48)
            for num in thisSplit: unassigned.remove( int(num) )
            # now each tile that was not in the trial gets a zero for having been absent
            for num in unassigned:
                try:
                    d[str(num)].append(0)
                except:
                    d[str(num)] = [0]
    
    # note that if a trial contained an incorrect answer or was not short enough, it is
    # entierly ignored. This might not be desireable, however. E.g. one might argue that
    # if a tile is often part of an incorrect trial, it should get a negative weight
    # so there are certainly many possibilities to make the formula more sophisticated
    
    #average over all trials for each tile 
    outDf = pd.DataFrame( pd.DataFrame( d ).mean() )
    # make the index nice and restructure
    outDf.index = [int(x) for x in outDf.index]
    outDf = outDf.sort_index()
    outDf = outDf.T
    outDf.index = [[thisName],[identDict[ident]],[emoDict[emoReverse[answer]]]]
    outDf = outDf.sortlevel()
    # return a row with all values of the current expression
    return outDf
```

In [21]:

```
getWeight(df,'p001','f','hap','hap')
```

Out[21]:

|  |  |  | 0 | 1 | 2 | 3 | 4 | 5 | 6 | 7 | 8 | 9 | 10 | 11 | 12 | 13 | 14 | 15 | 16 | 17 | 18 | 19 | 20 | 21 | 22 | 23 | 24 | 25 | 26 | 27 | 28 | 29 | 30 | 31 | 32 | 33 | 34 | 35 | 36 | 37 | 38 | 39 | 40 | 41 | 42 | 43 | 44 | 45 | 46 | 47 |
| --- | --- | --- | --- | --- | --- | --- | --- | --- | --- | --- | --- | --- | --- | --- | --- | --- | --- | --- | --- | --- | --- | --- | --- | --- | --- | --- | --- | --- | --- | --- | --- | --- | --- | --- | --- | --- | --- | --- | --- | --- | --- | --- | --- | --- | --- | --- | --- | --- | --- | --- |
| p001 | f | hap | 0.058594 | 0.097656 | 0.03125 | 0.09375 | 0.0 | 0.0625 | 0.0 | 0.128906 | 0.019531 | 0.042969 | 0.089844 | 0.15625 | 0.113281 | 0.144531 | 0.097656 | 0.144531 | 0.03125 | 0.074219 | 0.0 | 0.03125 | 0.0 | 0.207031 | 0.105469 | 0.0 | 0.0 | 0.105469 | 0.035156 | 0.03125 | 0.039062 | 0.113281 | 0.109375 | 0.03125 | 0.097656 | 0.078125 | 0.046875 | 0.125 | 0.132812 | 0.070312 | 0.078125 | 0.0 | 0.042969 | 0.121094 | 0.109375 | 0.15625 | 0.105469 | 0.113281 | 0.0 | 0.035156 |

In [22]:

```
def makePSC(bigWeight):

    # normalize
    byMean = bigWeight.T/bigWeight.T.mean()
    scaledDf = (( ( byMean ).T )*100) -100 # that's the PSC formula
    
    return scaledDf
```

In [23]:

```
makePSC( getWeight(df,'p001','f','hap','hap') )
```

Out[23]:

|  |  |  | 0 | 1 | 2 | 3 | 4 | 5 | 6 | 7 | 8 | 9 | 10 | 11 | 12 | 13 | 14 | 15 | 16 | 17 | 18 | 19 | 20 | 21 | 22 | 23 | 24 | 25 | 26 | 27 | 28 | 29 | 30 | 31 | 32 | 33 | 34 | 35 | 36 | 37 | 38 | 39 | 40 | 41 | 42 | 43 | 44 | 45 | 46 | 47 |
| --- | --- | --- | --- | --- | --- | --- | --- | --- | --- | --- | --- | --- | --- | --- | --- | --- | --- | --- | --- | --- | --- | --- | --- | --- | --- | --- | --- | --- | --- | --- | --- | --- | --- | --- | --- | --- | --- | --- | --- | --- | --- | --- | --- | --- | --- | --- | --- | --- | --- | --- |
| p001 | f | hap | -19.821826 | 33.63029 | -57.238307 | 28.285078 | -100.0 | -14.476615 | -100.0 | 76.391982 | -73.273942 | -41.202673 | 22.939866 | 113.808463 | 55.011136 | 97.772829 | 33.63029 | 97.772829 | -57.238307 | 1.55902 | -100.0 | -57.238307 | -100.0 | 183.296214 | 44.320713 | -100.0 | -100.0 | 44.320713 | -51.893096 | -57.238307 | -46.547884 | 55.011136 | 49.665924 | -57.238307 | 33.63029 | 6.904232 | -35.857461 | 71.046771 | 81.737194 | -3.786192 | 6.904232 | -100.0 | -41.202673 | 65.701559 | 49.665924 | 113.808463 | 44.320713 | 55.011136 | -100.0 | -51.893096 |

In [24]:

```
def getParticipantWeights(df,pName,emo):
    bigDf = pd.DataFrame()
    for ident in identReverse.keys():
        for answer in emoReverse.keys():
            thisDf = makePSC( getWeight(df,pName,ident,emo,answer) )
            bigDf = pd.concat([bigDf,thisDf])
    return bigDf.fillna(0)
```

In [25]:

```
getParticipantWeights(df,'p001','hap')
```

Out[25]:

|  |  |  | 0 | 1 | 2 | 3 | 4 | 5 | 6 | 7 | 8 | 9 | 10 | 11 | 12 | 13 | 14 | 15 | 16 | 17 | 18 | 19 | 20 | 21 | 22 | 23 | 24 | 25 | 26 | 27 | 28 | 29 | 30 | 31 | 32 | 33 | 34 | 35 | 36 | 37 | 38 | 39 | 40 | 41 | 42 | 43 | 44 | 45 | 46 | 47 |
| --- | --- | --- | --- | --- | --- | --- | --- | --- | --- | --- | --- | --- | --- | --- | --- | --- | --- | --- | --- | --- | --- | --- | --- | --- | --- | --- | --- | --- | --- | --- | --- | --- | --- | --- | --- | --- | --- | --- | --- | --- | --- | --- | --- | --- | --- | --- | --- | --- | --- | --- |
| p001 | m | ang | 0.000000 | 0.000000 | 0.000000 | 0.000000 | 0.000000 | 0.000000 | 0.000000 | 0.000000 | 0.000000 | 0.000000 | 0.000000 | 0.000000 | 0.000000 | 0.000000 | 0.000000 | 0.000000 | 0.000000 | 0.000000 | 0.0 | 0.000000 | 0.000000 | 0.000000 | 0.000000 | 0.000000 | 0.000000 | 0.000000 | 0.000000 | 0.000000 | 0.000000 | 0.000000 | 0.000000 | 0.000000 | 0.000000 | 0.000000 | 0.000000 | 0.000000 | 0.000000 | 0.000000 | 0.000000 | 0.000000 | 0.000000 | 0.000000 | 0.000000 | 0.000000 | 0.000000 | 0.000000 | 0.000000 | 0.000000 |
|  |  | fea | 0.000000 | 0.000000 | 0.000000 | 0.000000 | 0.000000 | 0.000000 | 0.000000 | 0.000000 | 0.000000 | 0.000000 | 0.000000 | 0.000000 | 0.000000 | 0.000000 | 0.000000 | 0.000000 | 0.000000 | 0.000000 | 0.0 | 0.000000 | 0.000000 | 0.000000 | 0.000000 | 0.000000 | 0.000000 | 0.000000 | 0.000000 | 0.000000 | 0.000000 | 0.000000 | 0.000000 | 0.000000 | 0.000000 | 0.000000 | 0.000000 | 0.000000 | 0.000000 | 0.000000 | 0.000000 | 0.000000 | 0.000000 | 0.000000 | 0.000000 | 0.000000 | 0.000000 | 0.000000 | 0.000000 | 0.000000 |
|  |  | ntr | 0.000000 | 0.000000 | 0.000000 | 0.000000 | 0.000000 | 0.000000 | 0.000000 | 0.000000 | 0.000000 | 0.000000 | 0.000000 | 0.000000 | 0.000000 | 0.000000 | 0.000000 | 0.000000 | 0.000000 | 0.000000 | 0.0 | 0.000000 | 0.000000 | 0.000000 | 0.000000 | 0.000000 | 0.000000 | 0.000000 | 0.000000 | 0.000000 | 0.000000 | 0.000000 | 0.000000 | 0.000000 | 0.000000 | 0.000000 | 0.000000 | 0.000000 | 0.000000 | 0.000000 | 0.000000 | 0.000000 | 0.000000 | 0.000000 | 0.000000 | 0.000000 | 0.000000 | 0.000000 | 0.000000 | 0.000000 |
|  |  | sad | 0.000000 | 0.000000 | 0.000000 | 0.000000 | 0.000000 | 0.000000 | 0.000000 | 0.000000 | 0.000000 | 0.000000 | 0.000000 | 0.000000 | 0.000000 | 0.000000 | 0.000000 | 0.000000 | 0.000000 | 0.000000 | 0.0 | 0.000000 | 0.000000 | 0.000000 | 0.000000 | 0.000000 | 0.000000 | 0.000000 | 0.000000 | 0.000000 | 0.000000 | 0.000000 | 0.000000 | 0.000000 | 0.000000 | 0.000000 | 0.000000 | 0.000000 | 0.000000 | 0.000000 | 0.000000 | 0.000000 | 0.000000 | 0.000000 | 0.000000 | 0.000000 | 0.000000 | 0.000000 | 0.000000 | 0.000000 |
|  |  | sup | 0.000000 | 0.000000 | 0.000000 | 0.000000 | 0.000000 | 0.000000 | 0.000000 | 0.000000 | 0.000000 | 0.000000 | 0.000000 | 0.000000 | 0.000000 | 0.000000 | 0.000000 | 0.000000 | 0.000000 | 0.000000 | 0.0 | 0.000000 | 0.000000 | 0.000000 | 0.000000 | 0.000000 | 0.000000 | 0.000000 | 0.000000 | 0.000000 | 0.000000 | 0.000000 | 0.000000 | 0.000000 | 0.000000 | 0.000000 | 0.000000 | 0.000000 | 0.000000 | 0.000000 | 0.000000 | 0.000000 | 0.000000 | 0.000000 | 0.000000 | 0.000000 | 0.000000 | 0.000000 | 0.000000 | 0.000000 |
|  |  | hap | -100.000000 | -22.291407 | 91.282690 | -100.000000 | -22.291407 | -100.000000 | -16.313823 | -22.291407 | 37.484433 | -34.246575 | 79.327522 | -64.134496 | -22.291407 | 186.924035 | 55.417186 | -28.268991 | 180.946451 | -58.156912 | -100.0 | 79.327522 | 49.439601 | 228.767123 | 43.462017 | 13.574097 | 145.080946 | -34.246575 | -100.000000 | 7.596513 | 73.349938 | -28.268991 | 55.417186 | -82.067248 | 67.372354 | -64.134496 | -22.291407 | 25.529265 | -100.000000 | -28.268991 | -22.291407 | 37.484433 | -34.246575 | -4.358655 | -64.134496 | -82.067248 | -64.134496 | 73.349938 | -46.201743 | -64.134496 |
|  |  | dis | 0.000000 | 0.000000 | 0.000000 | 0.000000 | 0.000000 | 0.000000 | 0.000000 | 0.000000 | 0.000000 | 0.000000 | 0.000000 | 0.000000 | 0.000000 | 0.000000 | 0.000000 | 0.000000 | 0.000000 | 0.000000 | 0.0 | 0.000000 | 0.000000 | 0.000000 | 0.000000 | 0.000000 | 0.000000 | 0.000000 | 0.000000 | 0.000000 | 0.000000 | 0.000000 | 0.000000 | 0.000000 | 0.000000 | 0.000000 | 0.000000 | 0.000000 | 0.000000 | 0.000000 | 0.000000 | 0.000000 | 0.000000 | 0.000000 | 0.000000 | 0.000000 | 0.000000 | 0.000000 | 0.000000 | 0.000000 |
|  | f | ang | 0.000000 | 0.000000 | 0.000000 | 0.000000 | 0.000000 | 0.000000 | 0.000000 | 0.000000 | 0.000000 | 0.000000 | 0.000000 | 0.000000 | 0.000000 | 0.000000 | 0.000000 | 0.000000 | 0.000000 | 0.000000 | 0.0 | 0.000000 | 0.000000 | 0.000000 | 0.000000 | 0.000000 | 0.000000 | 0.000000 | 0.000000 | 0.000000 | 0.000000 | 0.000000 | 0.000000 | 0.000000 | 0.000000 | 0.000000 | 0.000000 | 0.000000 | 0.000000 | 0.000000 | 0.000000 | 0.000000 | 0.000000 | 0.000000 | 0.000000 | 0.000000 | 0.000000 | 0.000000 | 0.000000 | 0.000000 |
|  |  | fea | 0.000000 | 0.000000 | 0.000000 | 0.000000 | 0.000000 | 0.000000 | 0.000000 | 0.000000 | 0.000000 | 0.000000 | 0.000000 | 0.000000 | 0.000000 | 0.000000 | 0.000000 | 0.000000 | 0.000000 | 0.000000 | 0.0 | 0.000000 | 0.000000 | 0.000000 | 0.000000 | 0.000000 | 0.000000 | 0.000000 | 0.000000 | 0.000000 | 0.000000 | 0.000000 | 0.000000 | 0.000000 | 0.000000 | 0.000000 | 0.000000 | 0.000000 | 0.000000 | 0.000000 | 0.000000 | 0.000000 | 0.000000 | 0.000000 | 0.000000 | 0.000000 | 0.000000 | 0.000000 | 0.000000 | 0.000000 |
|  |  | ntr | 0.000000 | 0.000000 | 0.000000 | 0.000000 | 0.000000 | 0.000000 | 0.000000 | 0.000000 | 0.000000 | 0.000000 | 0.000000 | 0.000000 | 0.000000 | 0.000000 | 0.000000 | 0.000000 | 0.000000 | 0.000000 | 0.0 | 0.000000 | 0.000000 | 0.000000 | 0.000000 | 0.000000 | 0.000000 | 0.000000 | 0.000000 | 0.000000 | 0.000000 | 0.000000 | 0.000000 | 0.000000 | 0.000000 | 0.000000 | 0.000000 | 0.000000 | 0.000000 | 0.000000 | 0.000000 | 0.000000 | 0.000000 | 0.000000 | 0.000000 | 0.000000 | 0.000000 | 0.000000 | 0.000000 | 0.000000 |
|  |  | sad | 0.000000 | 0.000000 | 0.000000 | 0.000000 | 0.000000 | 0.000000 | 0.000000 | 0.000000 | 0.000000 | 0.000000 | 0.000000 | 0.000000 | 0.000000 | 0.000000 | 0.000000 | 0.000000 | 0.000000 | 0.000000 | 0.0 | 0.000000 | 0.000000 | 0.000000 | 0.000000 | 0.000000 | 0.000000 | 0.000000 | 0.000000 | 0.000000 | 0.000000 | 0.000000 | 0.000000 | 0.000000 | 0.000000 | 0.000000 | 0.000000 | 0.000000 | 0.000000 | 0.000000 | 0.000000 | 0.000000 | 0.000000 | 0.000000 | 0.000000 | 0.000000 | 0.000000 | 0.000000 | 0.000000 | 0.000000 |
|  |  | sup | 0.000000 | 0.000000 | 0.000000 | 0.000000 | 0.000000 | 0.000000 | 0.000000 | 0.000000 | 0.000000 | 0.000000 | 0.000000 | 0.000000 | 0.000000 | 0.000000 | 0.000000 | 0.000000 | 0.000000 | 0.000000 | 0.0 | 0.000000 | 0.000000 | 0.000000 | 0.000000 | 0.000000 | 0.000000 | 0.000000 | 0.000000 | 0.000000 | 0.000000 | 0.000000 | 0.000000 | 0.000000 | 0.000000 | 0.000000 | 0.000000 | 0.000000 | 0.000000 | 0.000000 | 0.000000 | 0.000000 | 0.000000 | 0.000000 | 0.000000 | 0.000000 | 0.000000 | 0.000000 | 0.000000 | 0.000000 |
|  |  | hap | -19.821826 | 33.630290 | -57.238307 | 28.285078 | -100.000000 | -14.476615 | -100.000000 | 76.391982 | -73.273942 | -41.202673 | 22.939866 | 113.808463 | 55.011136 | 97.772829 | 33.630290 | 97.772829 | -57.238307 | 1.559020 | -100.0 | -57.238307 | -100.000000 | 183.296214 | 44.320713 | -100.000000 | -100.000000 | 44.320713 | -51.893096 | -57.238307 | -46.547884 | 55.011136 | 49.665924 | -57.238307 | 33.630290 | 6.904232 | -35.857461 | 71.046771 | 81.737194 | -3.786192 | 6.904232 | -100.000000 | -41.202673 | 65.701559 | 49.665924 | 113.808463 | 44.320713 | 55.011136 | -100.000000 | -51.893096 |
|  |  | dis | 0.000000 | 0.000000 | 0.000000 | 0.000000 | 0.000000 | 0.000000 | 0.000000 | 0.000000 | 0.000000 | 0.000000 | 0.000000 | 0.000000 | 0.000000 | 0.000000 | 0.000000 | 0.000000 | 0.000000 | 0.000000 | 0.0 | 0.000000 | 0.000000 | 0.000000 | 0.000000 | 0.000000 | 0.000000 | 0.000000 | 0.000000 | 0.000000 | 0.000000 | 0.000000 | 0.000000 | 0.000000 | 0.000000 | 0.000000 | 0.000000 | 0.000000 | 0.000000 | 0.000000 | 0.000000 | 0.000000 | 0.000000 | 0.000000 | 0.000000 | 0.000000 | 0.000000 | 0.000000 | 0.000000 | 0.000000 |

## Combining The AU assigments and the tile metrics¶

In [26]:

```
def writeMetric(thisBig,p,ident,express,answer,picDict=picDict,thisCoord=thisCoord):
    
    facePic = ident+'_'+express
    auList = picDict[facePic]['auList']
    
    metricDf = thisBig.ix[p].ix[ident].ix[answer]

    # transforming the coordinates to indices from 0 to 47
    cCodes = {}
    for c,coord in enumerate(thisCoord):
        cCodes[ c ] = coord

    # dict to write to
    auDict = {float(np.nan):[]}
    
    # tracker for tiles belonging to no au
    notNan = []
    
    for au in auList:

        auDict[au] = []
        
        # mapping of coordinates to AUs
        d = makeBinAUs( makeAUTable(auList) ).to_dict()
        
        # looping through the metrics
        for key in metricDf.to_dict():
            thisCoord = d[au][cCodes[int(key)]]
            # get the metric
            thisMetric = metricDf.to_dict()[key]
            if d[au][tuple(cCodes[int(key)])] == 1:
                # adding value to 
                auDict[au].append( thisMetric)

                # keep track that this is not a nan
                notNan.append(key)

    # do that for the remaining nans
    for key in metricDf.to_dict():
        if key not in notNan:
            thisCoord = d[au][cCodes[int(key)]]
            # get the metric
            thisMetric = metricDf.to_dict()[key]
            # adding value to 
            auDict[float(np.nan)].append( thisMetric)

    # transform to df
    auDf = pd.DataFrame(index=[p])
    
    for entry in auDict:
        auDf[entry] = np.mean(auDict[entry])

    # cleaning up columns names
    cleanCols = []
    for e in auDf.columns:
        if type(e) == str:
            cleanCols.append(e[e.rfind('_')+1:e.rfind('.')] )
        else:
            cleanCols.append(e)

    auDf.columns = cleanCols
    return auDf
```

In [27]:

```
def writeAllMetrics(p,ident,express):
    
    df = pd.read_csv(p,
                     header=0,
                     index_col=[0,1,2]
                    )
    pName = 'p'+ ('00'+ p[p.find('0')+1:p.rfind('.')])[-3:]
    thisBig = getParticipantWeights(df,pName,express)
    bigDf = pd.DataFrame()
    for answer in emoReverse.keys():
        thisDf = writeMetric(thisBig,pName,ident,express,answer)
        thisDf.columns = [[answer]*len(thisDf.columns),thisDf.columns]
        bigDf = pd.concat([bigDf,thisDf],axis=1)
    return bigDf
```

In [28]:

```
writeAllMetrics(logList[-1],'f','hap')
```

Out[28]:

|  | ang | | | | fea | | | | ntr | | | | sad | | | | sup | | | | hap | | | | dis | | | |
| --- | --- | --- | --- | --- | --- | --- | --- | --- | --- | --- | --- | --- | --- | --- | --- | --- | --- | --- | --- | --- | --- | --- | --- | --- | --- | --- | --- | --- |
|  | NaN | AU12 | AU25 | AU6 | NaN | AU12 | AU25 | AU6 | NaN | AU12 | AU25 | AU6 | NaN | AU12 | AU25 | AU6 | NaN | AU12 | AU25 | AU6 | NaN | AU12 | AU25 | AU6 | NaN | AU12 | AU25 | AU6 |
| p096 | 0.0 | 0.0 | 0.0 | 0.0 | 0.0 | 0.0 | 0.0 | 0.0 | 20.376176 | -100.0 | -100.0 | 30.909091 | 0.0 | 0.0 | 0.0 | 0.0 | 0.0 | 0.0 | 0.0 | 0.0 | -26.812104 | 104.081633 | 69.09621 | 20.816327 | 0.0 | 0.0 | 0.0 | 0.0 |

In [29]:

```
def writeAllParticipants(logList,ident,express):
    bigDf = pd.DataFrame()
    for logFile in logList:
        thisDf = writeAllMetrics(logFile,ident,express)
        bigDf = pd.concat([bigDf,thisDf])
    
    return bigDf
```

### This will take brutally long, so we save this once as csv's and later load from those¶

In [30]:

```
def realBig(logList):
    for ident in identReverse.keys():
        for emo in emoReverse.keys():
            print "working on",ident,emo,'...'
            bigAU = writeAllParticipants(logList,ident,emo)
            bigAU.to_csv('../outputs/actionUnits_'+ident+'_'+emo+'.csv')
```

In [31]:

```
#### realBig(logList)
```

### Taking the nan (no Action Unit) as baseline¶

In [32]:

```
bigAU = pd.read_csv('../outputs/actionUnits_m_ang.csv',
                    index_col=[0],header=[0,1])
bigAU.head()
```

Out[32]:

|  | ang | | | | fea | | | | ntr | | | | sad | | | | sup | | | | hap | | | | dis | | | |
| --- | --- | --- | --- | --- | --- | --- | --- | --- | --- | --- | --- | --- | --- | --- | --- | --- | --- | --- | --- | --- | --- | --- | --- | --- | --- | --- | --- | --- |
|  | nan | AU25+29 | AU7 | AU4 | nan | AU25+29 | AU7 | AU4 | nan | AU25+29 | AU7 | AU4 | nan | AU25+29 | AU7 | AU4 | nan | AU25+29 | AU7 | AU4 | nan | AU25+29 | AU7 | AU4 | nan | AU25+29 | AU7 | AU4 |
| p001 | 0.000000 | 0.000000 | 0.000000 | 0.000000 | -3.879850 | 60.486322 | 2.12766 | -41.641337 | -19.327731 | 46.938776 | -14.285714 | 46.938776 | -38.423029 | 104.255319 | 78.723404 | 82.370821 | 0.0 | 0.0 | 0.0 | 0.0 | 0.0 | 0.0 | 0.0 | 0.0 | -9.243697 | 95.918367 | -14.285714 | -51.020408 |
| p002 | -6.348282 | -6.647808 | 33.663366 | 37.482320 | 9.803922 | 52.380952 | -100.00000 | -100.000000 | 14.168798 | -34.409938 | -100.000000 | -34.409938 | -8.359133 | 28.320802 | 33.333333 | 12.280702 | 0.0 | 0.0 | 0.0 | 0.0 | 0.0 | 0.0 | 0.0 | 0.0 | -5.882353 | 71.428571 | 0.000000 | -42.857143 |
| p003 | -19.191919 | 26.984127 | 85.858586 | 66.233766 | 15.508021 | -37.662338 | -100.00000 | -37.662338 | 0.000000 | 0.000000 | 0.000000 | 0.000000 | 0.000000 | 0.000000 | 0.000000 | 0.000000 | 0.0 | 0.0 | 0.0 | 0.0 | 0.0 | 0.0 | 0.0 | 0.0 | -7.073716 | 12.839060 | 112.658228 | 21.518987 |
| p004 | -20.204604 | 26.708075 | 173.913043 | 71.428571 | 0.000000 | 0.000000 | 0.00000 | 0.000000 | 0.000000 | 0.000000 | 0.000000 | 0.000000 | -19.327731 | 193.877551 | -100.000000 | -100.000000 | 0.0 | 0.0 | 0.0 | 0.0 | 0.0 | 0.0 | 0.0 | 0.0 | 0.000000 | 0.000000 | 0.000000 | 0.000000 |
| p005 | -13.459621 | 14.285714 | 69.491525 | 51.089588 | 0.000000 | 0.000000 | 0.00000 | 0.000000 | 0.000000 | 0.000000 | 0.000000 | 0.000000 | 19.457014 | 5.494505 | -100.000000 | -100.000000 | 0.0 | 0.0 | 0.0 | 0.0 | 0.0 | 0.0 | 0.0 | 0.0 | 0.000000 | 0.000000 | 0.000000 | 0.000000 |

In [33]:

```
def baselineCorrection(df,cond):
    # select the condition
    thisCorr = df[cond]
    diffDf = pd.DataFrame()

    # for all action units
    for actionUnit in thisCorr.columns:
        # we subtract the baseline
        thisDiff = thisCorr[actionUnit]-thisCorr[u'nan']
        diffDf[actionUnit] = thisDiff
    diffDf = diffDf.drop('nan',axis=1)
    
    # restore the original structure of the multicolumns
    diffDf.columns = [[cond]*len(diffDf.columns),diffDf.columns]
    
    return diffDf
```

Example:

In [34]:

```
bigAU['ang'].head()
```

Out[34]:

|  | nan | AU25+29 | AU7 | AU4 |
| --- | --- | --- | --- | --- |
| p001 | 0.000000 | 0.000000 | 0.000000 | 0.000000 |
| p002 | -6.348282 | -6.647808 | 33.663366 | 37.482320 |
| p003 | -19.191919 | 26.984127 | 85.858586 | 66.233766 |
| p004 | -20.204604 | 26.708075 | 173.913043 | 71.428571 |
| p005 | -13.459621 | 14.285714 | 69.491525 | 51.089588 |

In [35]:

```
baselineCorrection(bigAU,'ang').head()
```

Out[35]:

|  | ang | | |
| --- | --- | --- | --- |
|  | AU25+29 | AU7 | AU4 |
| p001 | 0.000000 | 0.000000 | 0.000000 |
| p002 | -0.299526 | 40.011648 | 43.830602 |
| p003 | 46.176046 | 105.050505 | 85.425685 |
| p004 | 46.912678 | 194.117647 | 91.633175 |
| p005 | 27.745335 | 82.951147 | 64.549210 |

In [36]:

```
def makeBaseline(bigAU):
    baselineDf = pd.DataFrame()
    for face in bigAU.columns.levels[0]:
        thisDf = baselineCorrection(bigAU,face)
        baselineDf = pd.concat([baselineDf,thisDf],axis=1)
    
    return baselineDf
```

In [37]:

```
baselineDf = makeBaseline(bigAU)
```

In [38]:

```
baselineDf['ang'].head()
```

Out[38]:

|  | AU25+29 | AU7 | AU4 |
| --- | --- | --- | --- |
| p001 | 0.000000 | 0.000000 | 0.000000 |
| p002 | -0.299526 | 40.011648 | 43.830602 |
| p003 | 46.176046 | 105.050505 | 85.425685 |
| p004 | 46.912678 | 194.117647 | 91.633175 |
| p005 | 27.745335 | 82.951147 | 64.549210 |

## Basic plotting¶

In [39]:

```
#http://stackoverflow.com/a/214657
def rgb2hex(rgb):
    return '#%02x%02x%02x' % rgb
```

In [40]:

```
def makeActionUnitPlot(ident,emo,ans):
    
    face = ident+'_'+emo
    
    bigAU = pd.read_csv('../outputs/actionUnits_'+face+'.csv',
                    index_col=[0],header=[0,1])
    
    baselineDf = makeBaseline(bigAU)
    
    # select the face condition
    thisDf = baselineDf[ans]
    
    colNames = thisDf.columns
    # get number of participants (with values)
    n = int(thisDf.describe().ix['count'][-1])
    
    # get all metrics into a df which we sort descending
    plotDf = pd.DataFrame()
    plotDf['mean'] = thisDf.mean()
    #print float( thisDf.mean() ), float( thisDf.describe().ix['mean'] )
    plotDf['ci'] = thisDf.std()/np.sqrt(n)*1.96
 
    plotDf['color'] = [rgb2hex(getColor('../auLabels/'+face+'_'+colName+'.png')) for colName in colNames]
    plotDf = plotDf.sort_values(by="mean",ascending=False)
   
    # plot this bar at the correct position and using the correct color
    # this is done in a 
    plt.xticks(np.arange(len(plotDf.index))+0.45,plotDf.index,rotation=45,fontsize=12)
    #plt.ylim(-10,100)
    plt.axhline(0,color='k')
    plt.title(ans,fontsize=20)
    im = plt.bar(range(len(plotDf.index)) ,
            plotDf['mean'],
            yerr=plotDf['ci'],
            color= plotDf['color'],
            ecolor='k')
    
    return im
```

In [41]:

```
makeActionUnitPlot('m','ang','dis');
```

## Visualize it all¶

### get the images with the hand-drawn AUs¶

In [42]:

```
myPaintList =  []
for fGender in ['f','m']:
    thisList = []
    for fEmo in myLabels.values()[:-1]:
        thisFace = getFile('../auLabels/auVisualisation/',fGender+'_'+fEmo+'*.png')[-1]
        thisList.append(thisFace)
    myPaintList.append(thisList)
```

### plot all confusions and the face that was rated¶

In [43]:

```
def makePlot(ident,emo):
    fig = plt.figure(figsize=(16,10))
    
    ax = plt.subplot(2,4,1)
    im=Image.open(myPaintList[identReverse[ident]][emoReverse[emo]],'r')
    ax.imshow(im)
    ax.set_yticks([]); ax.set_xticks([])
    
    count = 2
    for answer in ['hap','sad','ang','fea','dis','sup','ntr']:#emoReverse.keys():
        ax = plt.subplot(2,4,count)
        ax = makeActionUnitPlot(ident,emo,answer);
        plt.ylim(-35,105)
        plt.yticks(range(-20,130,20),[str(x)+'%' for x in range(-20,121,20) ])
        sns.despine()
        count+=1
    plt.tight_layout()
    plt.savefig('../auConfusions/confPlot_'+ident+'_'+emo+'.png',dpi=300)
    return fig
```

save all to file

In [44]:

```
for i in ['f','m']:
    for e in ['hap','sad','ang','fea','dis','sup']:
        makePlot(i,e);
```

get a list of all saved files

In [45]:

```
auPics = getFile('../auConfusions/','confPlot*.png')
```

### Make an interactive plot¶

In [46]:

```
# Interactive plots for static html notebooks; using ipywidgets by Jake Vanderplas  
# https://github.com/jakevdp/ipywidgets-static

from ipywidgets_static import StaticInteract, RangeWidget, RadioWidget
```

Define function for interactive plot

In [47]:

```
def iPlot(i,e):
    fig = plt.figure(figsize=(20, 12))
    im = Image.open('../auConfusions/confPlot_'+i+'_'+e+'.png')
    plt.imshow(im)
    sns.despine(left=True,bottom=True)
    plt.xticks([]);plt.yticks([])
    return fig
```

Show interactive plot

In [48]:

```
StaticInteract(iPlot,
               i=RadioWidget(['f','m']),
               e=RadioWidget(['hap','sad','ang','fea','dis','sup'])
              )
```

Out[48]:

**e:** hap:  sad:  ang:  fea:  dis:  sup: 
  
**i:** f:  m:
