# Supplementary material for: Mapping the emotional face. How individual face parts contribute to successful emotion recognition
Source: PLoS One. 2017 May 11;12(5):e0177239. doi: 10.1371/journal.pone.0177239 (PMC5426715; doi:10.1371/journal.pone.0177239)
Supplement: S1 Code — (ZIP) [file pone.0177239.s003.zip › app/templates/base.html]

Face Experiment


- Instruktionen
- FAQ
- Kontakt

{% block content %}{% endblock %}


## Instruktionen:

  

Du sollst maskierte Gesichter hinsichtlich ihres Emotionsausdrucks bewerten.
Die Maske verschwindet nach und nach, wodurch das das darunter liegende Gesicht sichtbar wird.  
  
Du sollst **möglichst schnell** entscheiden, welchen Emotionsausdruck das gezeigte Gesicht hat.
So bald Du glaubst, dass Du das Emotionsausdruck erkannt hast, drückst Du auf den STOP-Knopf
Danach kannst Du Dich für eine von sieben Antwortmöglichkeiten entscheiden:  
Freude, Angst, Ärger, Ekel, Trauer, Überraschung und Neutral.

#### Kontakt:

Bei Fragen kannst Du Dich gerne an martin.wegrzyn@uni-bielefeld.de wenden.  
  
**Wir wünschen weiterhin viel Erfolg beim Experiment!**

DIESES FENSTER SCHLIESSEN

## Mögliche Probleme:

  
  
**Problem:** Die Darstellung und/oder das Verhalten des Experiments ist komisch!  
**Lösung:** Verwende eine aktuelle Version von Mozilla Firefox als Browser. Erlaube Cookies und JavaScript.  
  
**Problem:** Ich mache viel Fehler!  
**Lösung:** Das Experiment ist relativ schwer. Mach Dir keine Sorgen, wenn es mal nicht so gut klappt, das ist völlig normal.  
  
**Problem:** Das Experiment reagiert mit Verzörgerung  
**Lösung:** Das ist normal; es gibt eine Wartezeit von 1/2 Sekunde zwischen den Druchgängen  
  
**Problem:** Ich habe falsch geklickt und möchte den Durchgang wiederholen  
**Lösung:** Es zählt immer die erste Reaktion. Wenn Du einmal einen Fehler gemacht hast, arbeite einfach weiter  
  
  
  

#### Kontakt:

Bei Fragen kannst Du Dich gerne an martin.wegrzyn@uni-bielefeld.de wenden.  
  
**Wir wünschen weiterhin viel Erfolg beim Experiment!**  
  
 


DIESES FENSTER SCHLIESSEN

## Kontakt:

  
Bei Fragen kannst Du Dich gerne an martin.wegrzyn@uni-bielefeld.de wenden.  
  
**Wir wünschen weiterhin viel Erfolg beim Experiment!**  
  
 


DIESES FENSTER SCHLIESSEN
