# Supplementary material for: Mapping the emotional face. How individual face parts contribute to successful emotion recognition
Source: PLoS One. 2017 May 11;12(5):e0177239. doi: 10.1371/journal.pone.0177239 (PMC5426715; doi:10.1371/journal.pone.0177239)
Supplement: S1 Code — (ZIP) [file pone.0177239.s003.zip › app/templates/coursecredit.html]

{% extends "base.html" %}
{% block content %}
  
  

# Persönlicher Code

  

#### **Danke fürs Mitmachen!**

  

Du hast {{ runs }} Blöcke des Experiments absolviert und dafür nicht mehr als {{ time }} Minuten gebraucht.
Damit wir Dir Deine Versuchspersonenstunden gutschreiben können, trage Dich im Forschungsportal für die Studie ein und sende bitte **Deine Vp-Nummer und foldenden Code**  mit Hilfe des unten stehenden Formularfeldes an uns.

  

#### **Dein Code: {{ code }}**

  

Dieser Code wird einmalig generiert und **lässt keinerlei Rückschluss darauf zu, welche Ergebnisdatei von Dir stammt**. Er sagt wie viele Durchgänge Du absolviert hast und wie viel Zeit Du dafür benötigt hast.
  
Außerdem wird die von Dir benötigte Zeit in 10 Minunten-Schritten aufgerundet, so dass man die Zeitangabe aus dem Code nicht mit den Zeiten aus den Ergebnisdateien abgleichen kann.
Da Du zum verschicken der Vp-Nummer und des Codes auch nicht Deine eigene Email-Adresse verwenden musst, bleiben Deine **Daten komplett anonym**.  
Wir empfehlen Dir, den Code zur Sicherheit auch noch mal für Dich selbst irgendwo abzuspeichern.
  
  
Wenn Du magst, kannst Du das Formular auch dazu verwenden, um uns eine kurze Rückmeldung zu geben. Uns interessiert wie Du das Experiment findest, ob es irgendwelche Probleme beim Bearbeiten gab und was wir noch verbessern könnten. Danke.

{% if success == False %}

{% for message in form.message.errors %}
  

{{ message }}

{% endfor %}
  

{{ form.hidden\_tag() }}
{{ form.message.label }}  
{{ form.message(class\_="form-control") }}
  
  

{{ form.submit(class="btn btn-primary") }}

{% else %}

{{ success }}

{% endif %}
  
  
  
  
{% endblock %}
