# Supplementary material for: Mapping the emotional face. How individual face parts contribute to successful emotion recognition
Source: PLoS One. 2017 May 11;12(5):e0177239. doi: 10.1371/journal.pone.0177239 (PMC5426715; doi:10.1371/journal.pone.0177239)
Supplement: S1 Code — (ZIP) [file pone.0177239.s003.zip › app/templates/demographics.html]

{% extends "base.html" %}
{% block content %}

## Demographische Angaben

Nachfolgend möchten wir Dich bitten, ein paar allgemeine Informationen zu Deiner Person zu machen. Diese Angaben sind freiwillig, aber wir freuen uns, wenn Du möchlichst viele Fragen beantwortest. Die Fragen sind allgemein gehalten, so dass sie keinen Rückschluss auf Deine Person ermöglichen. So musst Du z.B. nicht Dein exaktes Alter angeben, sondern lediglich eine von 5 Altersgruppen auswählen.

{% for message in form.location.errors %}

{{ message }}

{% endfor %}
{% for message in form.age.errors %}

{{ message }}

{% endfor %}
{% for message in form.gender.errors %}

{{ message }}

{% endfor %}
{% for message in form.occupation.errors %}

{{ message }}

{% endfor %}
{% for message in form.advertisement.errors %}

{{ message }}

{% endfor %}
  
  

{{ form.hidden\_tag() }}

{{ form.location.label }}  
{{ form.location }}

  

{{ form.age.label }}  
{{ form.age }}

  

{{ form.gender.label }}  
{{ form.gender }}

{{ form.occupation.label }}  
{{ form.occupation }}

  

{{ form.advertisement.label }}  
{{ form.advertisement }}

  
  

{{ form.submit(class="btn btn-primary") }}

{% endblock %}
