# Supplementary material for: Mapping the emotional face. How individual face parts contribute to successful emotion recognition
Source: PLoS One. 2017 May 11;12(5):e0177239. doi: 10.1371/journal.pone.0177239 (PMC5426715; doi:10.1371/journal.pone.0177239)
Supplement: S1 Code — (ZIP) [file pone.0177239.s003.zip › app/templates/experiment.html]

{% extends "base.html" %}
{% block content %}


{% if i >= length %}
{%if j == blocks %}

### Prima, Du hast alle Durchgänge des Experiments absolviert. Wenn Du magst, kannst Du Dir nun eine detaillierte und nach Versuchsbedingungen aufgeschlüsselte Übersicht Deiner Ergebnisse anzeigen lassen.

{% else %}

### Prima, Du hast einen kompletten Durchgang des Experiments absolviert. Wenn Du magst, kannst Du eine kleine Pause einlegen und danach weiter machen.

{% endif %}

Dein bisheriges Ergebnis im Vergleich zu allen anderen Personen siehst Du unten. Du kannst versuchen, ob es Dir gelingt, beim nächsten Durchgang noch besser abzuschneiden. Je mehr Durchgänge Du absolvierst, desto genauer ist der Schätzer Deiner Leistung.

  
  
  
{%if j == blocks %}


{% else %}

{% endif %}

{% elif i >= 0 %}
  
  
  
  


  

{% elif i < 0 %}

# Nun geht es los.

### Du siehst gleich nacheinander Gesichter die maskiert sind. Versuche für jedes Gesicht eine möglichst schnelle und korrekte Entscheidung zu treffen. Viel Erfolg.

{% endif %}
  
  

{% if i < 0 %}


{% endif %}
{% if i>=0 and i < length %}

{% endif %}
{% endblock %}
