# Supplementary material for: Mapping the emotional face. How individual face parts contribute to successful emotion recognition
Source: PLoS One. 2017 May 11;12(5):e0177239. doi: 10.1371/journal.pone.0177239 (PMC5426715; doi:10.1371/journal.pone.0177239)
Supplement: S1 Code — (ZIP) [file pone.0177239.s003.zip › app/templates/impressum.html]

{% extends "startbase.html" %}
{% block content %}


Toggle navigation

cogut.org

- Experimente
- Unser Ansatz
- Historie
- Kontakt


# Impressum

### Angaben gemäß § 5 TMG:

Martin Wegrzyn  
Universität Bielefeld  
Fakultät für Psychologie und Sportwissenschaft  
Abteilung für Psychologie  
Universitätsstraße 25  
D-33615 Bielefeld  
  

### Kontakt:

Telefon: 0521 106-4527  
Fax: 0521 106-154454  
Mail: martin.wegrzyn@uni-bielefeld.de  
  

### Haftungsausschluss:

  

#### Haftung für Inhalte

Die Inhalte unserer Seiten wurden mit größter Sorgfalt erstellt. Für die Richtigkeit, Vollständigkeit und Aktualität der Inhalte können wir jedoch keine Gewähr übernehmen. Als Diensteanbieter sind wir gemäß § 7 Abs.1 TMG für eigene Inhalte auf diesen Seiten nach den allgemeinen Gesetzen verantwortlich. Nach §§ 8 bis 10 TMG sind wir als Diensteanbieter jedoch nicht verpflichtet, übermittelte oder gespeicherte fremde Informationen zu überwachen oder nach Umständen zu forschen, die auf eine rechtswidrige Tätigkeit hinweisen. Verpflichtungen zur Entfernung oder Sperrung der Nutzung von Informationen nach den allgemeinen Gesetzen bleiben hiervon unberührt. Eine diesbezügliche Haftung ist jedoch erst ab dem Zeitpunkt der Kenntnis einer konkreten Rechtsverletzung möglich. Bei Bekanntwerden von entsprechenden Rechtsverletzungen werden wir diese Inhalte umgehend entfernen.

#### Haftung für Links

Unser Angebot enthält Links zu externen Webseiten Dritter, auf deren Inhalte wir keinen Einfluss haben. Deshalb können wir für diese fremden Inhalte auch keine Gewähr übernehmen. Für die Inhalte der verlinkten Seiten ist stets der jeweilige Anbieter oder Betreiber der Seiten verantwortlich. Die verlinkten Seiten wurden zum Zeitpunkt der Verlinkung auf mögliche Rechtsverstöße überprüft. Rechtswidrige Inhalte waren zum Zeitpunkt der Verlinkung nicht erkennbar. Eine permanente inhaltliche Kontrolle der verlinkten Seiten ist jedoch ohne konkrete Anhaltspunkte einer Rechtsverletzung nicht zumutbar. Bei Bekanntwerden von Rechtsverletzungen werden wir derartige Links umgehend entfernen.

#### Urheberrecht

Die durch die Seitenbetreiber erstellten Inhalte und Werke auf diesen Seiten unterliegen dem deutschen Urheberrecht. Die Vervielfältigung, Bearbeitung, Verbreitung und jede Art der Verwertung außerhalb der Grenzen des Urheberrechtes bedürfen der schriftlichen Zustimmung des jeweiligen Autors bzw. Erstellers. Downloads und Kopien dieser Seite sind nur für den privaten, nicht kommerziellen Gebrauch gestattet. Soweit die Inhalte auf dieser Seite nicht vom Betreiber erstellt wurden, werden die Urheberrechte Dritter beachtet. Insbesondere werden Inhalte Dritter als solche gekennzeichnet. Sollten Sie trotzdem auf eine Urheberrechtsverletzung aufmerksam werden, bitten wir um einen entsprechenden Hinweis. Bei Bekanntwerden von Rechtsverletzungen werden wir derartige Inhalte umgehend entfernen.

#### Datenschutz

Die Nutzung unserer Webseite ist in der Regel ohne Angabe personenbezogener Daten möglich. Soweit auf unseren Seiten personenbezogene Daten (beispielsweise Name, Anschrift oder eMail-Adressen) erhoben werden, erfolgt dies, soweit möglich, stets auf freiwilliger Basis. Diese Daten werden ohne Ihre ausdrückliche Zustimmung nicht an Dritte weitergegeben.
Wir weisen darauf hin, dass die Datenübertragung im Internet (z.B. bei der Kommunikation per E-Mail) Sicherheitslücken aufweisen kann. Ein lückenloser Schutz der Daten vor dem Zugriff durch Dritte ist nicht möglich.
Der Nutzung von im Rahmen der Impressumspflicht veröffentlichten Kontaktdaten durch Dritte zur Übersendung von nicht ausdrücklich angeforderter Werbung und Informationsmaterialien wird hiermit ausdrücklich widersprochen. Die Betreiber der Seiten behalten sich ausdrücklich rechtliche Schritte im Falle der unverlangten Zusendung von Werbeinformationen, etwa durch Spam-Mails, vor.

  
  
  
  
  
  
{% endblock %}
