# Supplementary material for: Mapping the emotional face. How individual face parts contribute to successful emotion recognition
Source: PLoS One. 2017 May 11;12(5):e0177239. doi: 10.1371/journal.pone.0177239 (PMC5426715; doi:10.1371/journal.pone.0177239)
Supplement: S1 Code — (ZIP) [file pone.0177239.s003.zip › app/templates/index.html]

{% extends "startbase.html" %}
{% block content %}


Toggle navigation

cogut.org

- Experimente
- Unser Ansatz
- Ressourcen
- Kontakt
- a


willkommen auf cogut.org!

KOGNITIVE EXPERIMENTE.  
ONLINE.

Mach bei einem Experiment mit!


## Experimente

### Eine Übersicht der aktuell laufenden Experimente.

#### GESICHTER-QUIZ

Versuche, möglichst schnell den Ausdruck eines
maskierten Gesichts zu erraten.

## Unser Ansatz

### Wie wir arbeiten. Was wir erreichen möchten.

#### Experimente die motivieren.

Wir möchten dass unsere ProbandInnen sich nicht langweilen. Du entscheidest, wie lange Du mitmachen willst und bekommst sofortige Rückmeldung über deine Ergebnisse.

#### Große Stichproben.

Wir möchten möglichst viele Daten erheben und nach Möglichkeit niemals mit dem Erheben aufhören müssen. Kreuzvalidierungen und die Prädiktion zukünftiger Ergebnisse soll so ermöglicht werden.

#### Aufeinander aufbauen.

Wir möchten eine Platform anbieten, auf der man allen Code, alle Daten und das komplette Stimulusmaterial frei einsehen, teilen, verändern und für die eigene Forschung nutzen kann.

## Ressourcen

### Code und Daten.

#### Code auf GitHub.

Hier steht der ganze Code zur Verfügung, mit dem cogut programmiert wurde.

#### Download von Daten.

Hier kann man alle Daten herunterladen, die bisher erhoben wurden.

#### Publikationen.

Hier gibt es eine Liste von Publikationen, die auf cogut-Daten aufbauen.


## Kontakt

  
  

### Bei Fragen, Anregungen und Kritik schreib uns eine Email.


- cogut nutzt folgende Software:
- Python
- Flask
- Tornado
- Werkzeug
- Jinja
- NumPy
- Pandas
- matplotlib
- Seaborn
- Bootstrap

- Weitere Infos:
- Code und Daten auf GitHub
- cogut ist freie Software
- Impressum


## GESICHTER-QUIZ

Wahrnehmungsstudie. Dauer ca. 15-60 Minuten (frei wählbar).

  

Du siehst einzelne Gesichter, die einen bestimmten Ausdruck zeigen und zunächst völlig verdeckt sind.
Nach und nach werden immer mehr Partien des Gesichts aufgedeckt.
Versuche, möglichst schnell den Gesichtsausdruck zu erkennen!

**Du willst mitmachen?** Dann klicke auf Start:

START

SCHLIESSEN

{% endblock %}
