# Supplementary material for: Mapping the emotional face. How individual face parts contribute to successful emotion recognition
Source: PLoS One. 2017 May 11;12(5):e0177239. doi: 10.1371/journal.pone.0177239 (PMC5426715; doi:10.1371/journal.pone.0177239)
Supplement: S1 Code — (ZIP) [file pone.0177239.s003.zip › app/templates/instruction.html]

{% extends "base.html" %}
{% block content %}

### Liebe Probandin, lieber Proband,

vielen Dank für Deine Teilnahme an unserem Experiment. Die Studie „Unterscheidung von Emotionsausdrücken“ untersucht Wahrnehmungsprozesse, die bei der Verarbeitung von Gesichtern stattfinden. Um dieser Frage nachzugehen, werden Dir eine Reihe von Gesichtern präsentiert, die Du hinsichtlich ihres Emotionsausdrucks bewerten sollst.
Die Gesichter sind durch eine Maske verdeckt, die nach und nach verschwindet und das darunter liegende Gesicht sichtbar werden lässt. Deine Aufgabe wird es sein, möglichst schnell zu entscheiden, welchen Emotionsausdruck das gezeigte Gesicht hat. Dabei sind sowohl Geschwindigkeit als auch Richtigkeit der Antwort wichtig. Du solltest in jedem Fall versuchen, so schnell wie möglich zu antworten. So bald Du glaubst, dass Du das Emotionsausdruck erkannt hast, drückst Du auf den Knopf der unter dem Gesicht eingeblendet ist. Danach kannst Du Dich für eine von sieben Antwortmöglichkeiten entscheiden (Freude, Angst, Ärger, Ekel, Trauer, Überraschung und Neutral).
Ein Balken am unteren Bildschirmrand zeigt Dir an, wie weit Du in dem aktuellen Durchgang schon gekommen bist. Insgesamt hat das Experiment zwei Durchgänge und dauert ca. 50 Minuten.
Nach jedem Durchgang des Experiments wirst Du Rückmeldung darüber bekommen, wie gut Du bisher abgeschnitten hast. Dabei wird die Geschwindigkeit gegen die Anzahl richtiger Antworten abgetragen. Wenn das Experiment beendet ist, erhältst eine detaillierte Rückmeldung Deiner Leistung im Vergleich mit allen anderen Personen, die bisher mitgemacht haben. Die entsprechenden Ergebnisgrafiken werden Dir auch als Download zur Verfügung stehen.

### Einverständniserklärung

Hiermit erkläre ich mich bereit, an einer Studie der AE Affektive Neuropsychologie der Universität Bielefeld teilzunehmen. Ich bin über Inhalt, Zweck und Umfang der Untersuchung informiert worden. **Die Teilnahme ist freiwillig. Ich kann jederzeit und ohne Angabe von Gründen meine Einwilligung widerrufen, ohne dass dies mit Nachteilen für mich verbunden wäre.** Die Versuchsleiter verpflichten sich, die Anonymität der erhobenen Daten zu gewährleisten und keinerlei Daten zu sammeln, die meine Identifikation ermöglichen könnten. Ich selbst werde in keinem der Textfelder meinen Namen eintragen oder sonstige zu meiner Identifikation beitragenden Angaben machen. Bei der anonymisierten Aufbewahrung der Daten wird eine Identifikation meiner Person nicht möglich sein. Dies bedeutet auch, dass meine individuellen Daten im Nachhinein nicht gelöscht werden können.
Ich erkläre mich bereit, dass die Ergebnisse in anonymisierter Form gespeichert und unter einer freien Lizenz (CC0; weitere Infos) zugänglich gemacht werden.  
Bei Fragen kann ich mich jederzeit an die Versuchsleiter wenden. Für weitere Rückfragen kann ich eine Mail an martin.wegrzyn@uni-bielefeld.de schicken.

  

{% for message in form.major.errors %}
  

{{ message }}

{% endfor %}
{% for message in form.consent.errors %}

{{ message }}

  
  
{% endfor %}
{{ form.hidden\_tag() }}
{{ form.major.label }}
{{ form.major }}
  
{{ form.consent.label }}
{{ form.consent }}
  
  
  

{{ form.submit(class="btn btn-primary btn-lg btn-block") }}

  
  
  

{% endblock %}
