# Supplementary material for: Mapping the emotional face. How individual face parts contribute to successful emotion recognition
Source: PLoS One. 2017 May 11;12(5):e0177239. doi: 10.1371/journal.pone.0177239 (PMC5426715; doi:10.1371/journal.pone.0177239)
Supplement: S1 Code — (ZIP) [file pone.0177239.s003.zip › app/templates/pub.html]

{% extends "startbase.html" %}
{% block content %}


Toggle navigation

cogut.org

- Experimente
- Unser Ansatz
- Historie
- Kontakt


in Arbeit...

  
  
  
  
  
  
{% endblock %}
